# Supplementary material for: Copine-6 is a TRPM3 escort protein controlling the sensitivity of sensory neurons to noxious heat
Source: EMBO J. 2025 Jun 19;44(15):4222–51. doi: 10.1038/s44318-025-00487-0 (PMC12317139; doi:10.1038/s44318-025-00487-0)
Supplement: Supplementary file 1 — Appendix [file 44318_2025_487_MOESM1_ESM.pdf]

Appendix for "Copine-6 is a TRPM3 escort protein controlling the sensitivity of sensory neurons to noxious heat"

Table of contents:

Appendix Figure S1………………………………………………… 2

Appendix Figure S2………………………………………………… 3

Appendix Figure S3………………………………………………… 4

Appendix Figure S4………………………………………………… 6

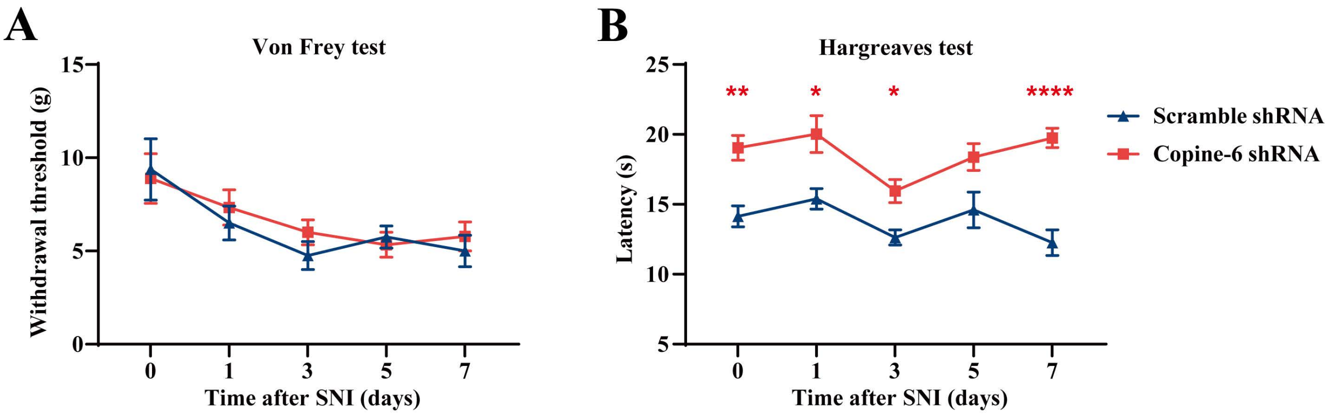

Appendix Figure S1. Knockdown of Copine-6 in DRG decreased thermal hypersensitivity in a rat neuropathic pain model. Rats were DRG-injected with either Copine-6 shRNA or Scramble shRNA control virions (both at $1x10^{13}$ viral genomes/mL; 2 µL). Spared nerve injury (SNI) model (see Methods) was established 35 day after the viral infections and mechanical (Von Frey test, A) and thermal (Hargreaves test, B) sensitivity was monitored; n = 8 rats in Scramble shRNA group and n = 9 rats in Copine-6 shRNA group for both, panels A and B; Data are shown as mean ± SEM; 0 d, ***P* = 0.0036; 1 d, **P* = 0.047; 3 d, **P* = 0.024; 7 d, *****P* < 0.0001 (two-way ANOVA followed by Bonferroni multiple comparisons test).

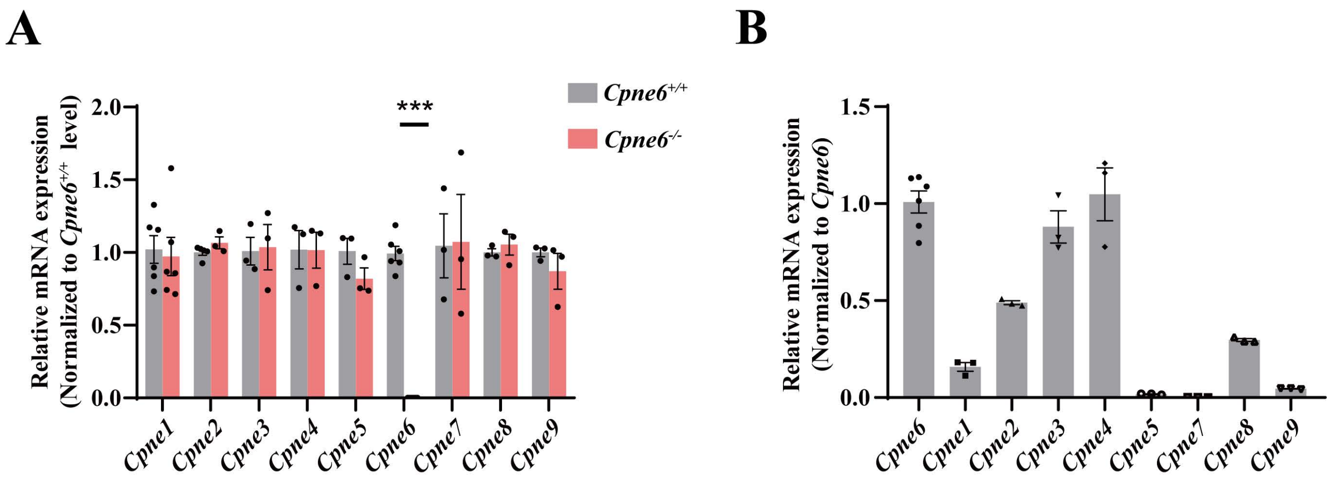

Appendix Figure S2. Expression of CPNE family in the mouse DRG. A, The mRNA expression levels of *Cpne1-9* genes in the DRG of *Cpne6*$^{-/-}$ mice, as compared to *Cpne6*$^{+/+}$ mice. *Cpne6*$^{+/+}$, n = 6, 5, 3, 3, 3, 6, 3, 3, 3; *Cpne6*$^{-/-}$, n = 6, 3, 3, 3, 3, 6, 3, 3, 3; Data are shown as mean ± SEM; ****P* < 0.001 (two-tailed independent t-test). **B,** The mRNA expression levels of *Cpne1-9* genes, relative to *Cpne6*. *Cpne6*, n = 6; *Cpne1-5 and 7-9*, n = 3.

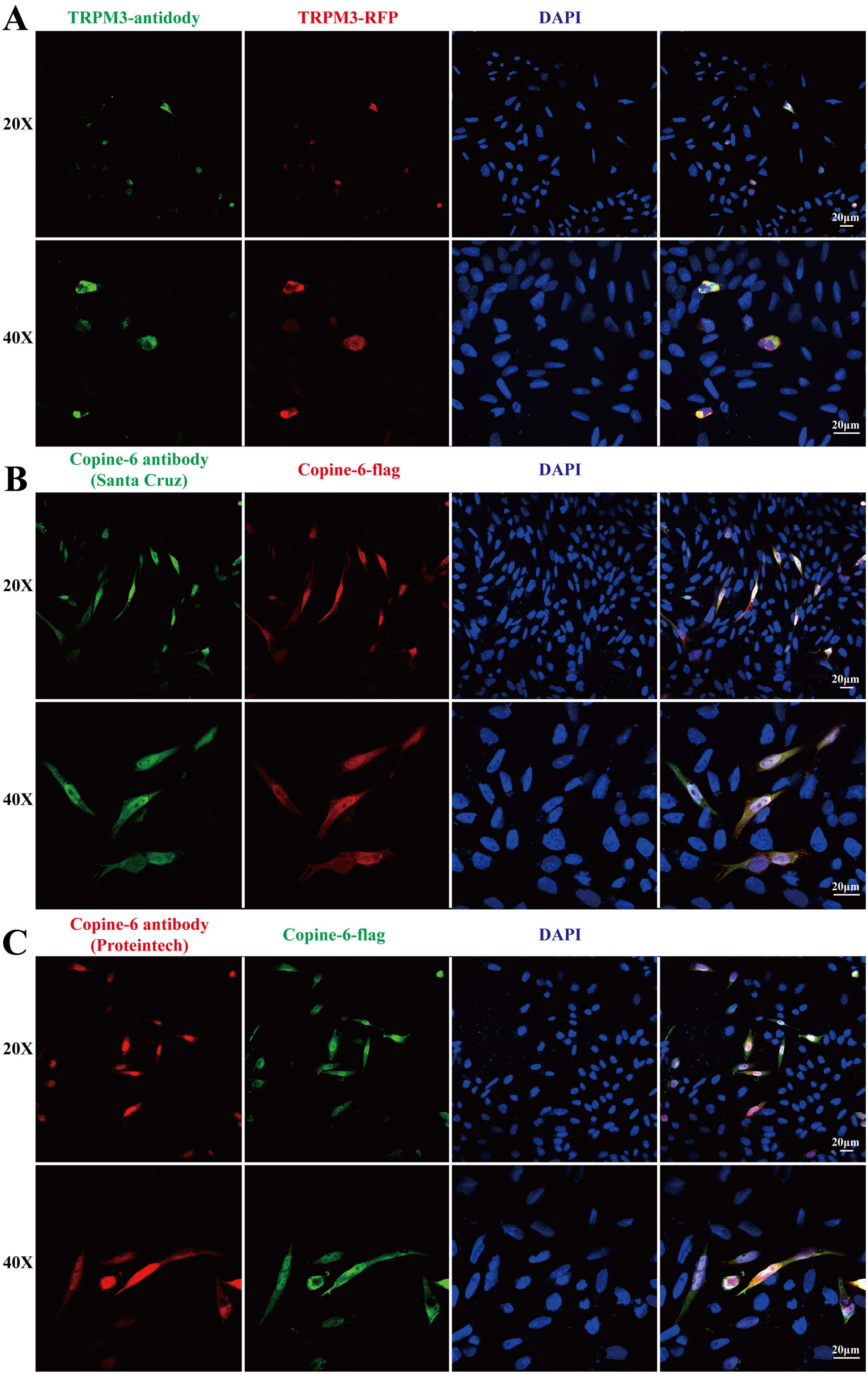
A
TRPM3-antidody
TRPM3-RFP
DAPI
20X
40X
20μm
B
Copine-6 antibody (Santa Cruz)
Copine-6-flag
DAPI
20X
40X
20μm
C
Copine-6 antibody (Proteintech)
Copine-6-flag
DAPI
20X
40X
20μm

Appendix Figure S3. Validation of TRPM3 and Copine-6 antibodies. A, Co-immunostaining of CHO cells transiently overexpressing TRPM3-RFP cDNA with antibodies against TRPM3 (green) and RFP (red); blue – DAPI. Scale bars are 20 μm. **B-C,** Co-immunostaining of CHO cells transiently overexpressing Copine-6-flag-cDNA with antibodies against Copine-6 (B: Santa Cruz, green; C: Proteintech, red) and flag (B: red; C: green); blue – DAPI. Scale bars are 20 μm.

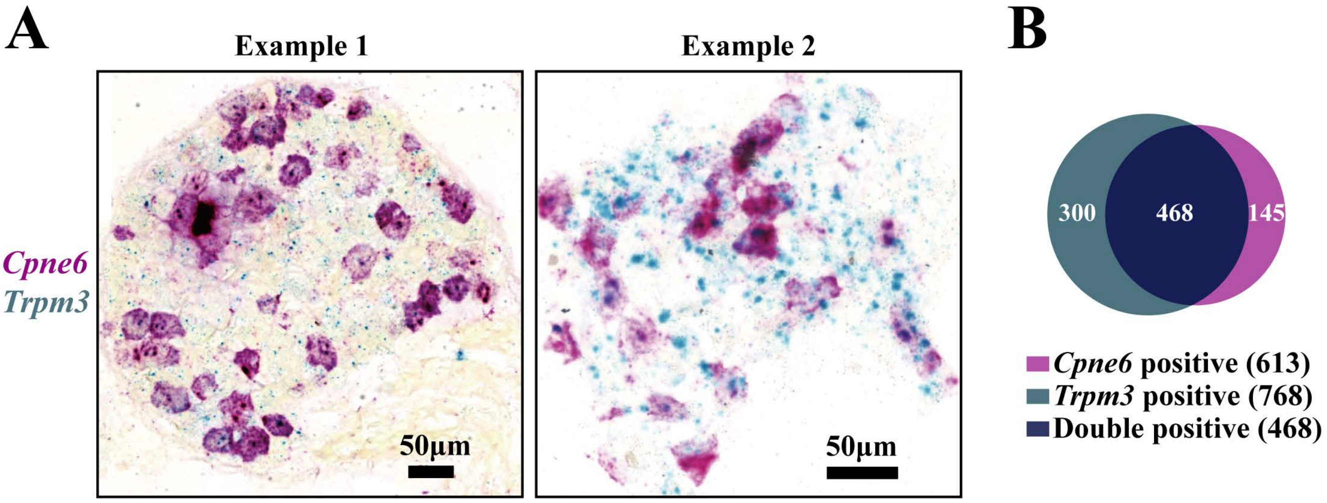

Appendix Figure S4. Co-expression of *Cpne6* and *Trpm3* in rat DRG as determined using RNAscope. A, Brightfield micrographs for co-labelling of DRG sections with RNAscope probes against *Cpne6* (purple) and or *Trpm3* (cyan). Scale bars are 50 µm. **B**, The pie chart quantifying the proportions of DRG neurons expressing *Cpne6* (purple) and *Trpm3* (cyan). Shown in dark blue are the proportions of neurons co-expressing *Cpne6* and *Trpm3*.
