# Supplementary figures and images for: Copine-6 is a TRPM3 escort protein controlling the sensitivity of sensory neurons to noxious heat (part 1 of 2)
Source: EMBO J. 2025 Jun 19;44(15):4222–51. doi: 10.1038/s44318-025-00487-0 (PMC12317139; doi:10.1038/s44318-025-00487-0)

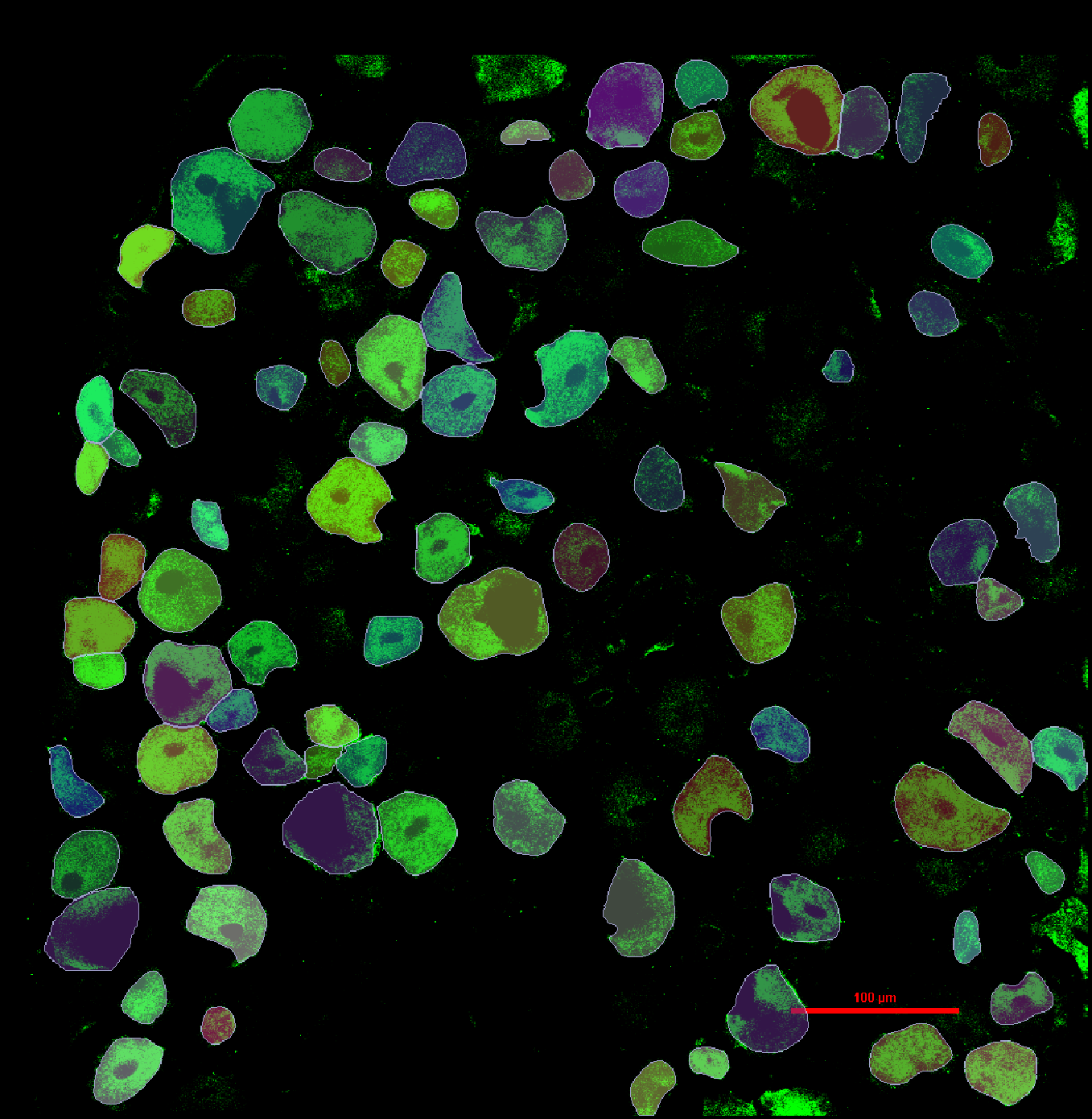

Supplement: Supplementary file 3 — Source data Fig. 1 [file 44318_2025_487_MOESM3_ESM.zip › Figure 1/1A/Cellpose.jpg]

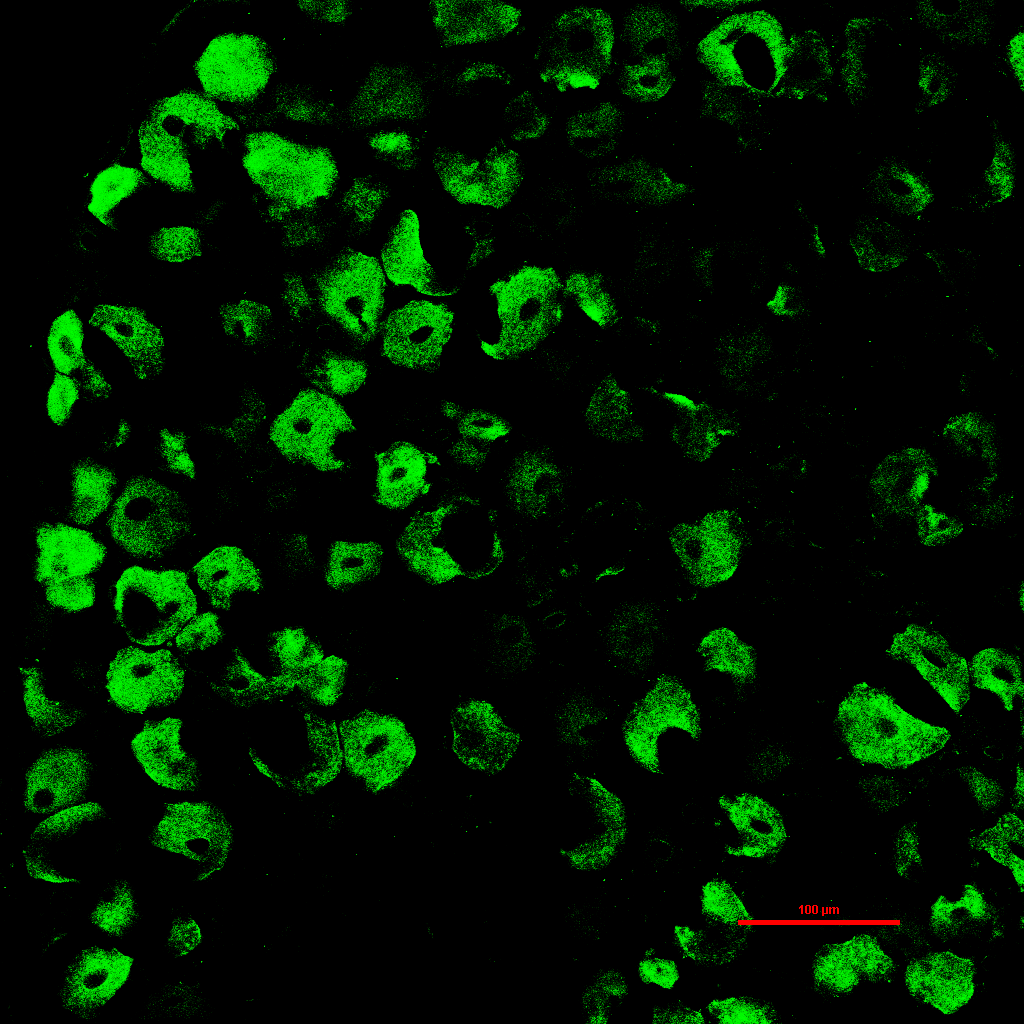

Supplement: Supplementary file 3 — Source data Fig. 1 [file 44318_2025_487_MOESM3_ESM.zip › Figure 1/1A/Copine-6.tif]

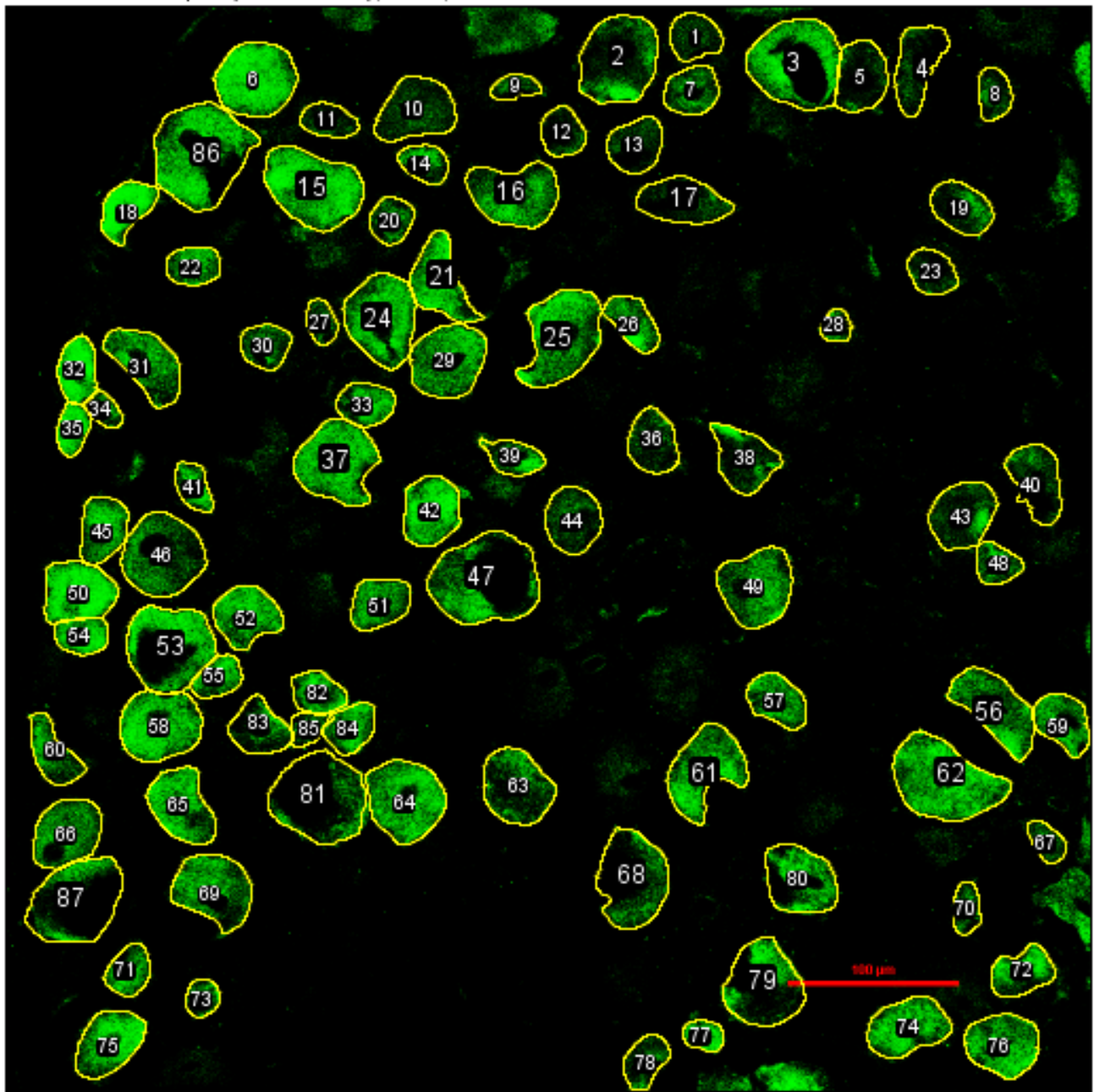

Supplement: Supplementary file 3 — Source data Fig. 1 [file 44318_2025_487_MOESM3_ESM.zip › Figure 1/1A/Image J.jpg]

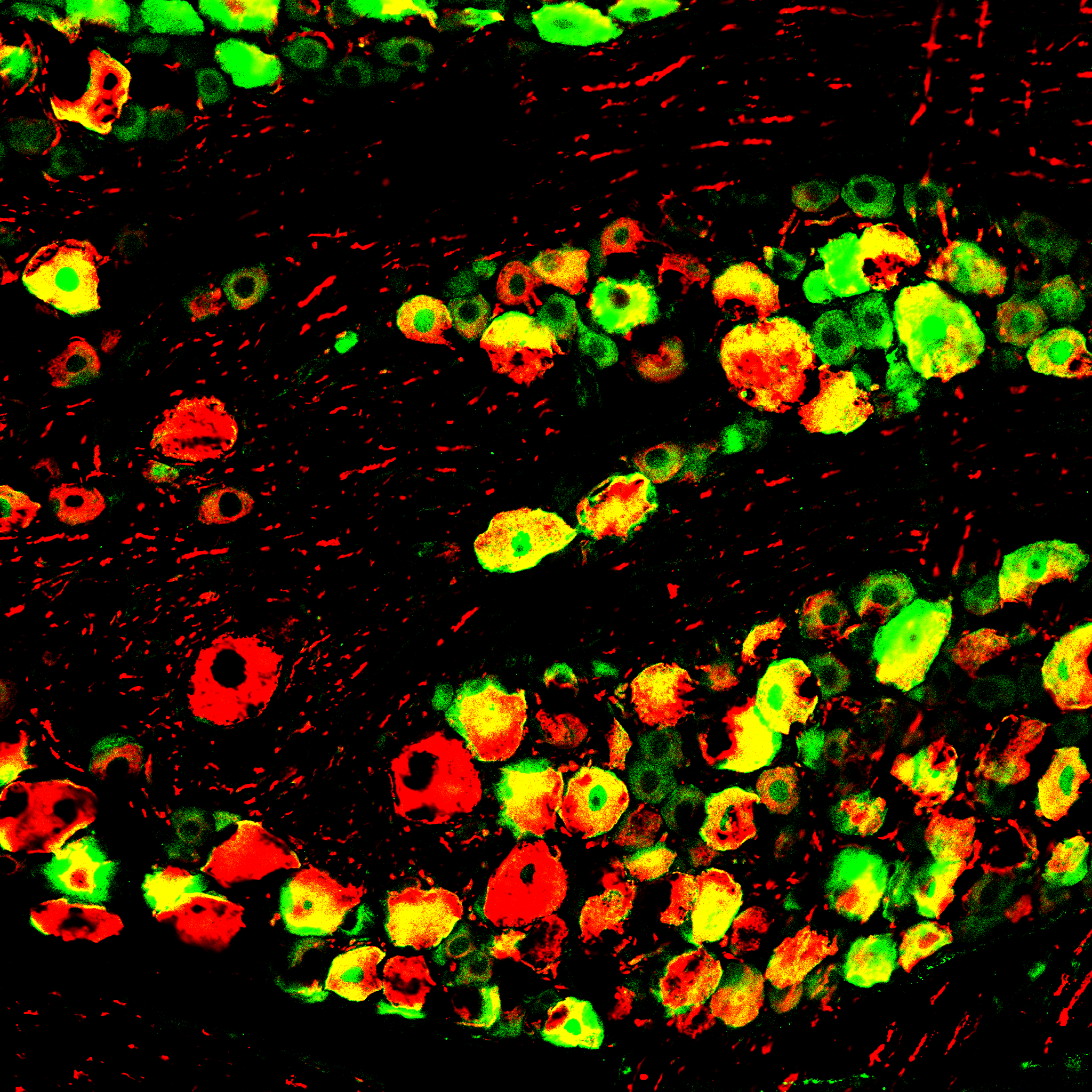

Supplement: Supplementary file 3 — Source data Fig. 1 [file 44318_2025_487_MOESM3_ESM.zip › Figure 1/1B/NF200 merge.tif]

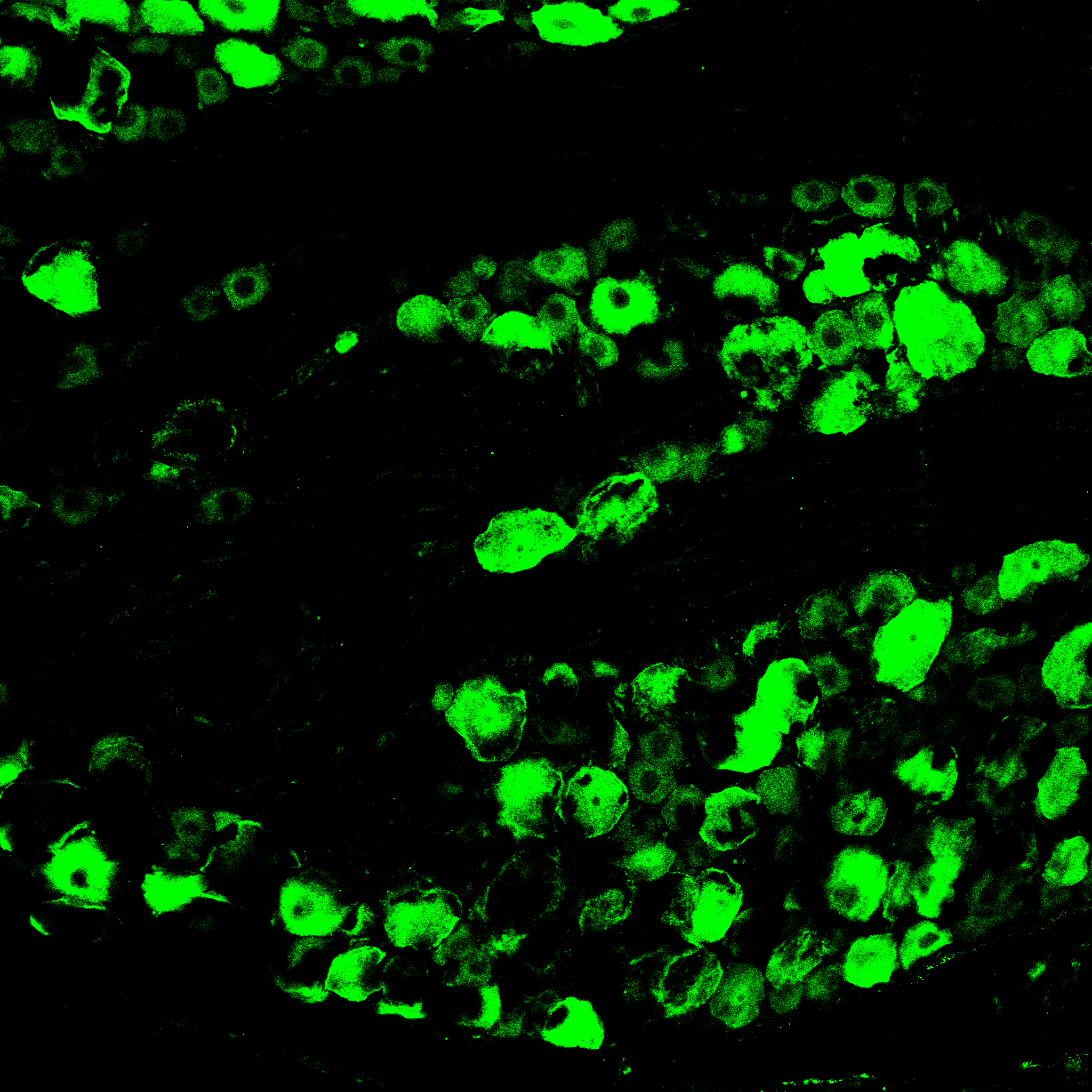

Supplement: Supplementary file 3 — Source data Fig. 1 [file 44318_2025_487_MOESM3_ESM.zip › Figure 1/1B/NF200-Copine-6.tif]

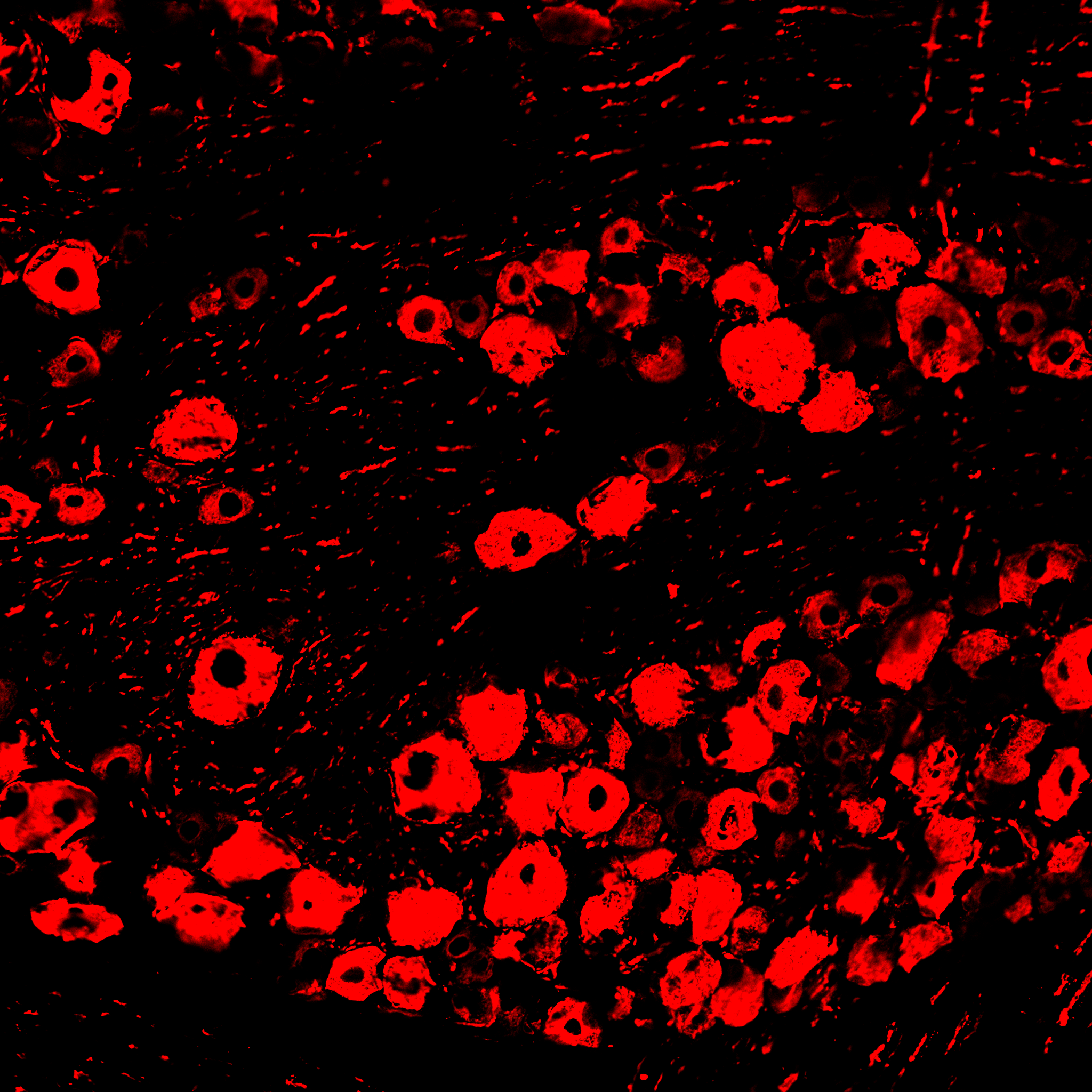

Supplement: Supplementary file 3 — Source data Fig. 1 [file 44318_2025_487_MOESM3_ESM.zip › Figure 1/1B/NF200.tif]

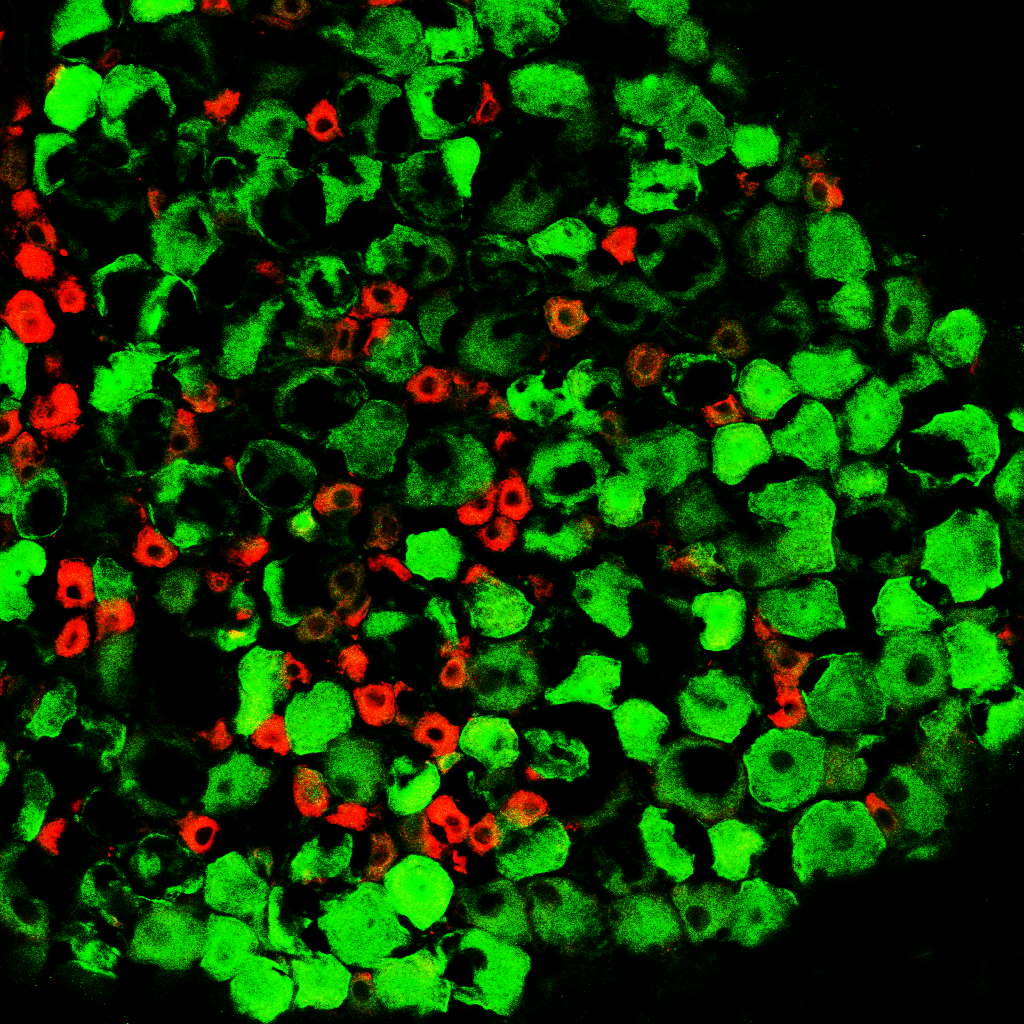

Supplement: Supplementary file 3 — Source data Fig. 1 [file 44318_2025_487_MOESM3_ESM.zip › Figure 1/1B/peripherin merge.tif]

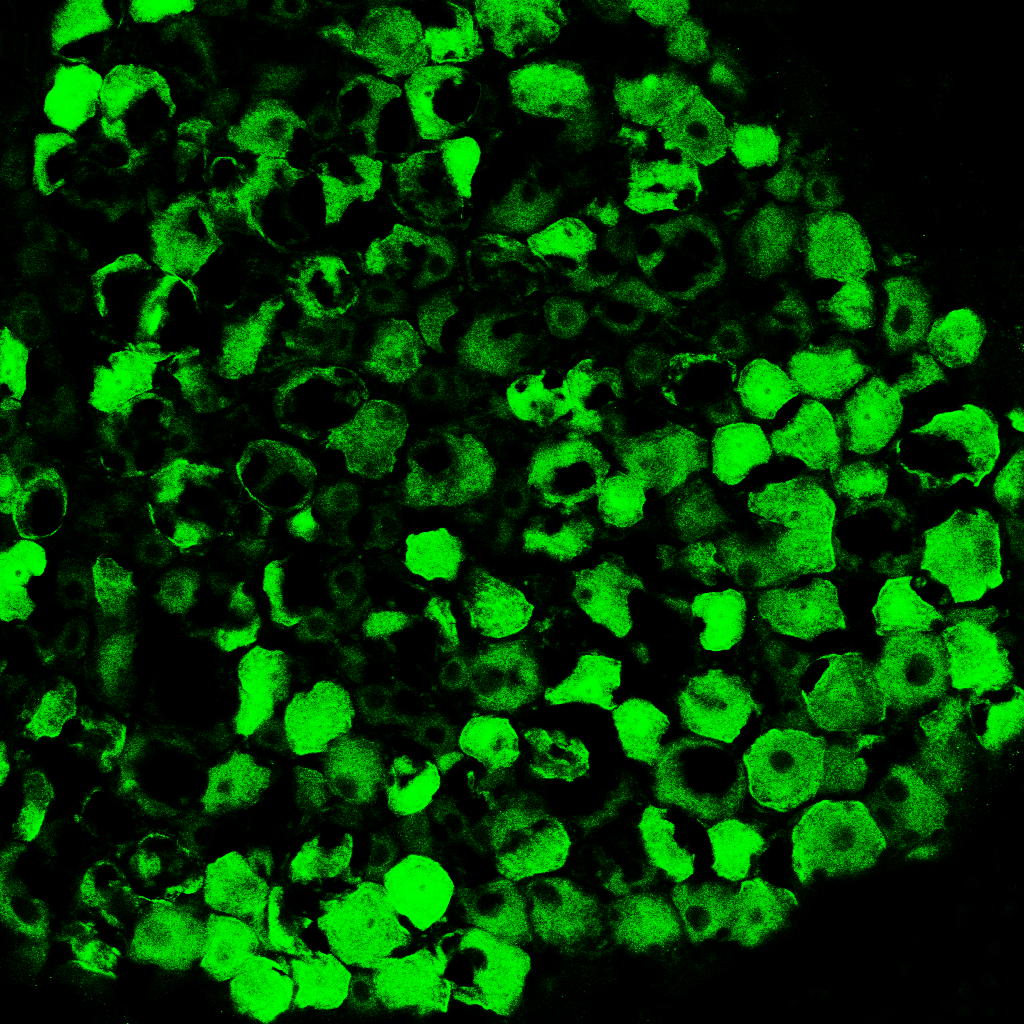

Supplement: Supplementary file 3 — Source data Fig. 1 [file 44318_2025_487_MOESM3_ESM.zip › Figure 1/1B/peripherin-Copine-6.tif]

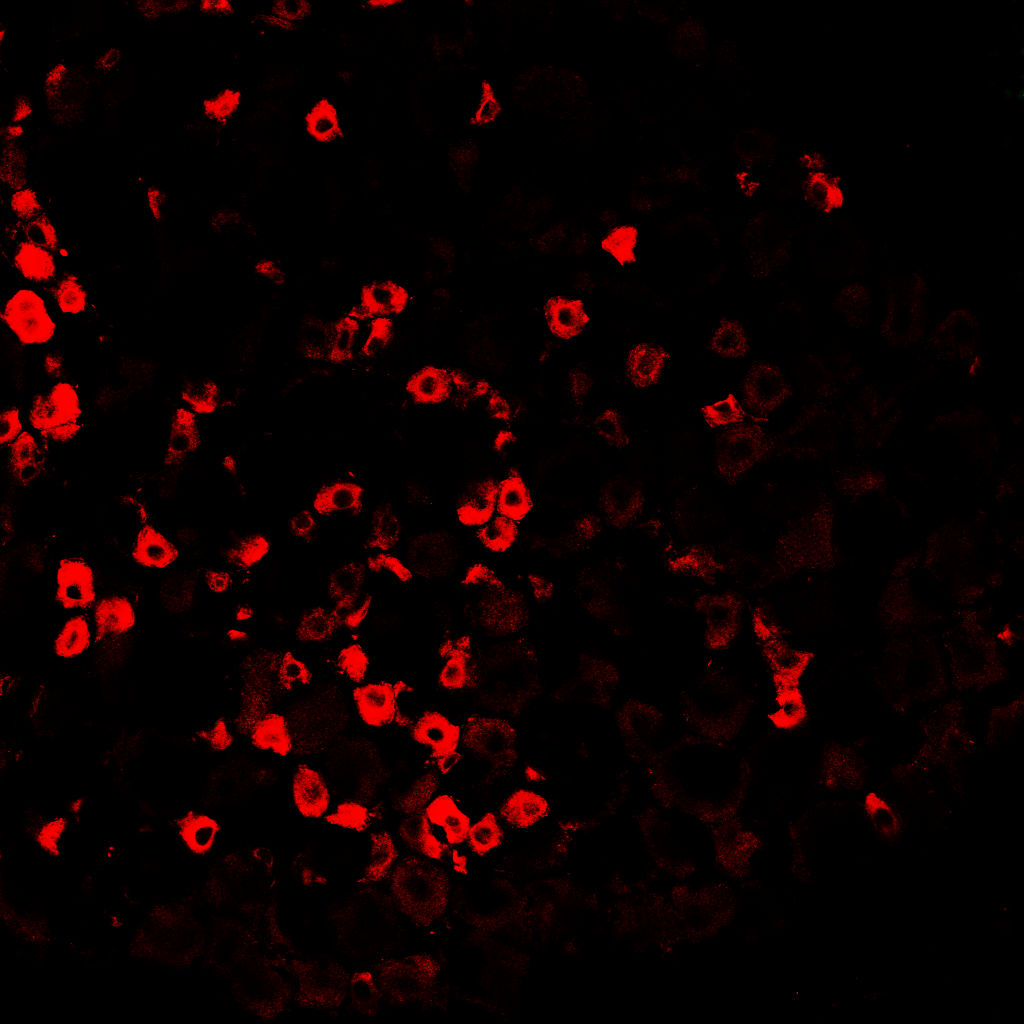

Supplement: Supplementary file 3 — Source data Fig. 1 [file 44318_2025_487_MOESM3_ESM.zip › Figure 1/1B/peripherin.tif]

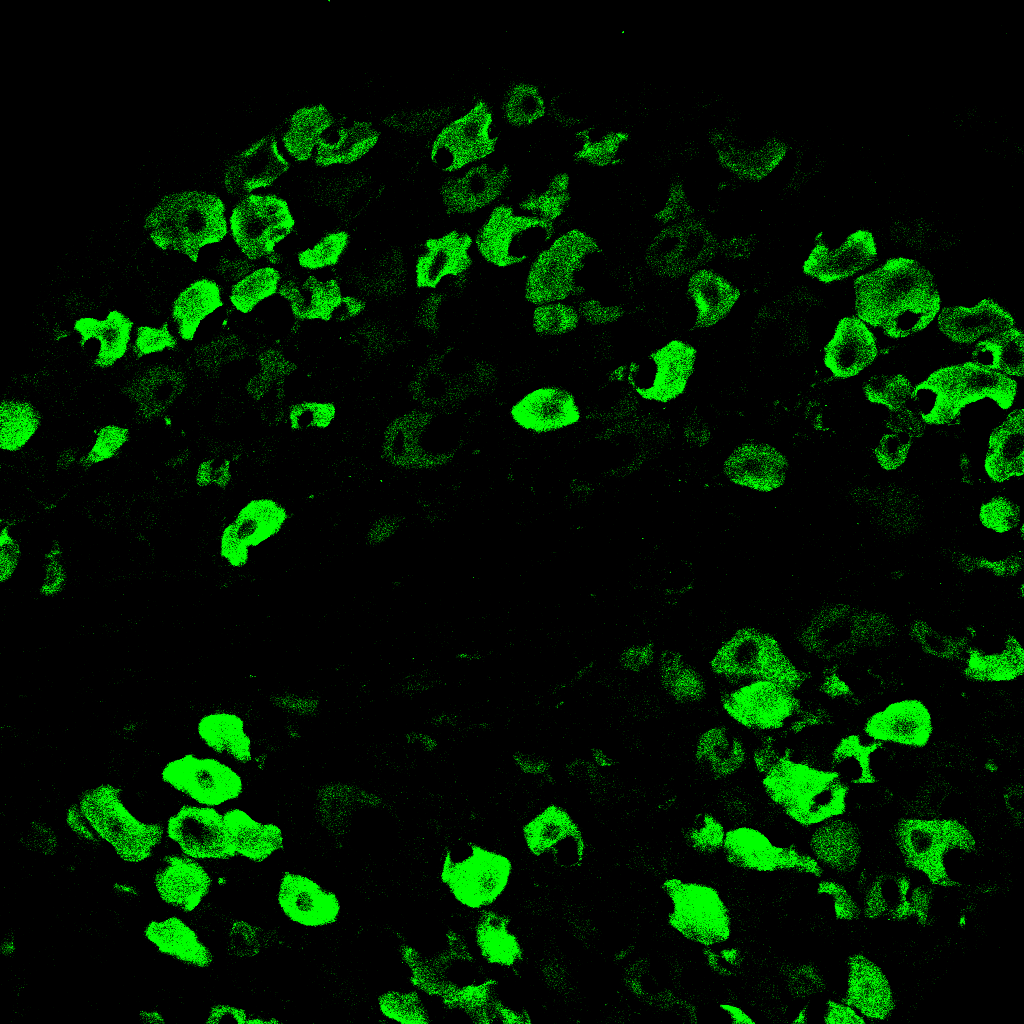

Supplement: Supplementary file 3 — Source data Fig. 1 [file 44318_2025_487_MOESM3_ESM.zip › Figure 1/1C/Copine-6.tif]

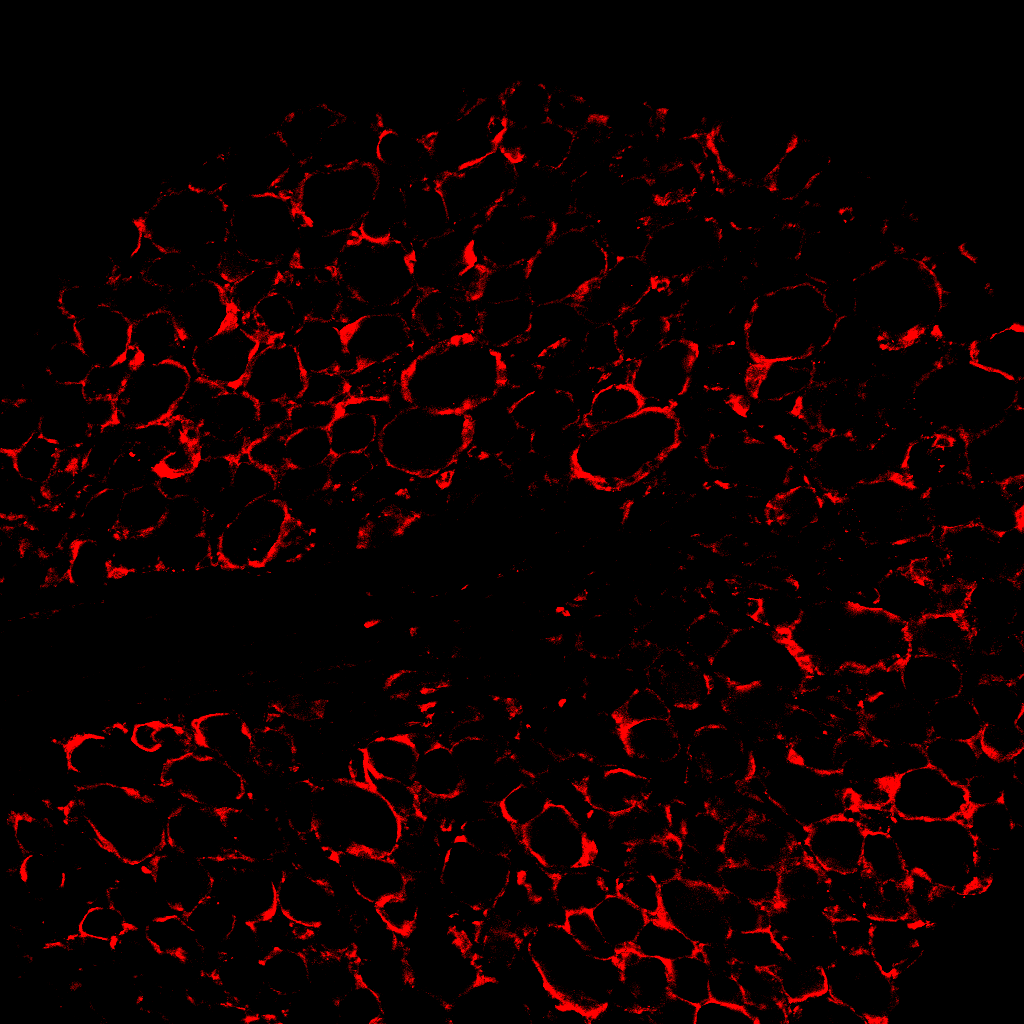

Supplement: Supplementary file 3 — Source data Fig. 1 [file 44318_2025_487_MOESM3_ESM.zip › Figure 1/1C/DBI.tif]

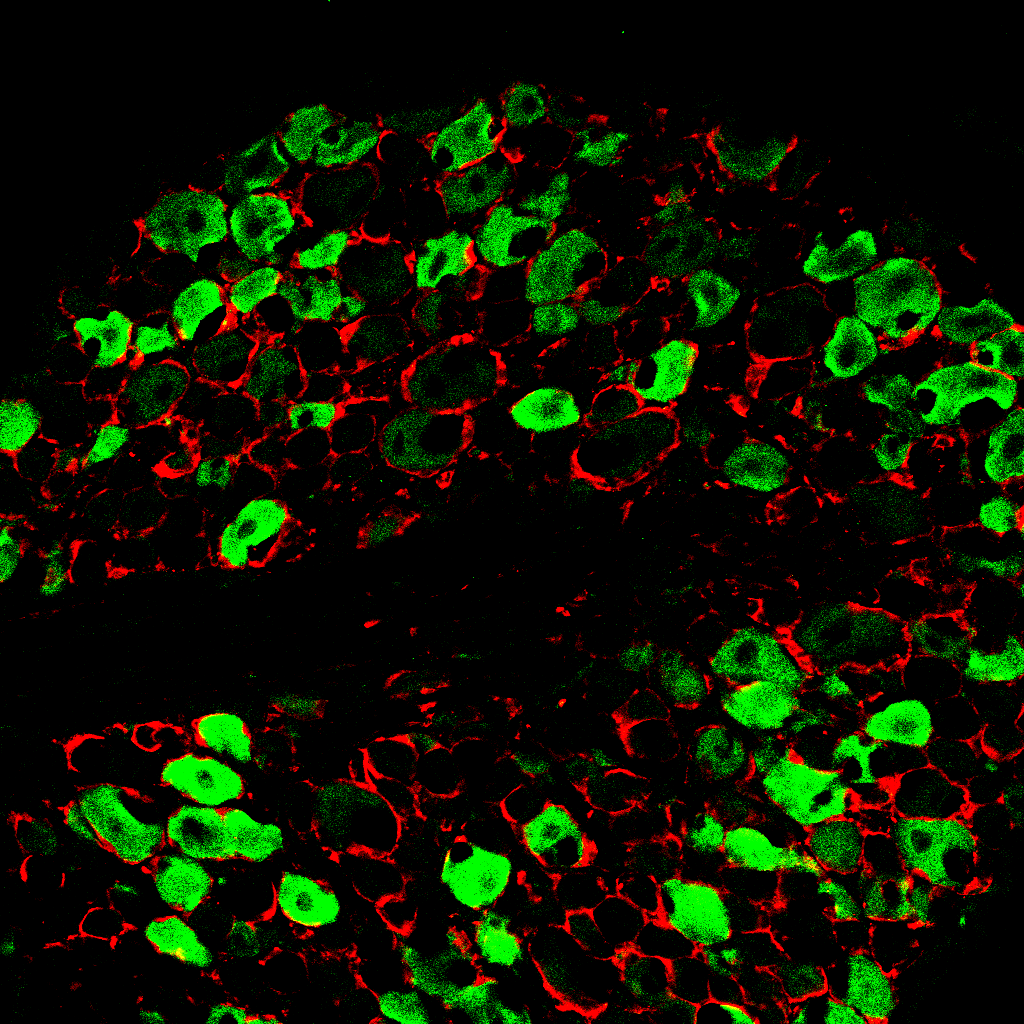

Supplement: Supplementary file 3 — Source data Fig. 1 [file 44318_2025_487_MOESM3_ESM.zip › Figure 1/1C/merge.tif]

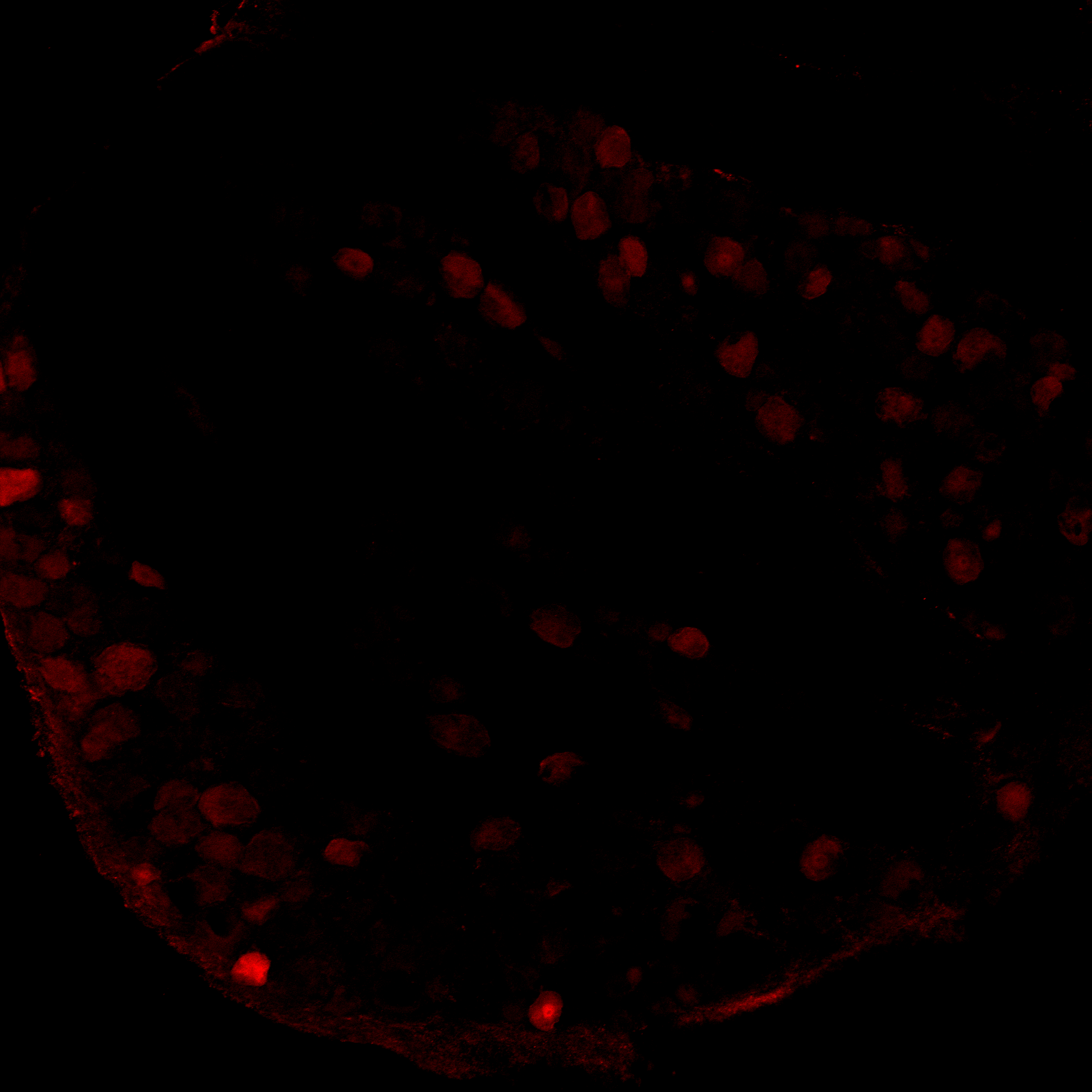

Supplement: Supplementary file 4 — Source data Fig. 2 [file 44318_2025_487_MOESM4_ESM.zip › Figure 2/2B/Copine-6 shRNA/Copine-6.tif]

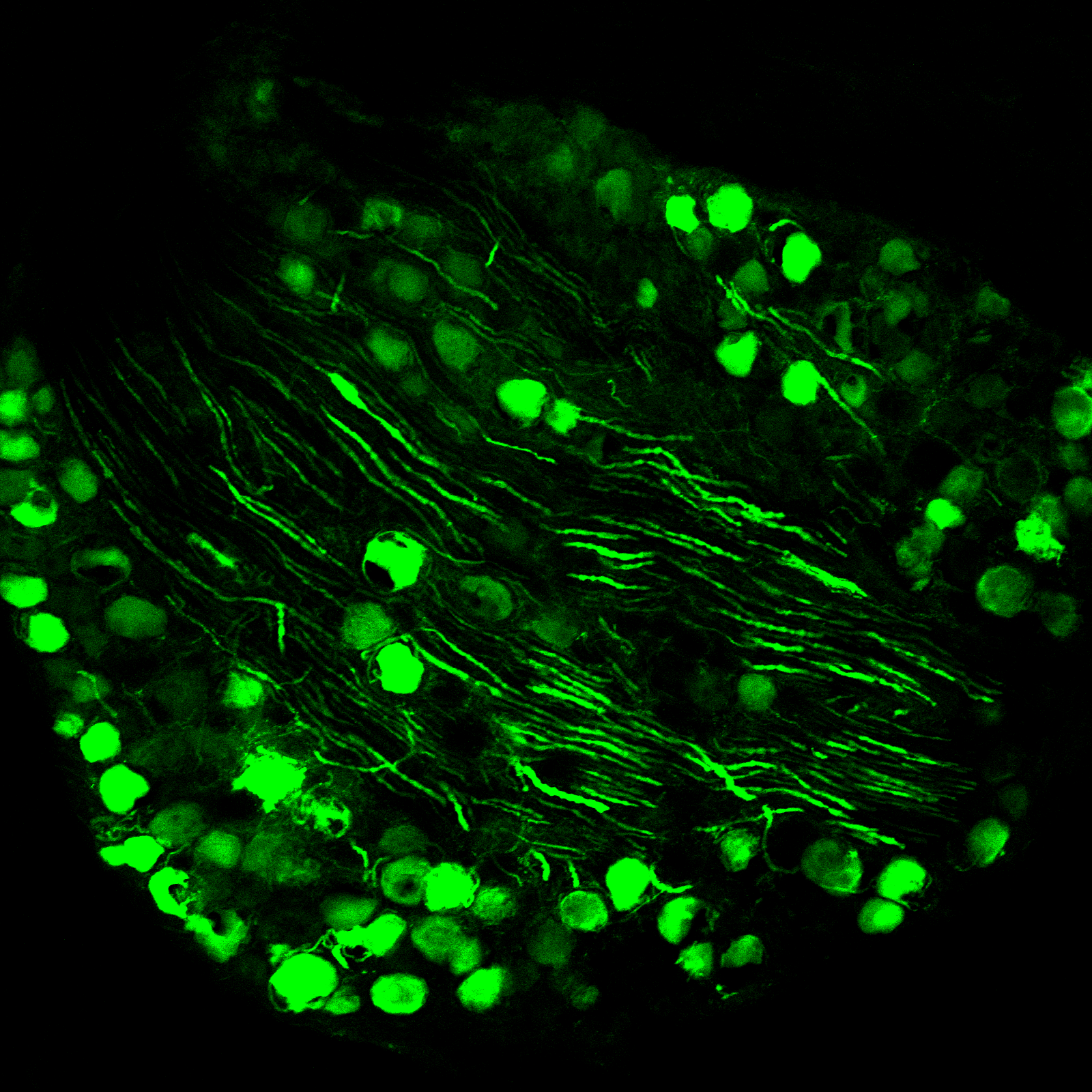

Supplement: Supplementary file 4 — Source data Fig. 2 [file 44318_2025_487_MOESM4_ESM.zip › Figure 2/2B/Copine-6 shRNA/EGFP.tif]

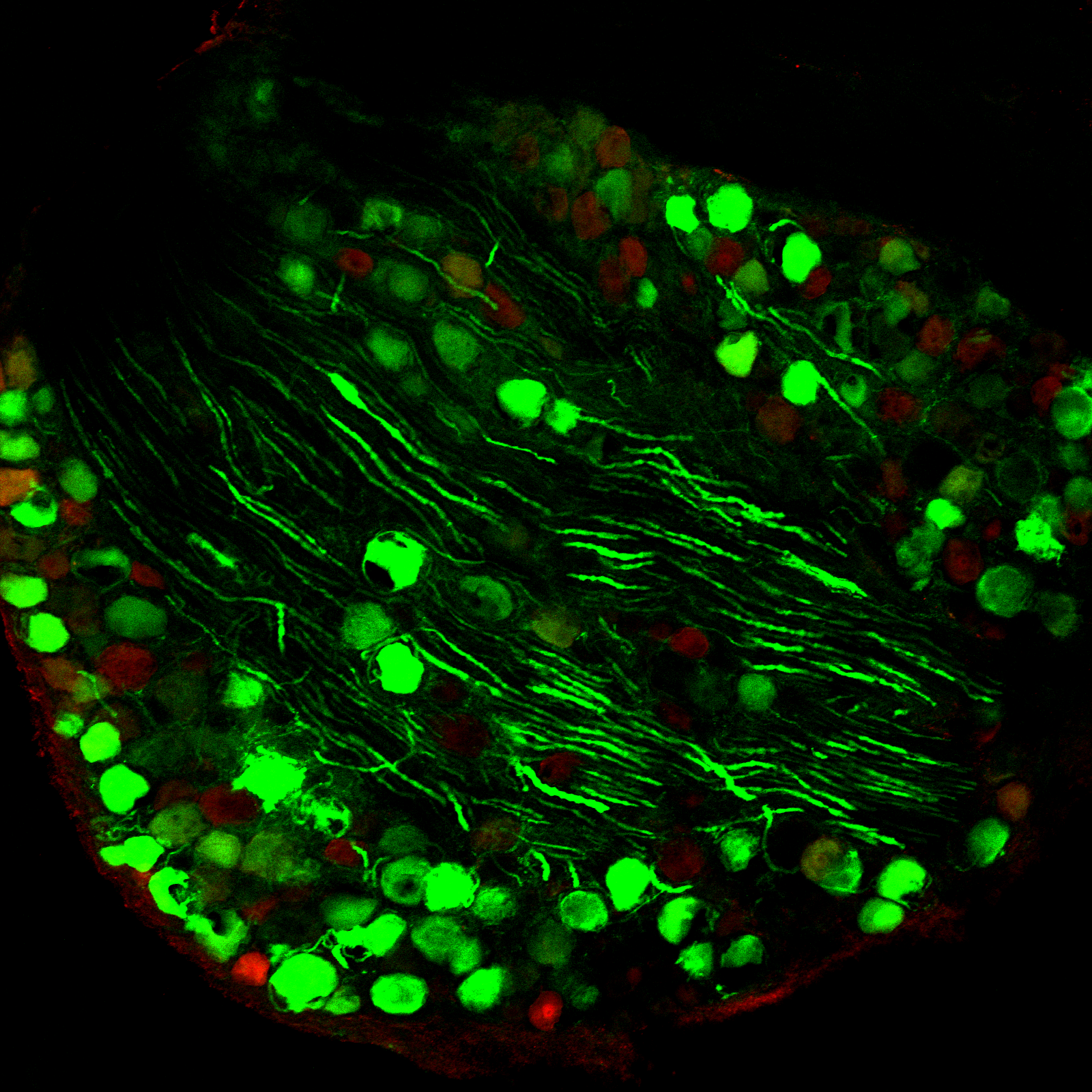

Supplement: Supplementary file 4 — Source data Fig. 2 [file 44318_2025_487_MOESM4_ESM.zip › Figure 2/2B/Copine-6 shRNA/merge.tif]

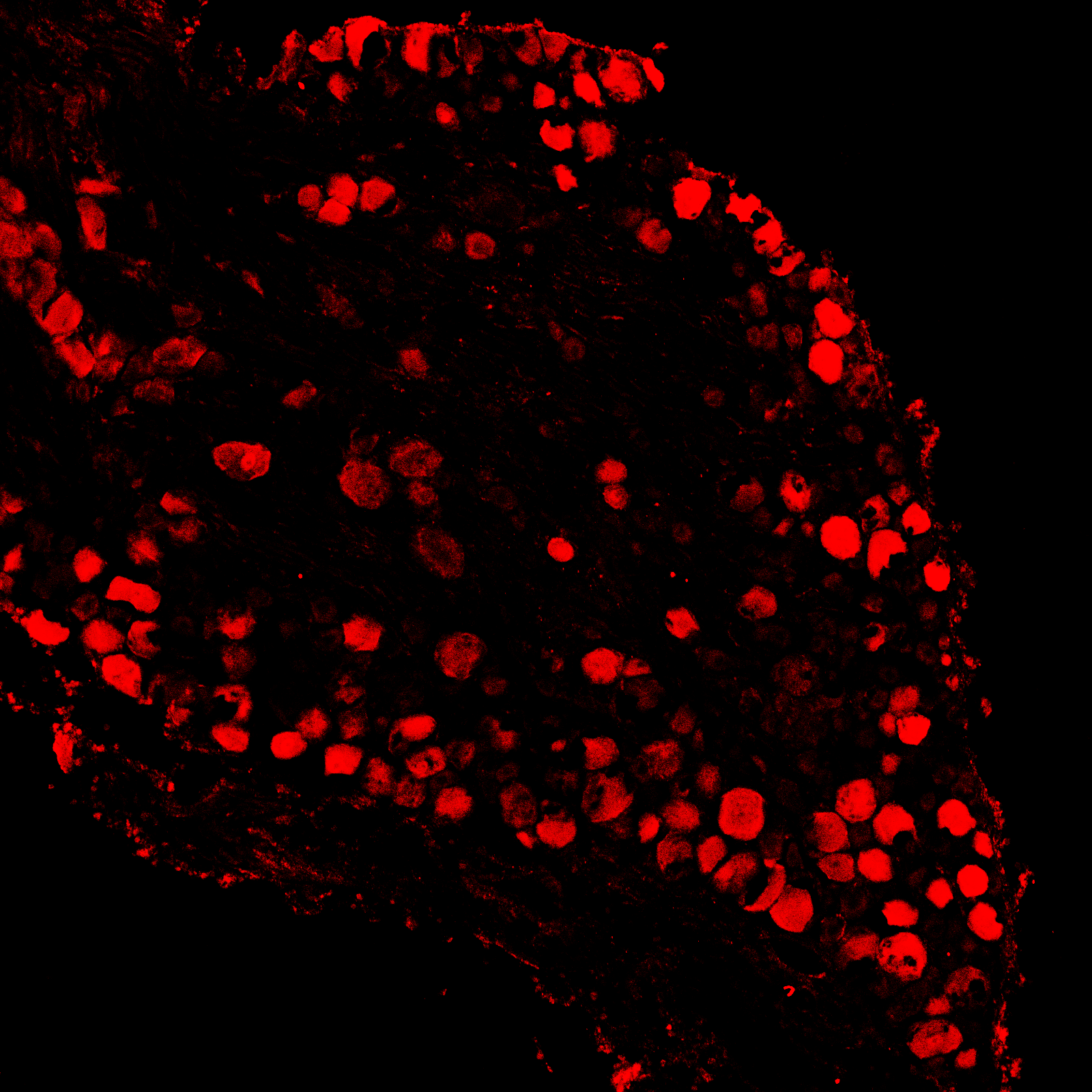

Supplement: Supplementary file 4 — Source data Fig. 2 [file 44318_2025_487_MOESM4_ESM.zip › Figure 2/2B/Scramble shRNA/Copine-6.tif]

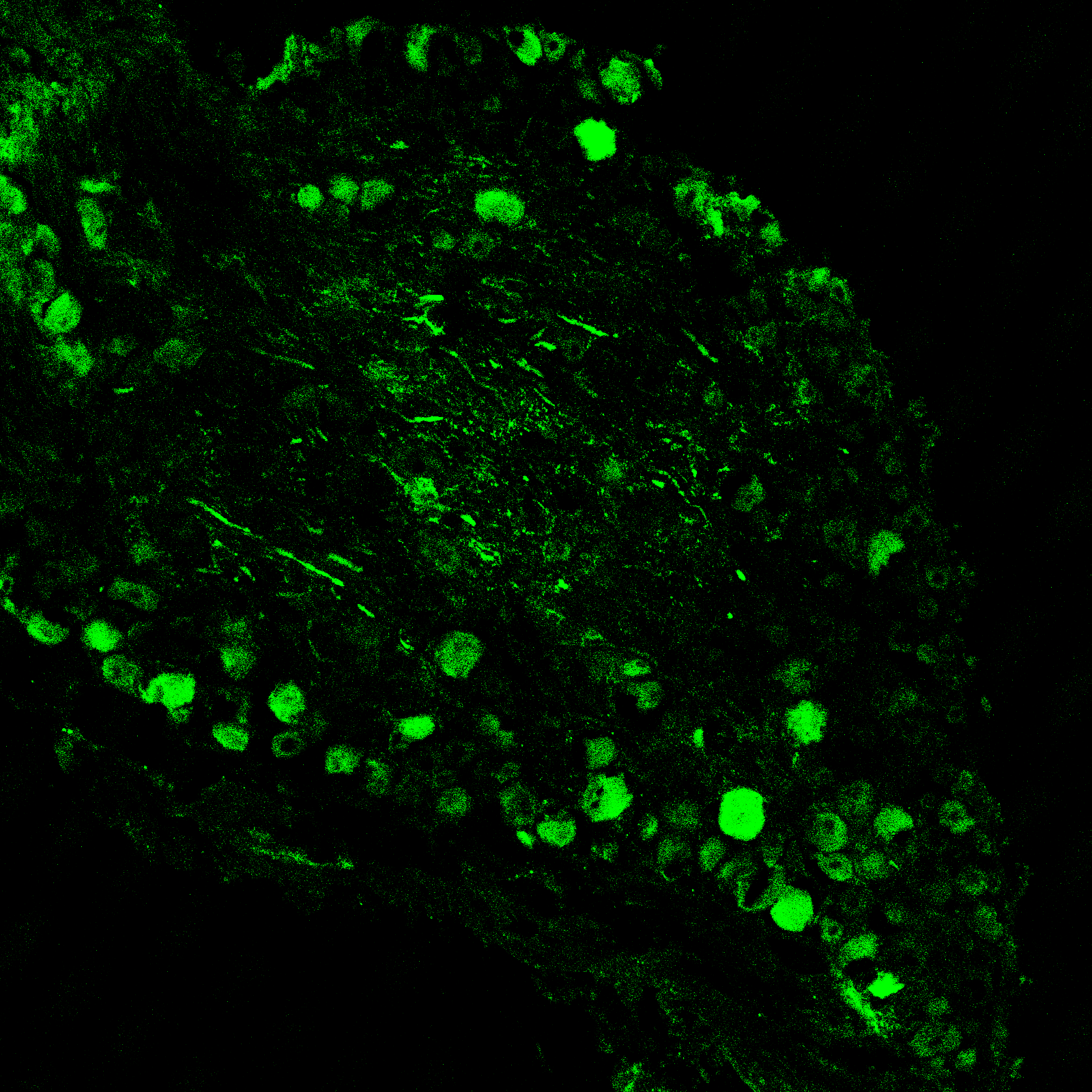

Supplement: Supplementary file 4 — Source data Fig. 2 [file 44318_2025_487_MOESM4_ESM.zip › Figure 2/2B/Scramble shRNA/EGFP.tif]

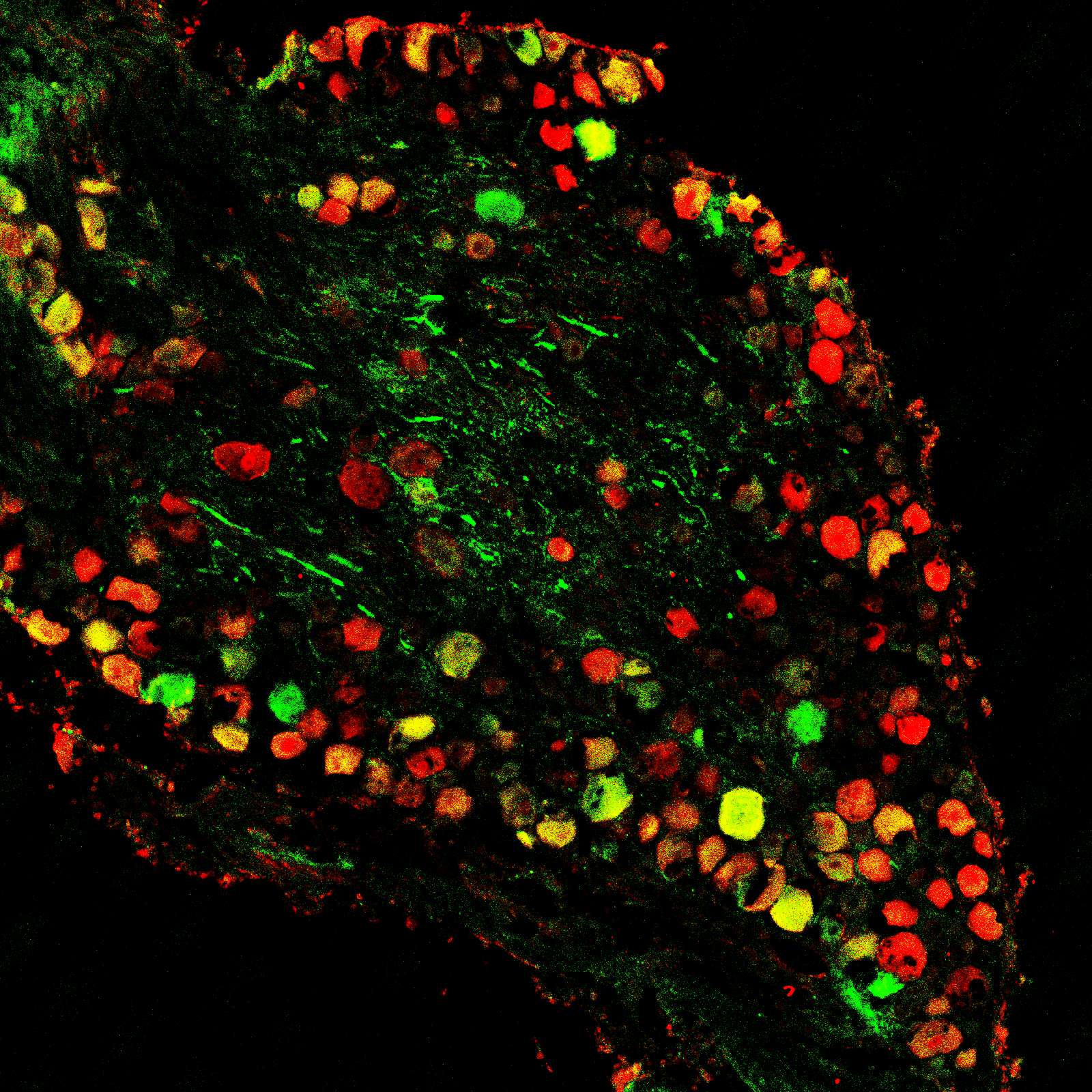

Supplement: Supplementary file 4 — Source data Fig. 2 [file 44318_2025_487_MOESM4_ESM.zip › Figure 2/2B/Scramble shRNA/merge.tif]

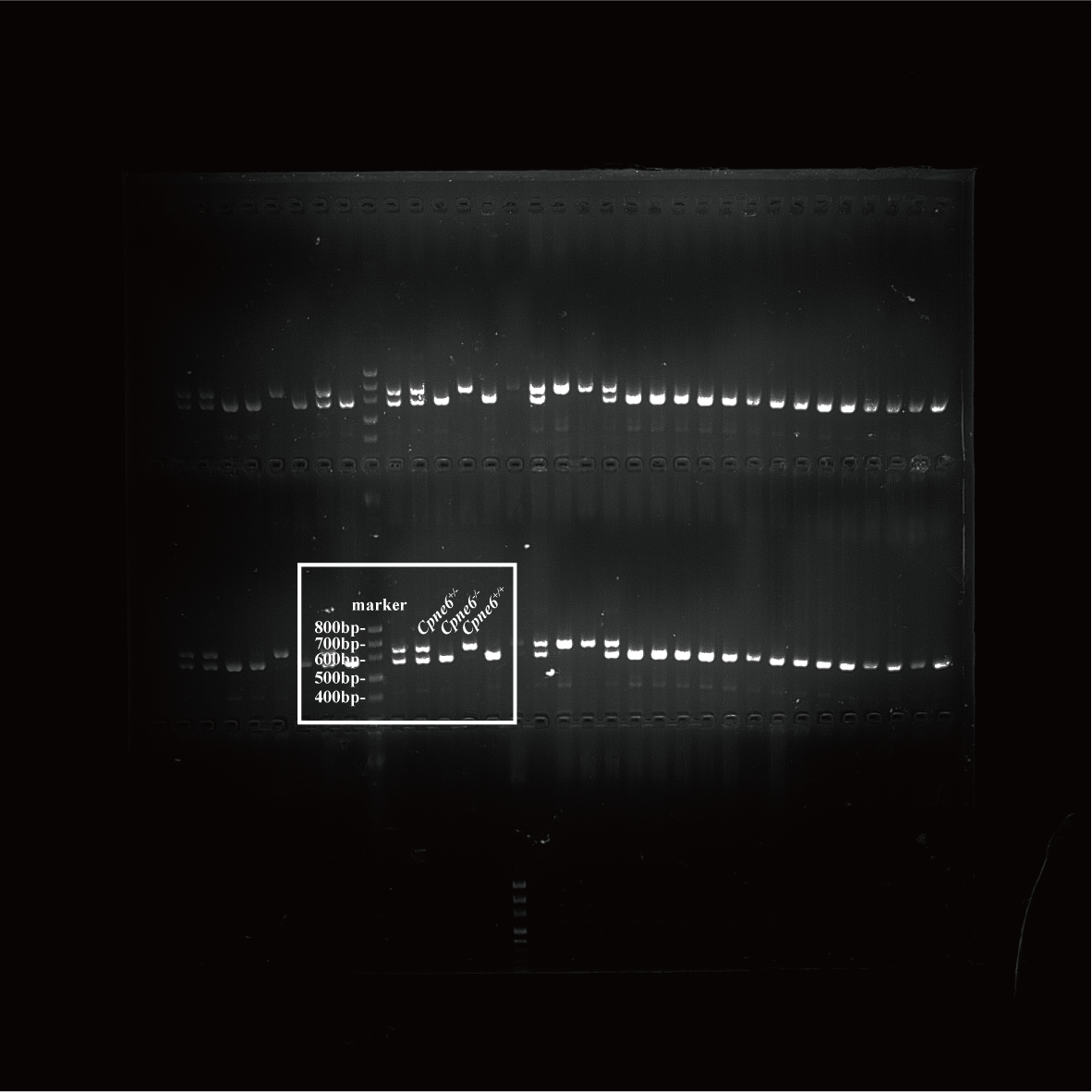

Supplement: Supplementary file 5 — Source data Fig. 3 [file 44318_2025_487_MOESM5_ESM.zip › Figure 3/3B.tif]

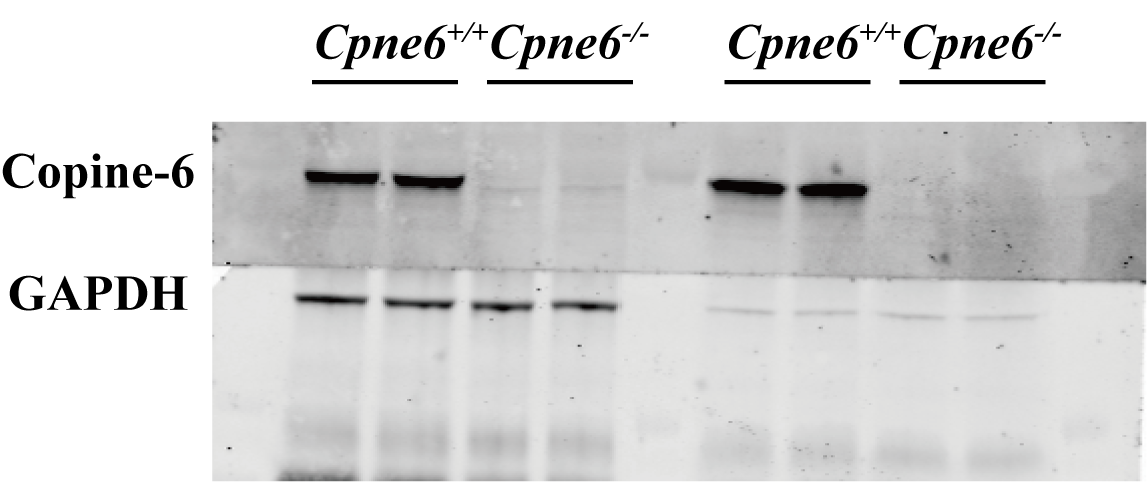

Supplement: Supplementary file 5 — Source data Fig. 3 [file 44318_2025_487_MOESM5_ESM.zip › Figure 3/3C.tif]

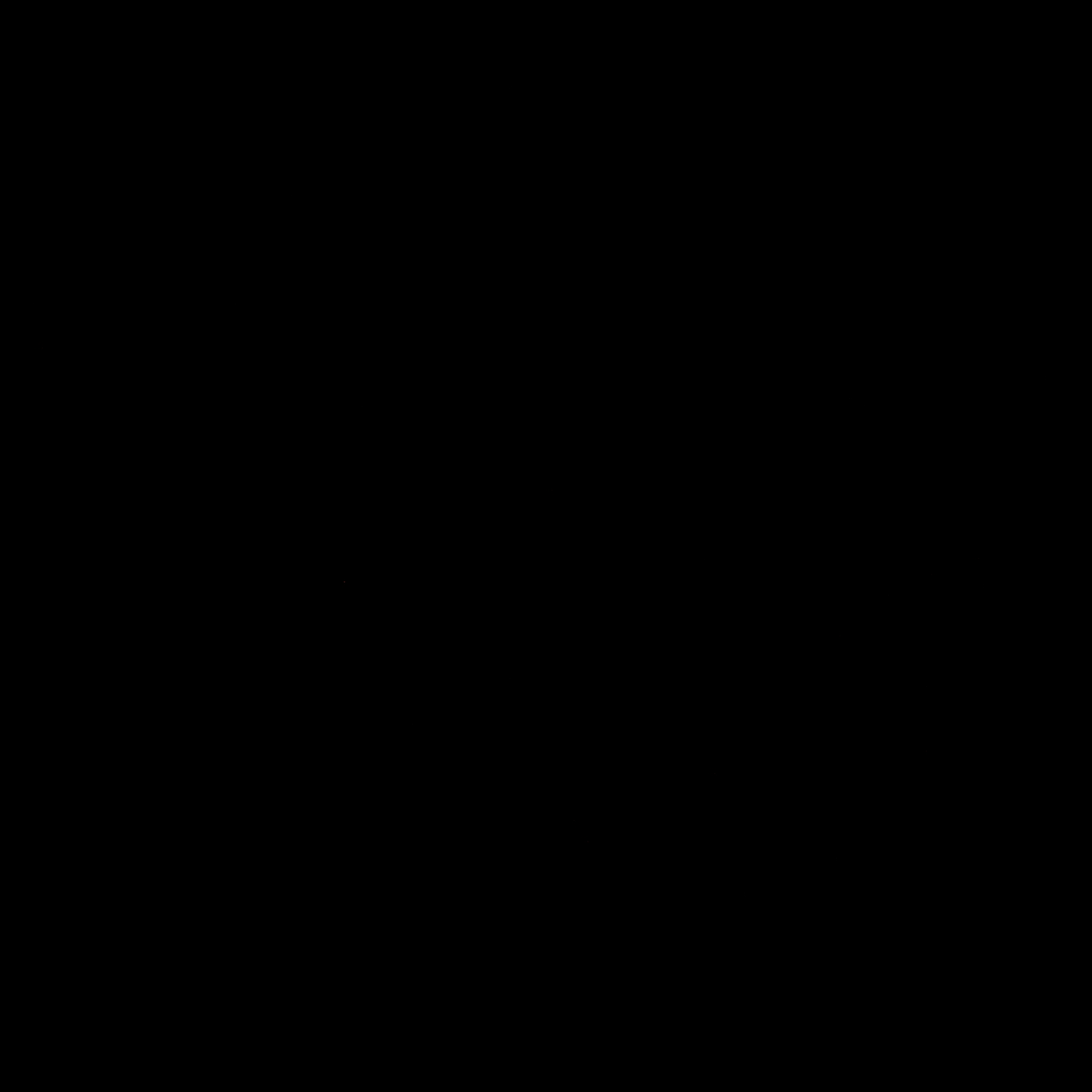

Supplement: Supplementary file 5 — Source data Fig. 3 [file 44318_2025_487_MOESM5_ESM.zip › Figure 3/3E/control-Copine-6.jpg]

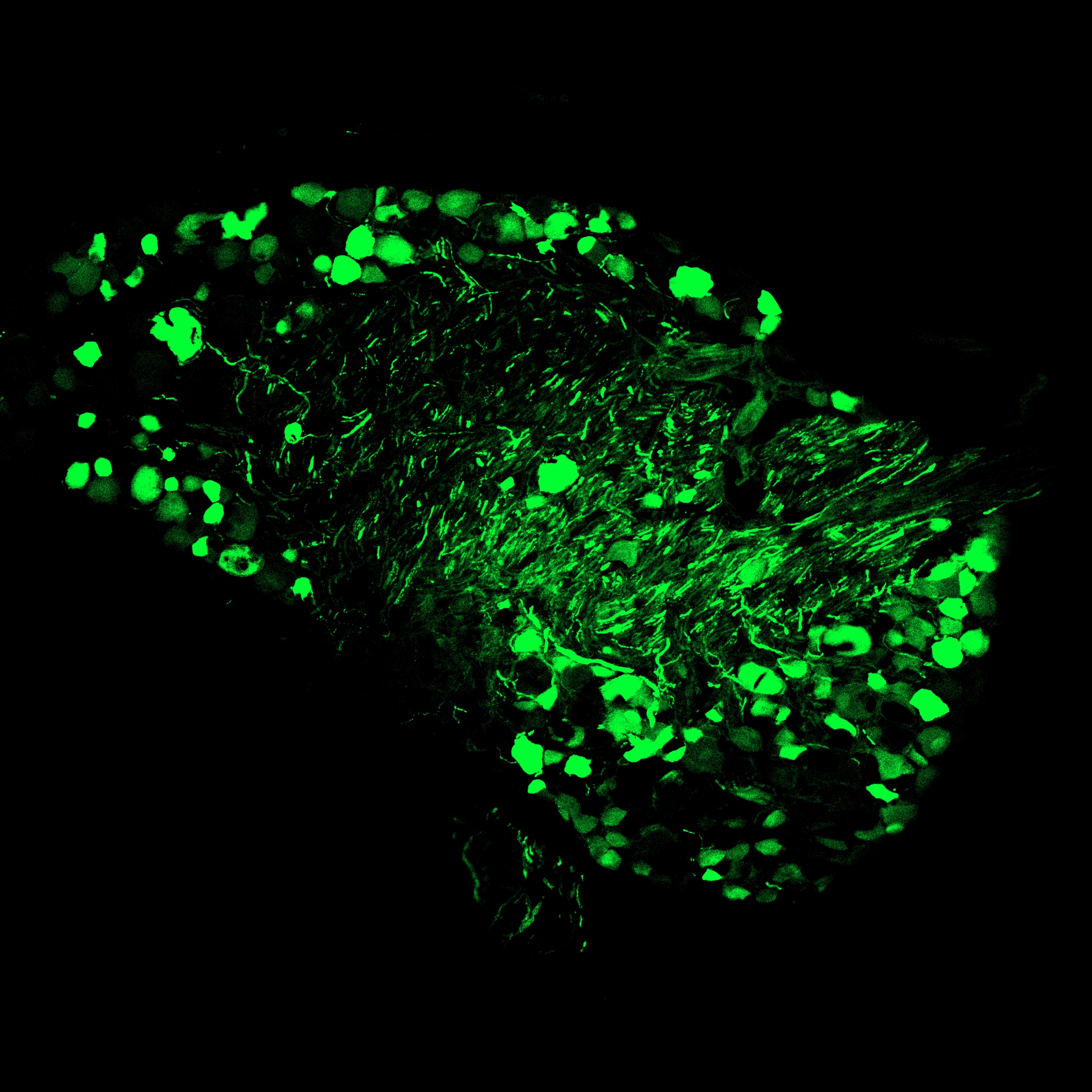

Supplement: Supplementary file 5 — Source data Fig. 3 [file 44318_2025_487_MOESM5_ESM.zip › Figure 3/3E/control-EGFP.jpg]

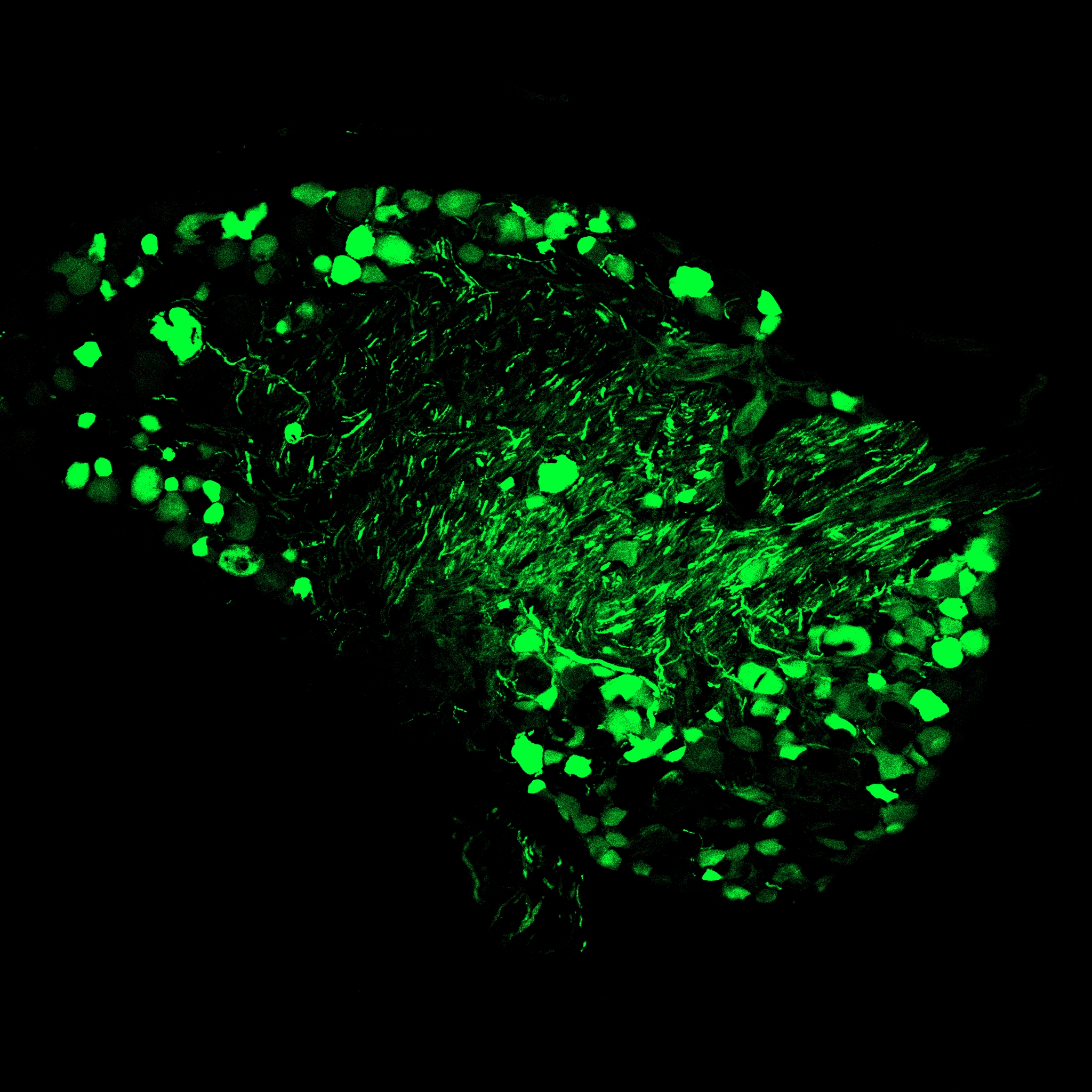

Supplement: Supplementary file 5 — Source data Fig. 3 [file 44318_2025_487_MOESM5_ESM.zip › Figure 3/3E/control-merge.jpg]

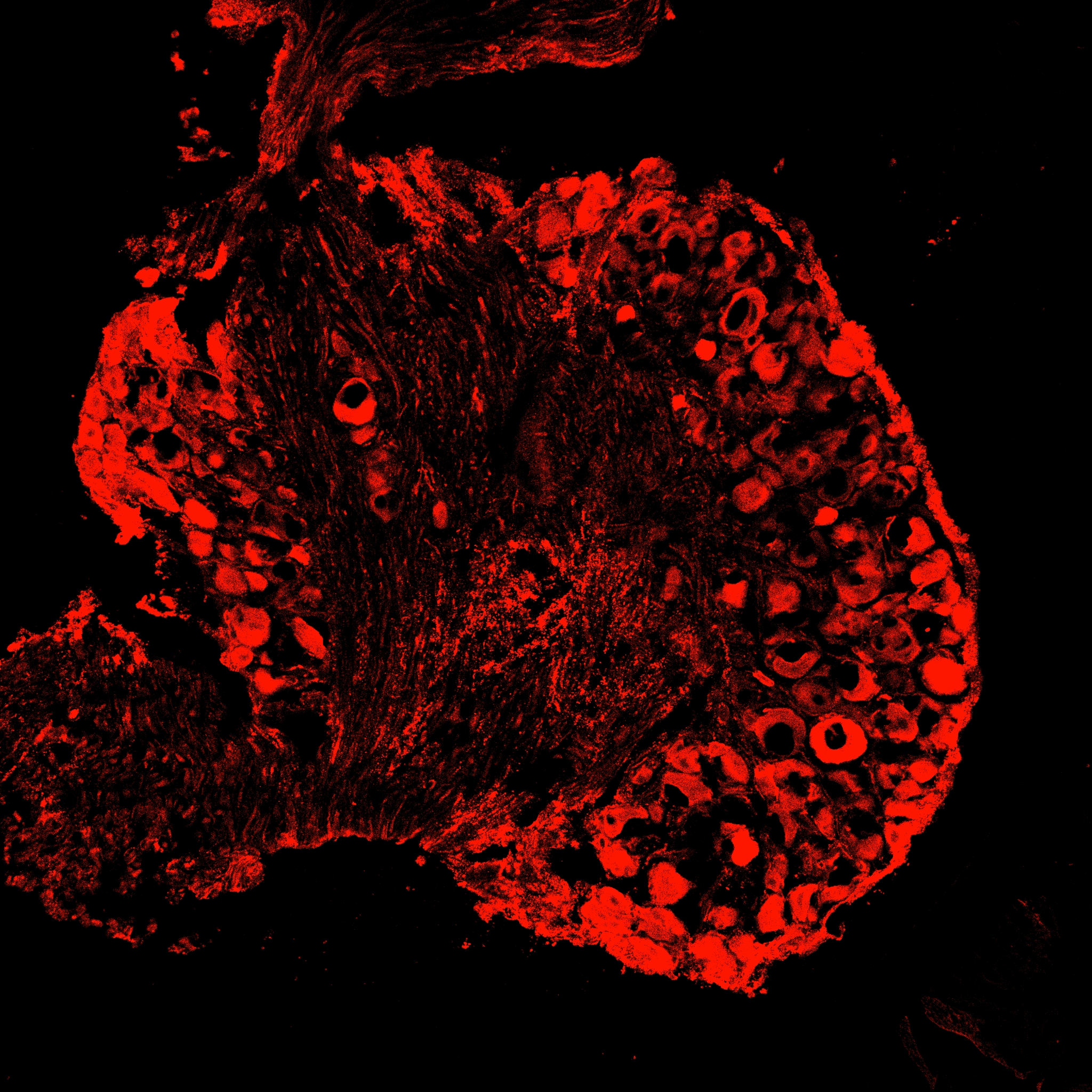

Supplement: Supplementary file 5 — Source data Fig. 3 [file 44318_2025_487_MOESM5_ESM.zip › Figure 3/3E/over-Copine-6.jpg]

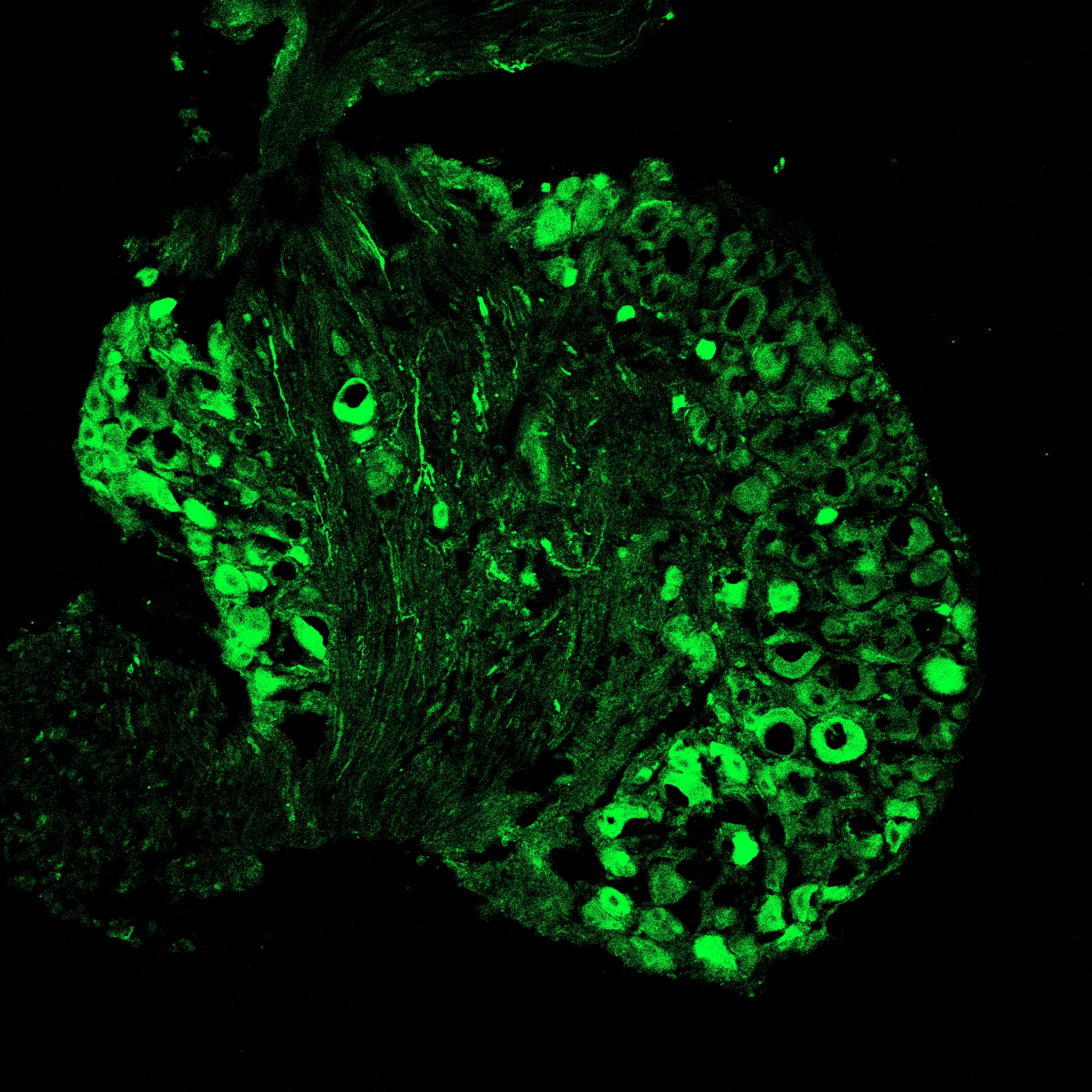

Supplement: Supplementary file 5 — Source data Fig. 3 [file 44318_2025_487_MOESM5_ESM.zip › Figure 3/3E/over-EGFP.jpg]

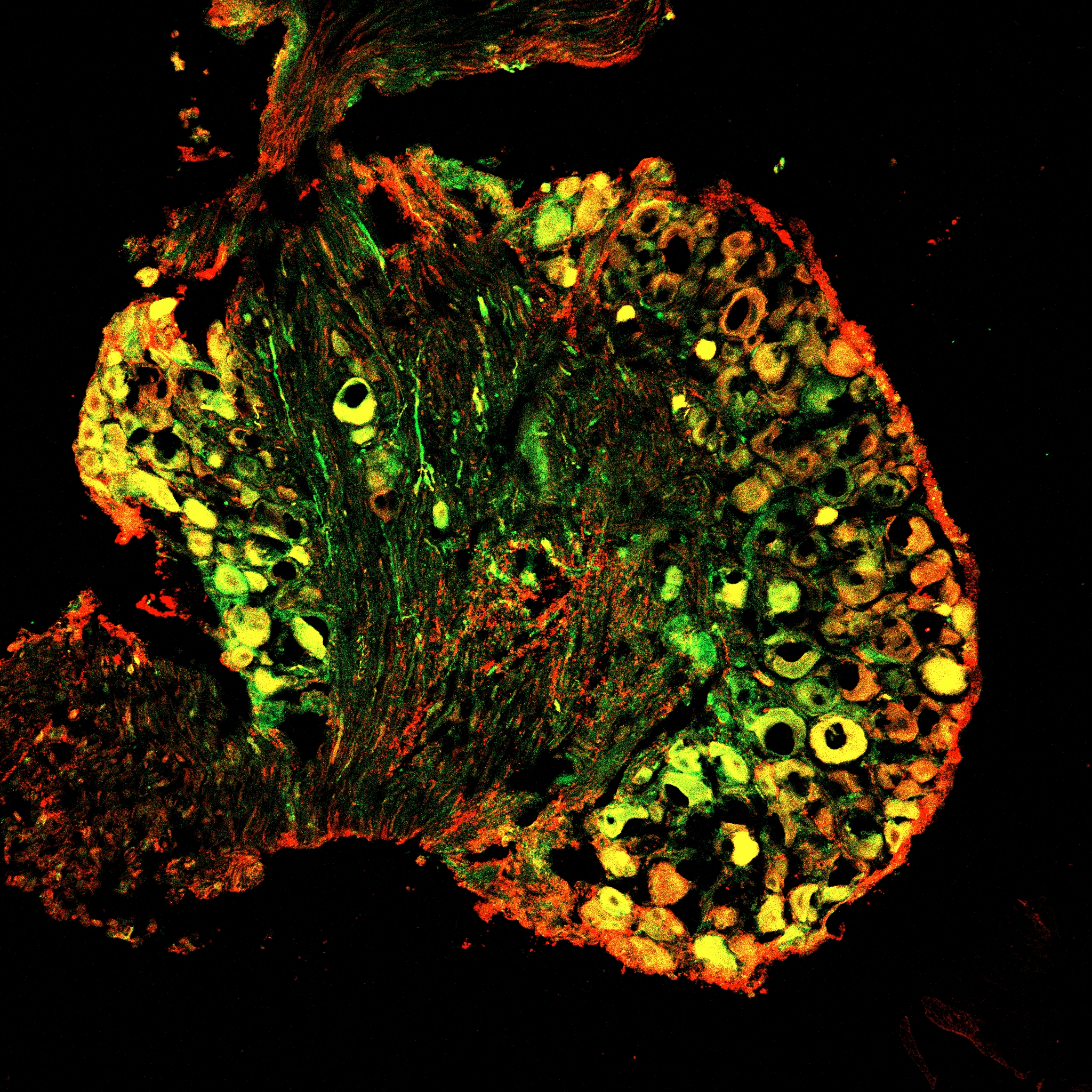

Supplement: Supplementary file 5 — Source data Fig. 3 [file 44318_2025_487_MOESM5_ESM.zip › Figure 3/3E/over-merge.jpg]

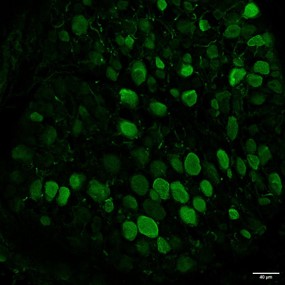

Supplement: Supplementary file 6 — Source data Fig. 4 [file 44318_2025_487_MOESM6_ESM.zip › Figure 4/4C/Copine-6.jpg]

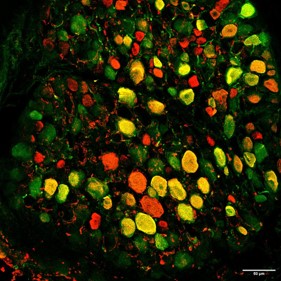

Supplement: Supplementary file 6 — Source data Fig. 4 [file 44318_2025_487_MOESM6_ESM.zip › Figure 4/4C/merge.jpg]

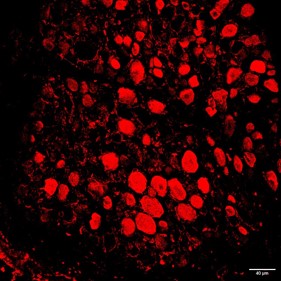

Supplement: Supplementary file 6 — Source data Fig. 4 [file 44318_2025_487_MOESM6_ESM.zip › Figure 4/4C/TRPM3.jpg]

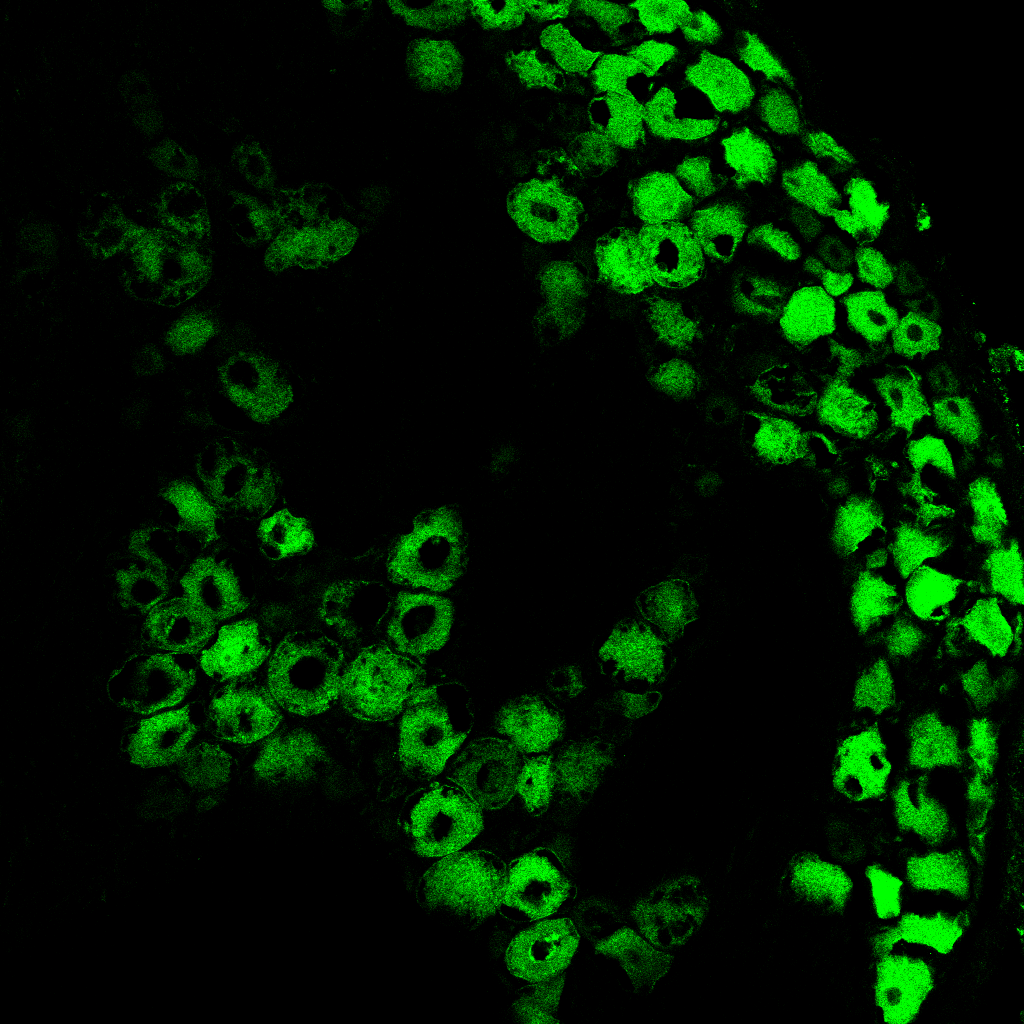

Supplement: Supplementary file 6 — Source data Fig. 4 [file 44318_2025_487_MOESM6_ESM.zip › Figure 4/4D/Copine-6.tif]

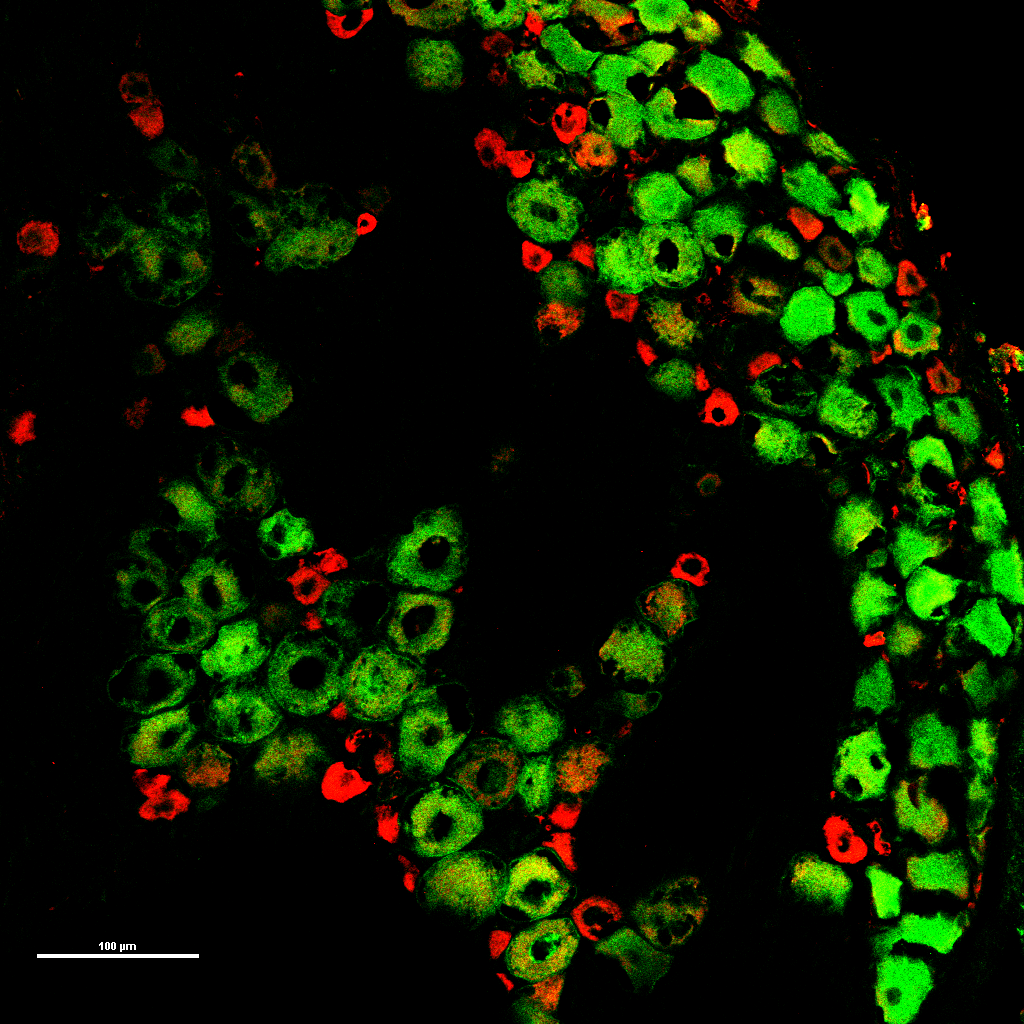

Supplement: Supplementary file 6 — Source data Fig. 4 [file 44318_2025_487_MOESM6_ESM.zip › Figure 4/4D/merge.tif]

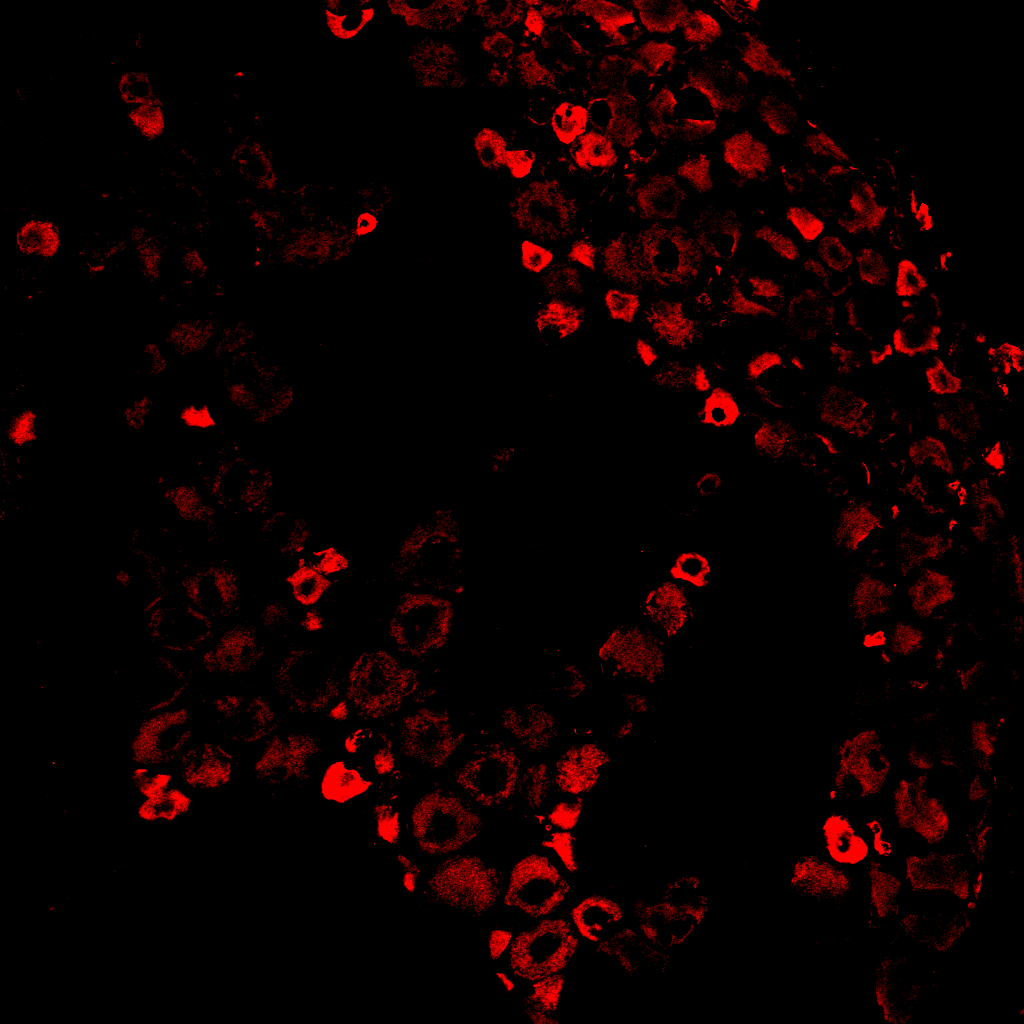

Supplement: Supplementary file 6 — Source data Fig. 4 [file 44318_2025_487_MOESM6_ESM.zip › Figure 4/4D/TRPV1.tif]

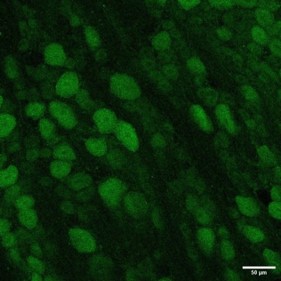

Supplement: Supplementary file 6 — Source data Fig. 4 [file 44318_2025_487_MOESM6_ESM.zip › Figure 4/4E/CPNE6.jpg]

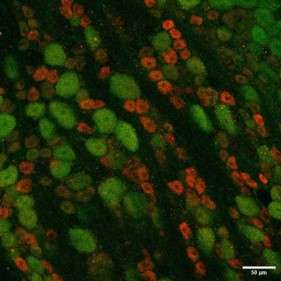

Supplement: Supplementary file 6 — Source data Fig. 4 [file 44318_2025_487_MOESM6_ESM.zip › Figure 4/4E/MERGE.jpg]

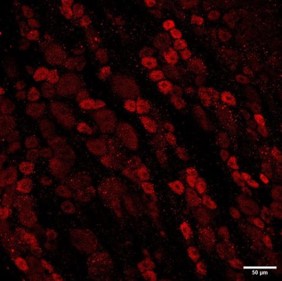

Supplement: Supplementary file 6 — Source data Fig. 4 [file 44318_2025_487_MOESM6_ESM.zip › Figure 4/4E/TRPA1.jpg]

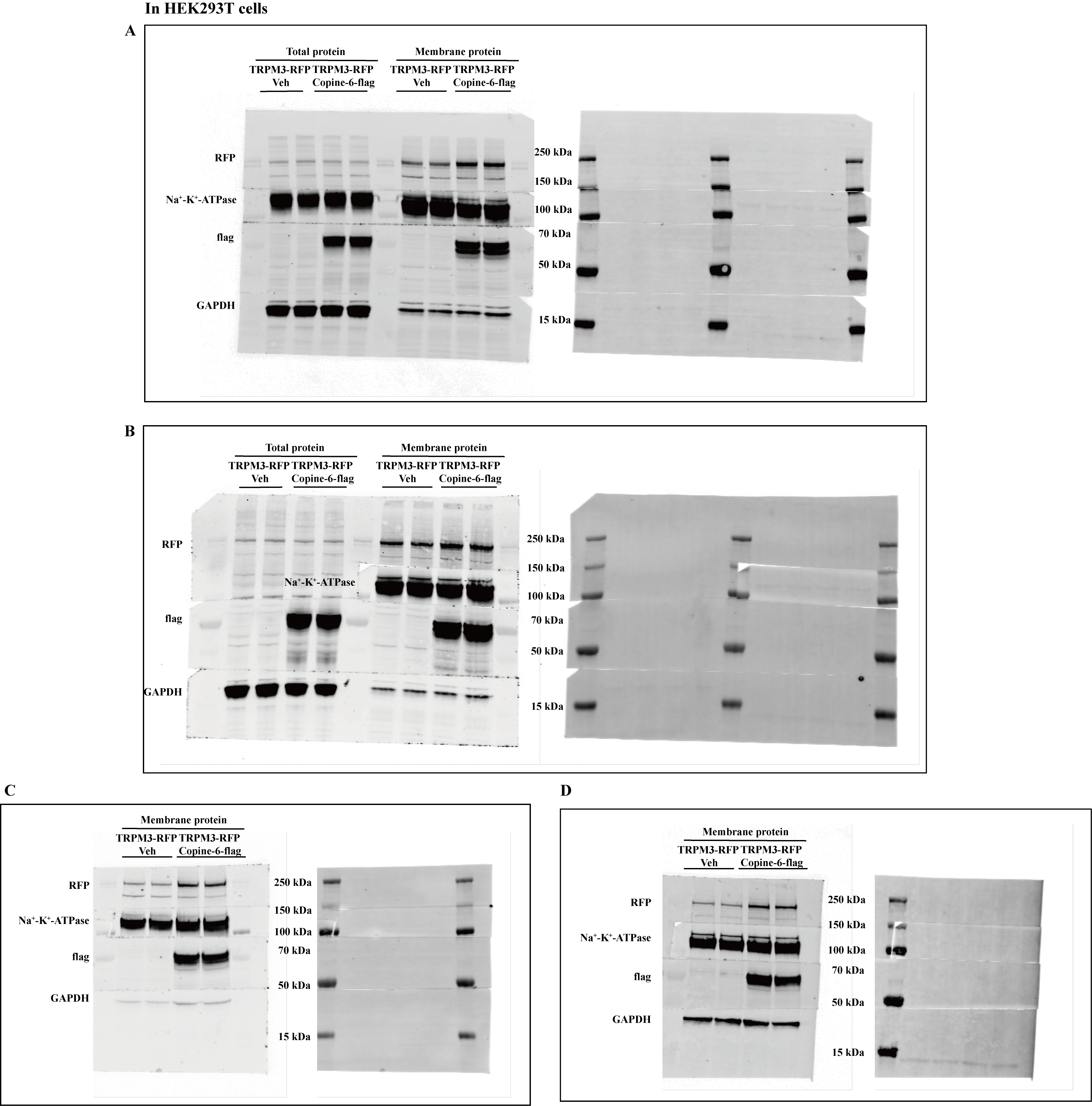

Supplement: Supplementary file 9 — Source data Fig. 7 [file 44318_2025_487_MOESM9_ESM.zip › Figure 7/7AB.tif]

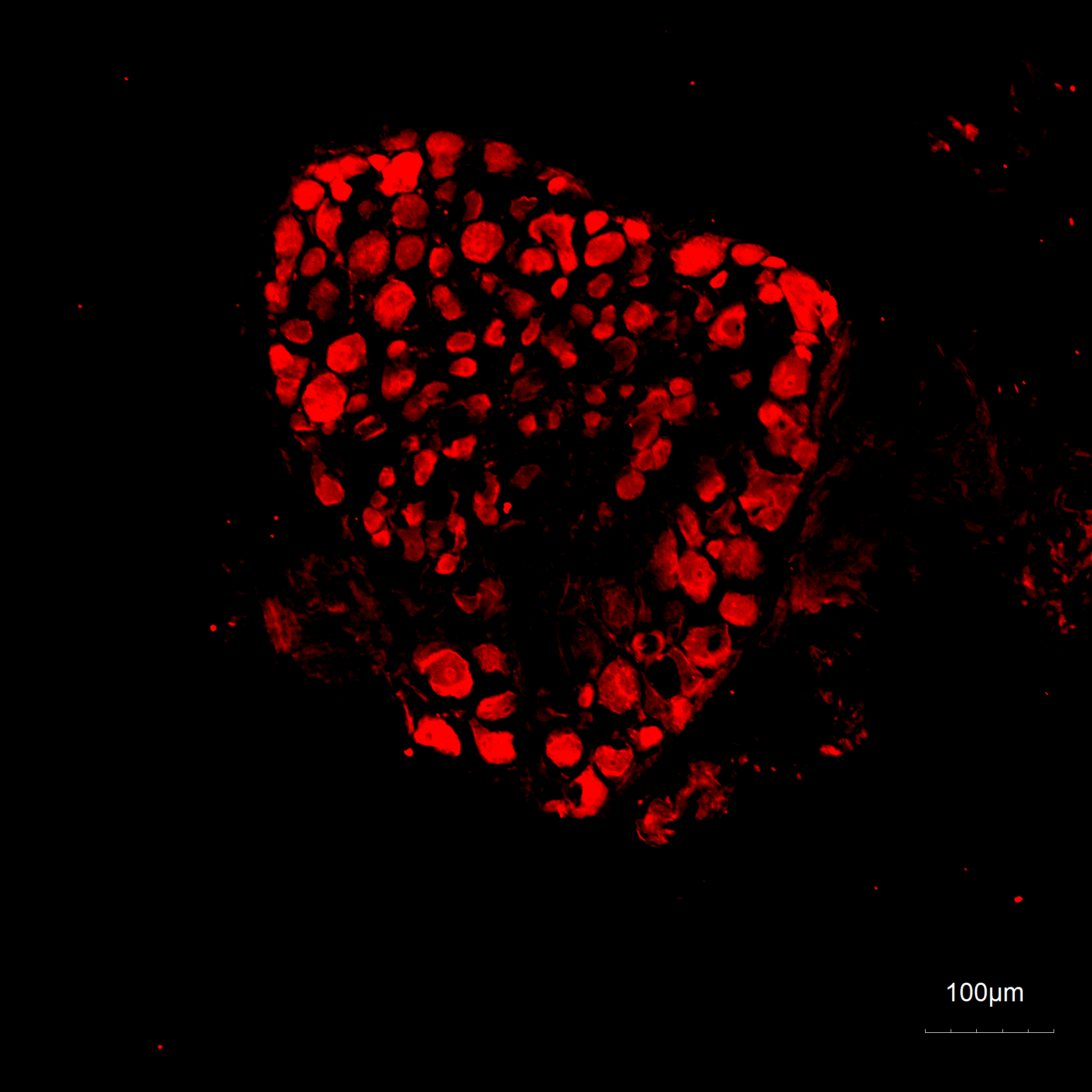

Supplement: Supplementary file 9 — Source data Fig. 7 [file 44318_2025_487_MOESM9_ESM.zip › Figure 7/7E/TRPM3-KO.tif]

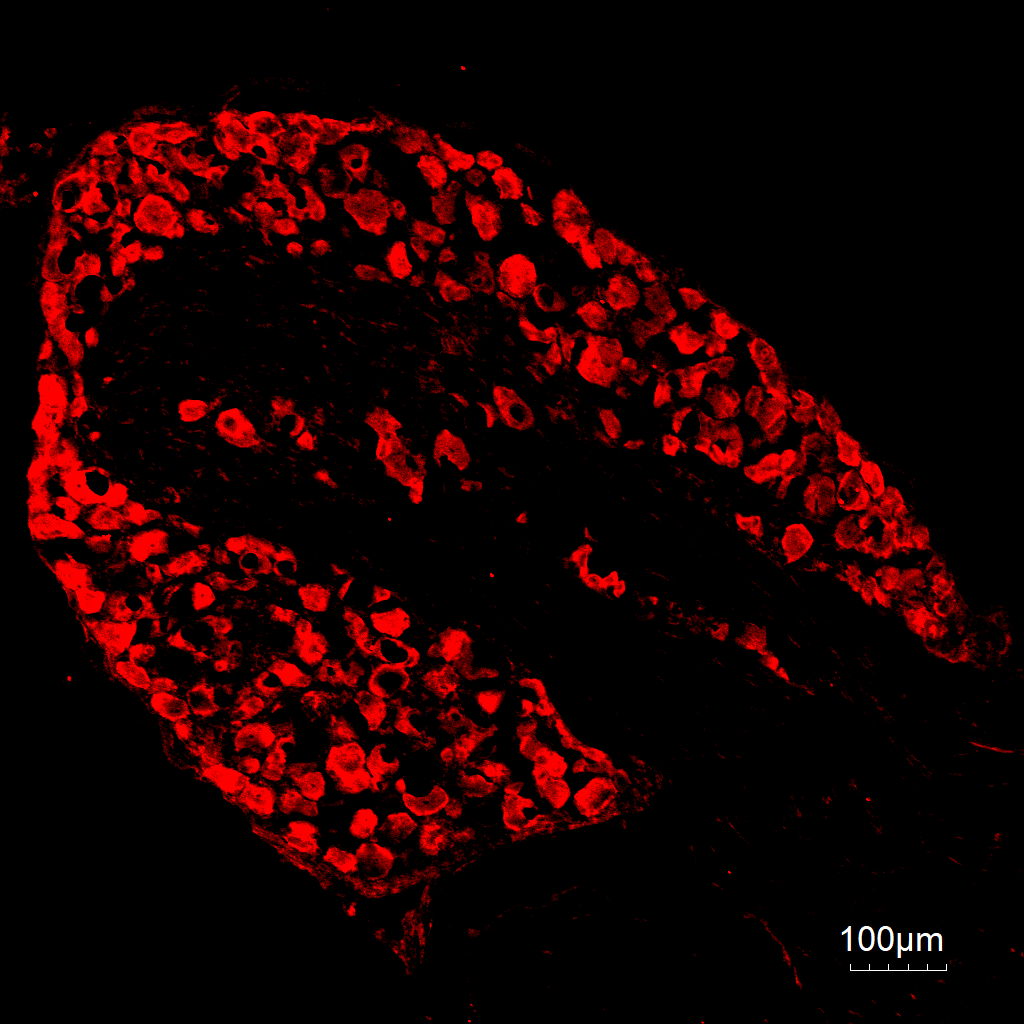

Supplement: Supplementary file 9 — Source data Fig. 7 [file 44318_2025_487_MOESM9_ESM.zip › Figure 7/7E/TRPM3-WT.tif]

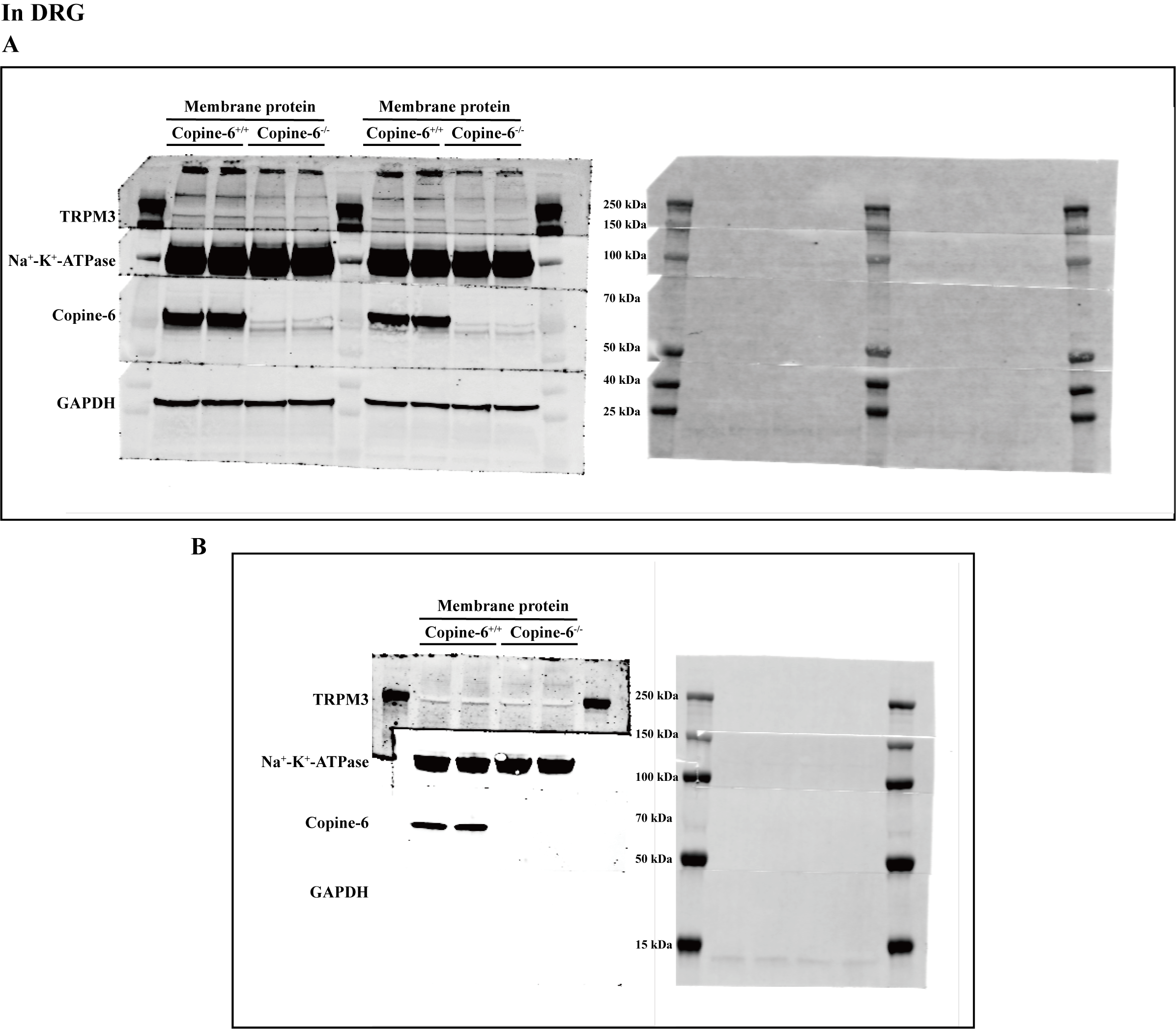

Supplement: Supplementary file 9 — Source data Fig. 7 [file 44318_2025_487_MOESM9_ESM.zip › Figure 7/7F.tif]

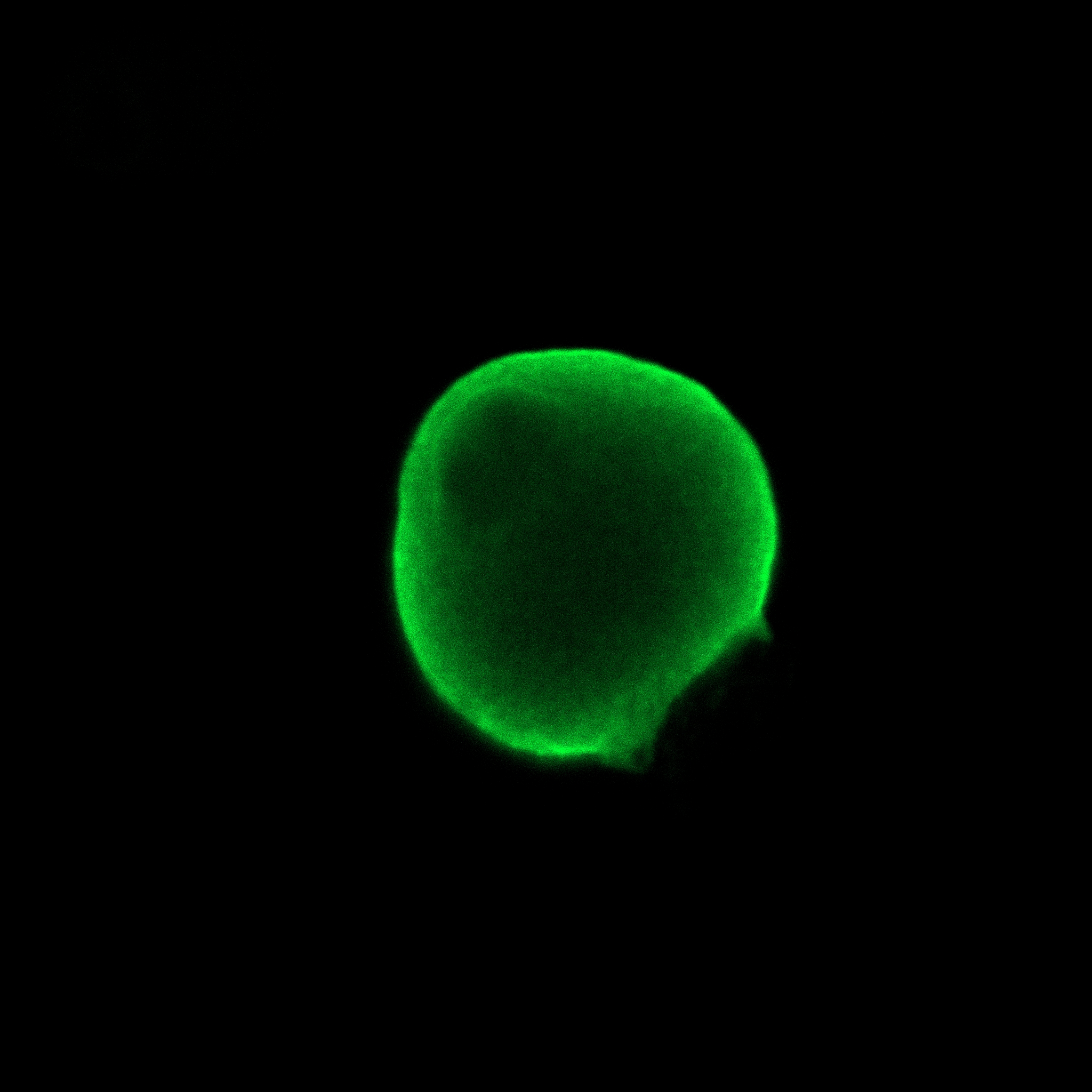

Supplement: Supplementary file 10 — Source data Fig. 8 [file 44318_2025_487_MOESM10_ESM.zip › Figure 8/8A 45/Copine-6.jpg]

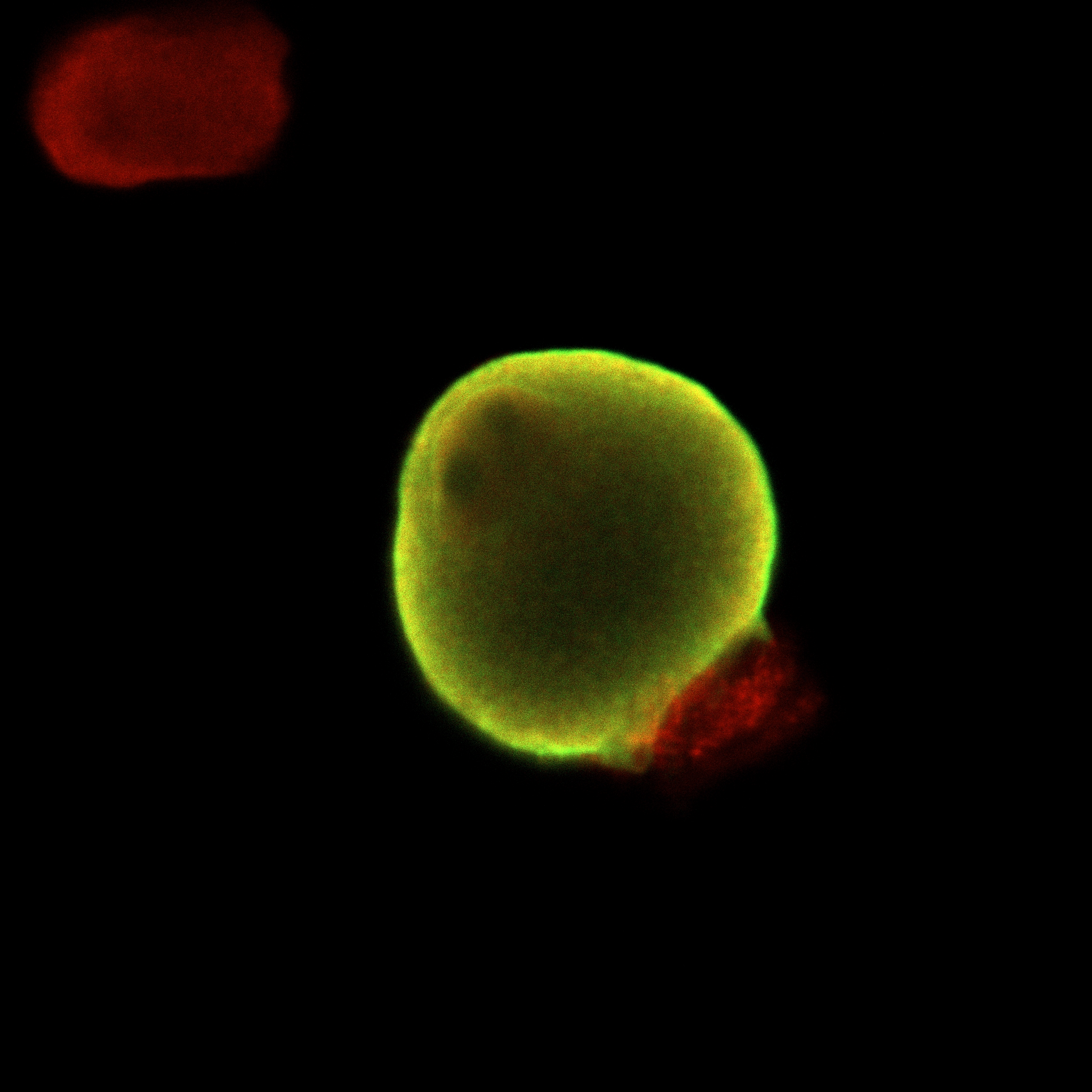

Supplement: Supplementary file 10 — Source data Fig. 8 [file 44318_2025_487_MOESM10_ESM.zip › Figure 8/8A 45/merge.jpg]

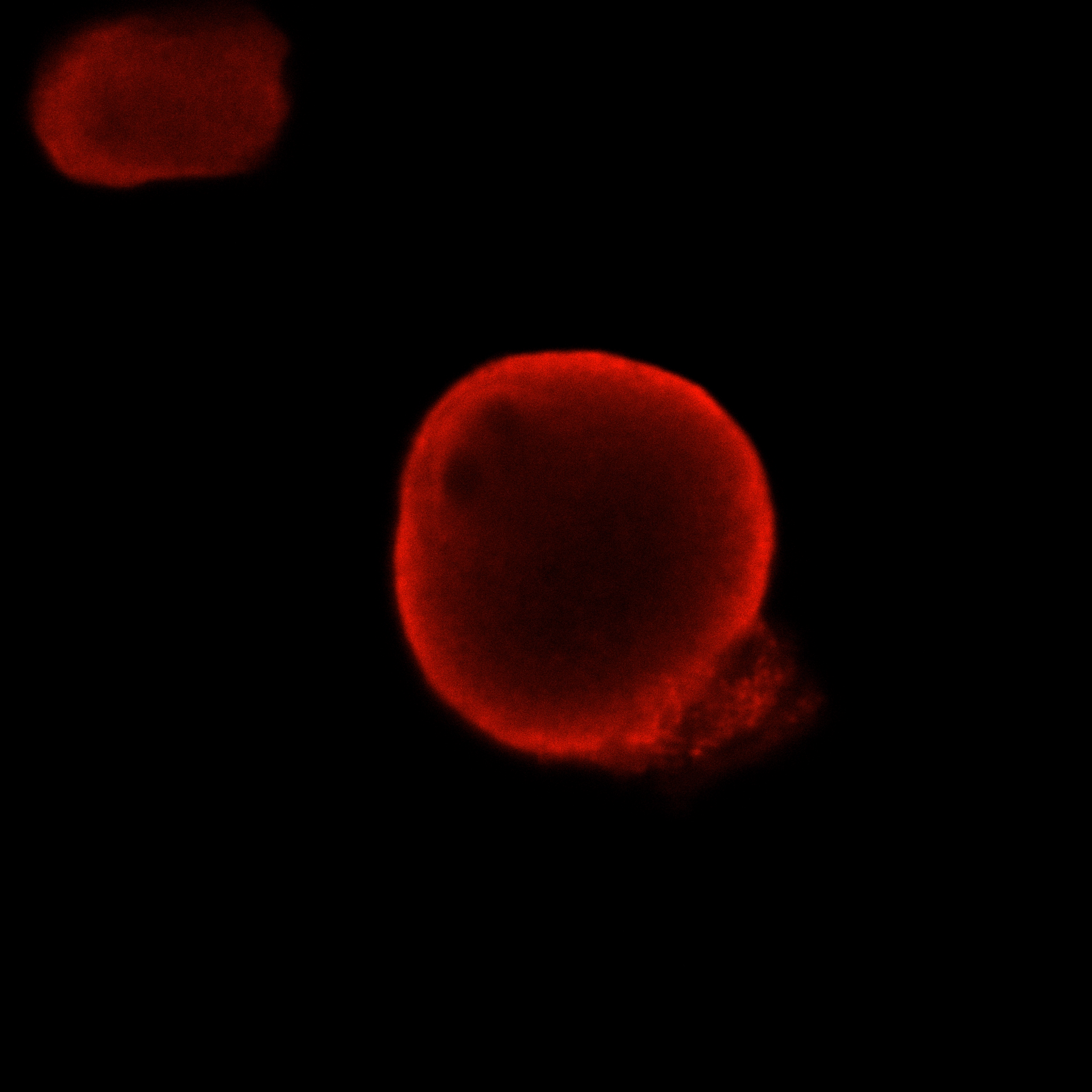

Supplement: Supplementary file 10 — Source data Fig. 8 [file 44318_2025_487_MOESM10_ESM.zip › Figure 8/8A 45/TRPM3.jpg]

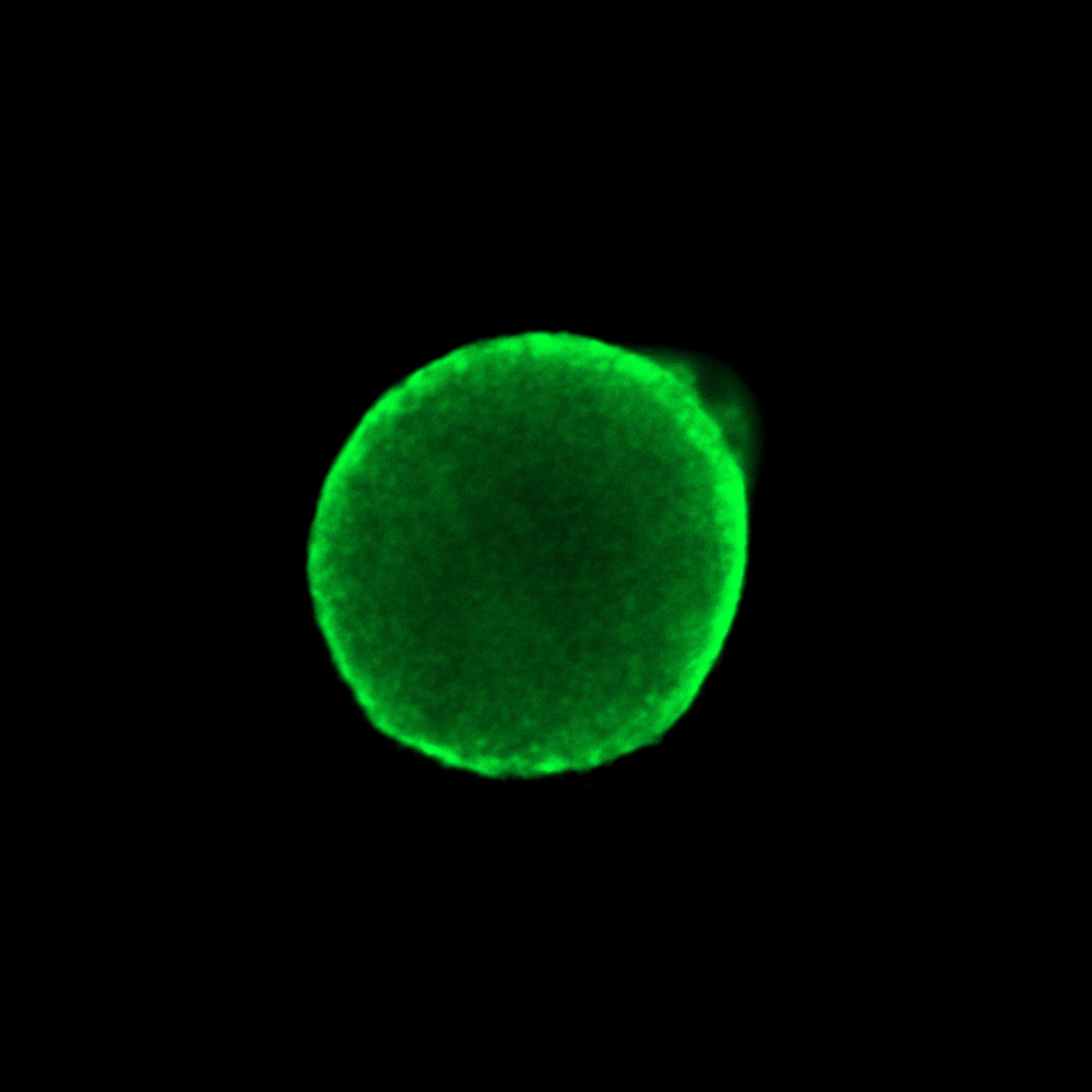

Supplement: Supplementary file 10 — Source data Fig. 8 [file 44318_2025_487_MOESM10_ESM.zip › Figure 8/8A CIM0216/Copine-6.jpg]

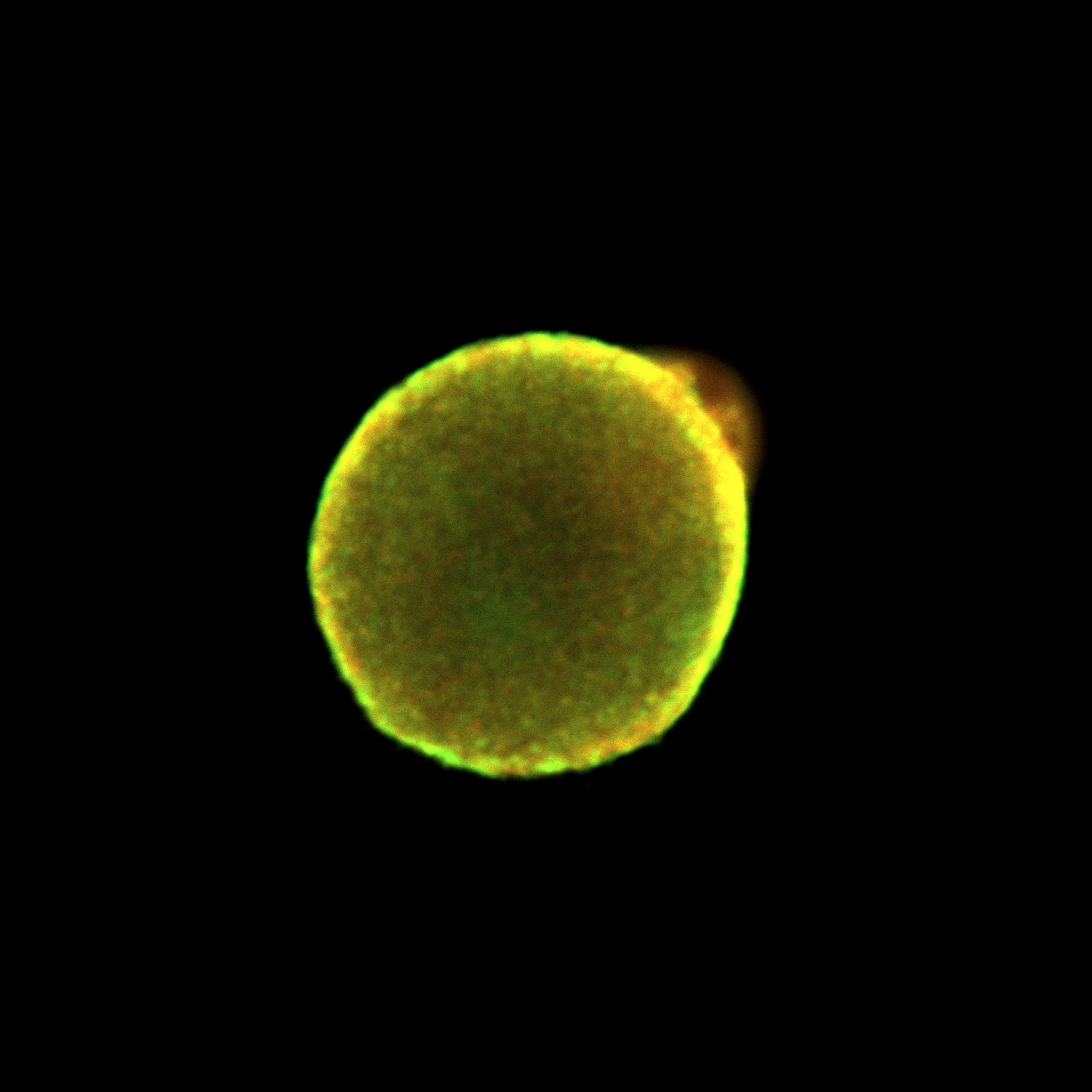

Supplement: Supplementary file 10 — Source data Fig. 8 [file 44318_2025_487_MOESM10_ESM.zip › Figure 8/8A CIM0216/merge.jpg]

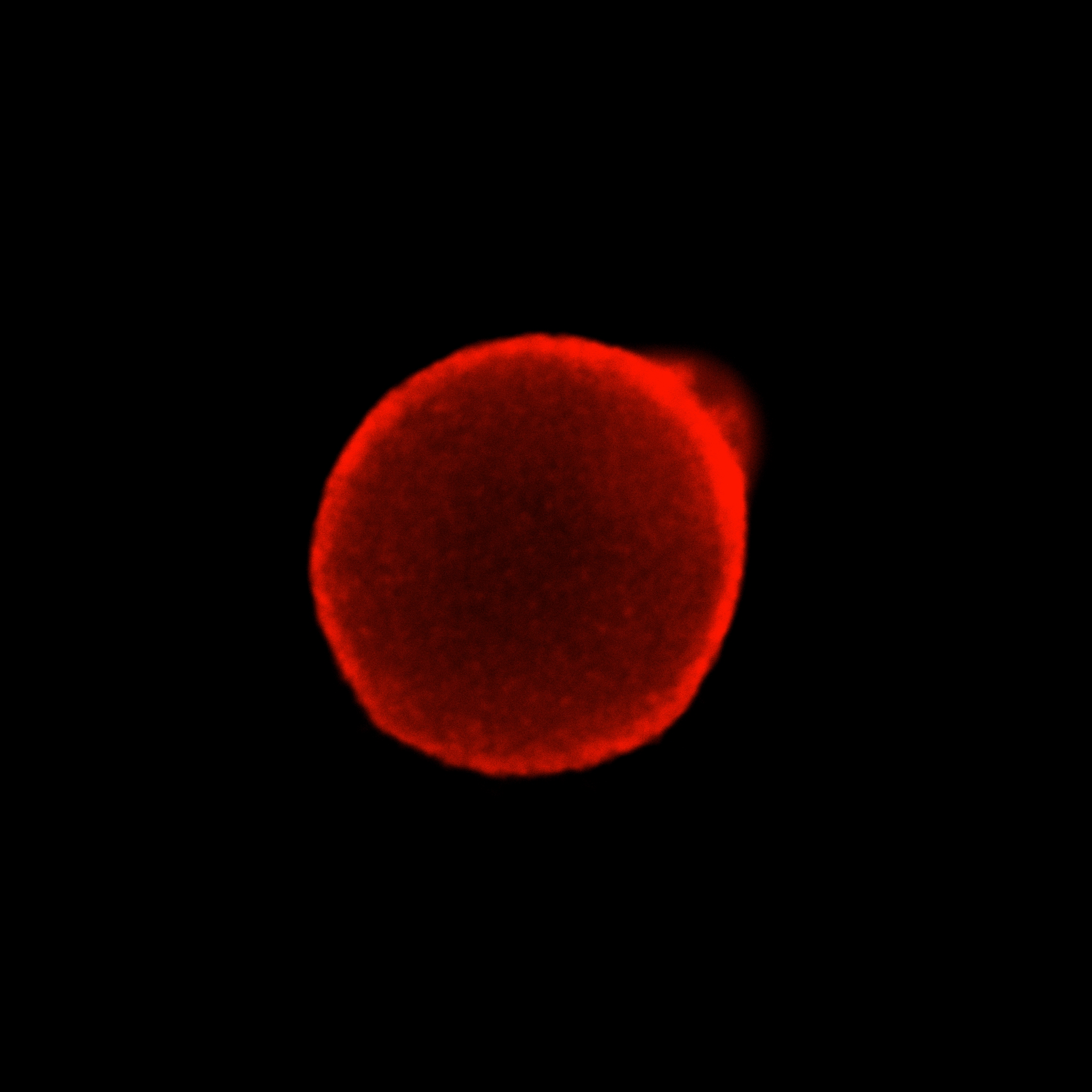

Supplement: Supplementary file 10 — Source data Fig. 8 [file 44318_2025_487_MOESM10_ESM.zip › Figure 8/8A CIM0216/TRPM3.jpg]

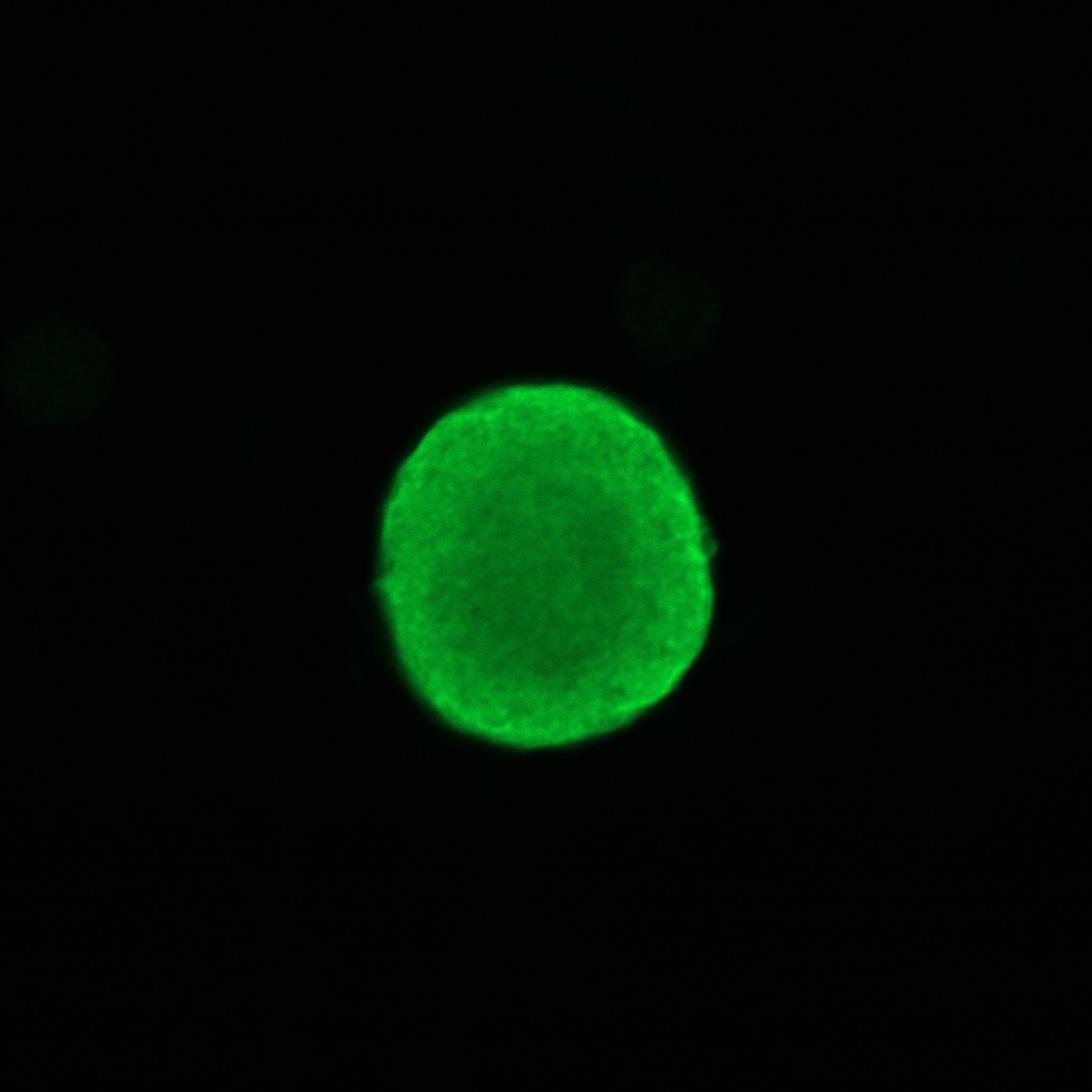

Supplement: Supplementary file 10 — Source data Fig. 8 [file 44318_2025_487_MOESM10_ESM.zip › Figure 8/8A control/Copine-6.jpg]

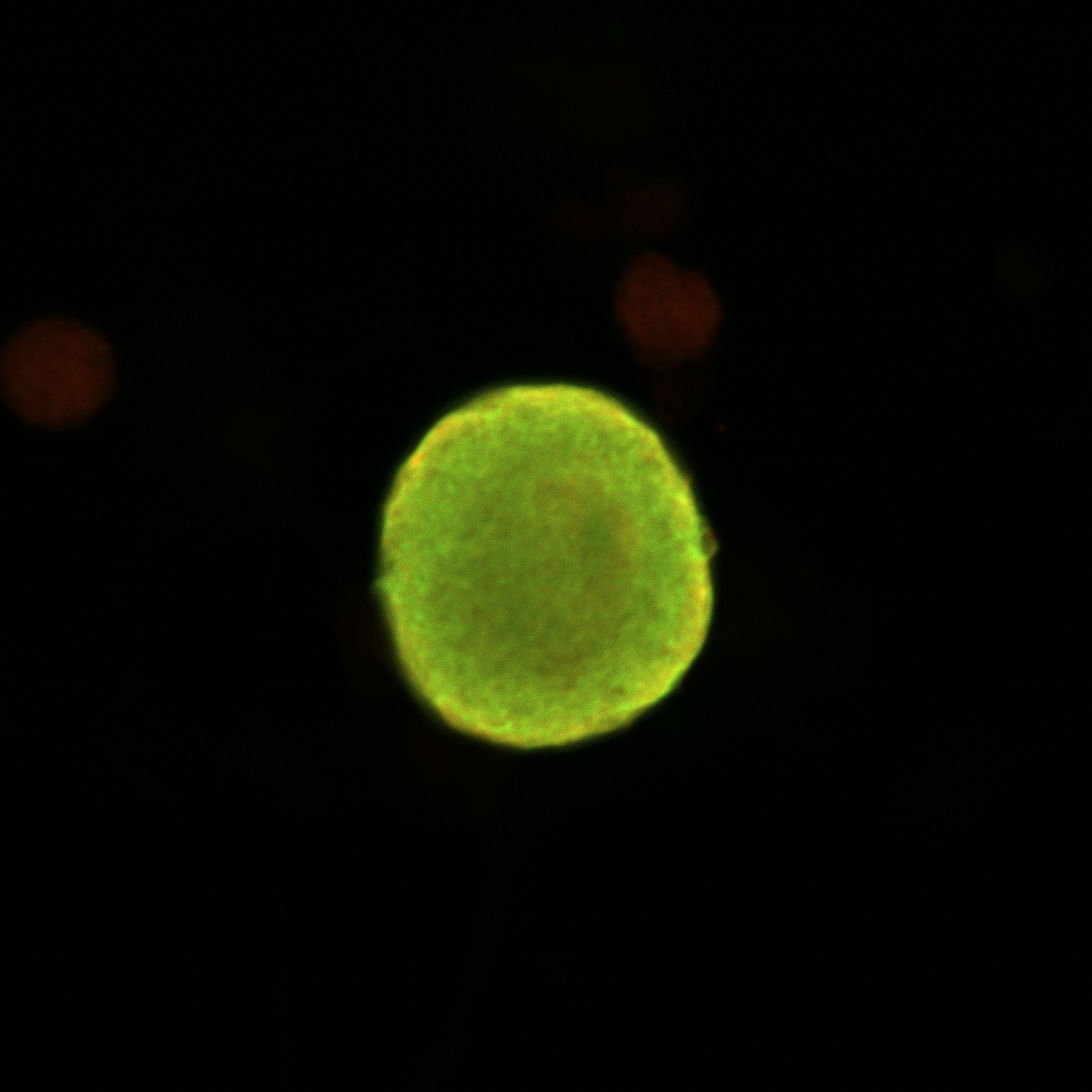

Supplement: Supplementary file 10 — Source data Fig. 8 [file 44318_2025_487_MOESM10_ESM.zip › Figure 8/8A control/merge.jpg]

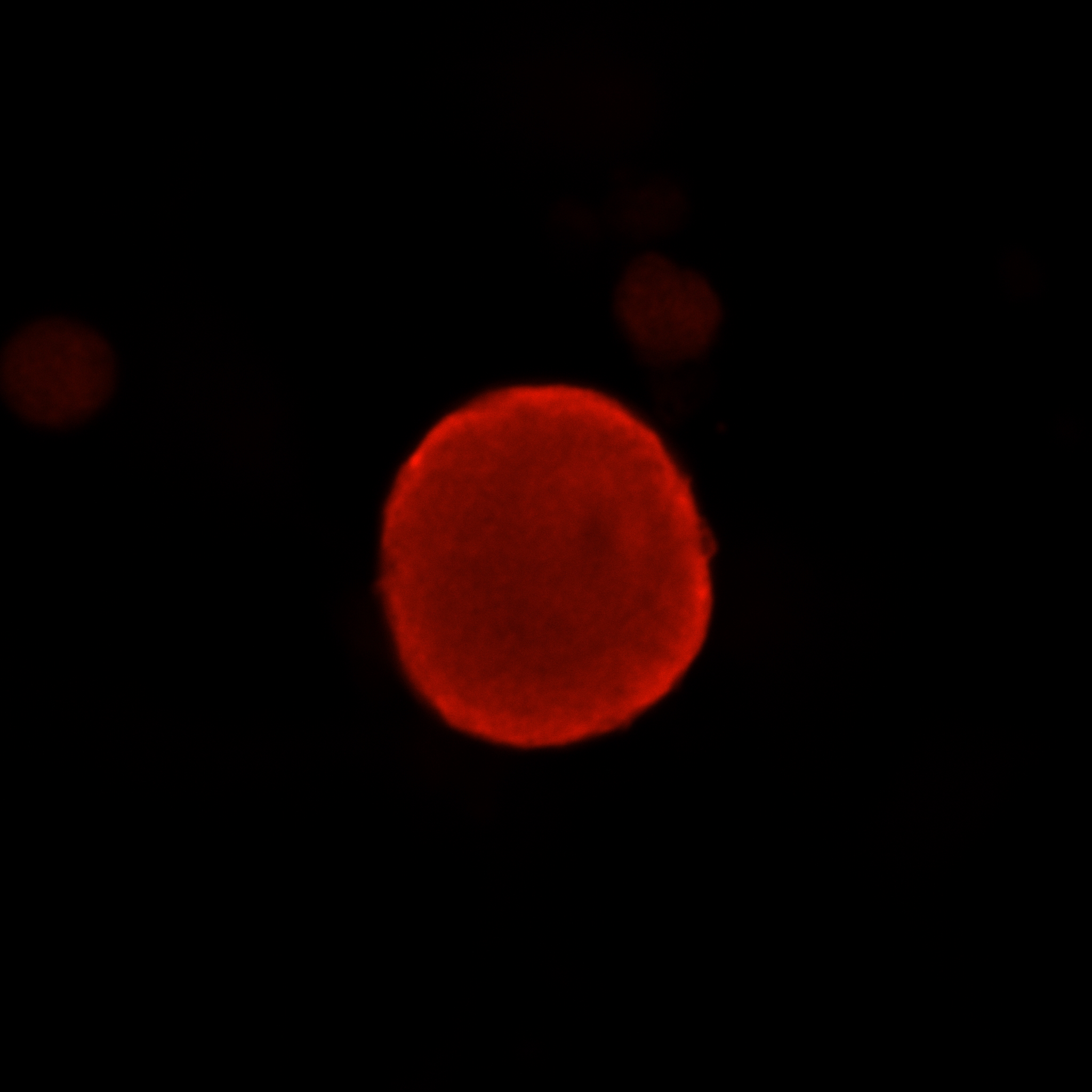

Supplement: Supplementary file 10 — Source data Fig. 8 [file 44318_2025_487_MOESM10_ESM.zip › Figure 8/8A control/TRPM3.jpg]

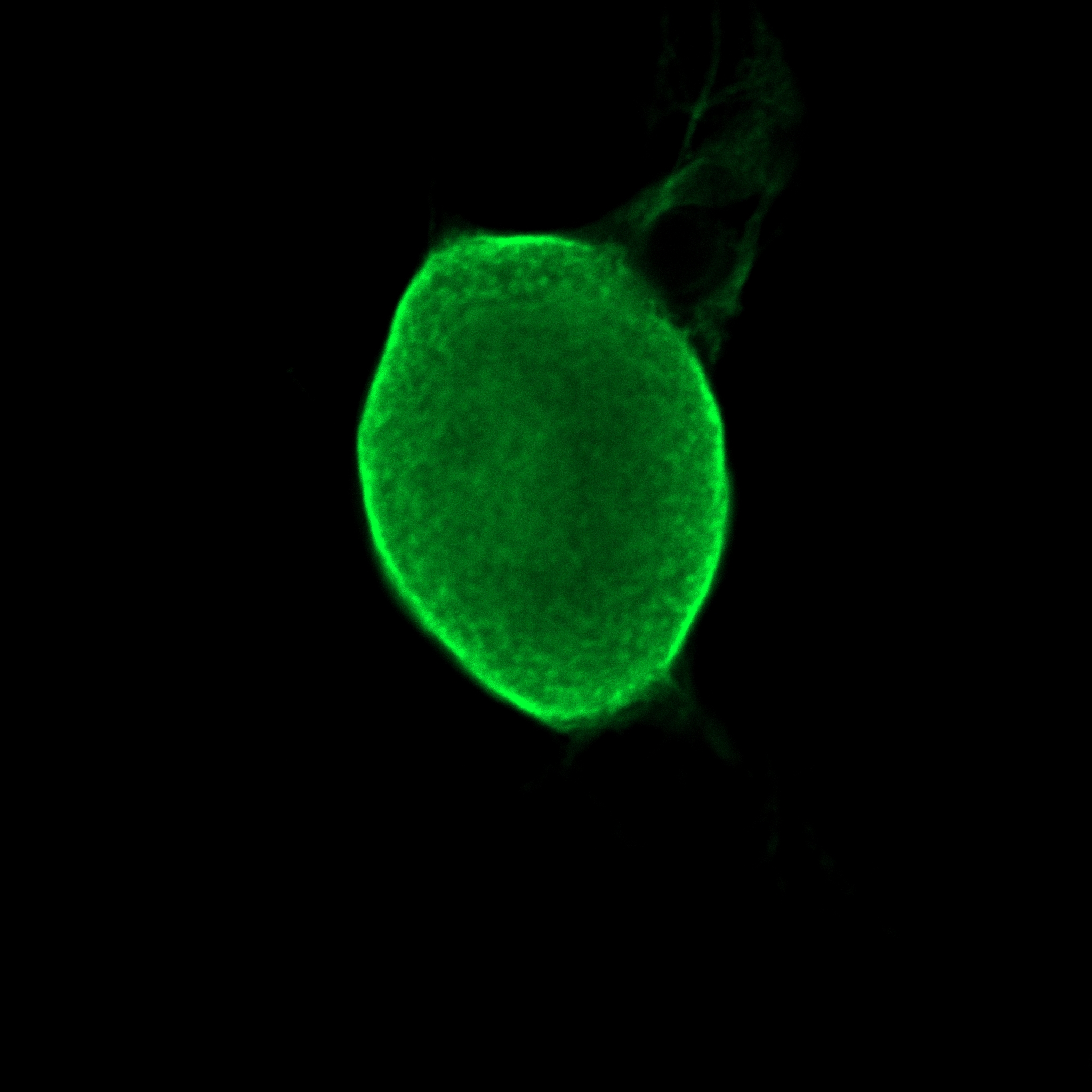

Supplement: Supplementary file 10 — Source data Fig. 8 [file 44318_2025_487_MOESM10_ESM.zip › Figure 8/8A ionomycin/Copine-6.jpg]

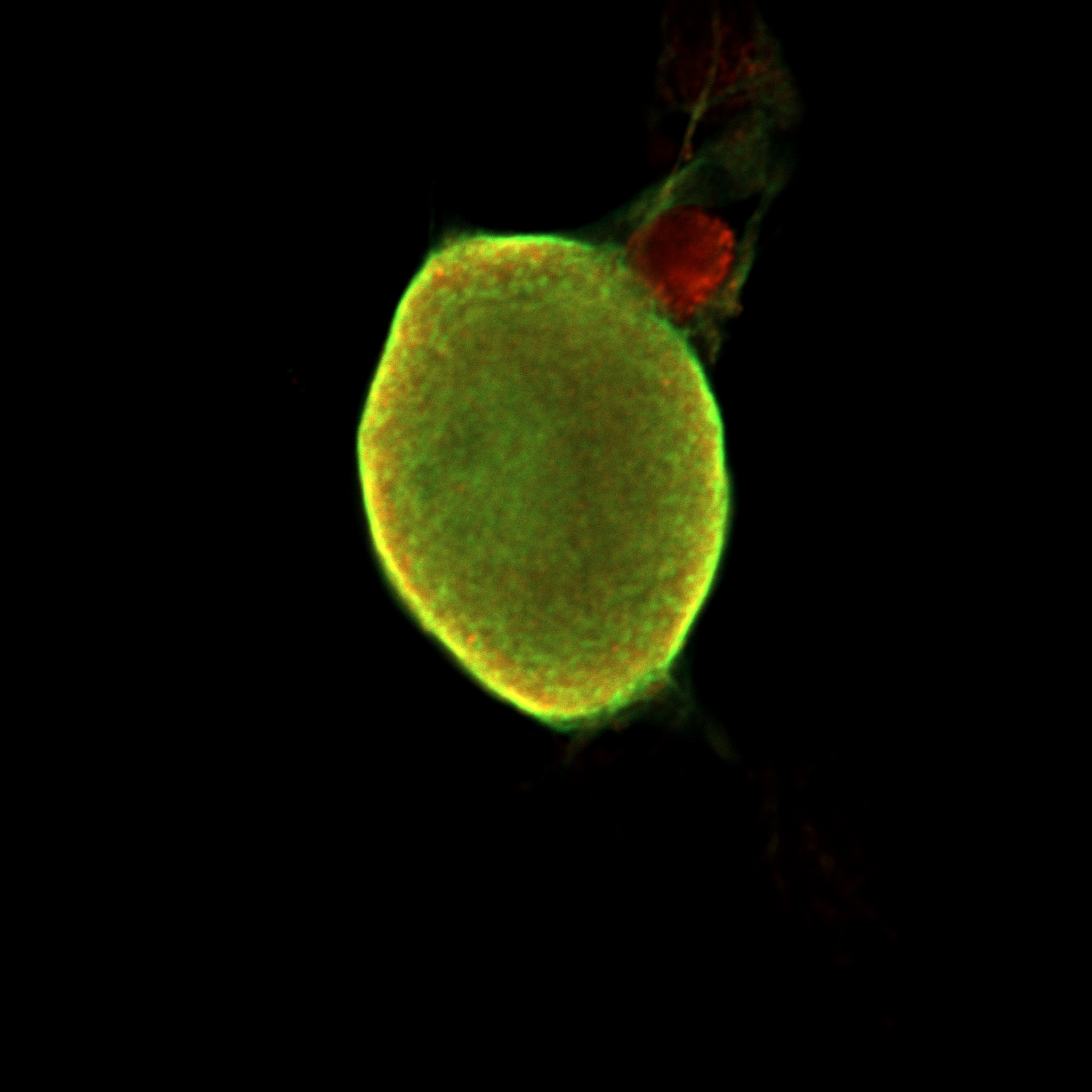

Supplement: Supplementary file 10 — Source data Fig. 8 [file 44318_2025_487_MOESM10_ESM.zip › Figure 8/8A ionomycin/merge.jpg]

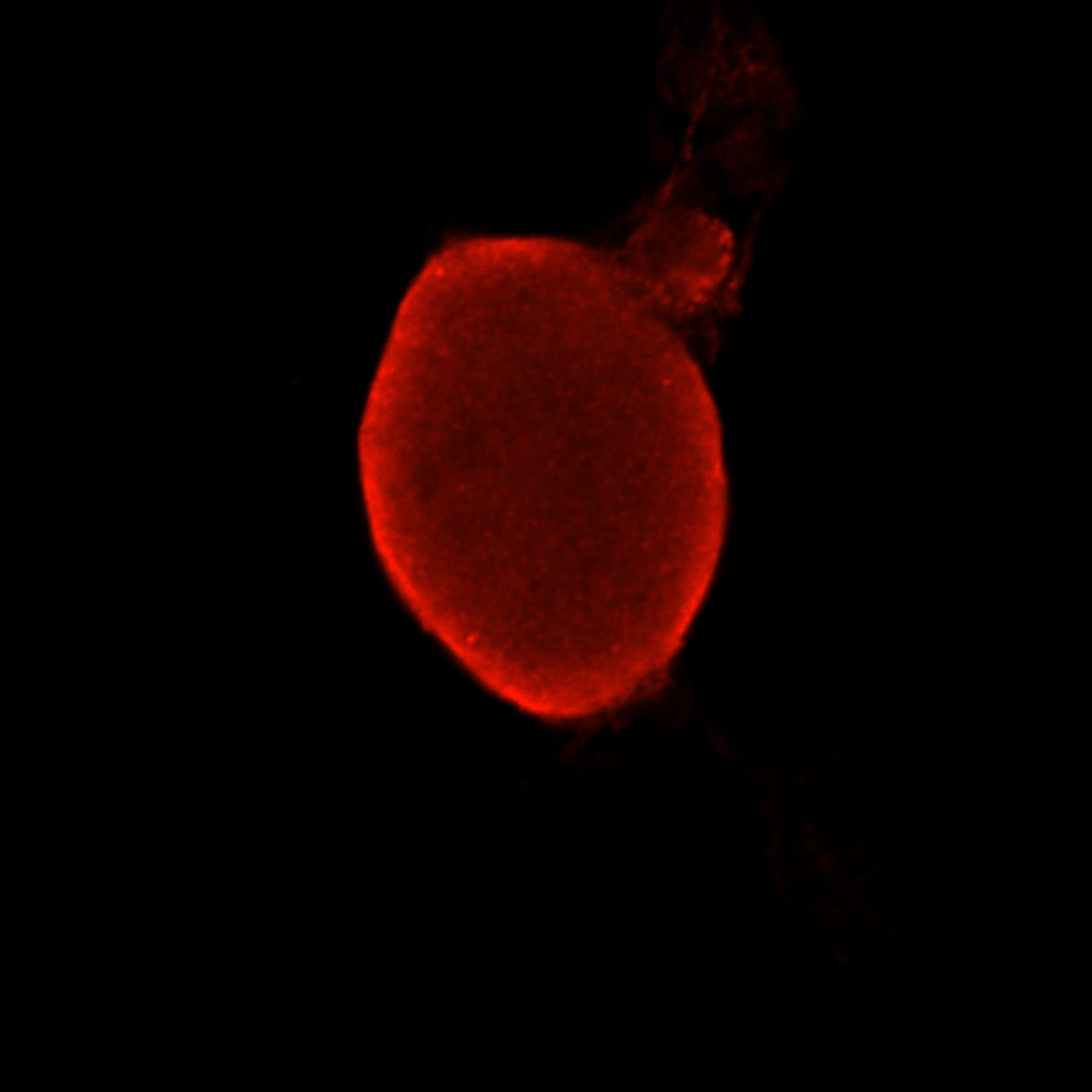

Supplement: Supplementary file 10 — Source data Fig. 8 [file 44318_2025_487_MOESM10_ESM.zip › Figure 8/8A ionomycin/TRPM3.jpg]

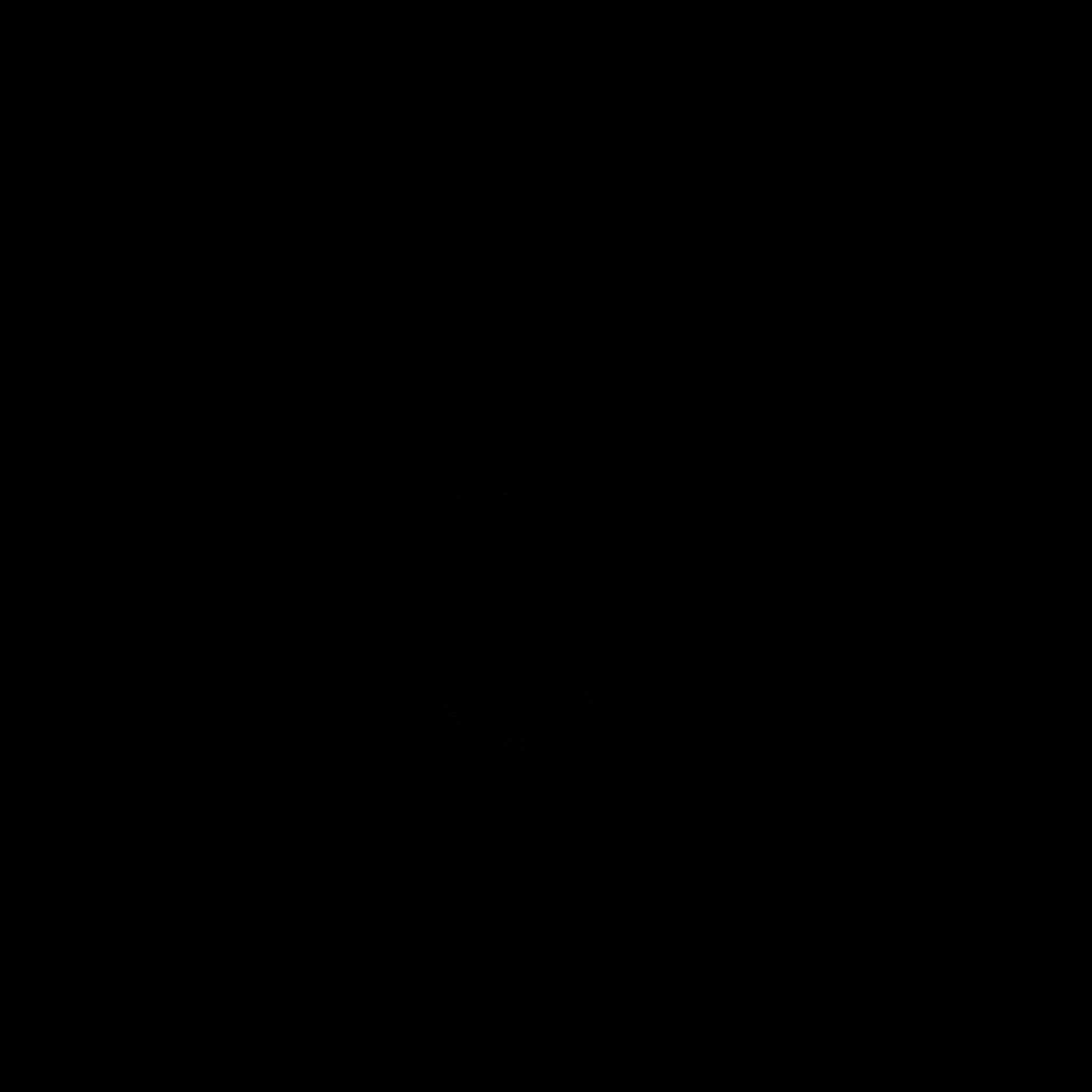

Supplement: Supplementary file 10 — Source data Fig. 8 [file 44318_2025_487_MOESM10_ESM.zip › Figure 8/8B 45/Copine-6.jpg]

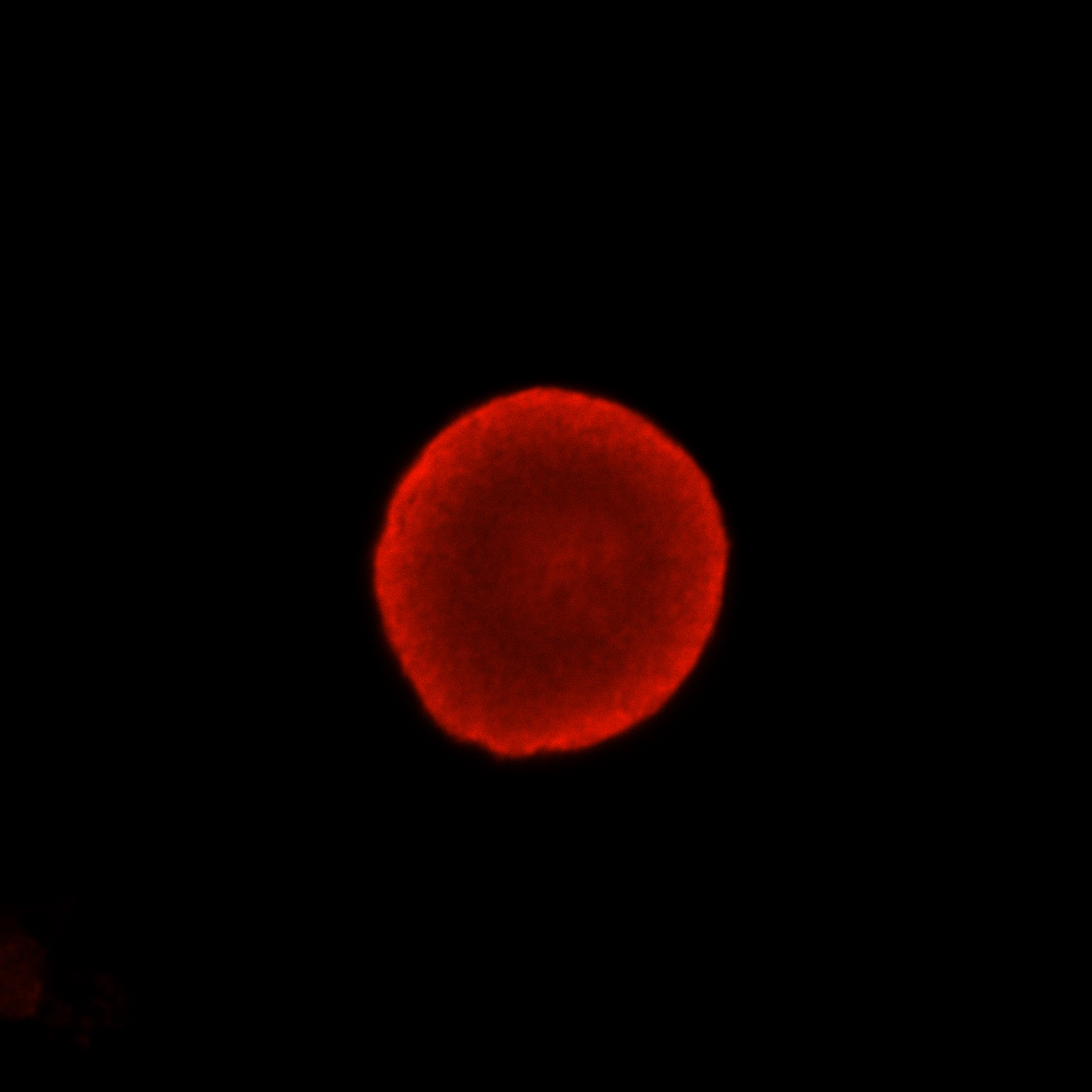

Supplement: Supplementary file 10 — Source data Fig. 8 [file 44318_2025_487_MOESM10_ESM.zip › Figure 8/8B 45/merge.jpg]

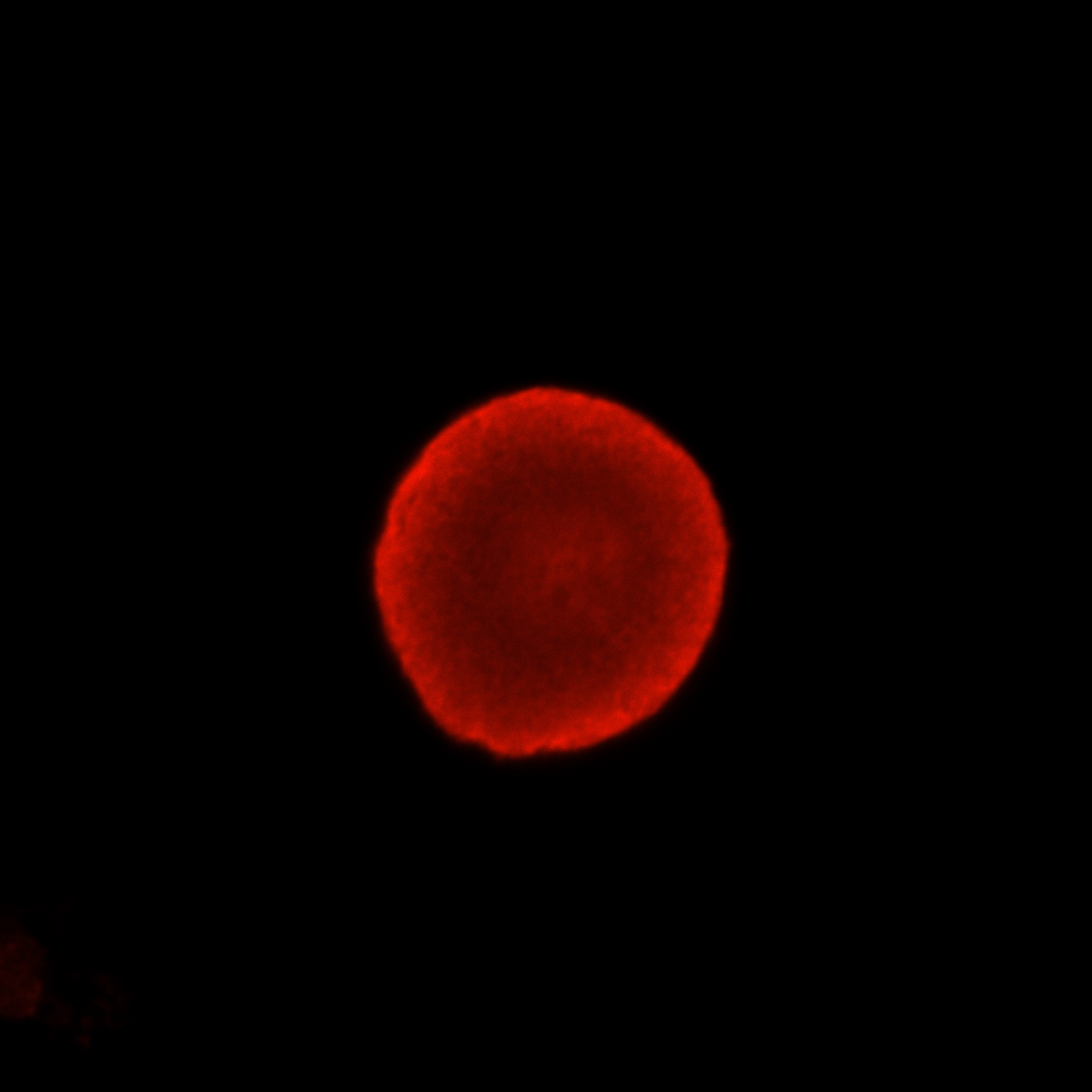

Supplement: Supplementary file 10 — Source data Fig. 8 [file 44318_2025_487_MOESM10_ESM.zip › Figure 8/8B 45/TRPM3.jpg]

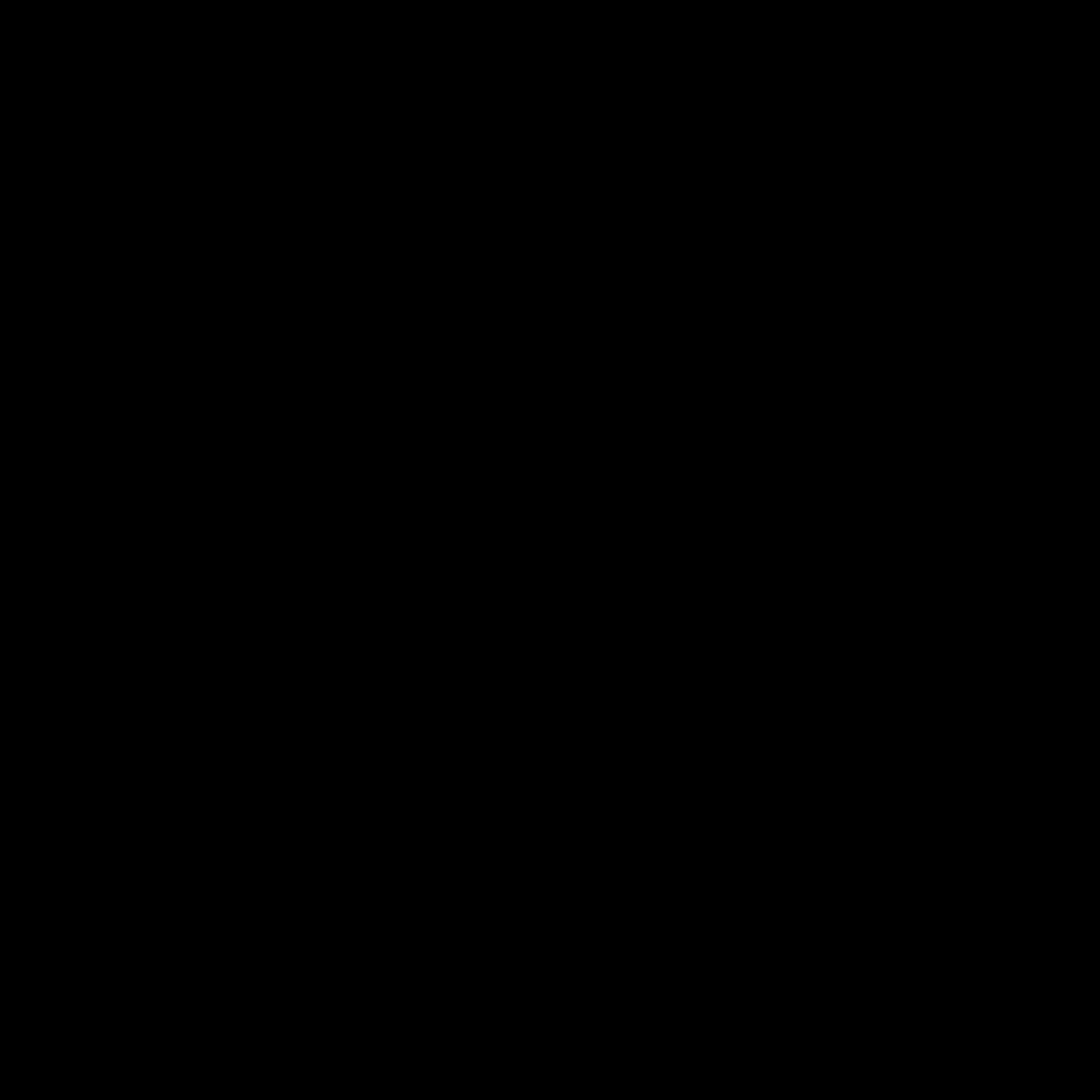

Supplement: Supplementary file 10 — Source data Fig. 8 [file 44318_2025_487_MOESM10_ESM.zip › Figure 8/8B CIM0216/Copine-6.jpg]

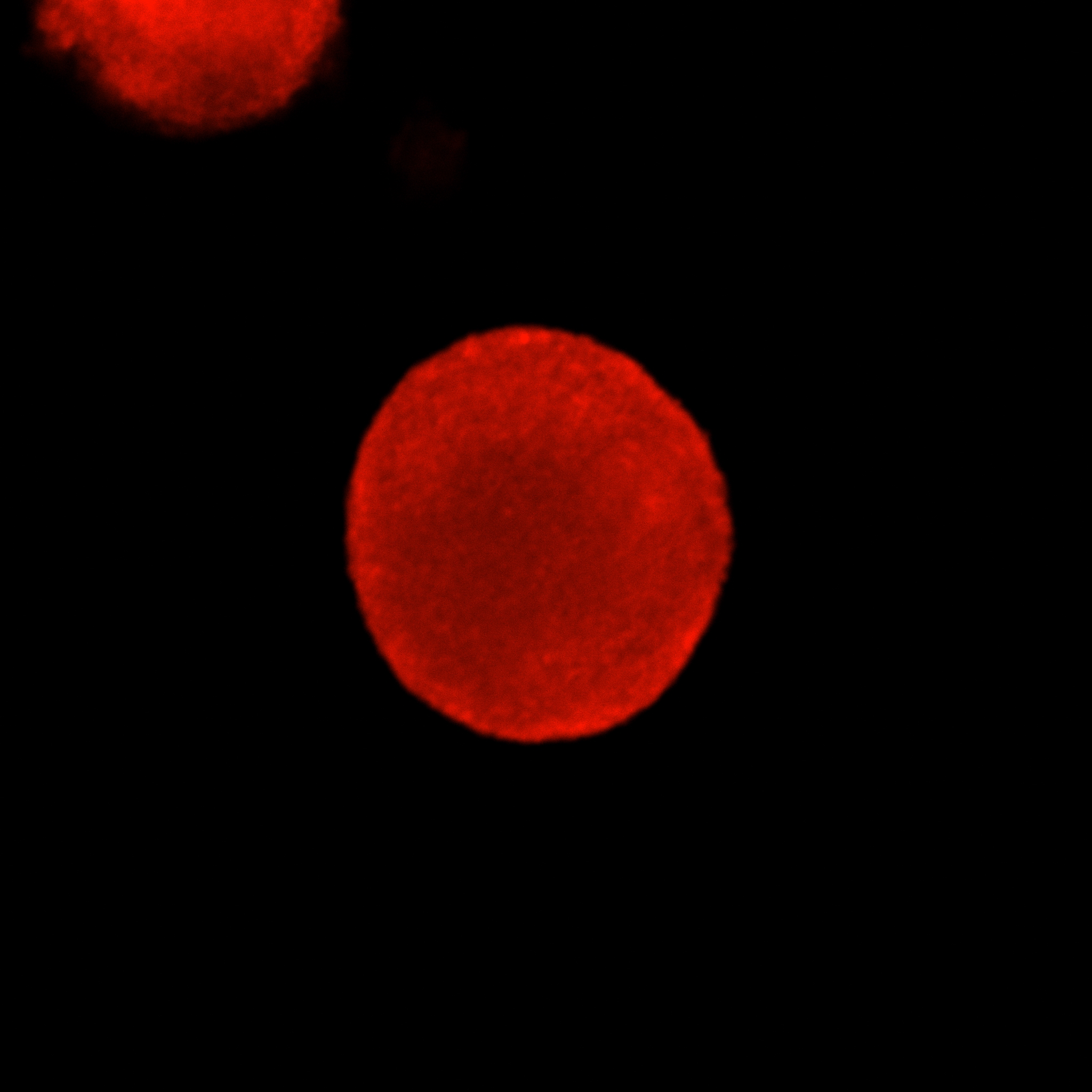

Supplement: Supplementary file 10 — Source data Fig. 8 [file 44318_2025_487_MOESM10_ESM.zip › Figure 8/8B CIM0216/merge.jpg]

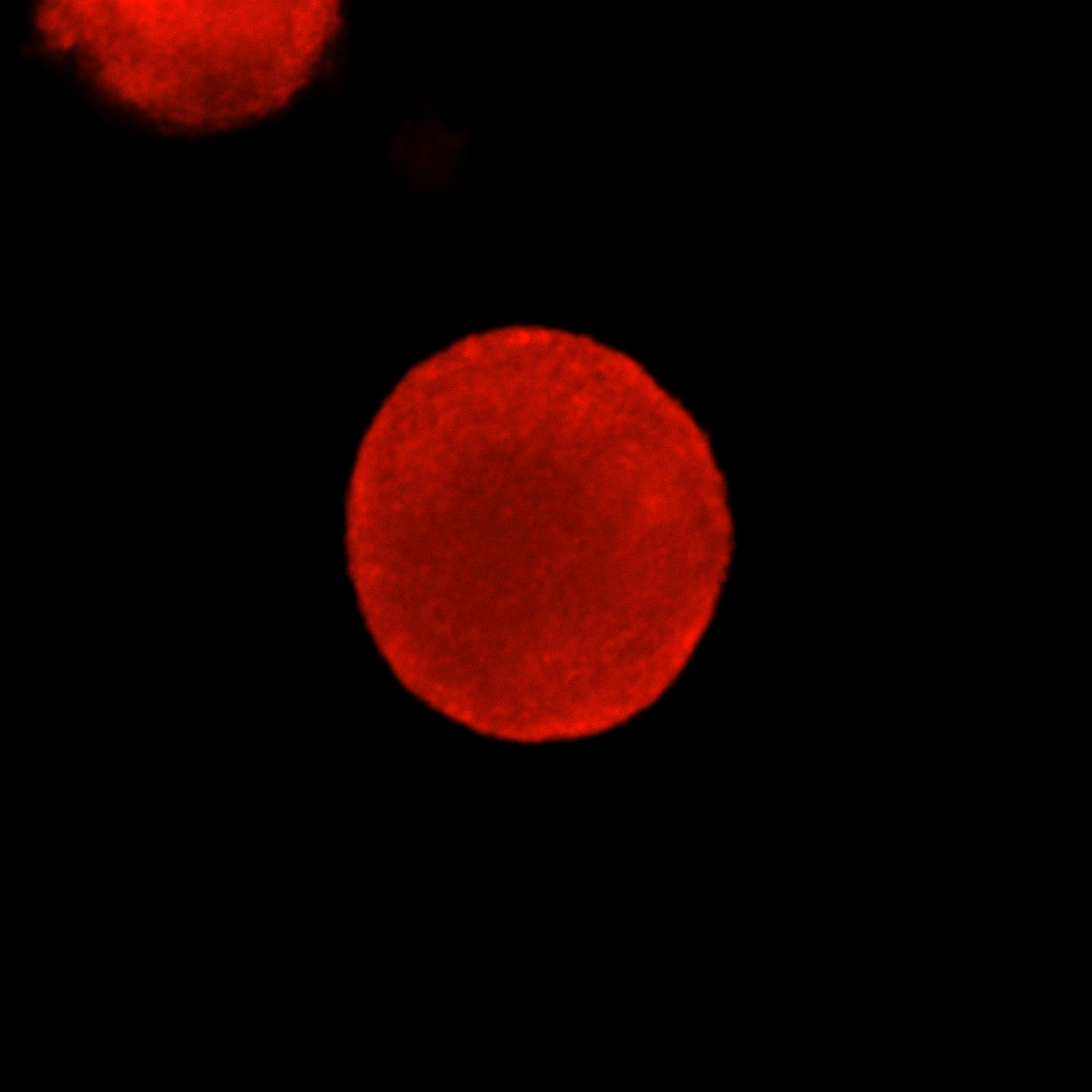

Supplement: Supplementary file 10 — Source data Fig. 8 [file 44318_2025_487_MOESM10_ESM.zip › Figure 8/8B CIM0216/TRPM3.jpg]

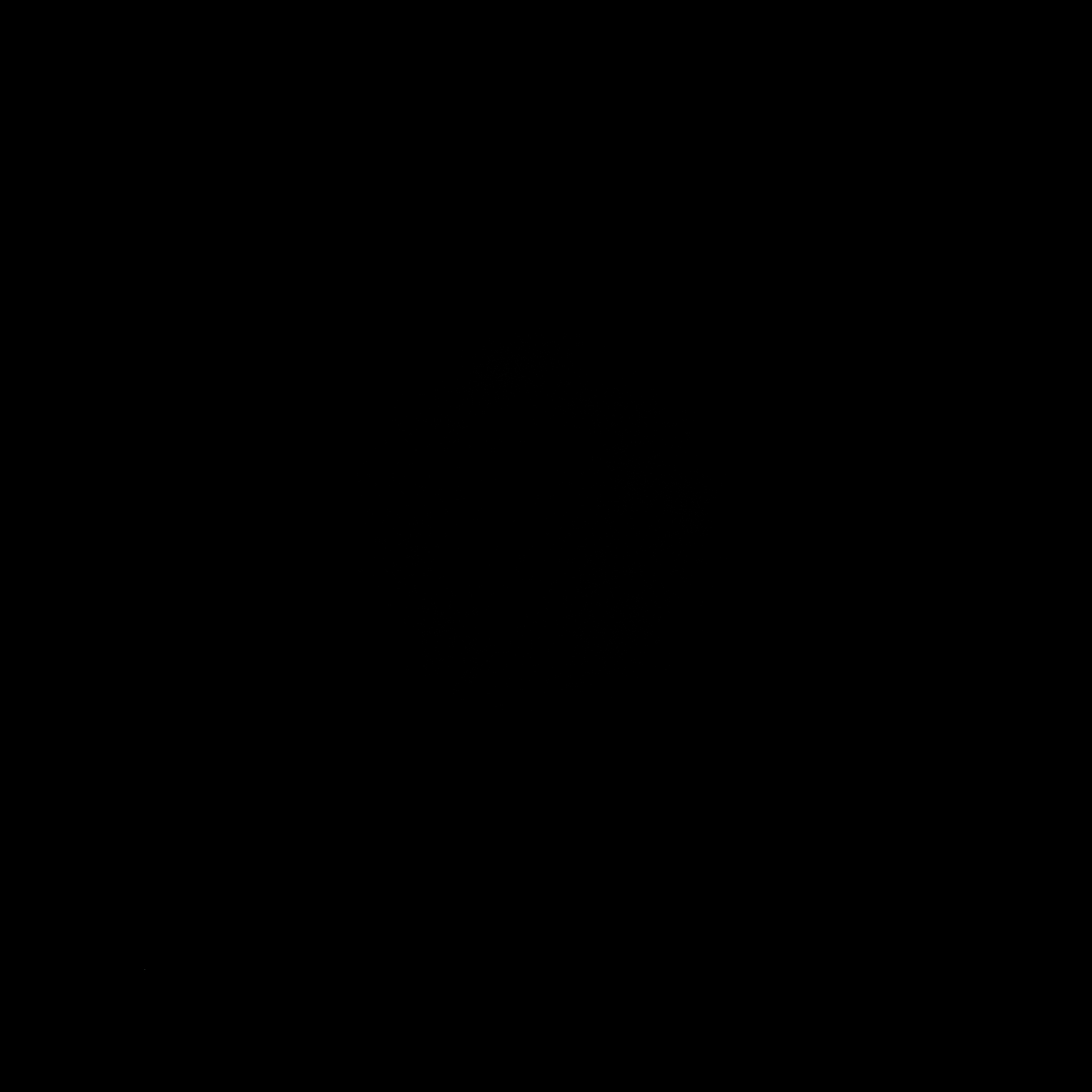

Supplement: Supplementary file 10 — Source data Fig. 8 [file 44318_2025_487_MOESM10_ESM.zip › Figure 8/8B control/Copine-6.jpg]

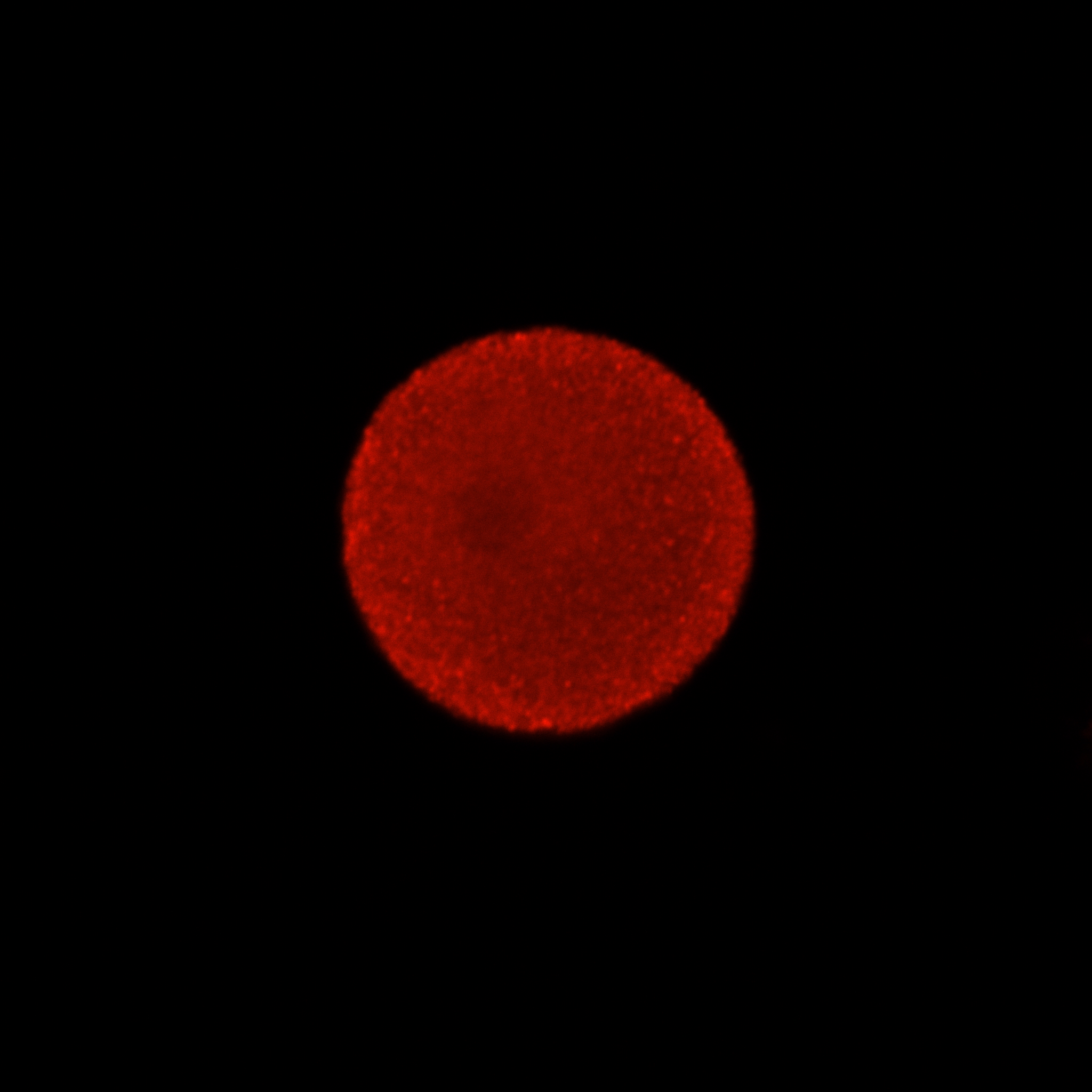

Supplement: Supplementary file 10 — Source data Fig. 8 [file 44318_2025_487_MOESM10_ESM.zip › Figure 8/8B control/merge.jpg]

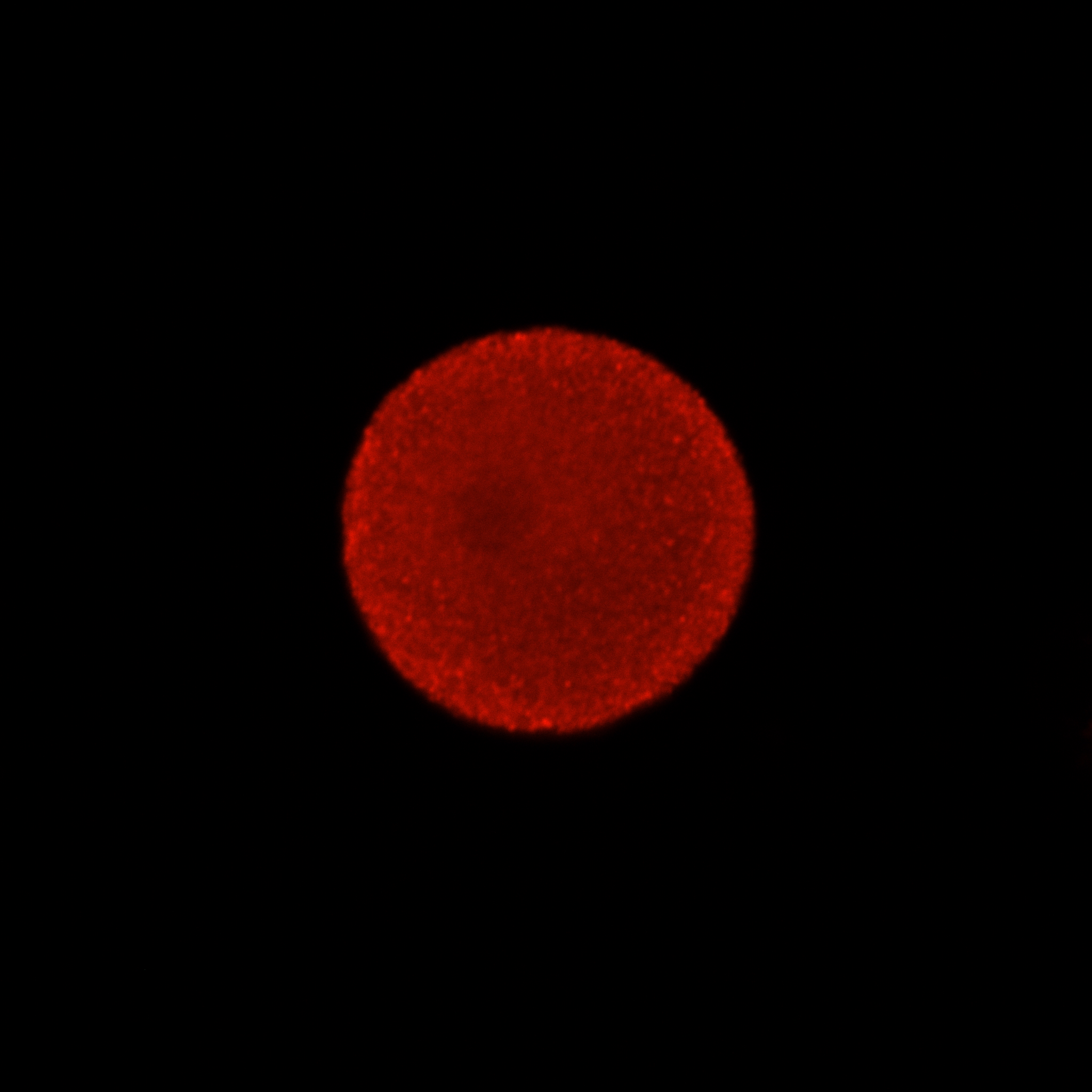

Supplement: Supplementary file 10 — Source data Fig. 8 [file 44318_2025_487_MOESM10_ESM.zip › Figure 8/8B control/TRPM3.jpg]

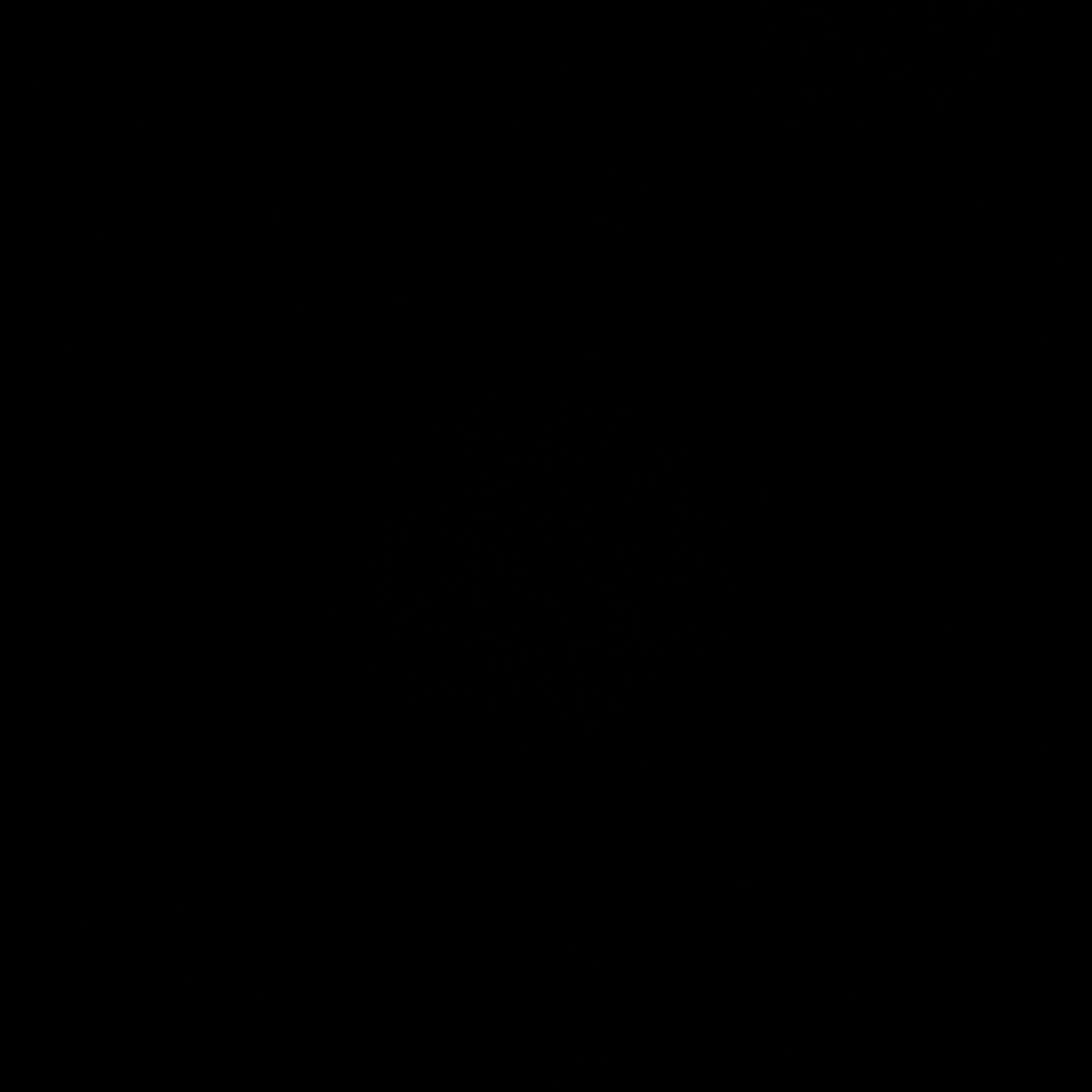

Supplement: Supplementary file 10 — Source data Fig. 8 [file 44318_2025_487_MOESM10_ESM.zip › Figure 8/8B ionomycin/Copine-6.jpg]

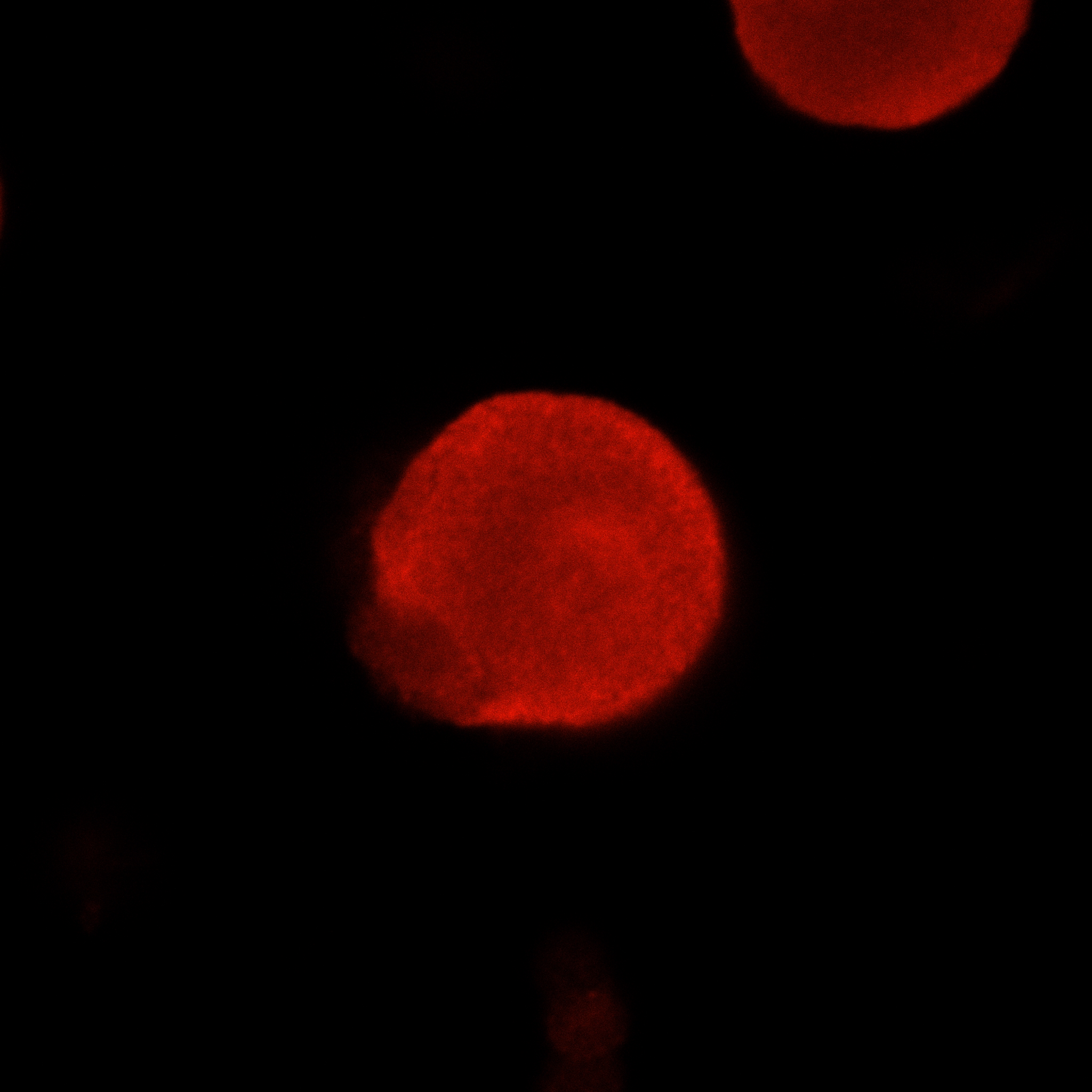

Supplement: Supplementary file 10 — Source data Fig. 8 [file 44318_2025_487_MOESM10_ESM.zip › Figure 8/8B ionomycin/merge.jpg]

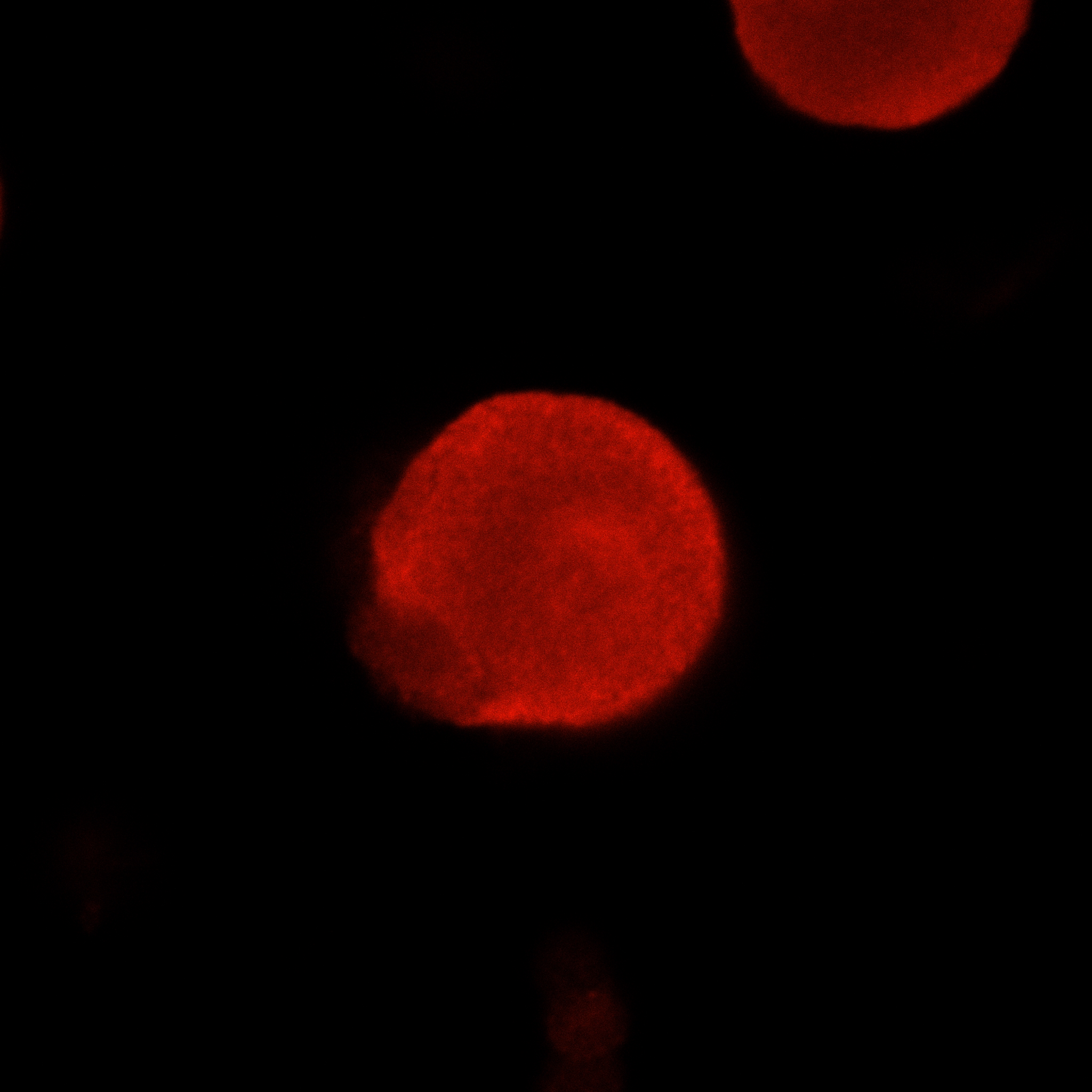

Supplement: Supplementary file 10 — Source data Fig. 8 [file 44318_2025_487_MOESM10_ESM.zip › Figure 8/8B ionomycin/TRPM3.jpg]

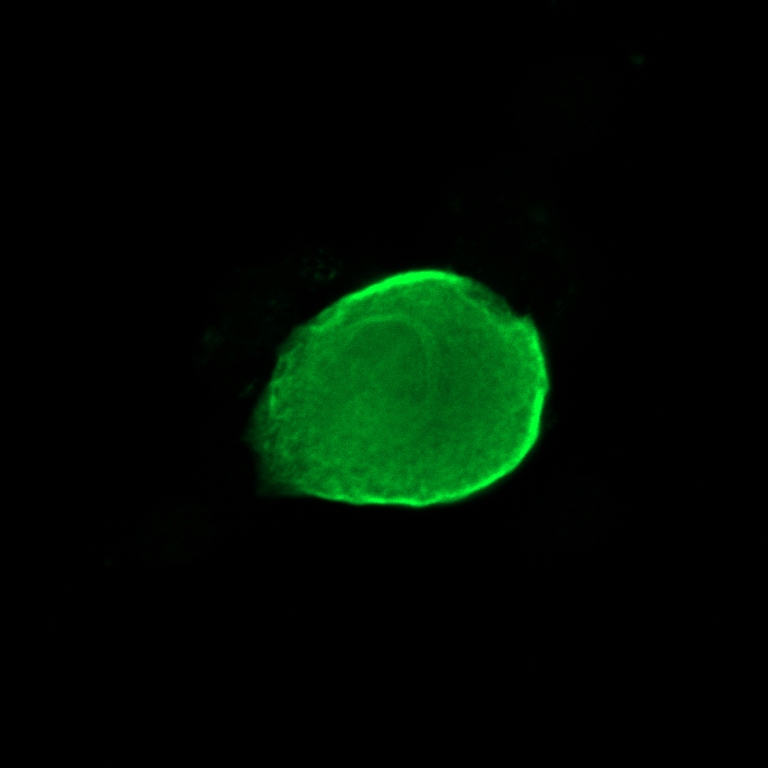

Supplement: Supplementary file 10 — Source data Fig. 8 [file 44318_2025_487_MOESM10_ESM.zip › Figure 8/8C 45/Copine-6.jpg]

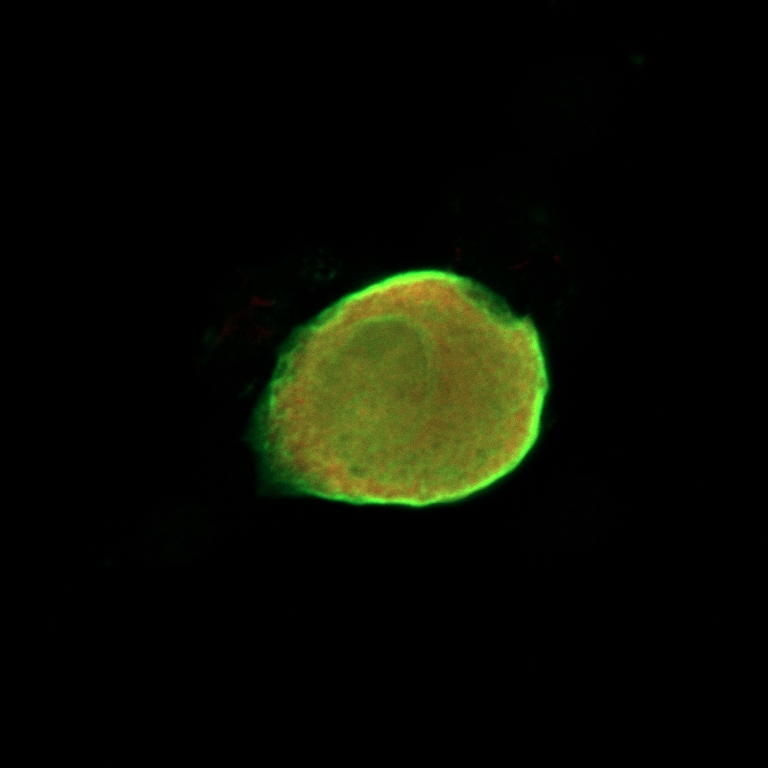

Supplement: Supplementary file 10 — Source data Fig. 8 [file 44318_2025_487_MOESM10_ESM.zip › Figure 8/8C 45/merge.jpg]

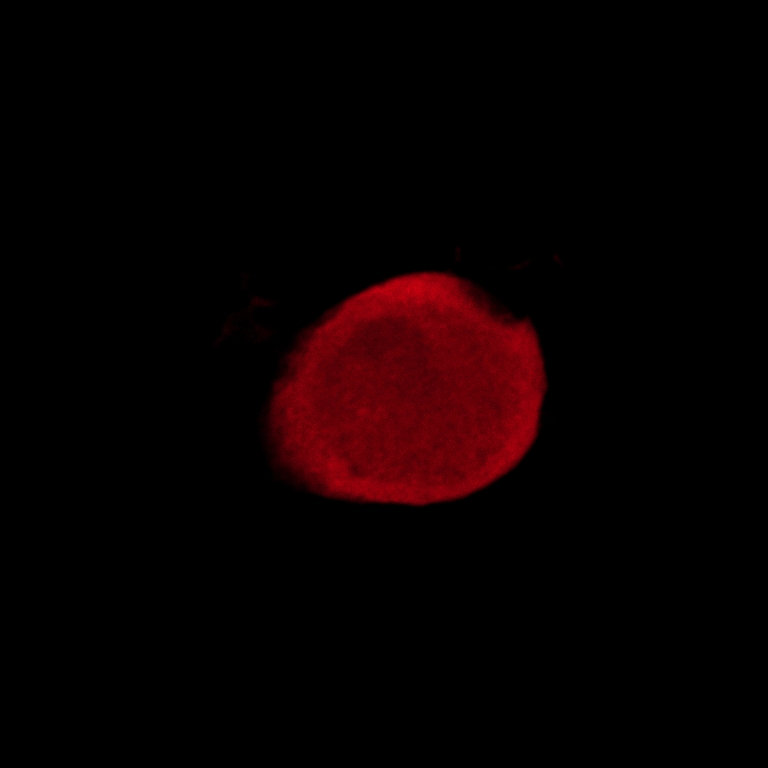

Supplement: Supplementary file 10 — Source data Fig. 8 [file 44318_2025_487_MOESM10_ESM.zip › Figure 8/8C 45/TRPV1.jpg]

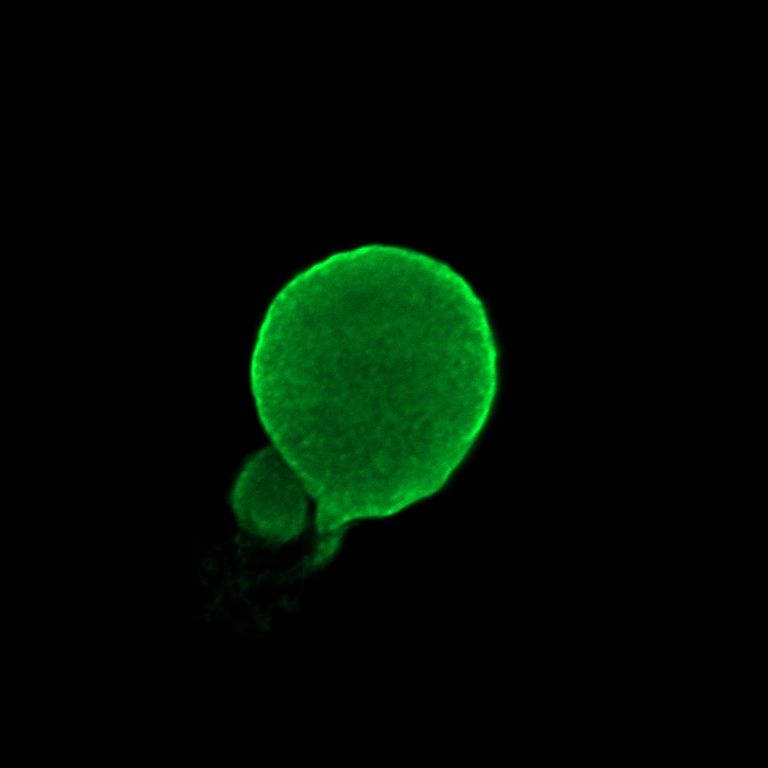

Supplement: Supplementary file 10 — Source data Fig. 8 [file 44318_2025_487_MOESM10_ESM.zip › Figure 8/8C capsaicin/Copine-6.jpg]

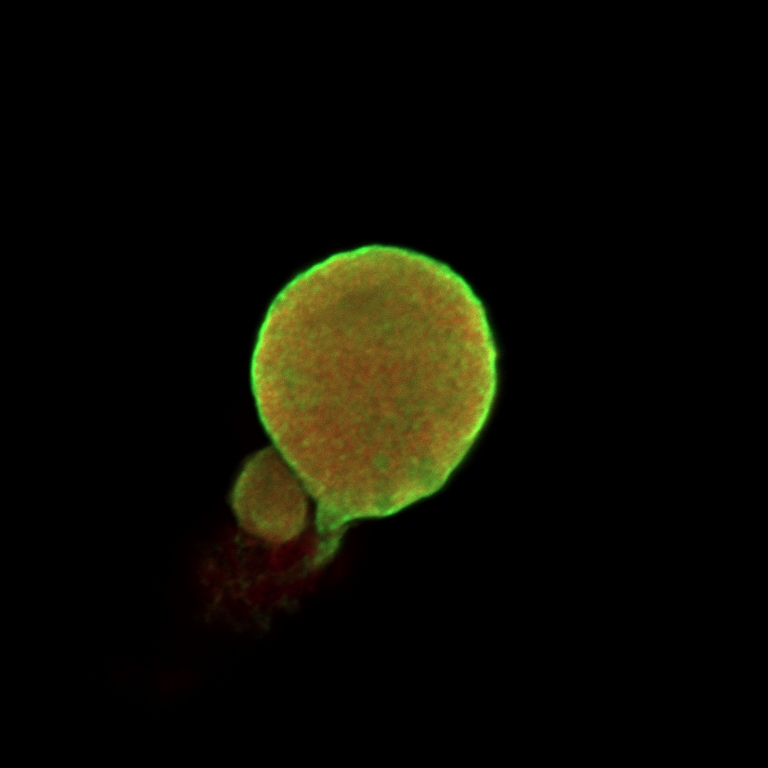

Supplement: Supplementary file 10 — Source data Fig. 8 [file 44318_2025_487_MOESM10_ESM.zip › Figure 8/8C capsaicin/merge.jpg]

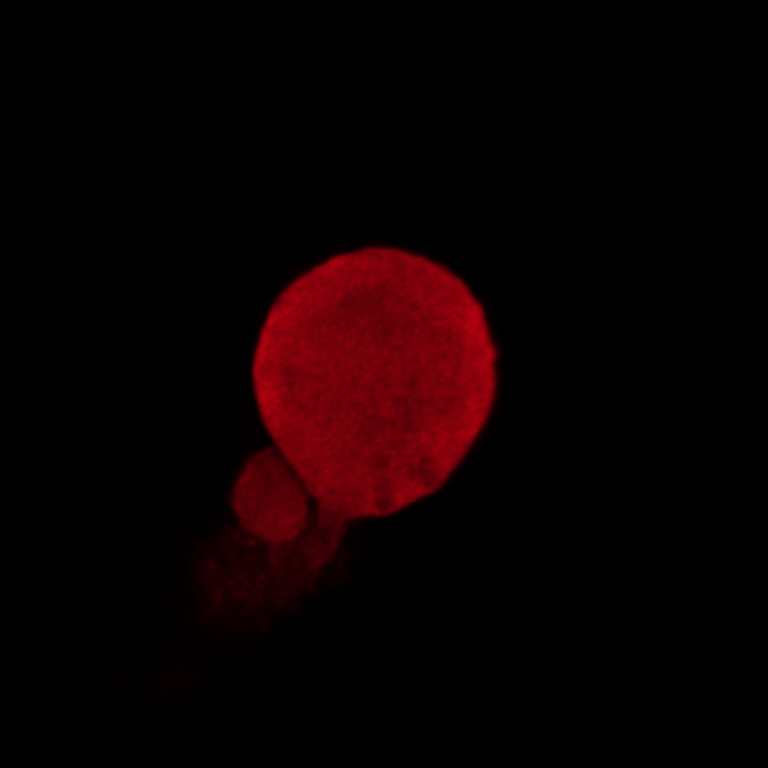

Supplement: Supplementary file 10 — Source data Fig. 8 [file 44318_2025_487_MOESM10_ESM.zip › Figure 8/8C capsaicin/TRPV1.jpg]

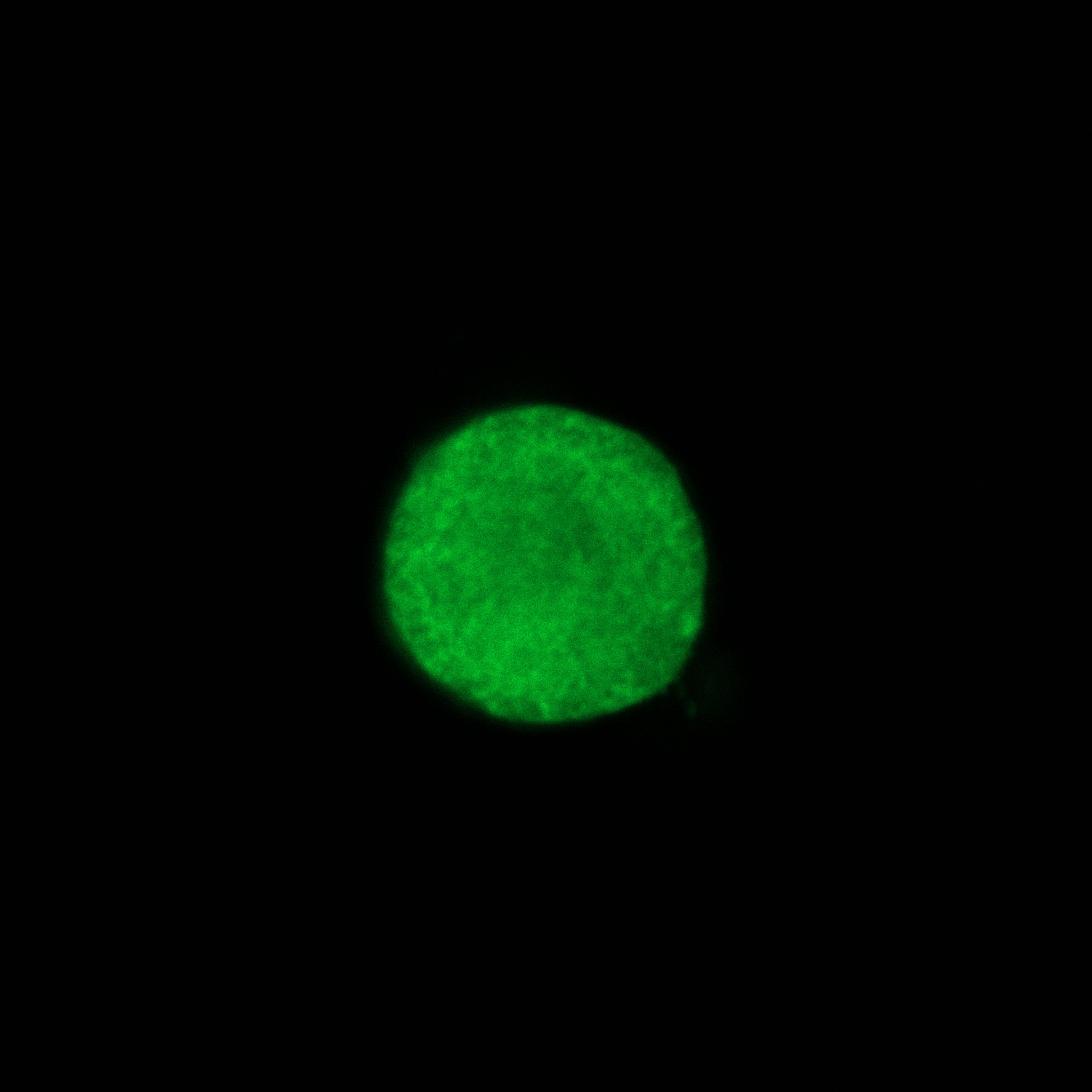

Supplement: Supplementary file 10 — Source data Fig. 8 [file 44318_2025_487_MOESM10_ESM.zip › Figure 8/8C control/Copine-6.jpg]

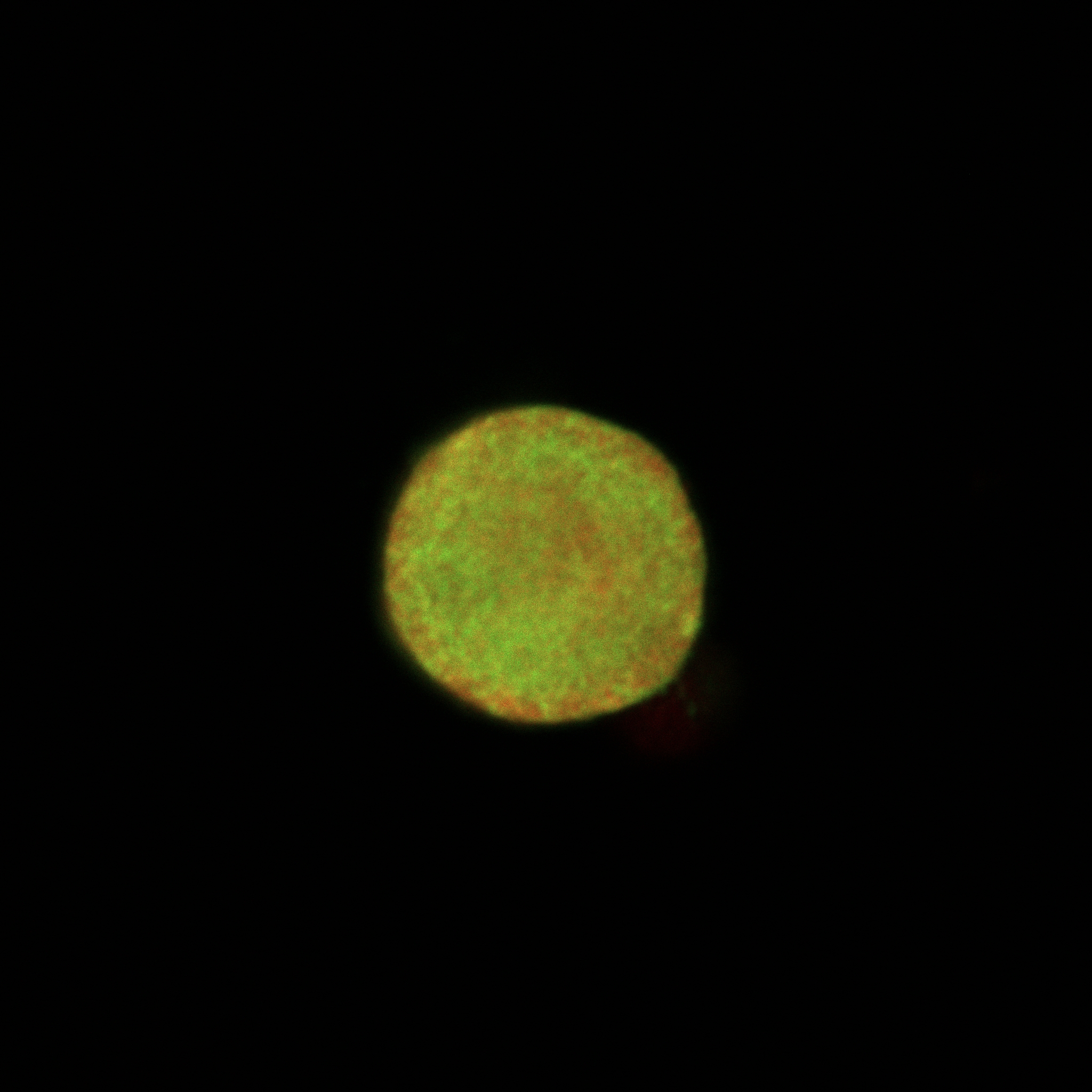

Supplement: Supplementary file 10 — Source data Fig. 8 [file 44318_2025_487_MOESM10_ESM.zip › Figure 8/8C control/merge.jpg]

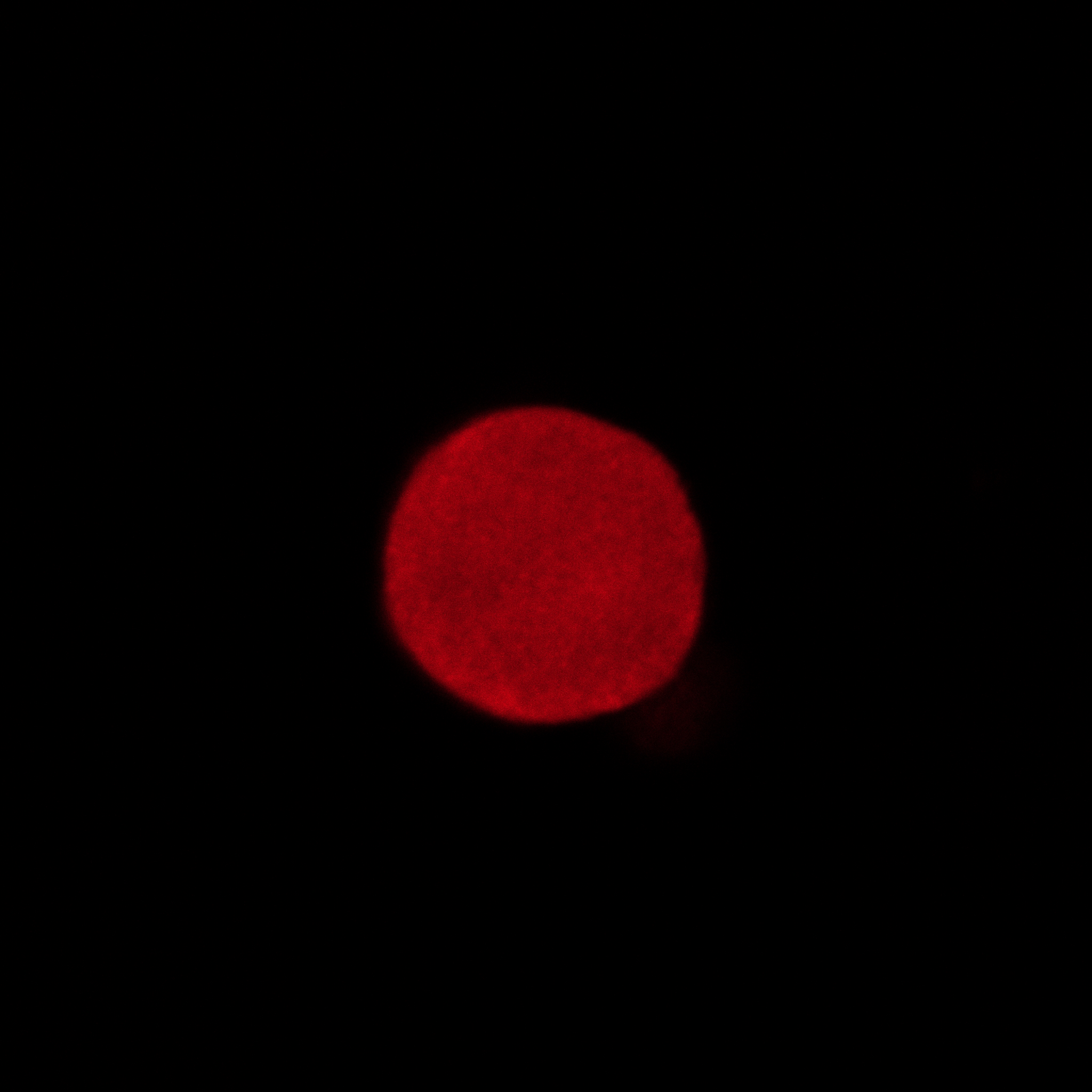

Supplement: Supplementary file 10 — Source data Fig. 8 [file 44318_2025_487_MOESM10_ESM.zip › Figure 8/8C control/TRPV1.jpg]

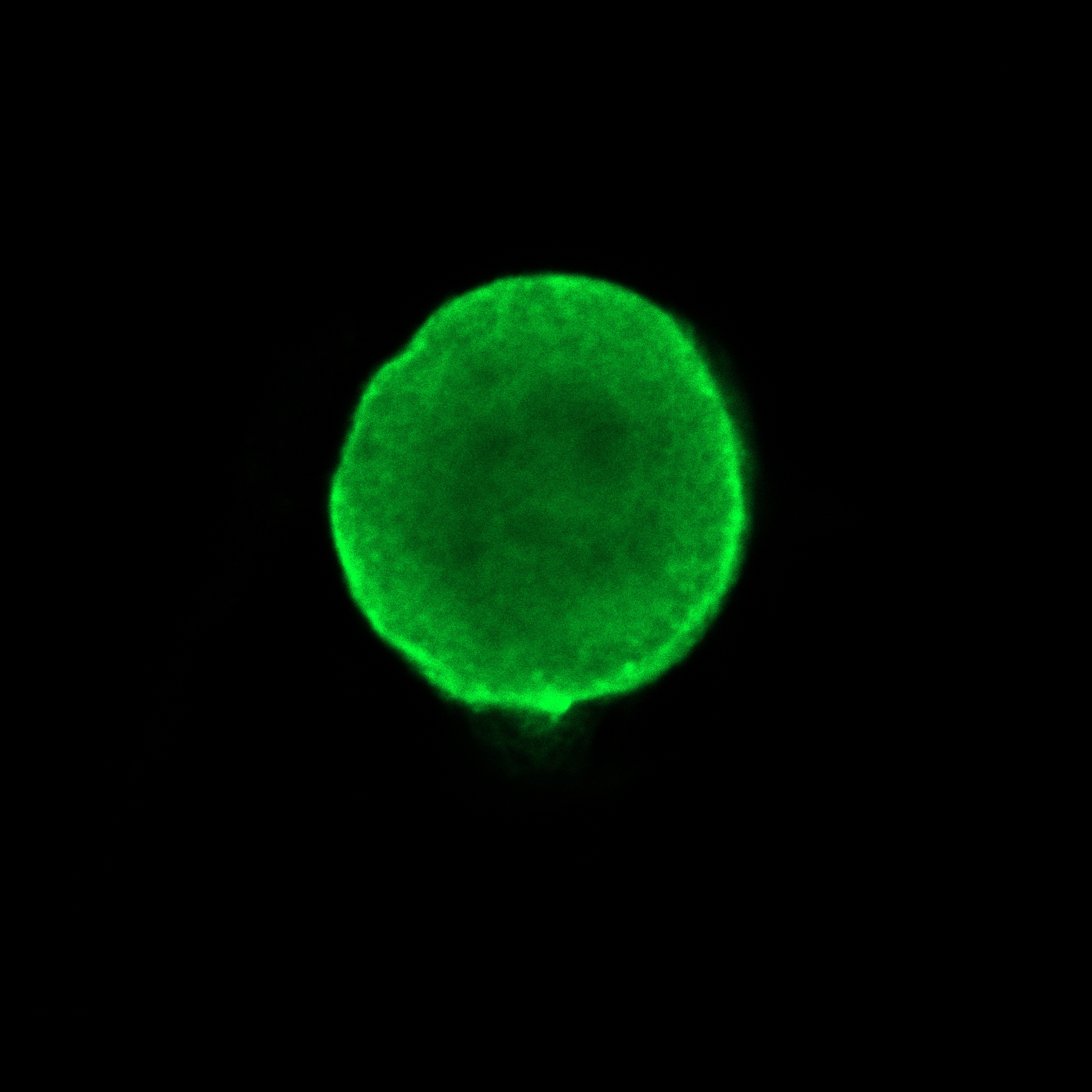

Supplement: Supplementary file 10 — Source data Fig. 8 [file 44318_2025_487_MOESM10_ESM.zip › Figure 8/8C ionomycin/Copine-6.jpg]

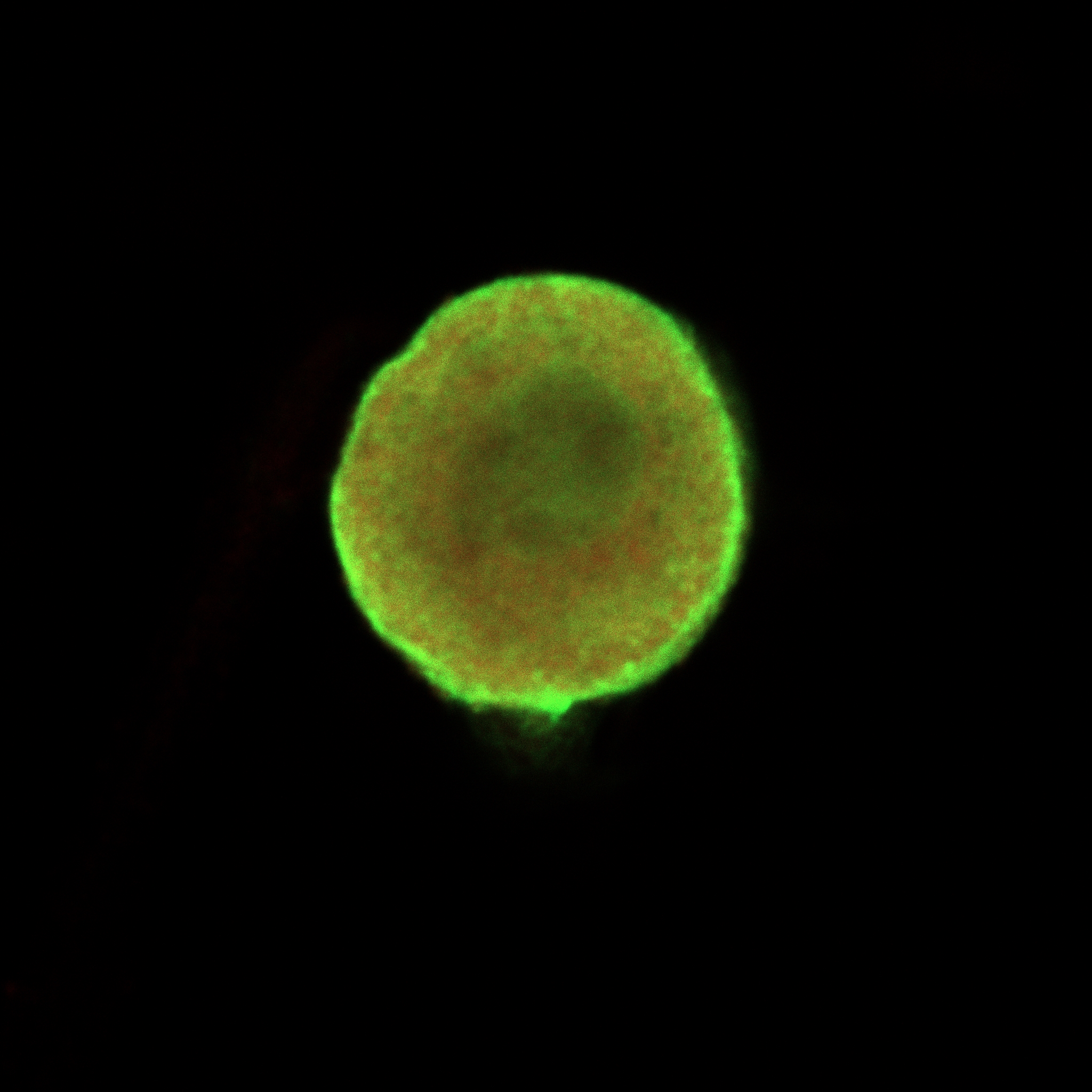

Supplement: Supplementary file 10 — Source data Fig. 8 [file 44318_2025_487_MOESM10_ESM.zip › Figure 8/8C ionomycin/merge.jpg]

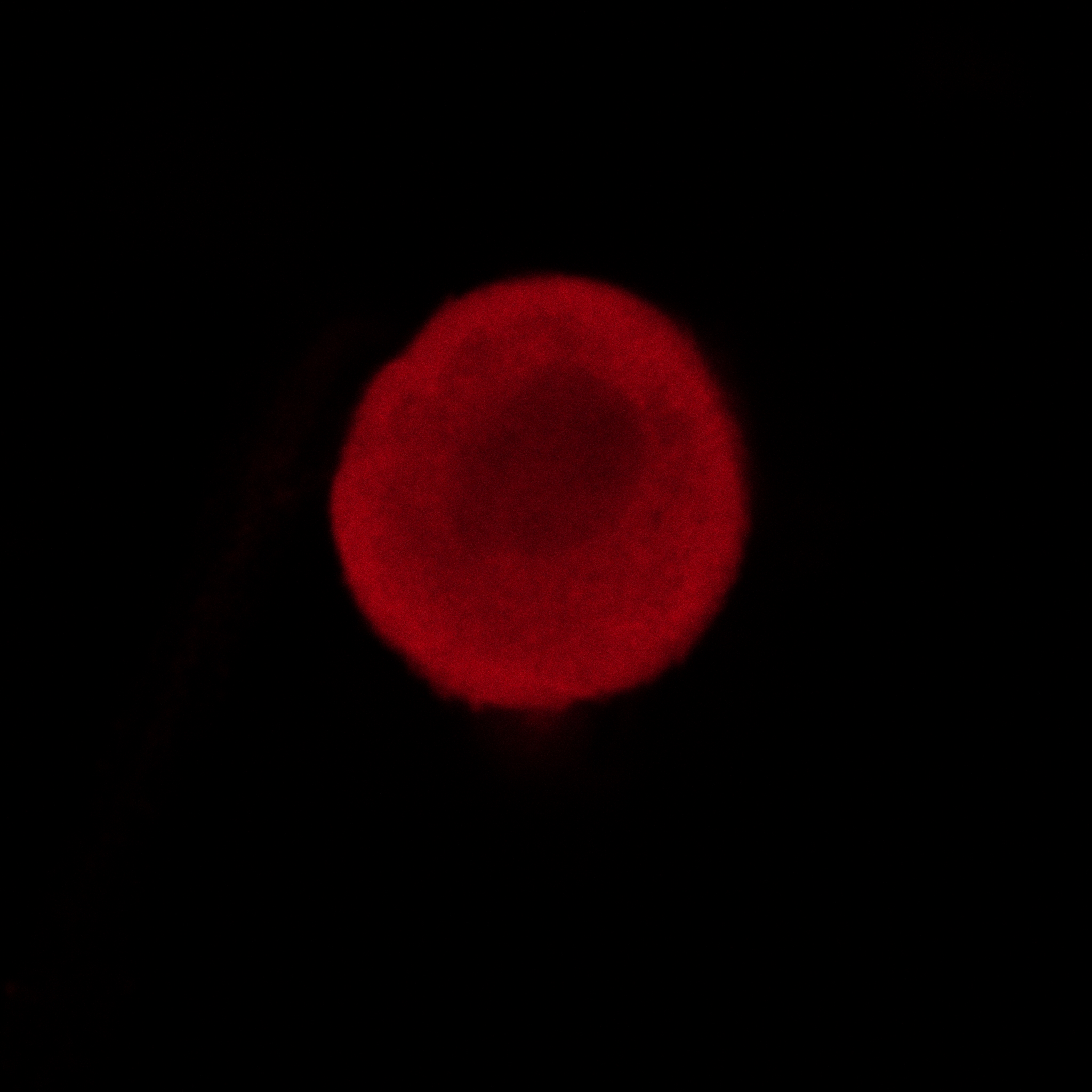

Supplement: Supplementary file 10 — Source data Fig. 8 [file 44318_2025_487_MOESM10_ESM.zip › Figure 8/8C ionomycin/TRPV1.jpg]

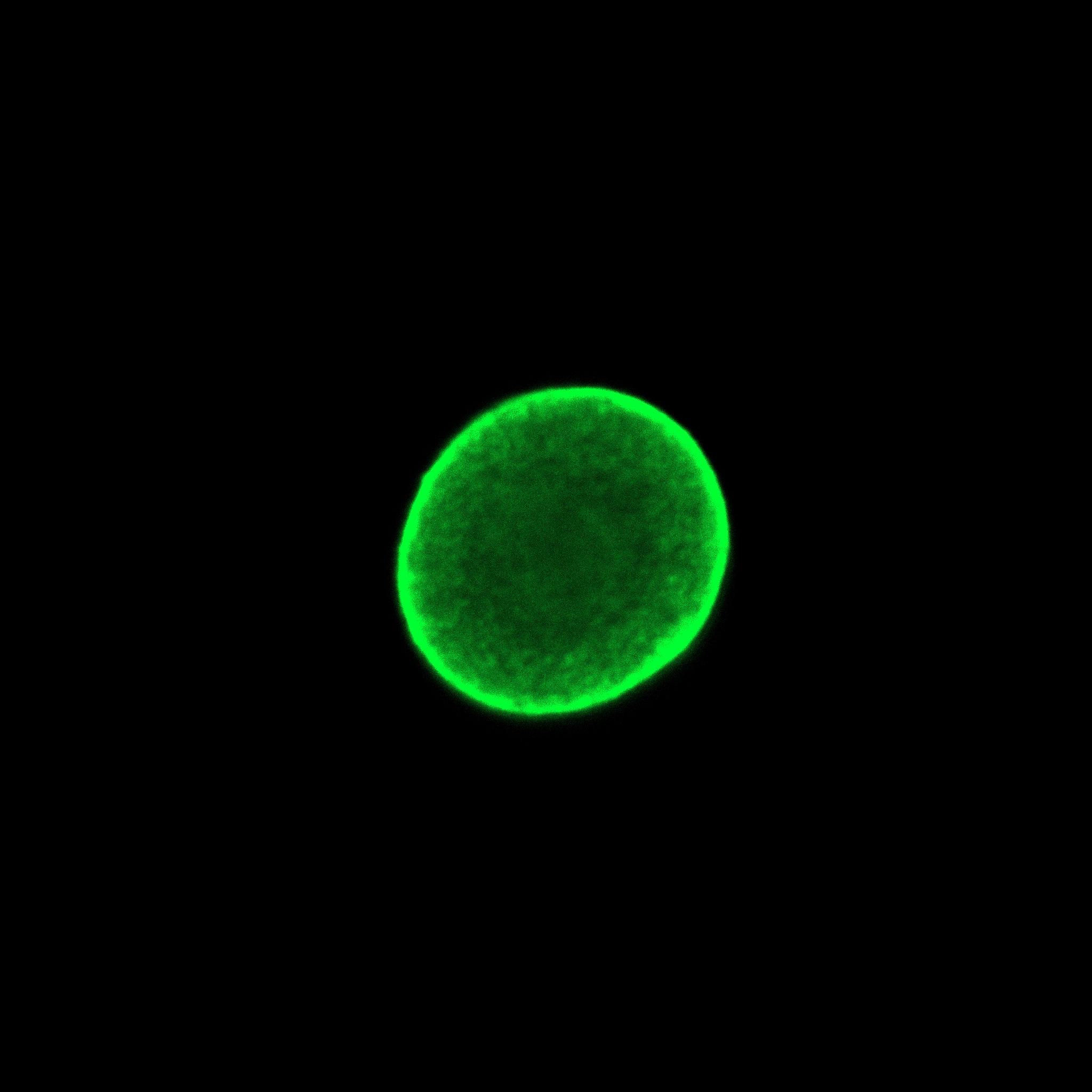

Supplement: Supplementary file 10 — Source data Fig. 8 [file 44318_2025_487_MOESM10_ESM.zip › Figure 8/8D 45/Copine-6.jpg]

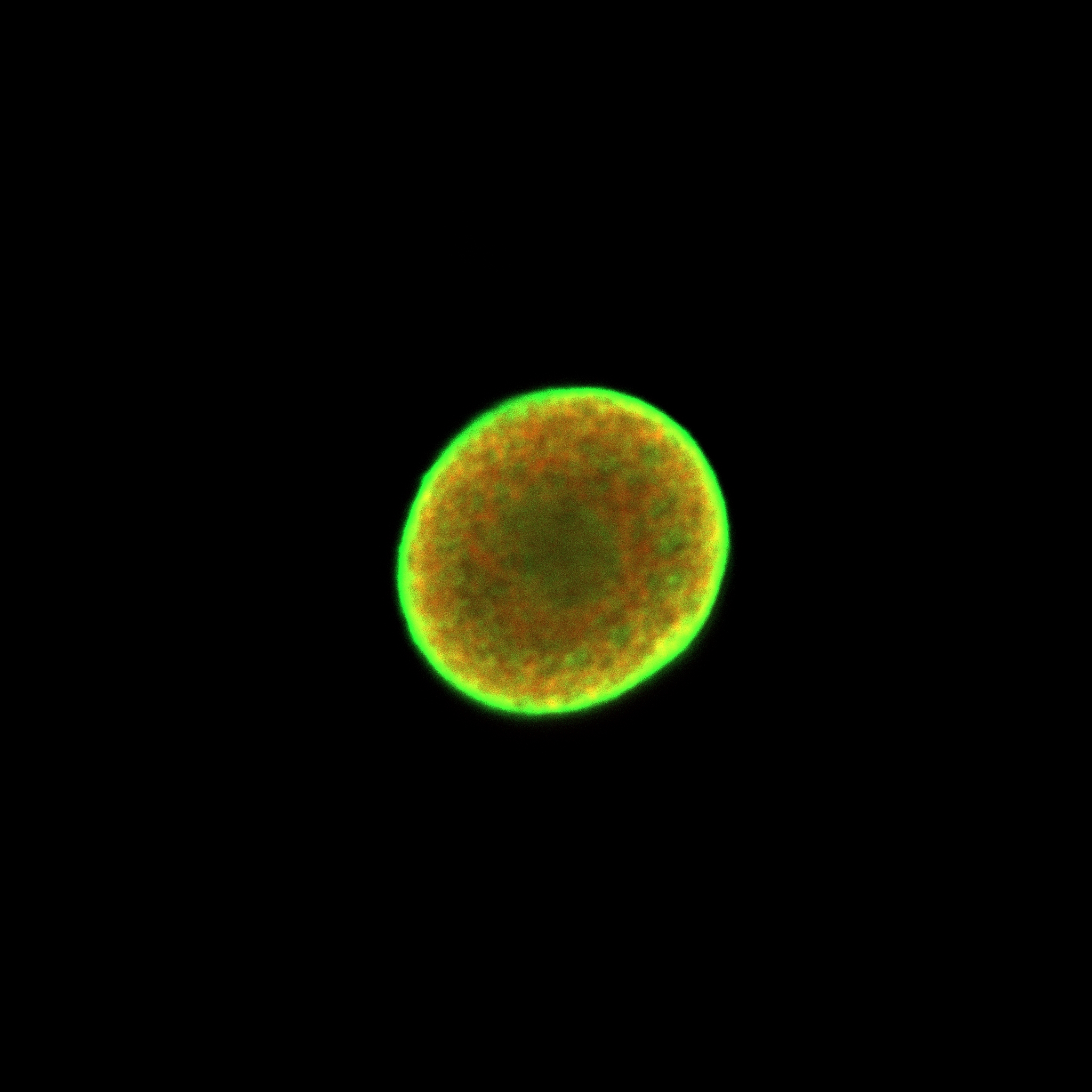

Supplement: Supplementary file 10 — Source data Fig. 8 [file 44318_2025_487_MOESM10_ESM.zip › Figure 8/8D 45/merge.jpg]

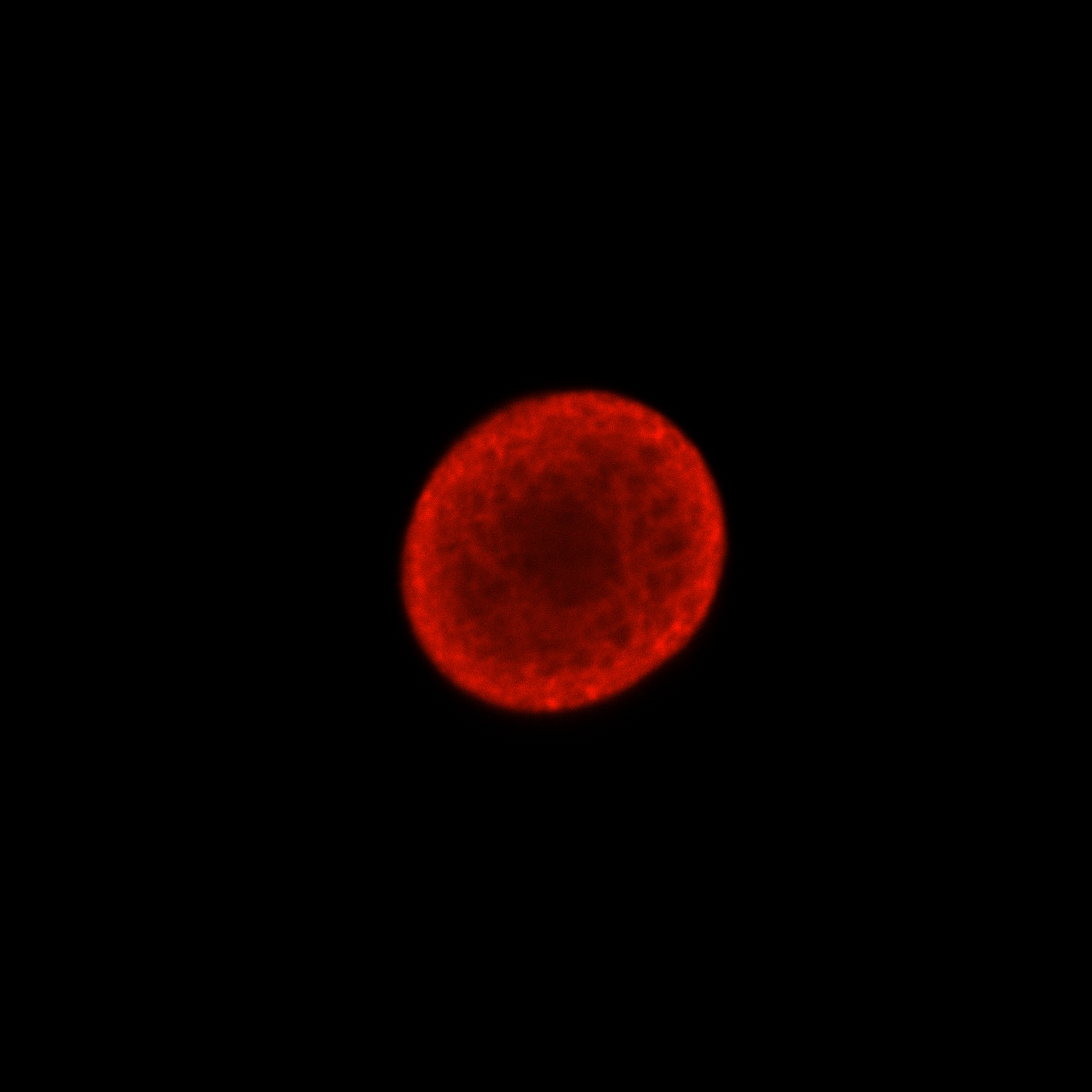

Supplement: Supplementary file 10 — Source data Fig. 8 [file 44318_2025_487_MOESM10_ESM.zip › Figure 8/8D 45/TRPA1.jpg]

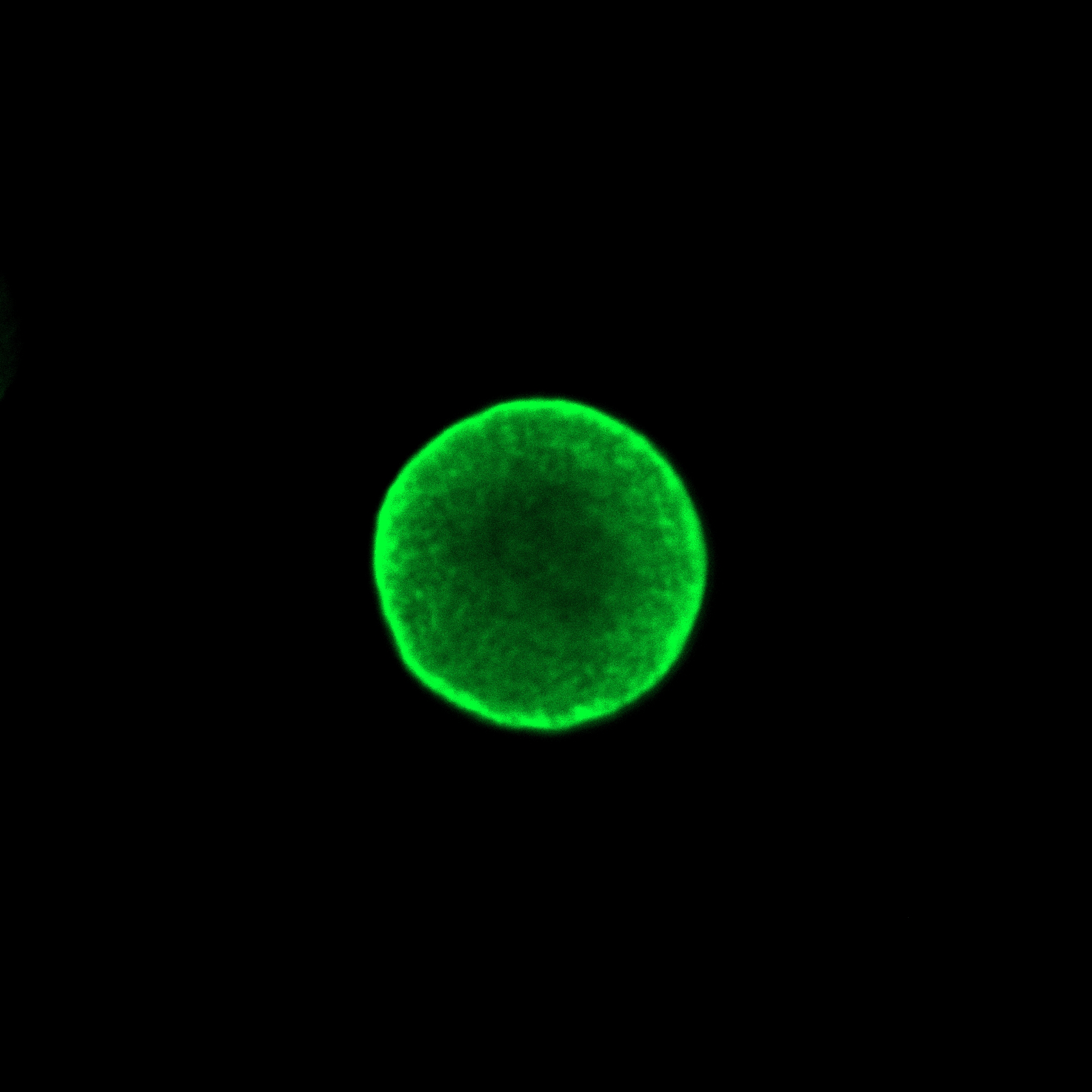

Supplement: Supplementary file 10 — Source data Fig. 8 [file 44318_2025_487_MOESM10_ESM.zip › Figure 8/8D AITC/Copine-6.jpg]

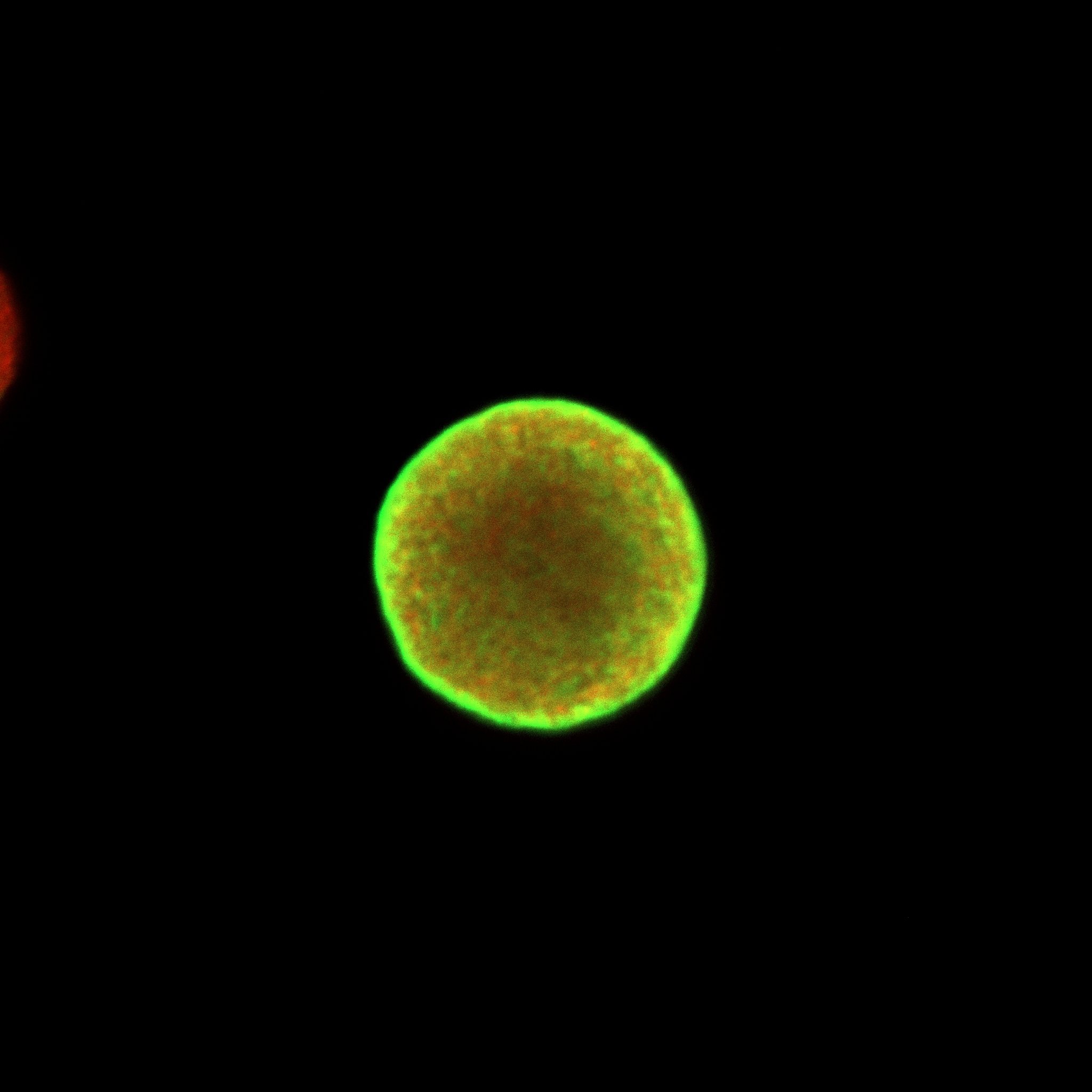

Supplement: Supplementary file 10 — Source data Fig. 8 [file 44318_2025_487_MOESM10_ESM.zip › Figure 8/8D AITC/merge.jpg]

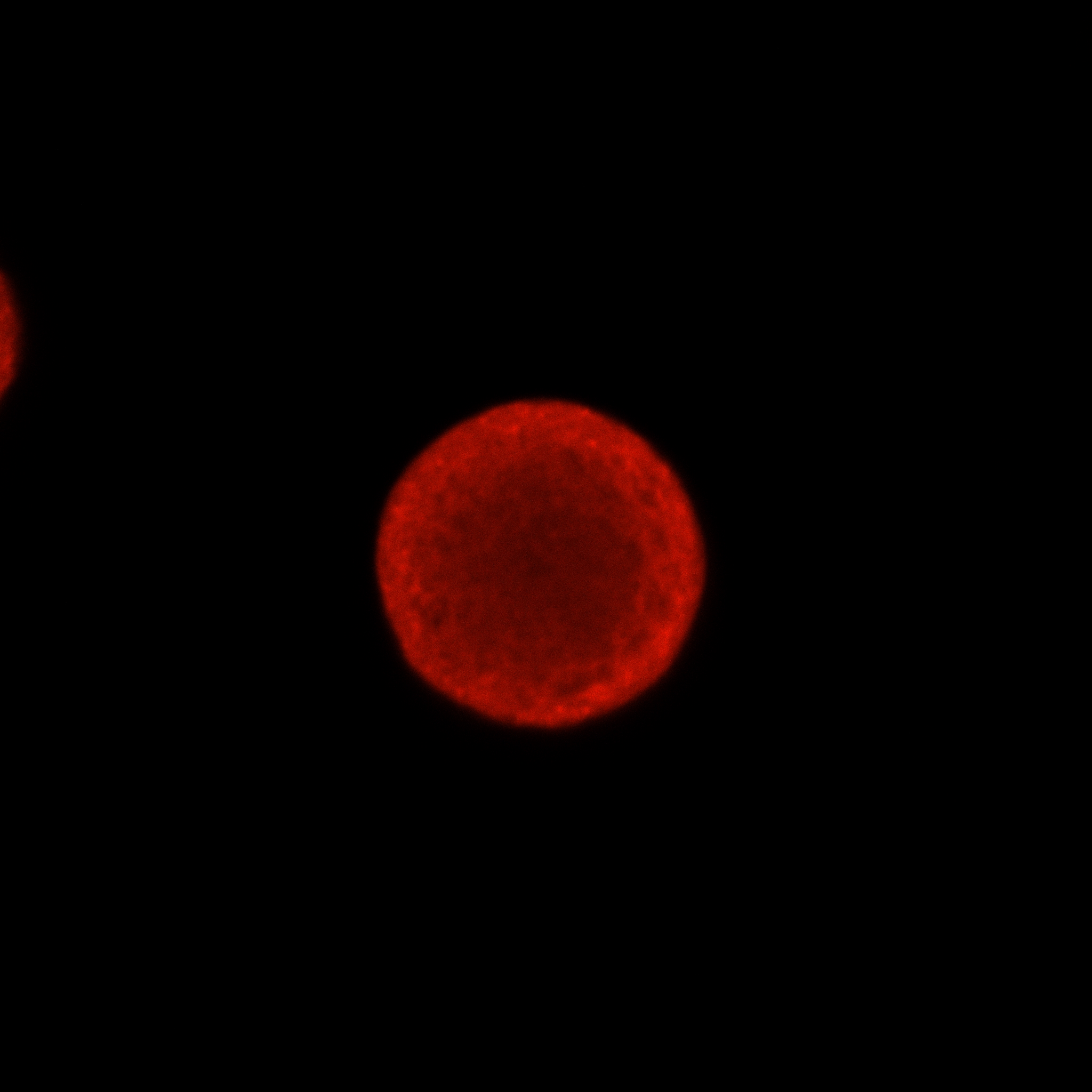

Supplement: Supplementary file 10 — Source data Fig. 8 [file 44318_2025_487_MOESM10_ESM.zip › Figure 8/8D AITC/TRPA1.jpg]

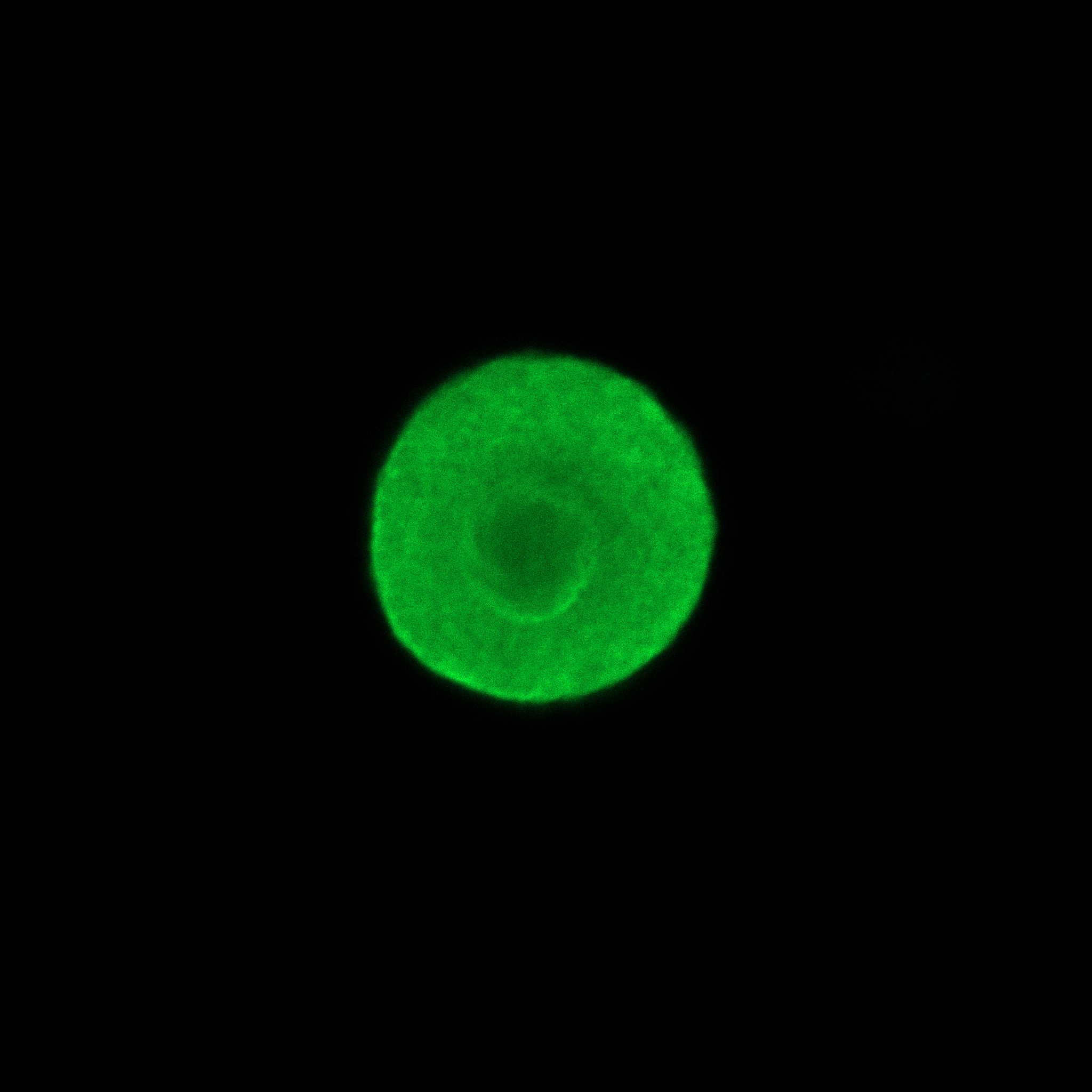

Supplement: Supplementary file 10 — Source data Fig. 8 [file 44318_2025_487_MOESM10_ESM.zip › Figure 8/8D control/Copine-6.jpg]

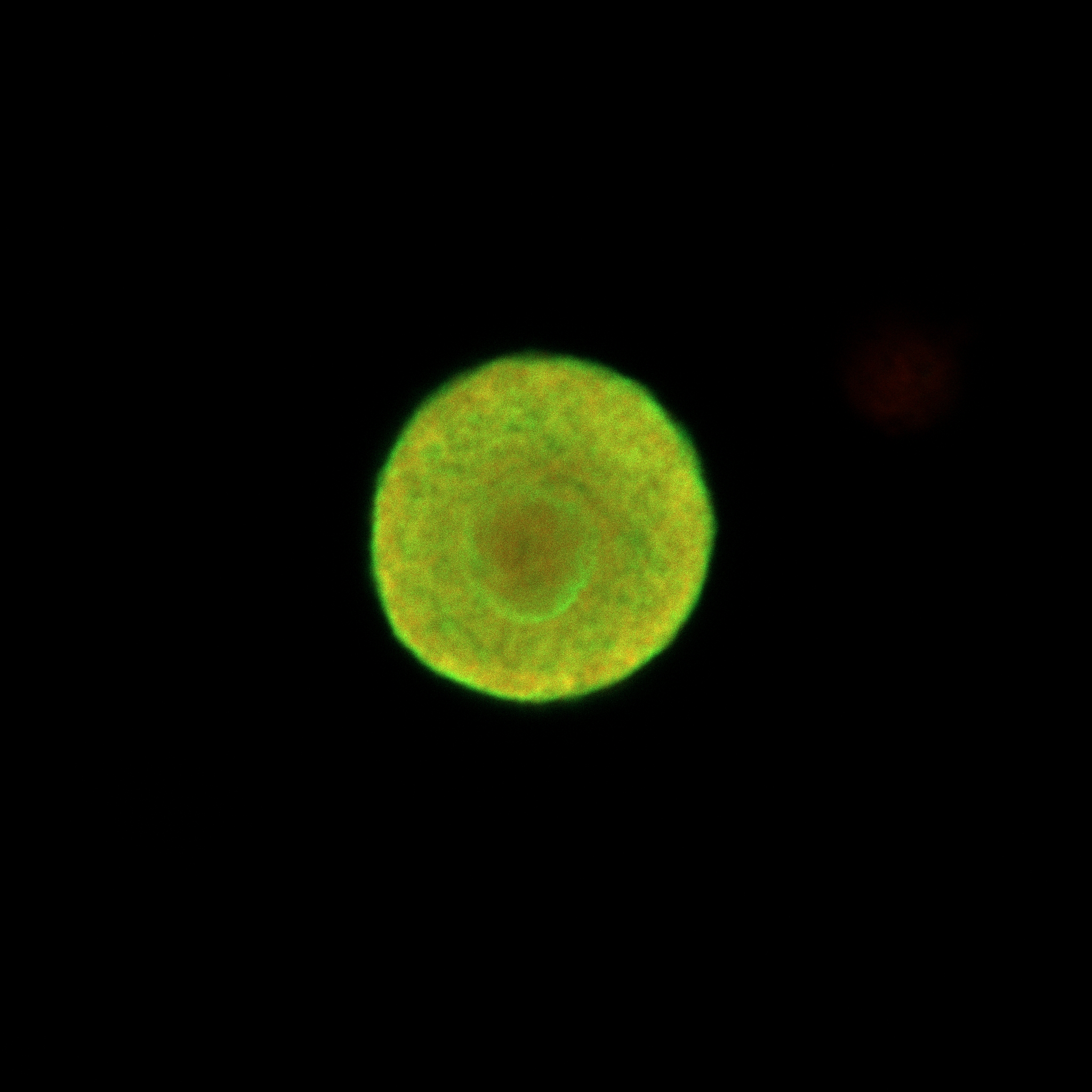

Supplement: Supplementary file 10 — Source data Fig. 8 [file 44318_2025_487_MOESM10_ESM.zip › Figure 8/8D control/merge.jpg]

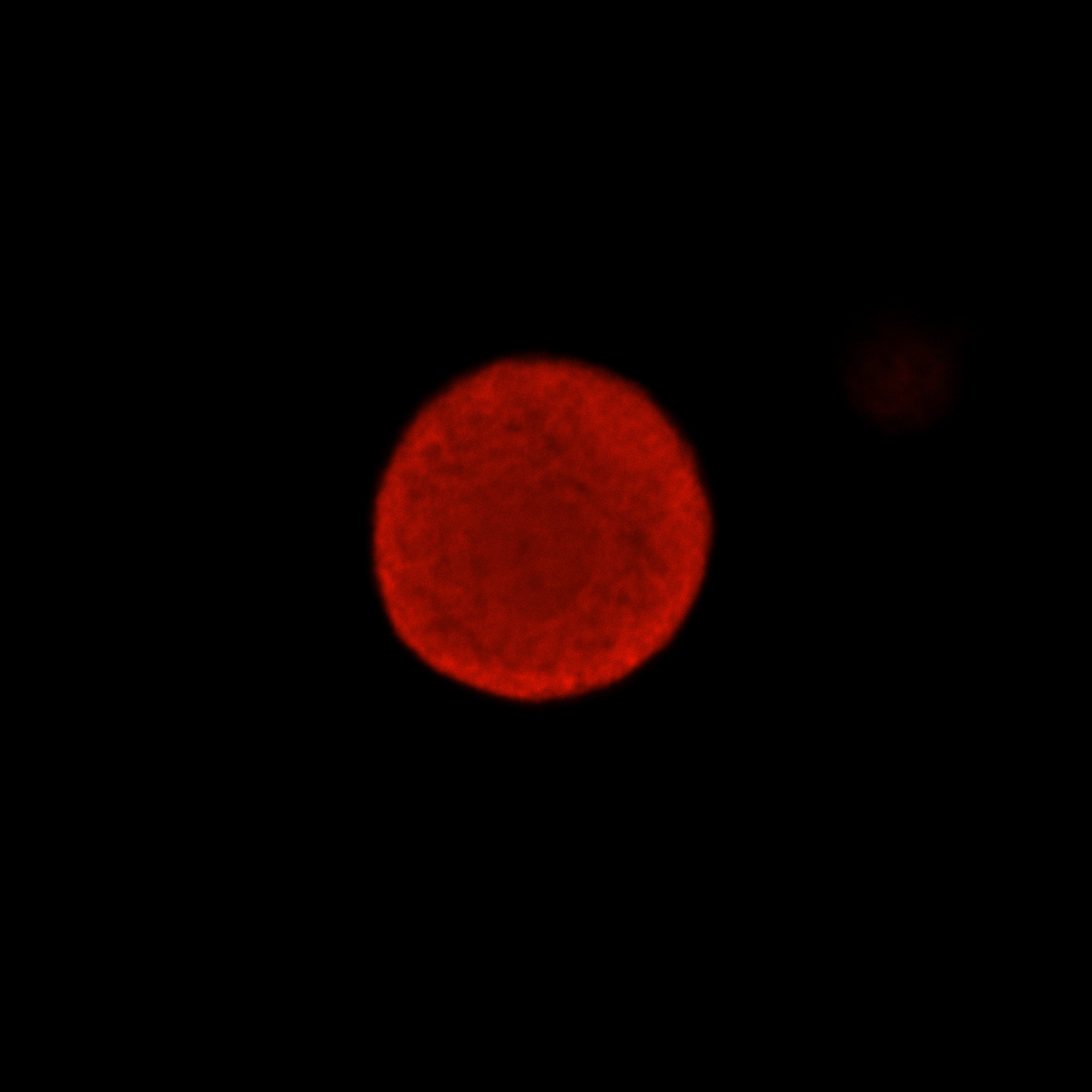

Supplement: Supplementary file 10 — Source data Fig. 8 [file 44318_2025_487_MOESM10_ESM.zip › Figure 8/8D control/TRPA1.jpg]

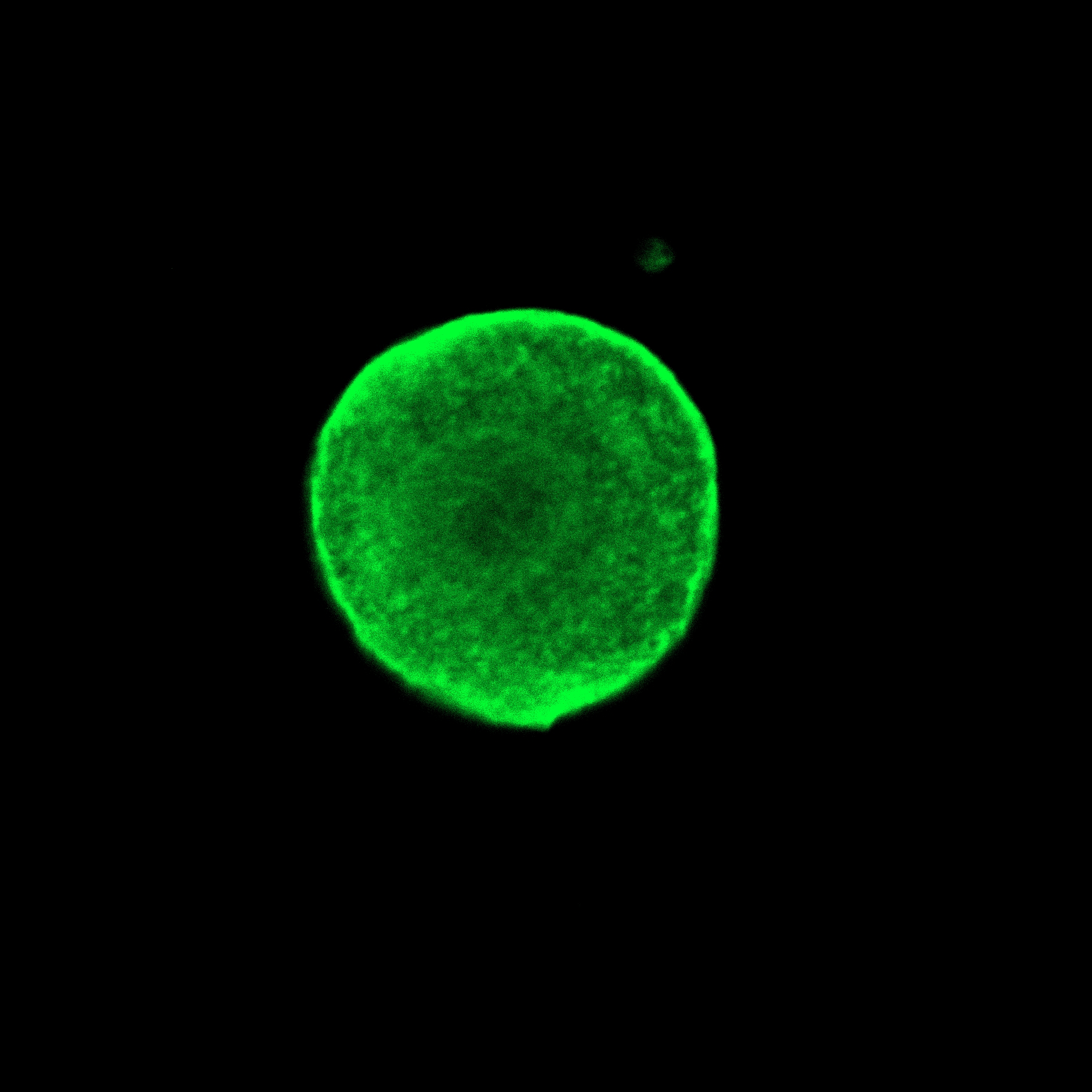

Supplement: Supplementary file 10 — Source data Fig. 8 [file 44318_2025_487_MOESM10_ESM.zip › Figure 8/8D ionomycin/Copine-6.jpg]

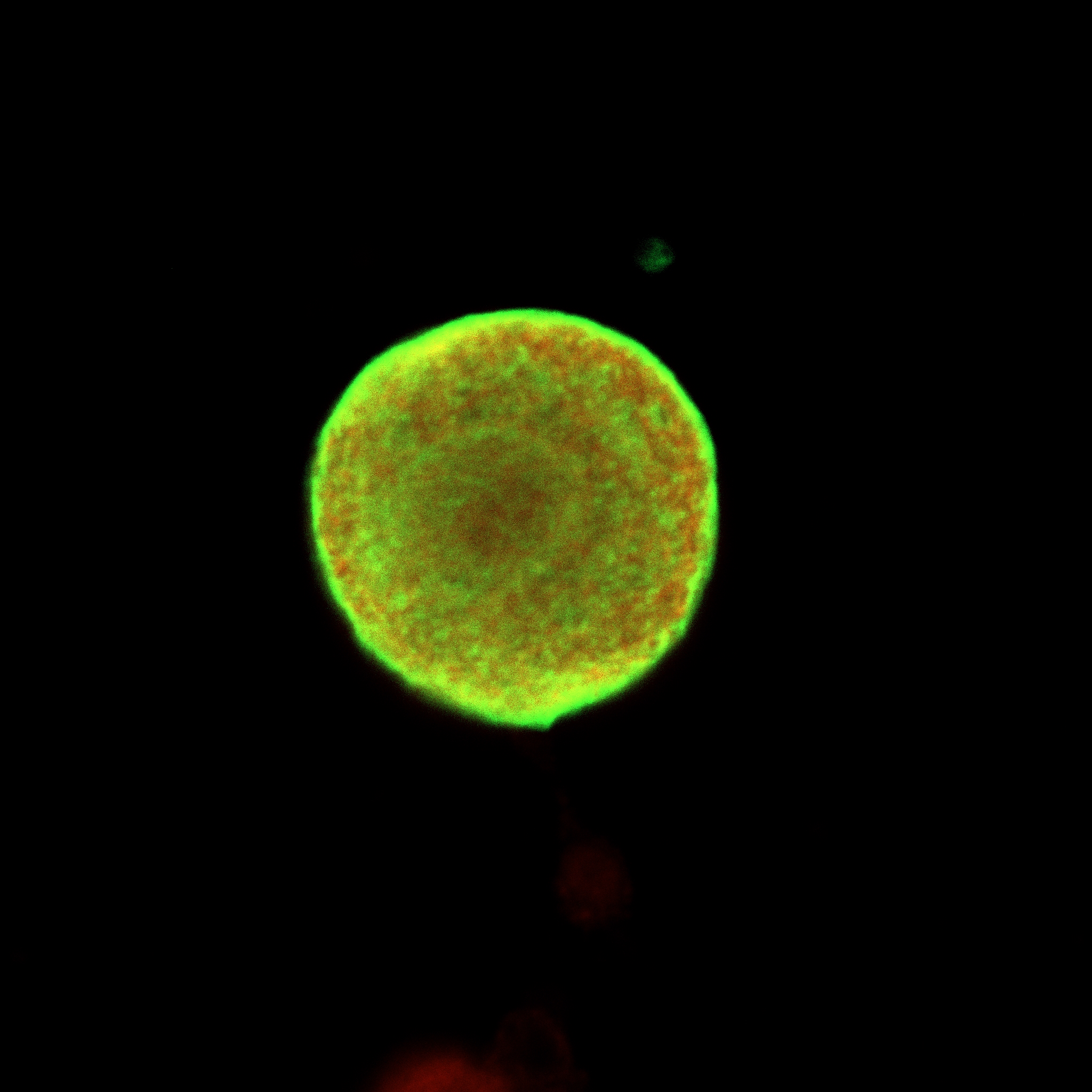

Supplement: Supplementary file 10 — Source data Fig. 8 [file 44318_2025_487_MOESM10_ESM.zip › Figure 8/8D ionomycin/merge.jpg]

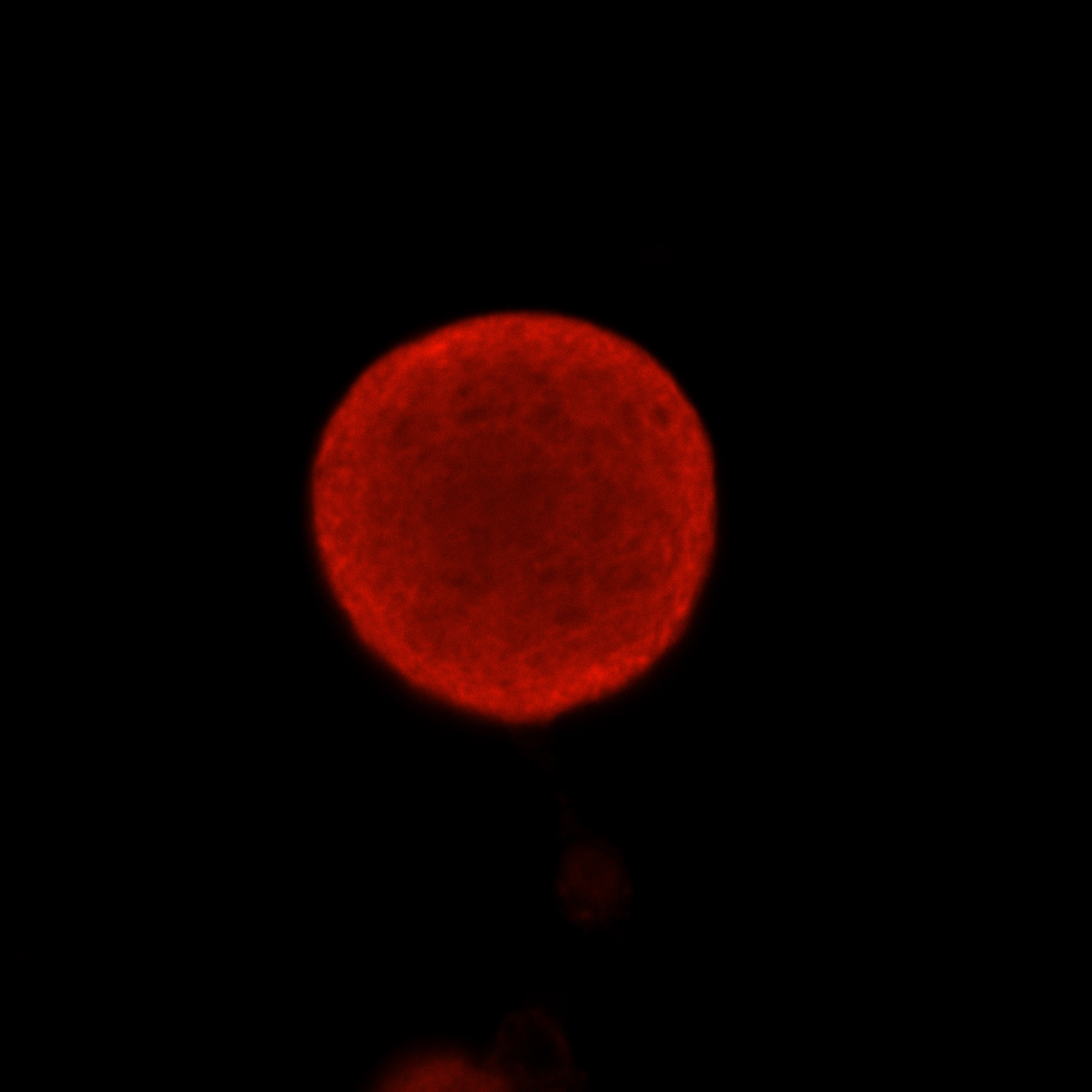

Supplement: Supplementary file 10 — Source data Fig. 8 [file 44318_2025_487_MOESM10_ESM.zip › Figure 8/8D ionomycin/TRPA1.jpg]

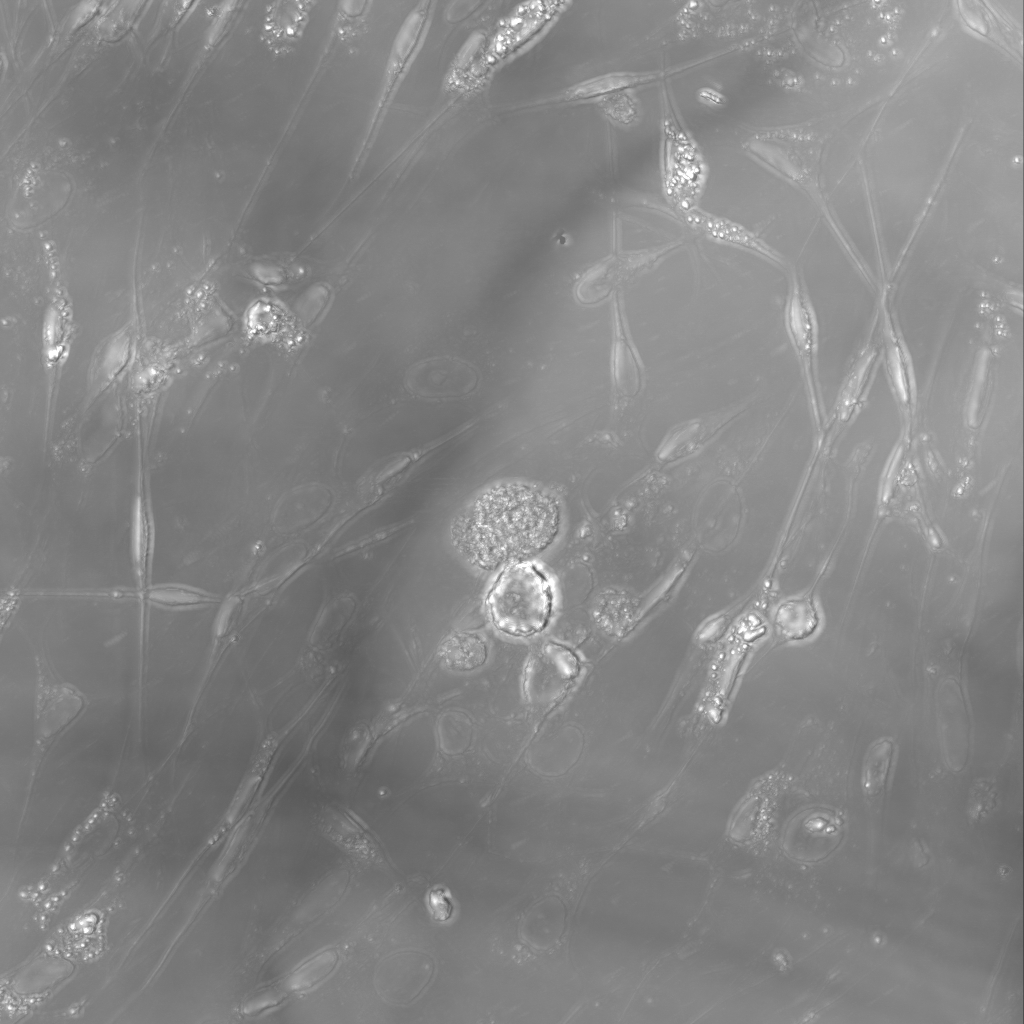

Supplement: Supplementary file 11 — Source data Fig. 9 [file 44318_2025_487_MOESM11_ESM.zip › Figure 9/9B/Copine6 TRPM3/BF.jpg]

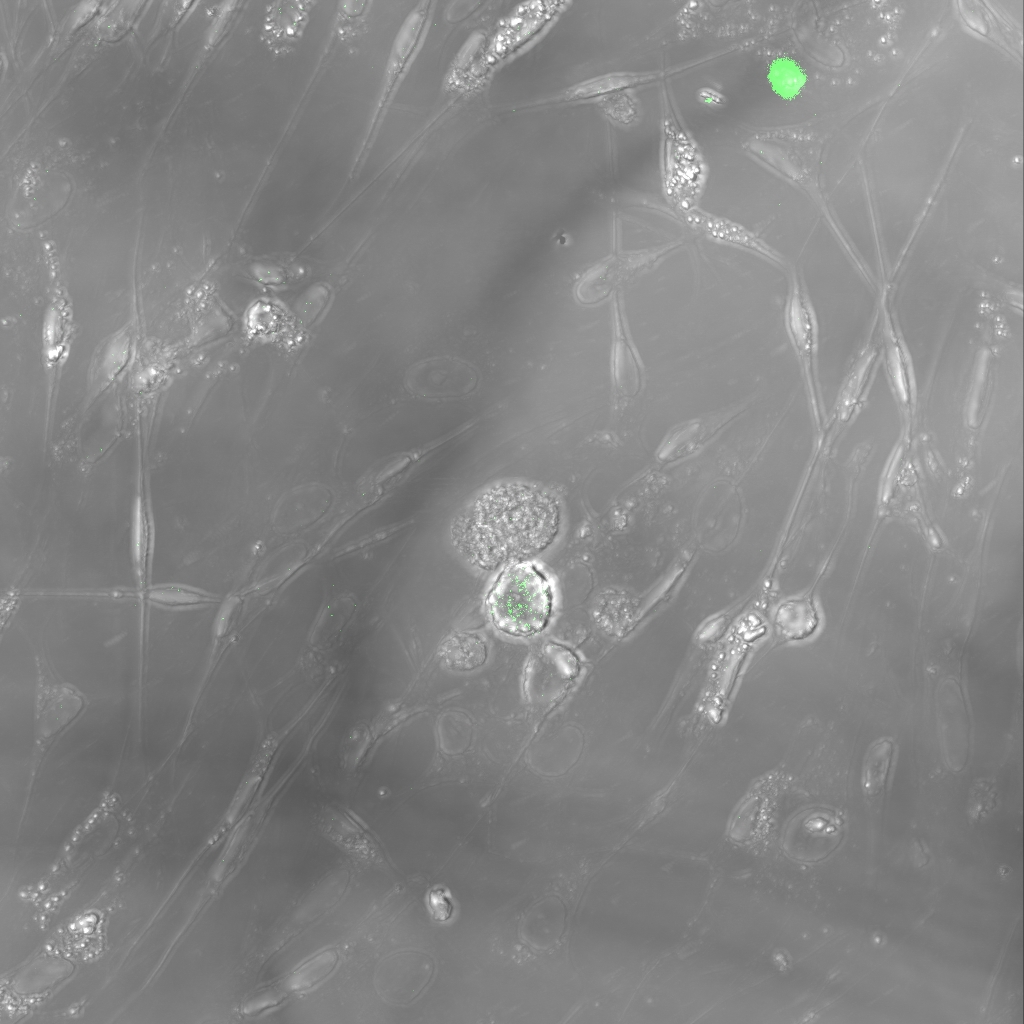

Supplement: Supplementary file 11 — Source data Fig. 9 [file 44318_2025_487_MOESM11_ESM.zip › Figure 9/9B/Copine6 TRPM3/merge.jpg]

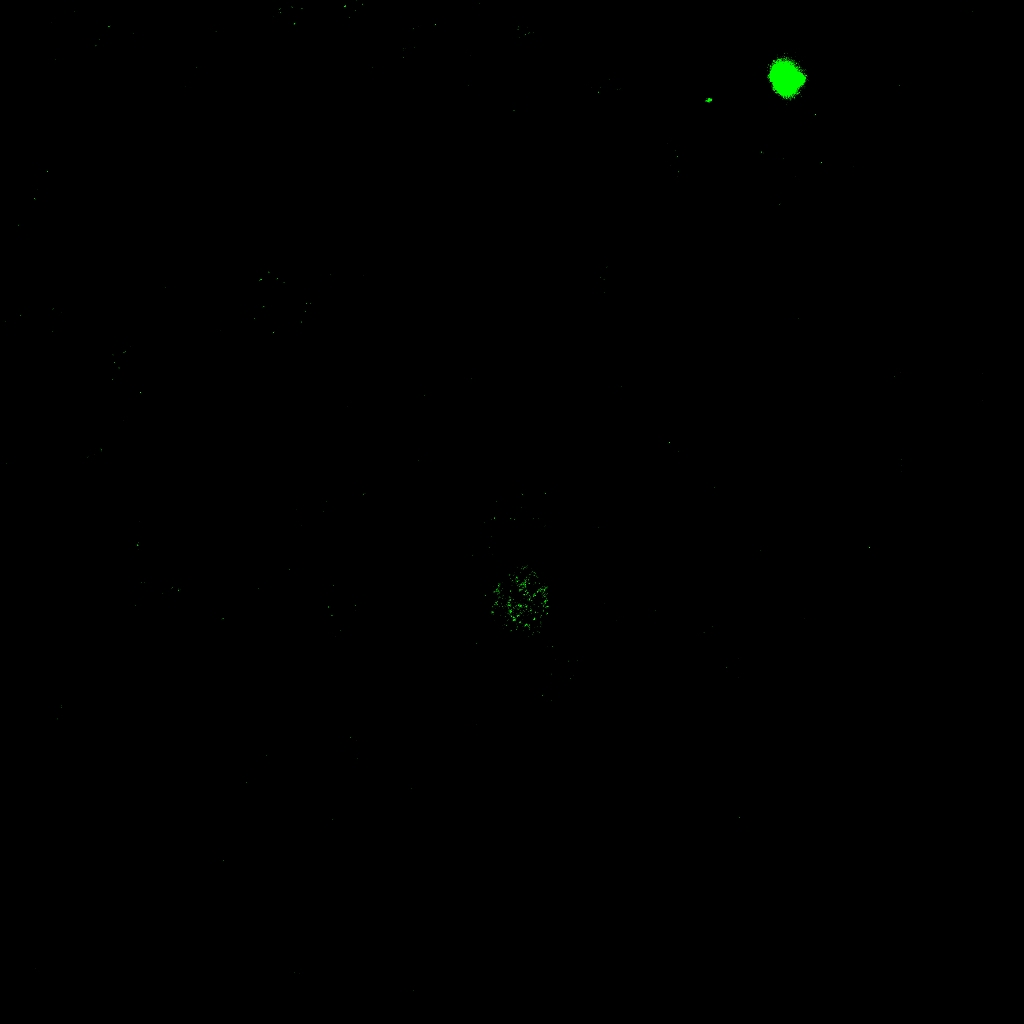

Supplement: Supplementary file 11 — Source data Fig. 9 [file 44318_2025_487_MOESM11_ESM.zip › Figure 9/9B/Copine6 TRPM3/PLA.jpg]

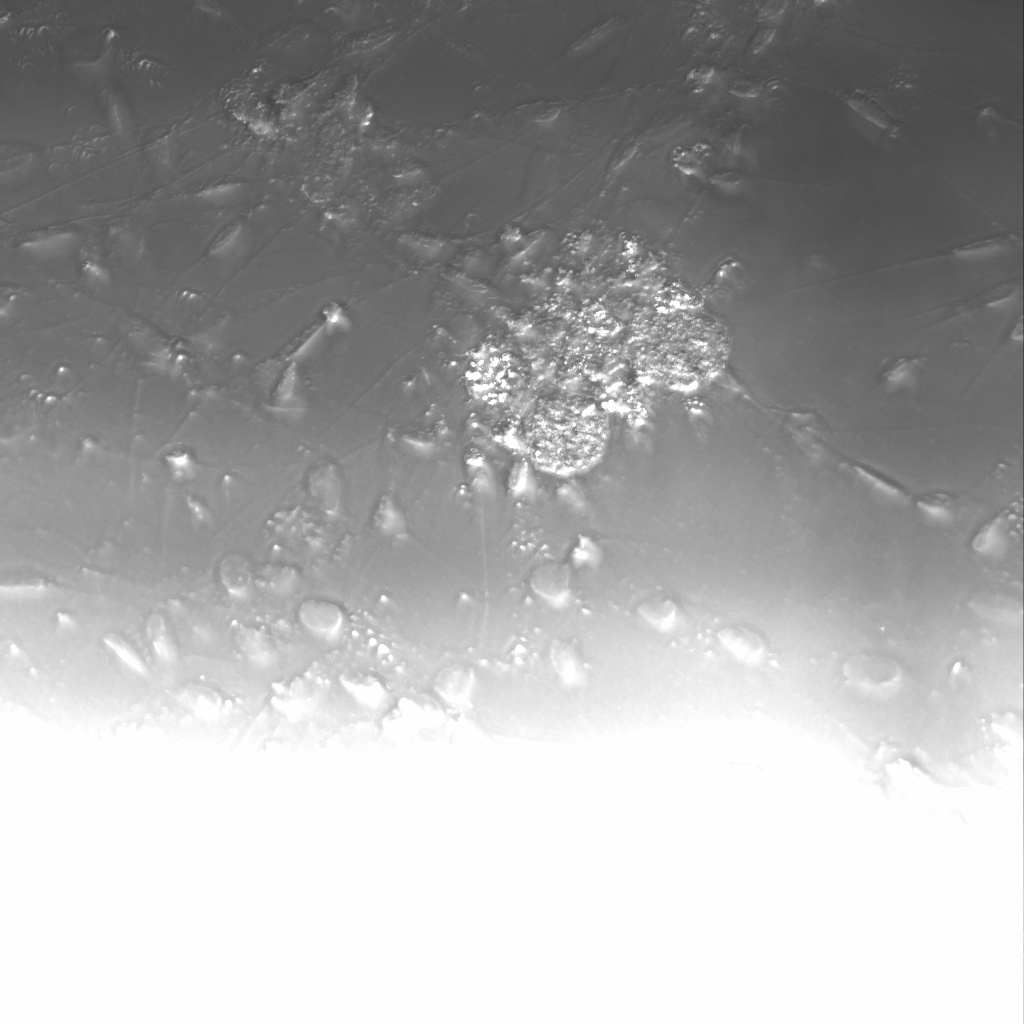

Supplement: Supplementary file 11 — Source data Fig. 9 [file 44318_2025_487_MOESM11_ESM.zip › Figure 9/9B/Copine6 TRPV1/BF.jpg]

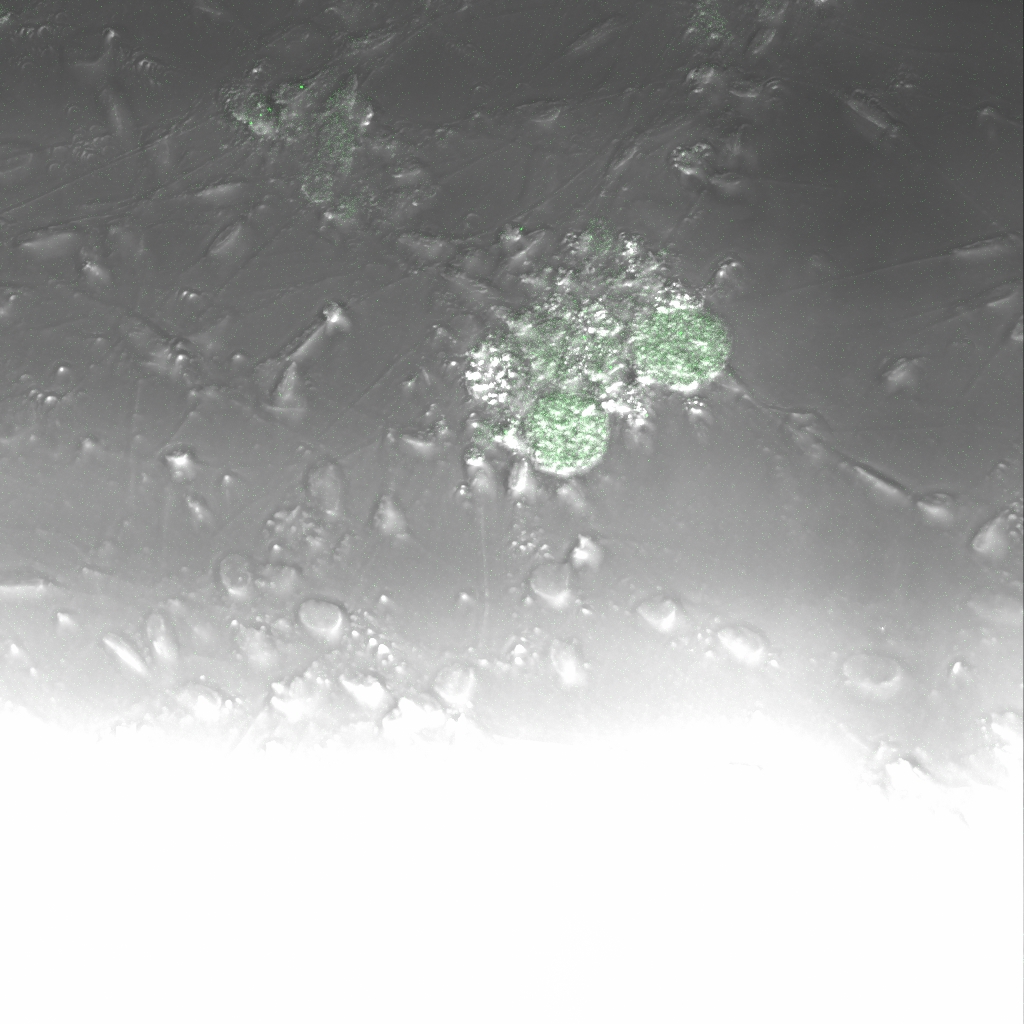

Supplement: Supplementary file 11 — Source data Fig. 9 [file 44318_2025_487_MOESM11_ESM.zip › Figure 9/9B/Copine6 TRPV1/merge.jpg]

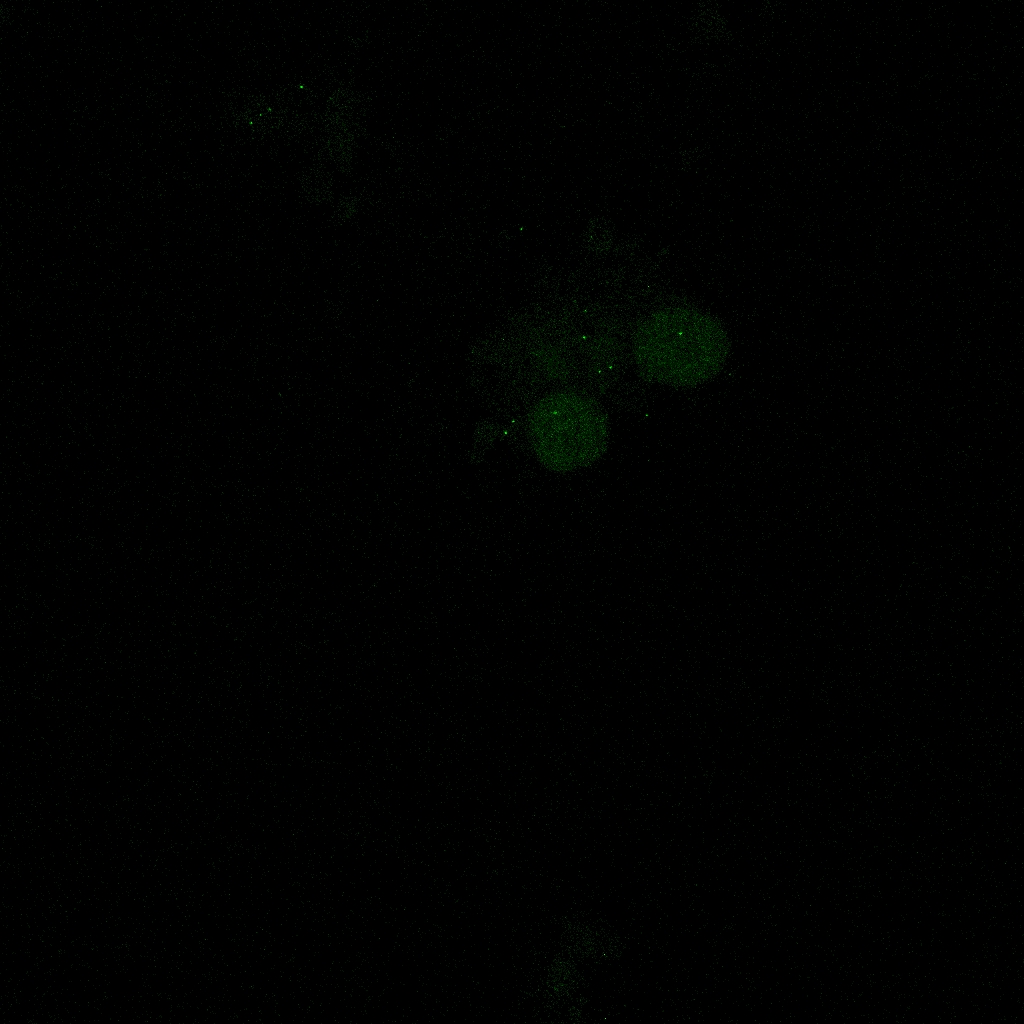

Supplement: Supplementary file 11 — Source data Fig. 9 [file 44318_2025_487_MOESM11_ESM.zip › Figure 9/9B/Copine6 TRPV1/PLA.jpg]

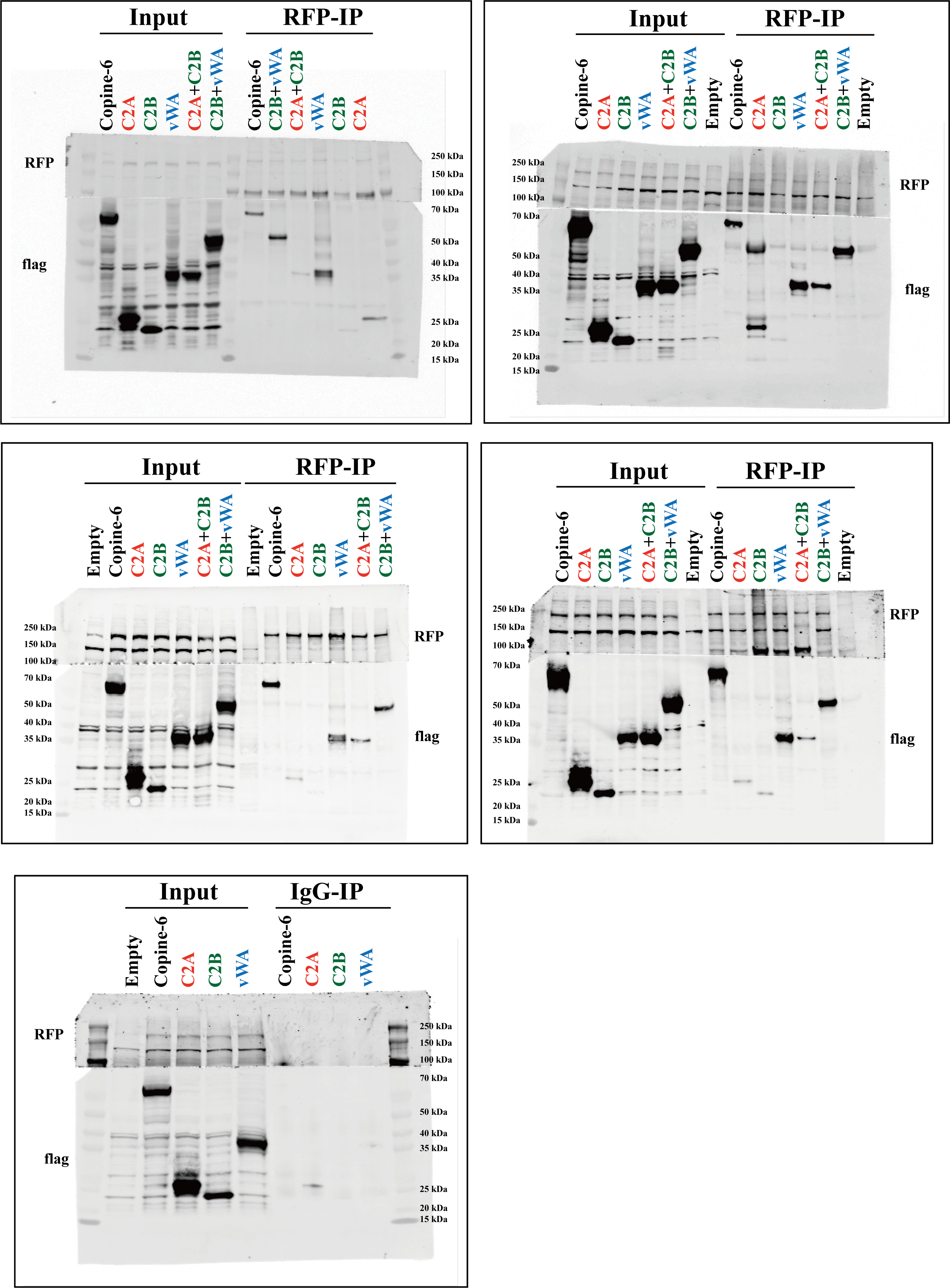

Supplement: Supplementary file 11 — Source data Fig. 9 [file 44318_2025_487_MOESM11_ESM.zip › Figure 9/9D.tif]

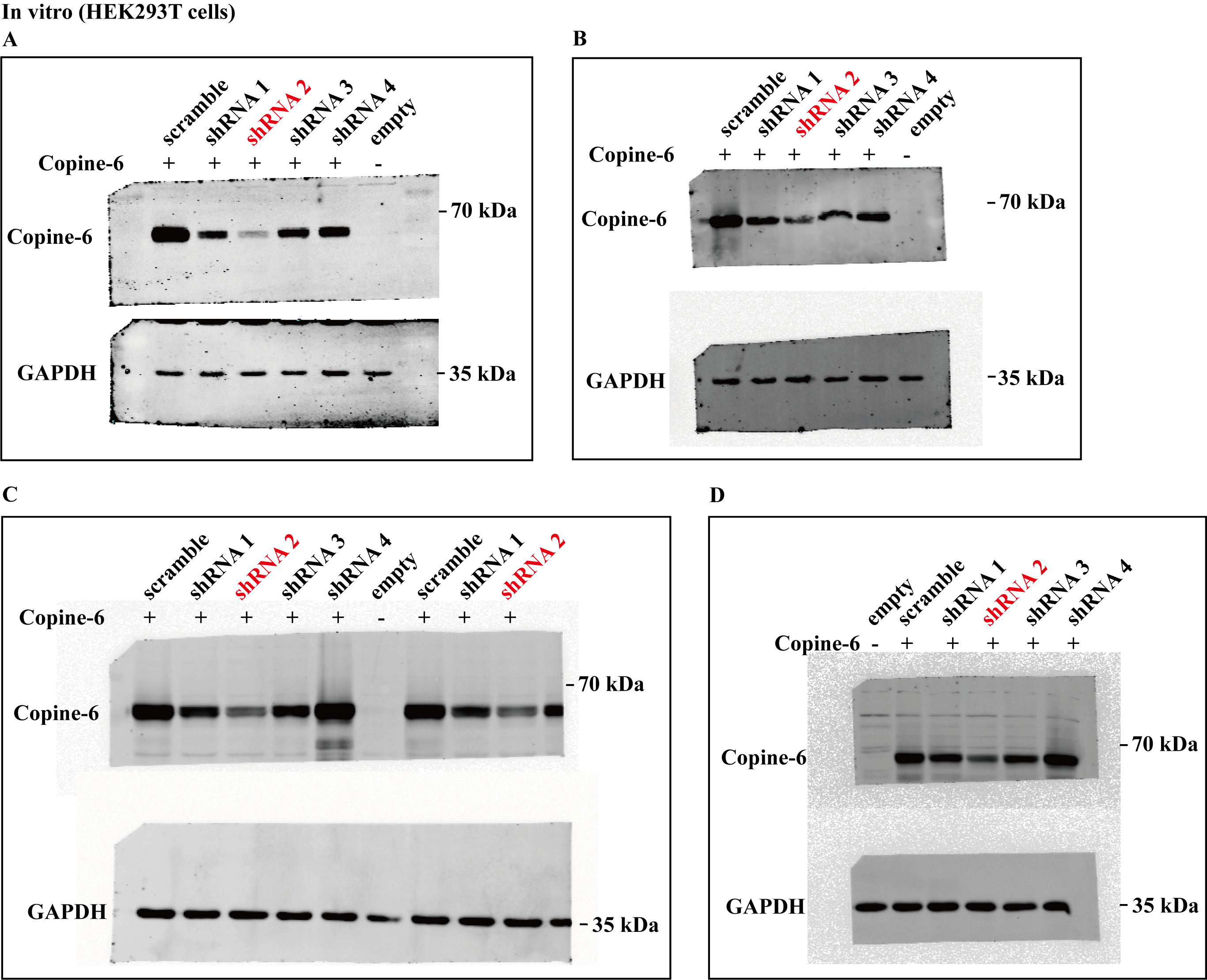

Supplement: Supplementary file 12 — EV Figure Source Data [file 44318_2025_487_MOESM12_ESM.zip › Figure EV1/1B.tif]

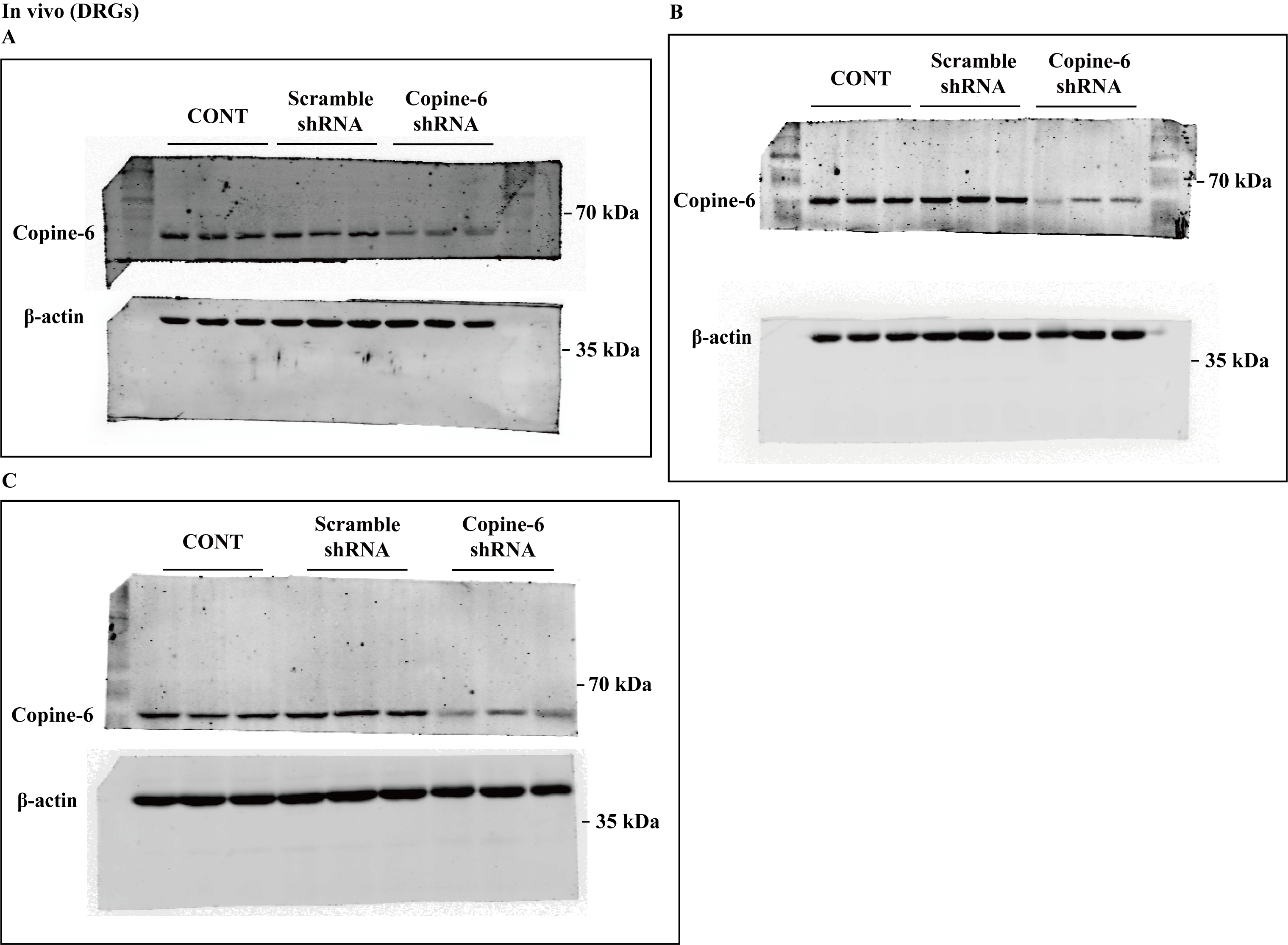

Supplement: Supplementary file 12 — EV Figure Source Data [file 44318_2025_487_MOESM12_ESM.zip › Figure EV1/1G.tif]

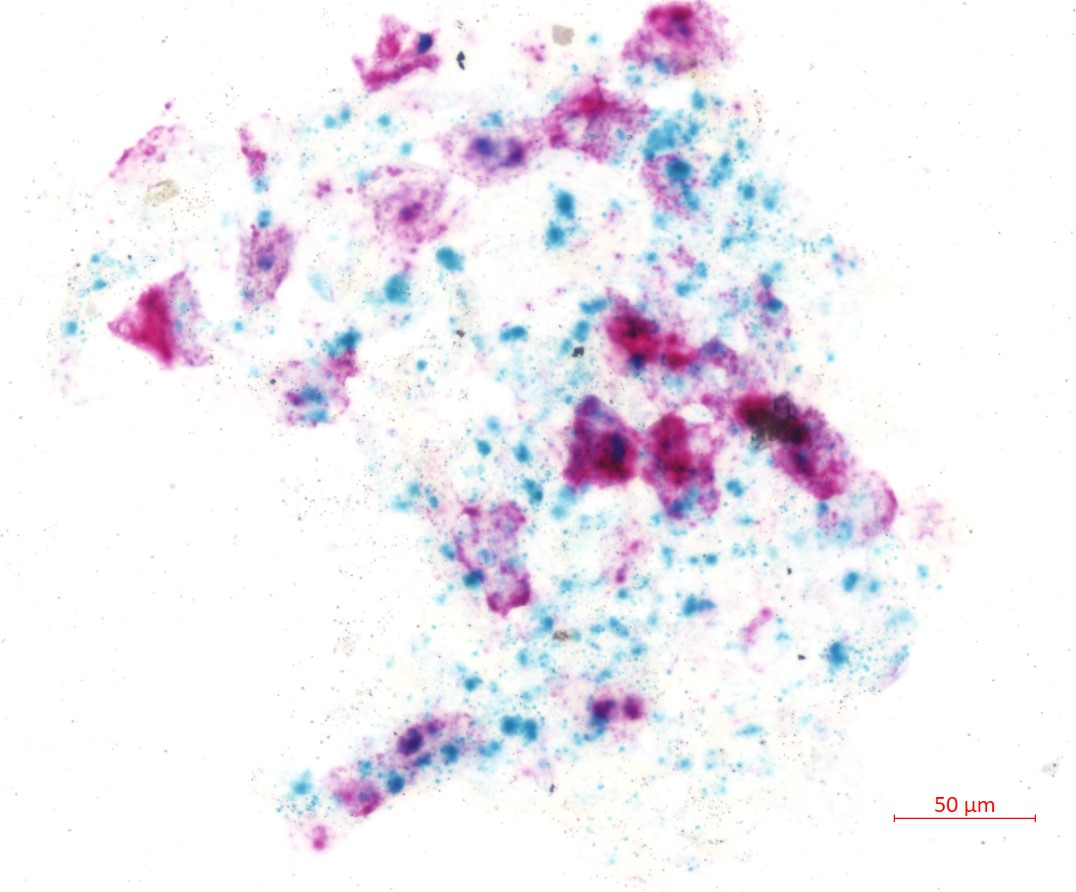

Supplement: Supplementary file 13 — Appendix Figure Source Data [file 44318_2025_487_MOESM13_ESM.zip › Appendix Figure S4/Example-1.jpg]

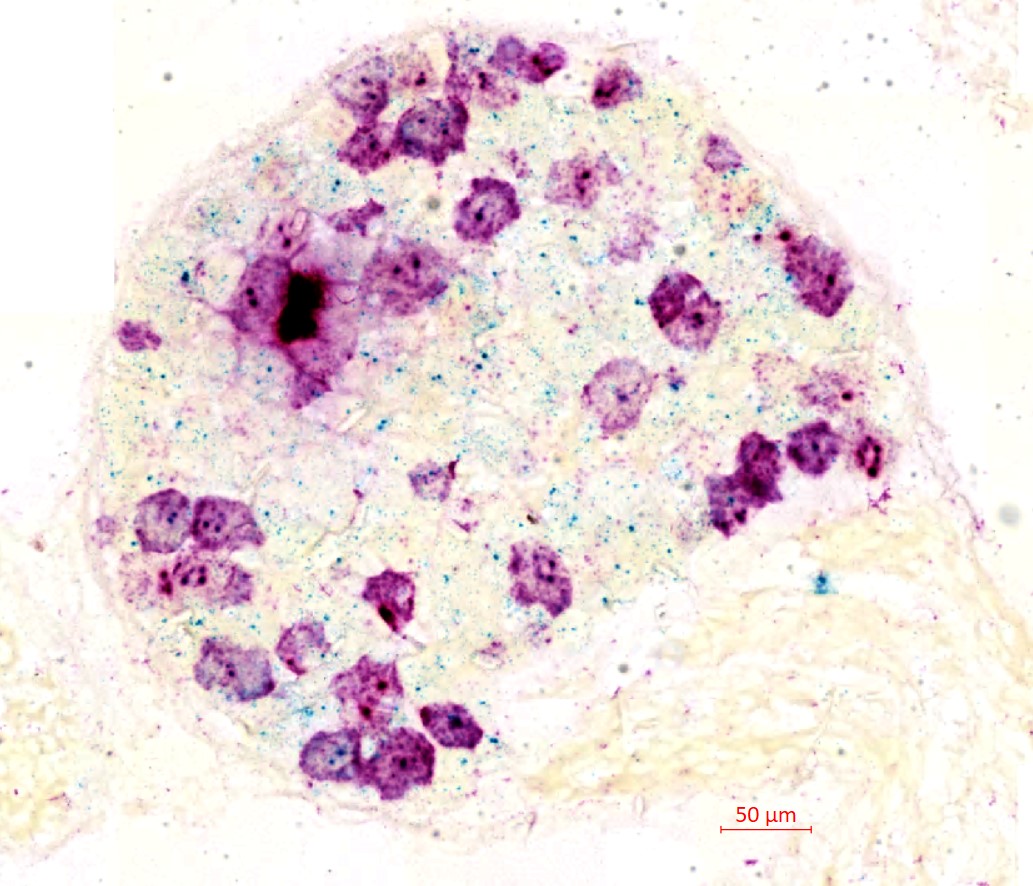

Supplement: Supplementary file 13 — Appendix Figure Source Data [file 44318_2025_487_MOESM13_ESM.zip › Appendix Figure S4/Example-2.jpg]

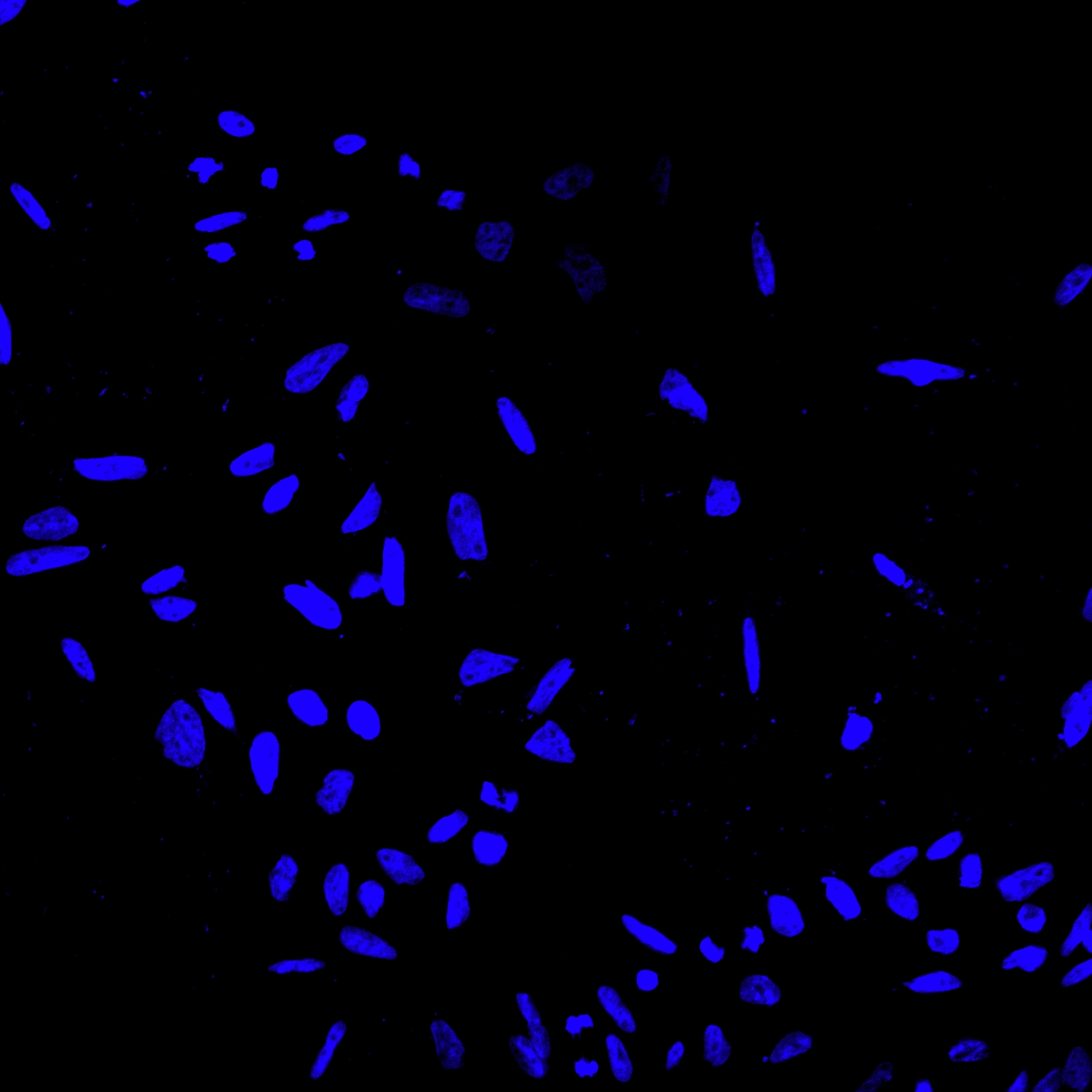

Supplement: Supplementary file 13 — Appendix Figure Source Data [file 44318_2025_487_MOESM13_ESM.zip › Appendix Figure S3/8A/20X/DAPI.jpg]

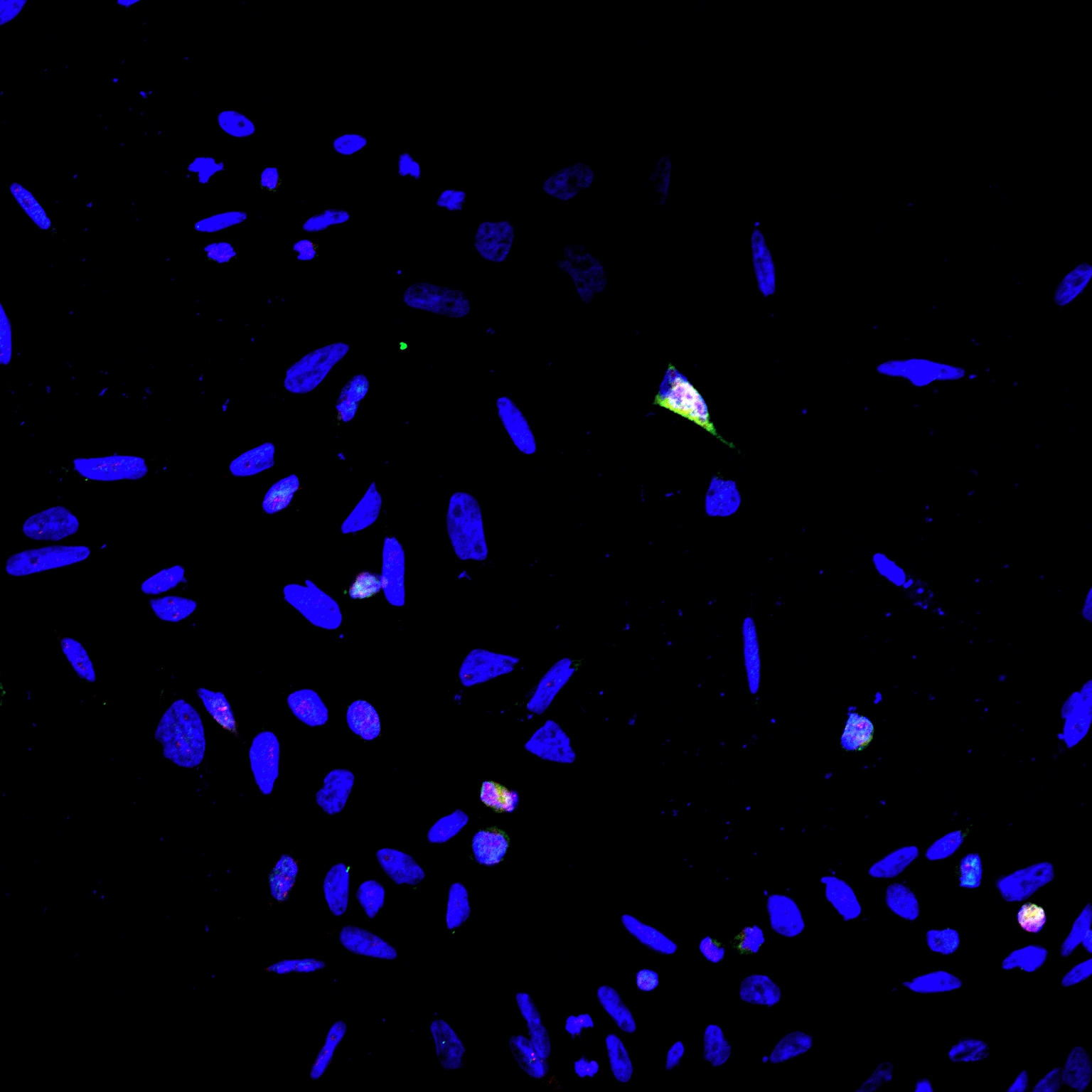

Supplement: Supplementary file 13 — Appendix Figure Source Data [file 44318_2025_487_MOESM13_ESM.zip › Appendix Figure S3/8A/20X/merge.jpg]
