# Supplementary figures and images for: Copine-6 is a TRPM3 escort protein controlling the sensitivity of sensory neurons to noxious heat (part 2 of 2)
Source: EMBO J. 2025 Jun 19;44(15):4222–51. doi: 10.1038/s44318-025-00487-0 (PMC12317139; doi:10.1038/s44318-025-00487-0)

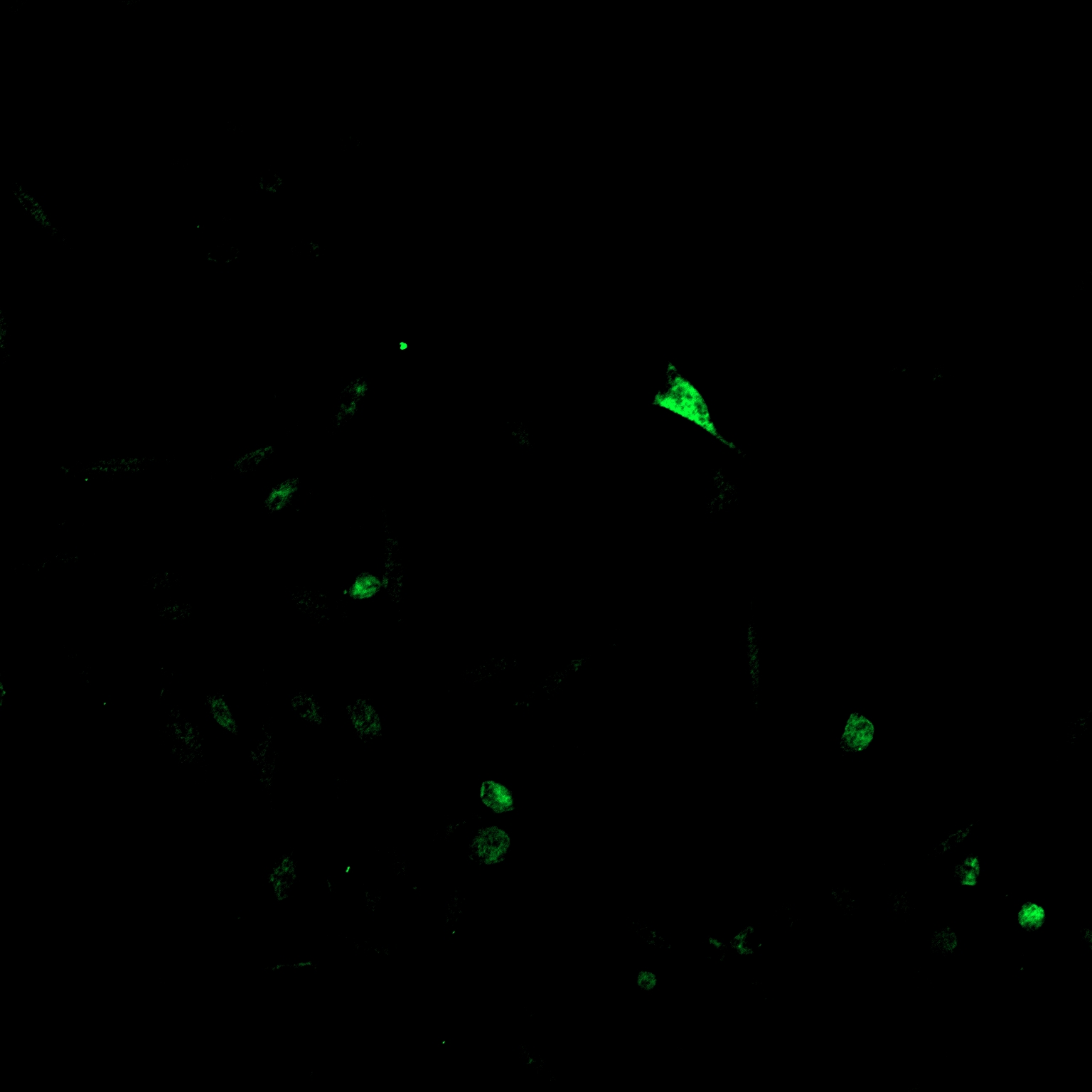

Supplement: Supplementary file 13 — Appendix Figure Source Data [file 44318_2025_487_MOESM13_ESM.zip › Appendix Figure S3/8A/20X/TRPM3-antibody.jpg]

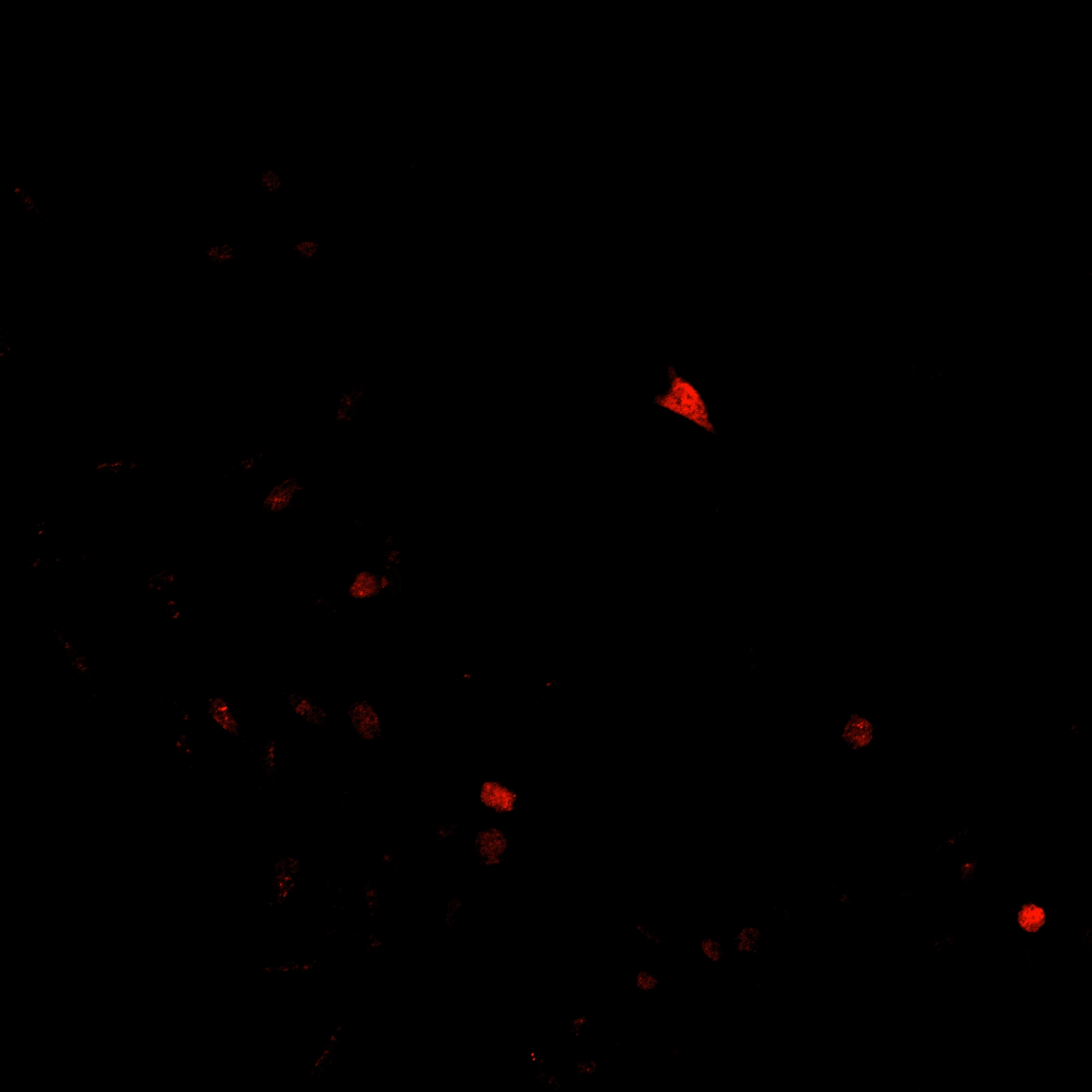

Supplement: Supplementary file 13 — Appendix Figure Source Data [file 44318_2025_487_MOESM13_ESM.zip › Appendix Figure S3/8A/20X/TRPM3-RFP.jpg]

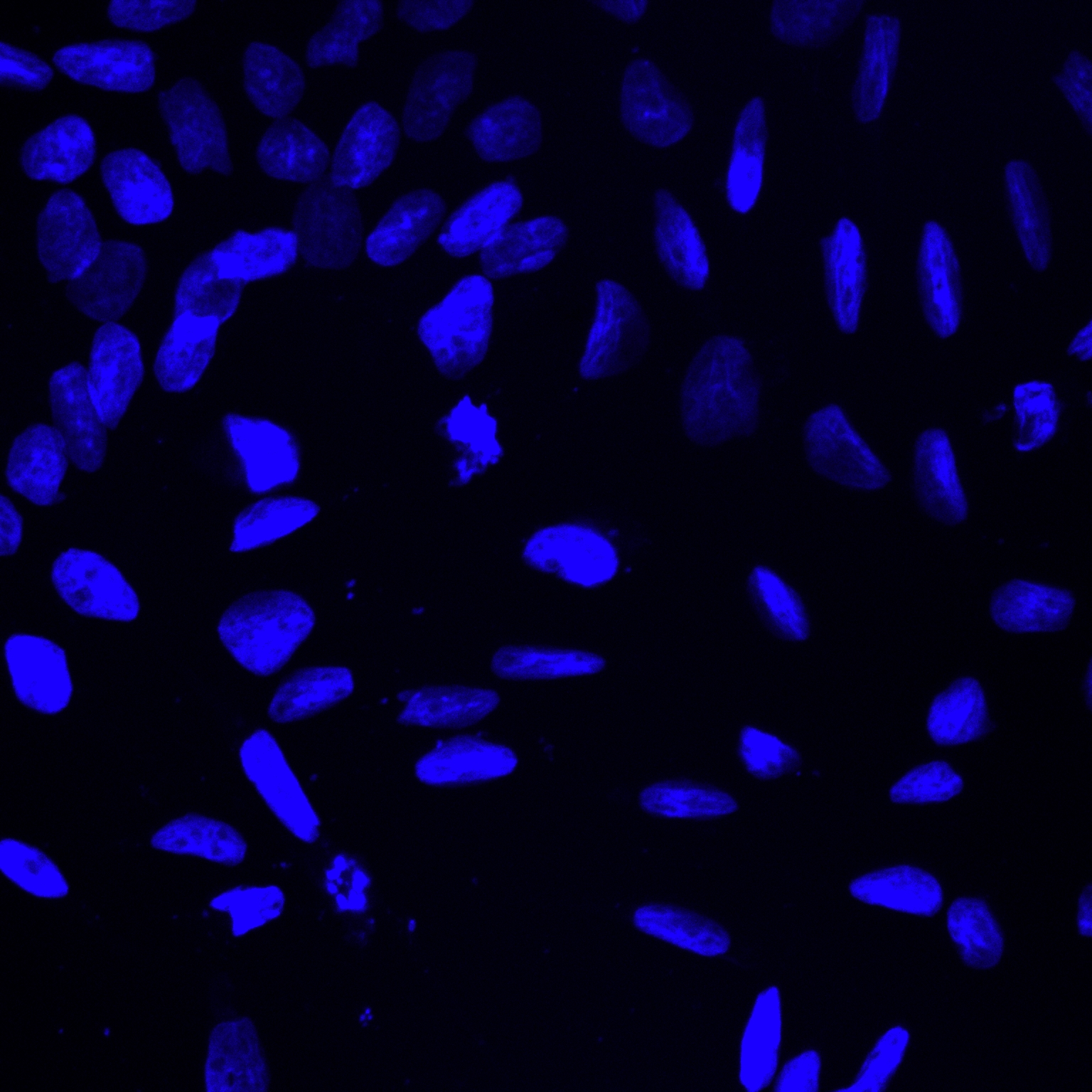

Supplement: Supplementary file 13 — Appendix Figure Source Data [file 44318_2025_487_MOESM13_ESM.zip › Appendix Figure S3/8A/40X/DAPI.jpg]

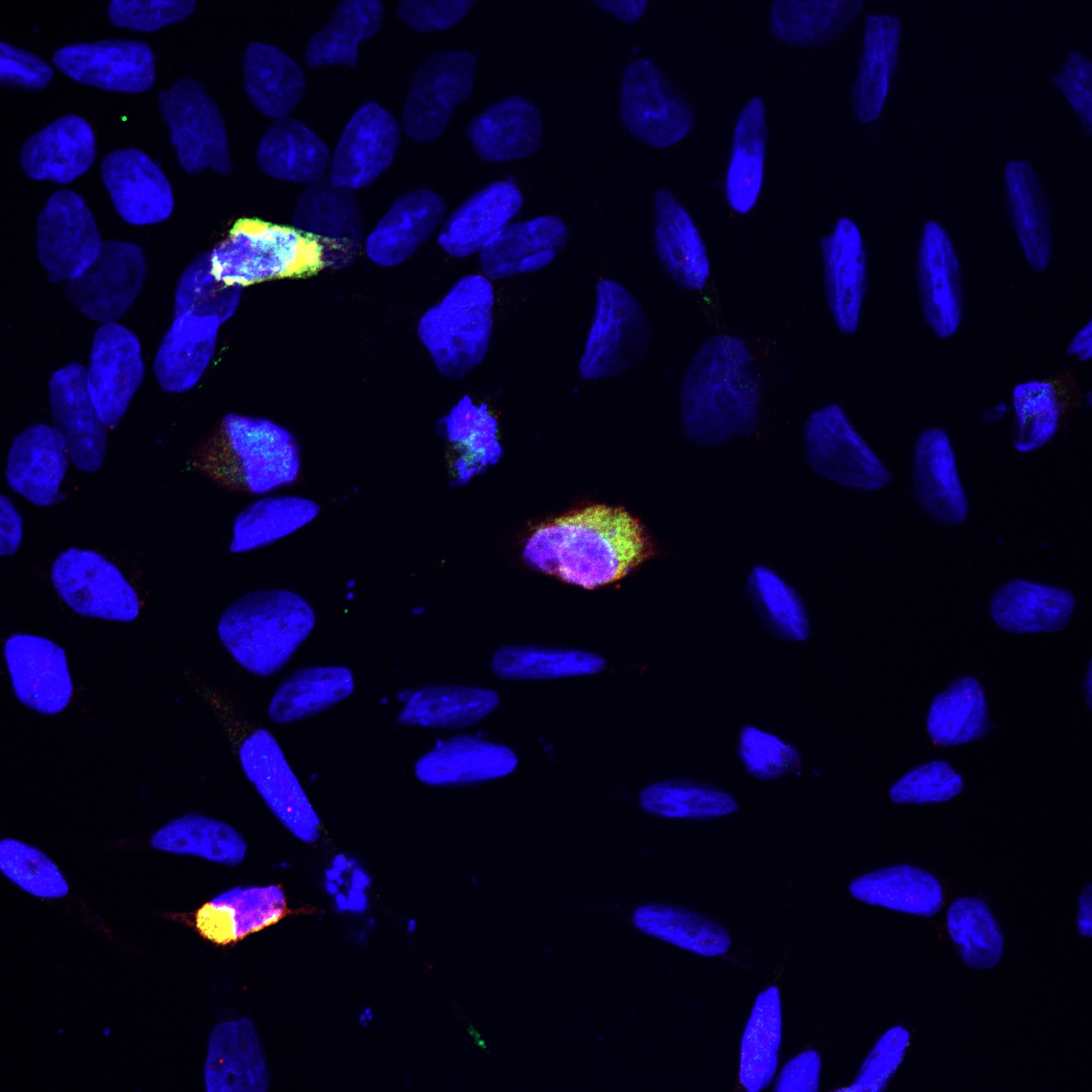

Supplement: Supplementary file 13 — Appendix Figure Source Data [file 44318_2025_487_MOESM13_ESM.zip › Appendix Figure S3/8A/40X/merge.jpg]

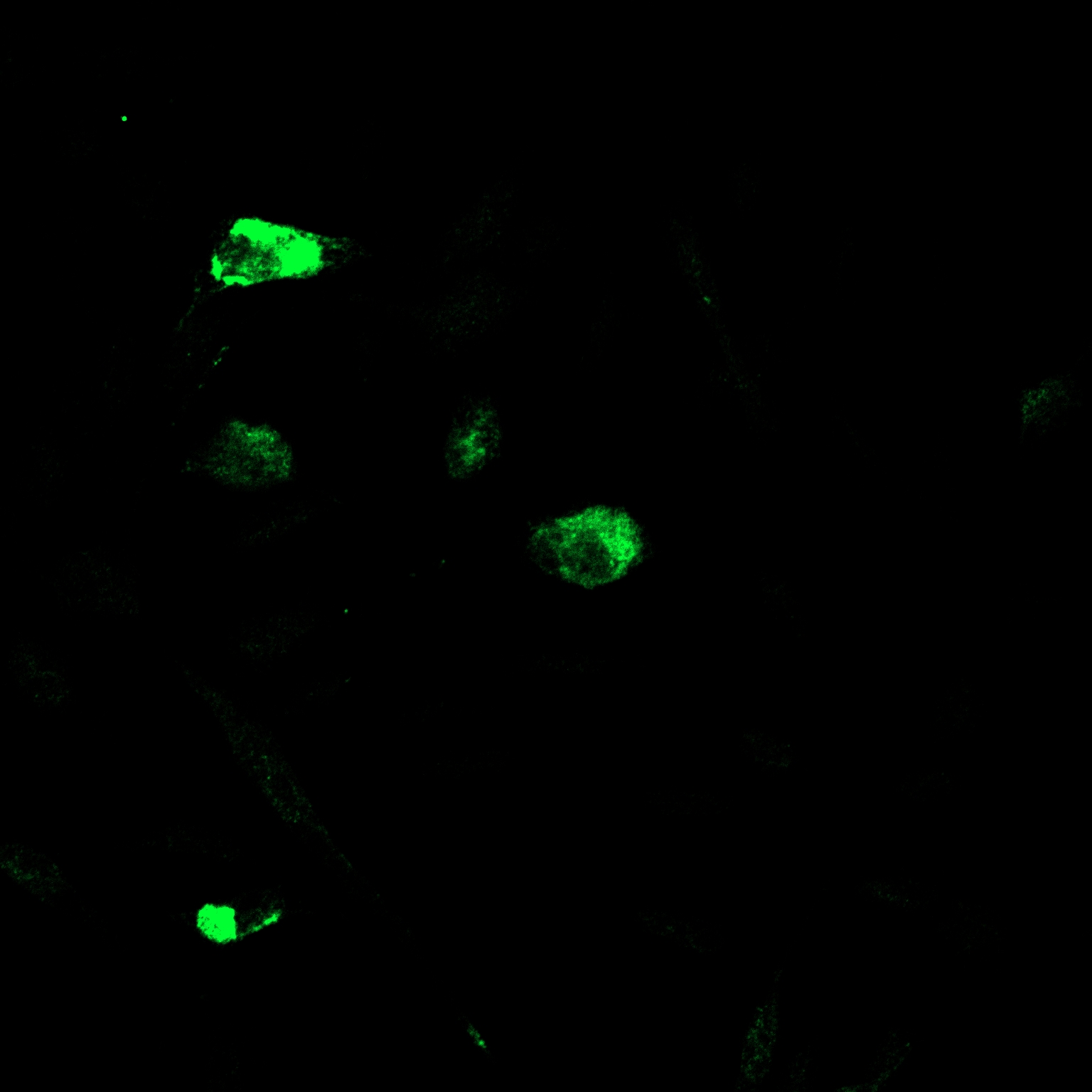

Supplement: Supplementary file 13 — Appendix Figure Source Data [file 44318_2025_487_MOESM13_ESM.zip › Appendix Figure S3/8A/40X/TRPM3-antibody.jpg]

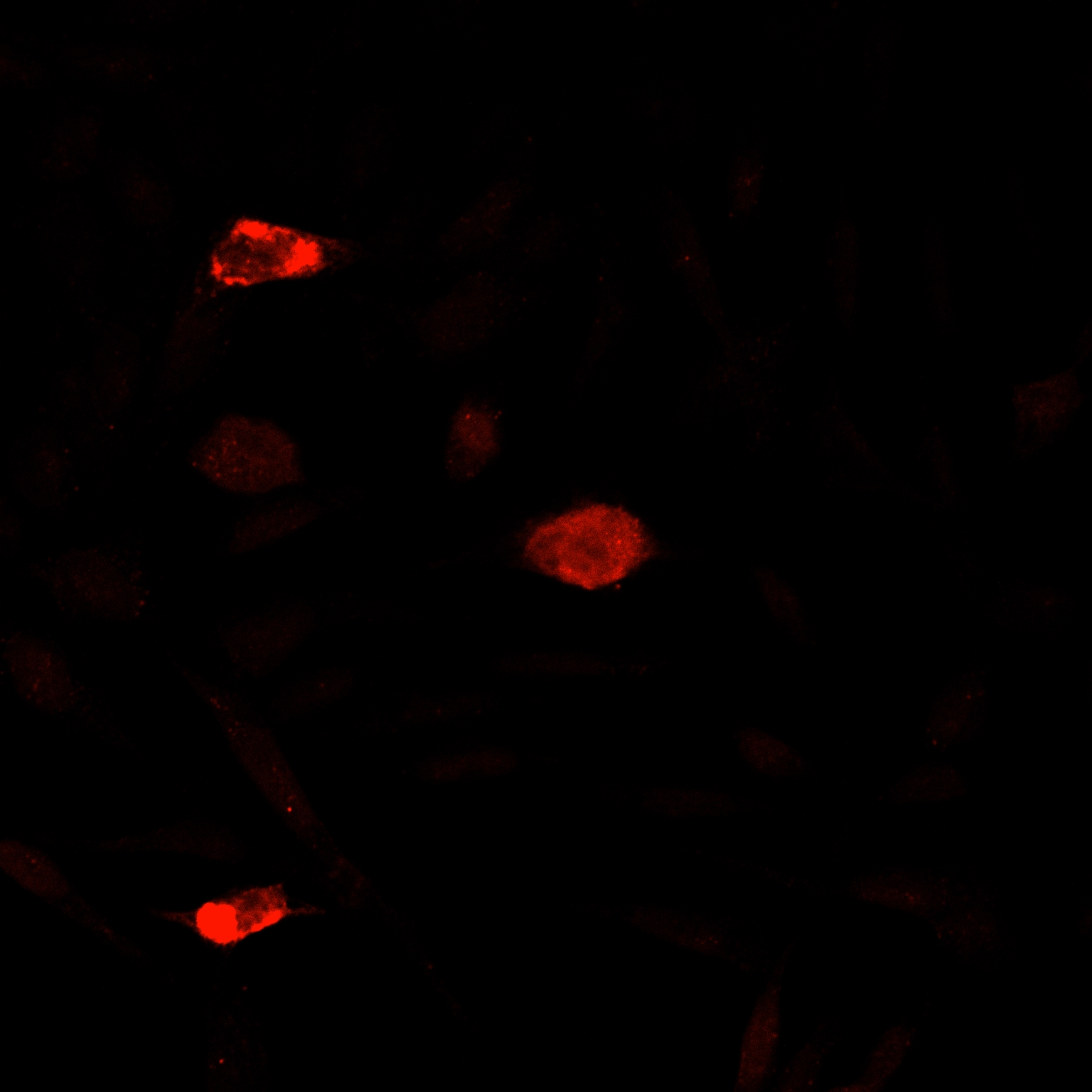

Supplement: Supplementary file 13 — Appendix Figure Source Data [file 44318_2025_487_MOESM13_ESM.zip › Appendix Figure S3/8A/40X/TRPM3-RFP.jpg]

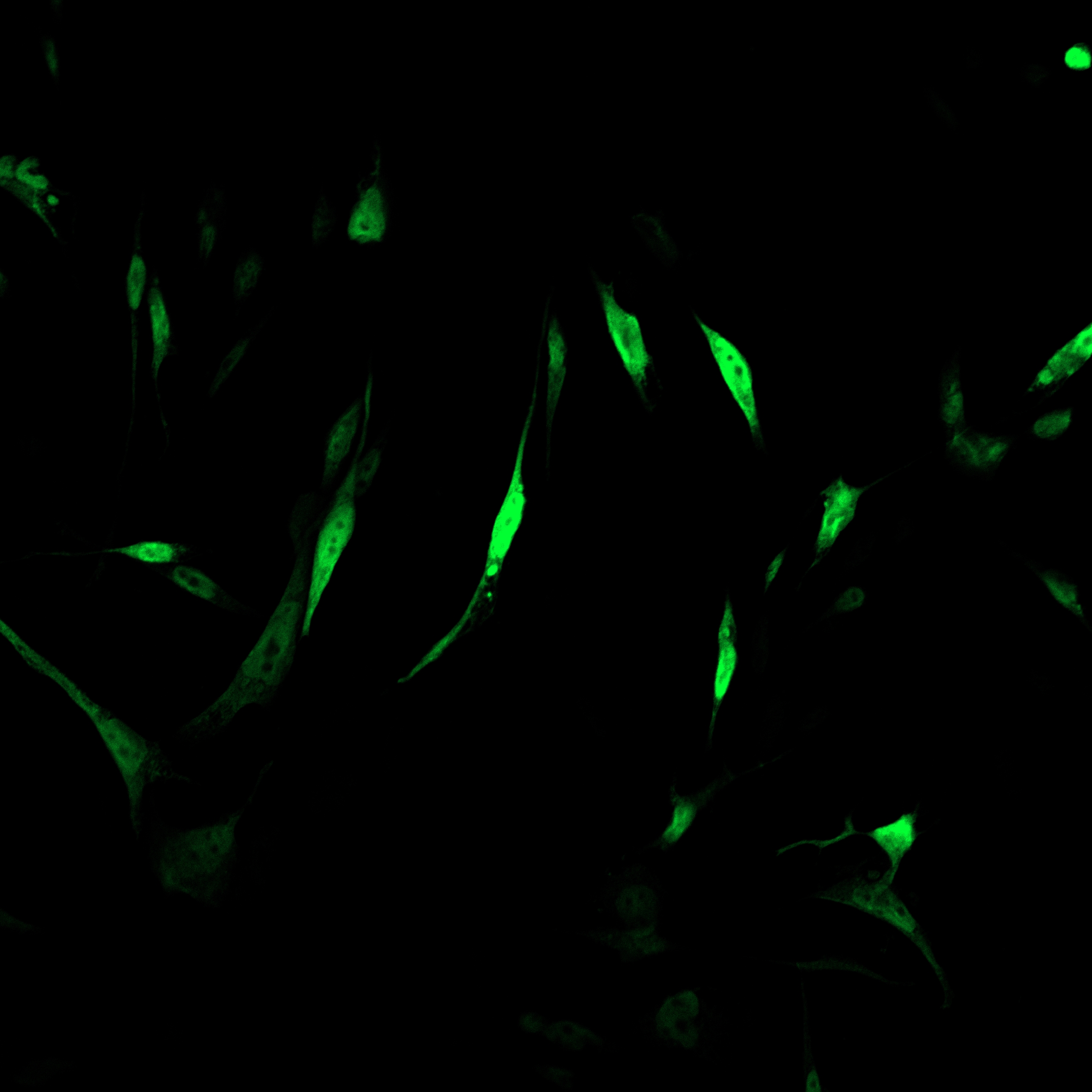

Supplement: Supplementary file 13 — Appendix Figure Source Data [file 44318_2025_487_MOESM13_ESM.zip › Appendix Figure S3/8B/20X/Copine-6(Santa).jpg]

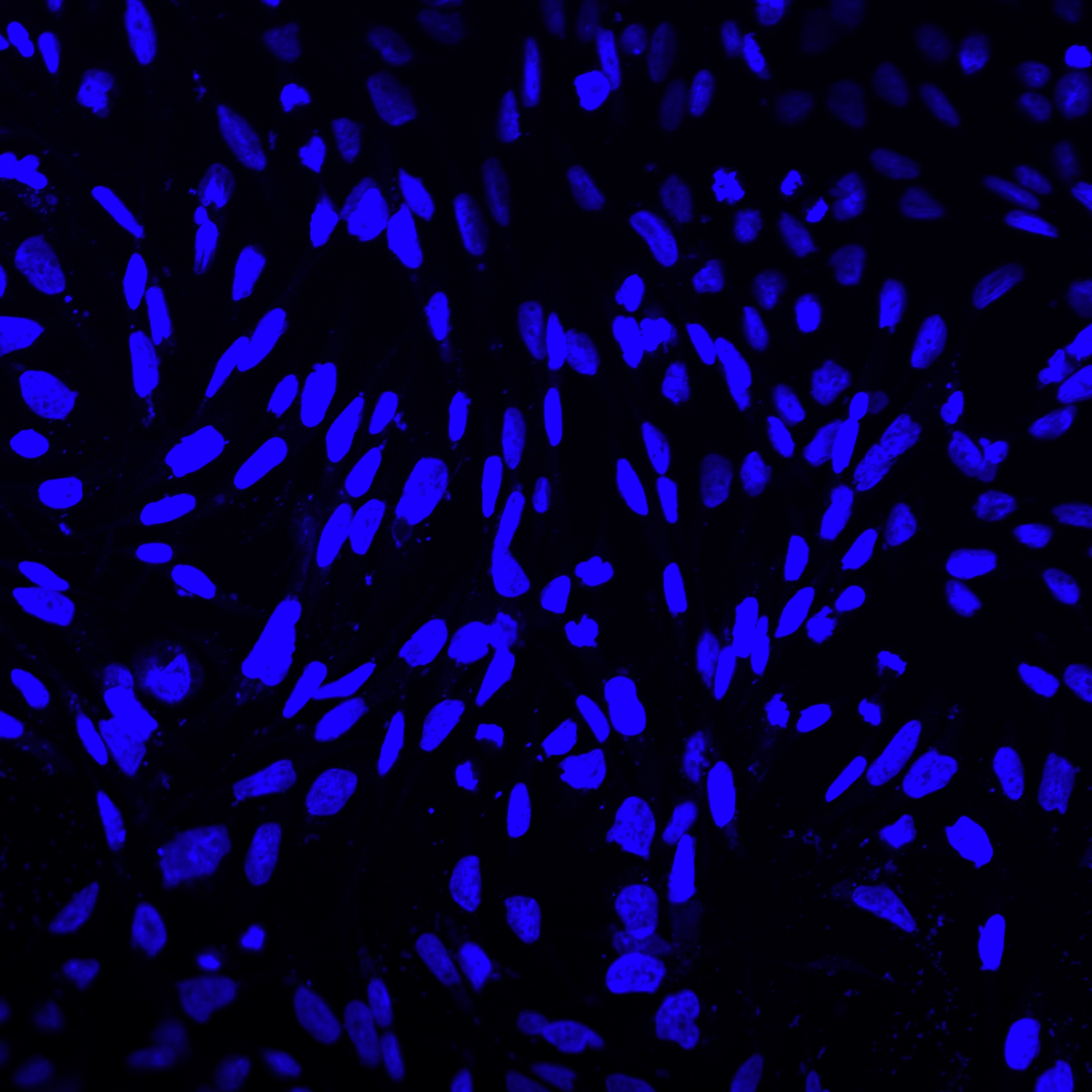

Supplement: Supplementary file 13 — Appendix Figure Source Data [file 44318_2025_487_MOESM13_ESM.zip › Appendix Figure S3/8B/20X/DAPI.jpg]

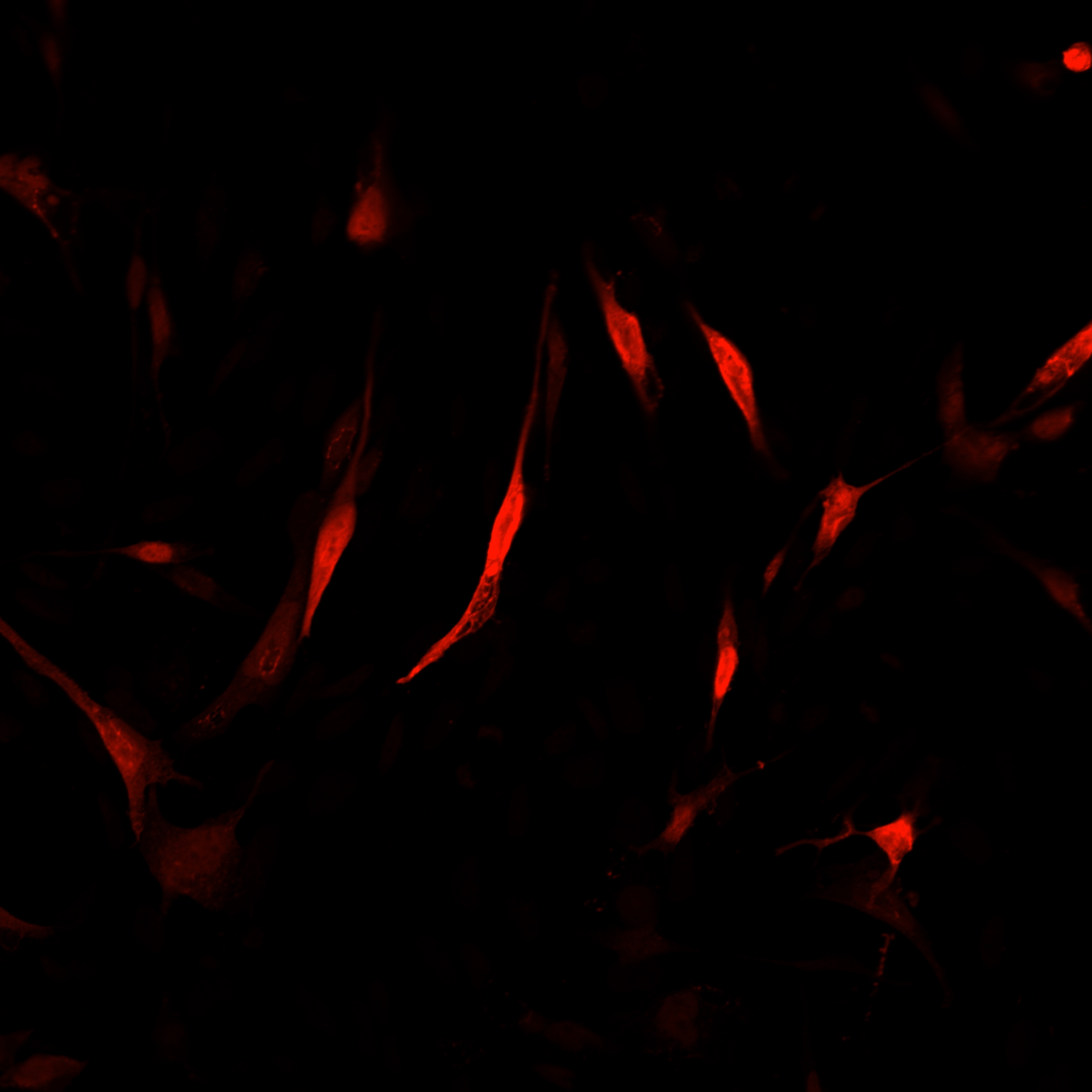

Supplement: Supplementary file 13 — Appendix Figure Source Data [file 44318_2025_487_MOESM13_ESM.zip › Appendix Figure S3/8B/20X/flag.jpg]

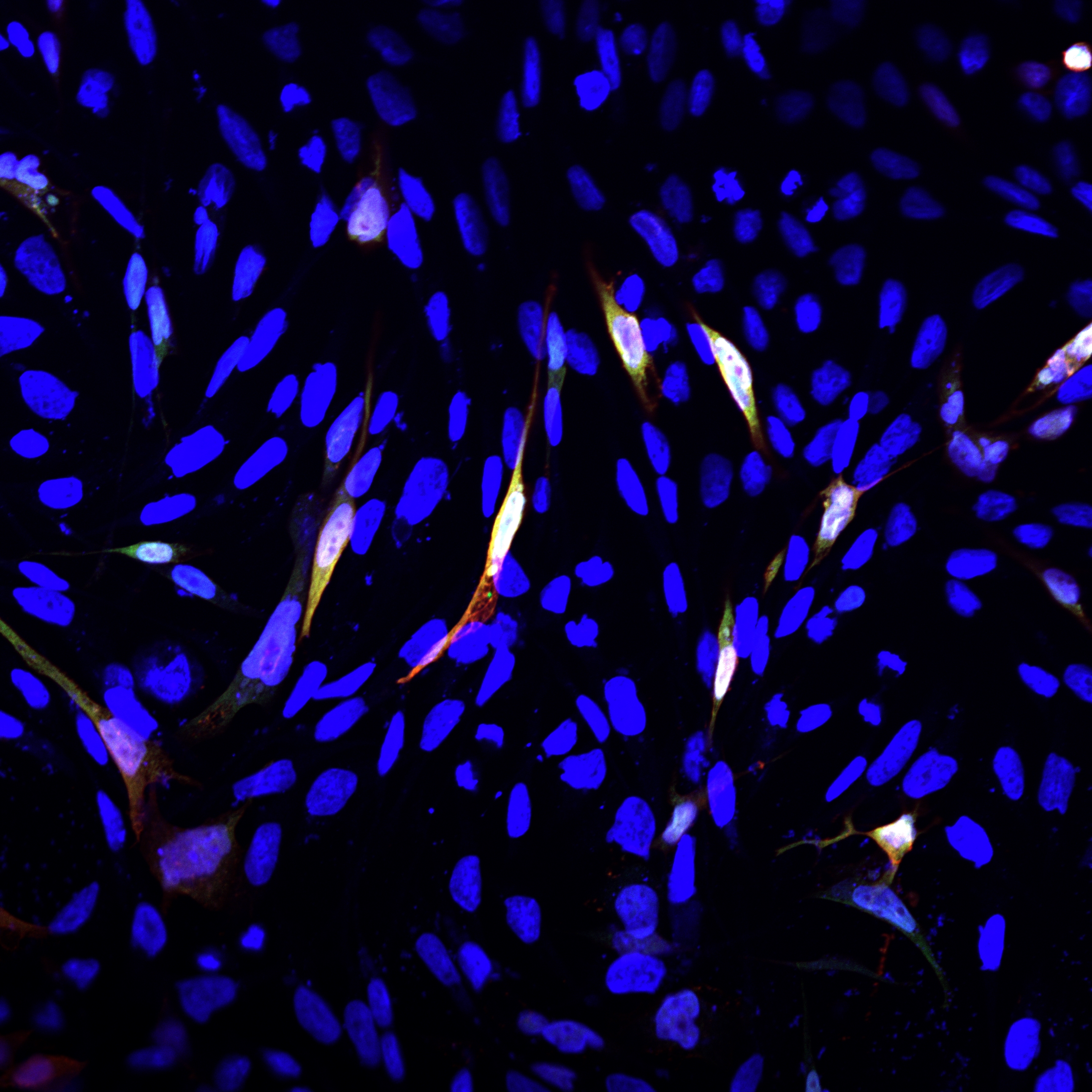

Supplement: Supplementary file 13 — Appendix Figure Source Data [file 44318_2025_487_MOESM13_ESM.zip › Appendix Figure S3/8B/20X/merge.jpg]

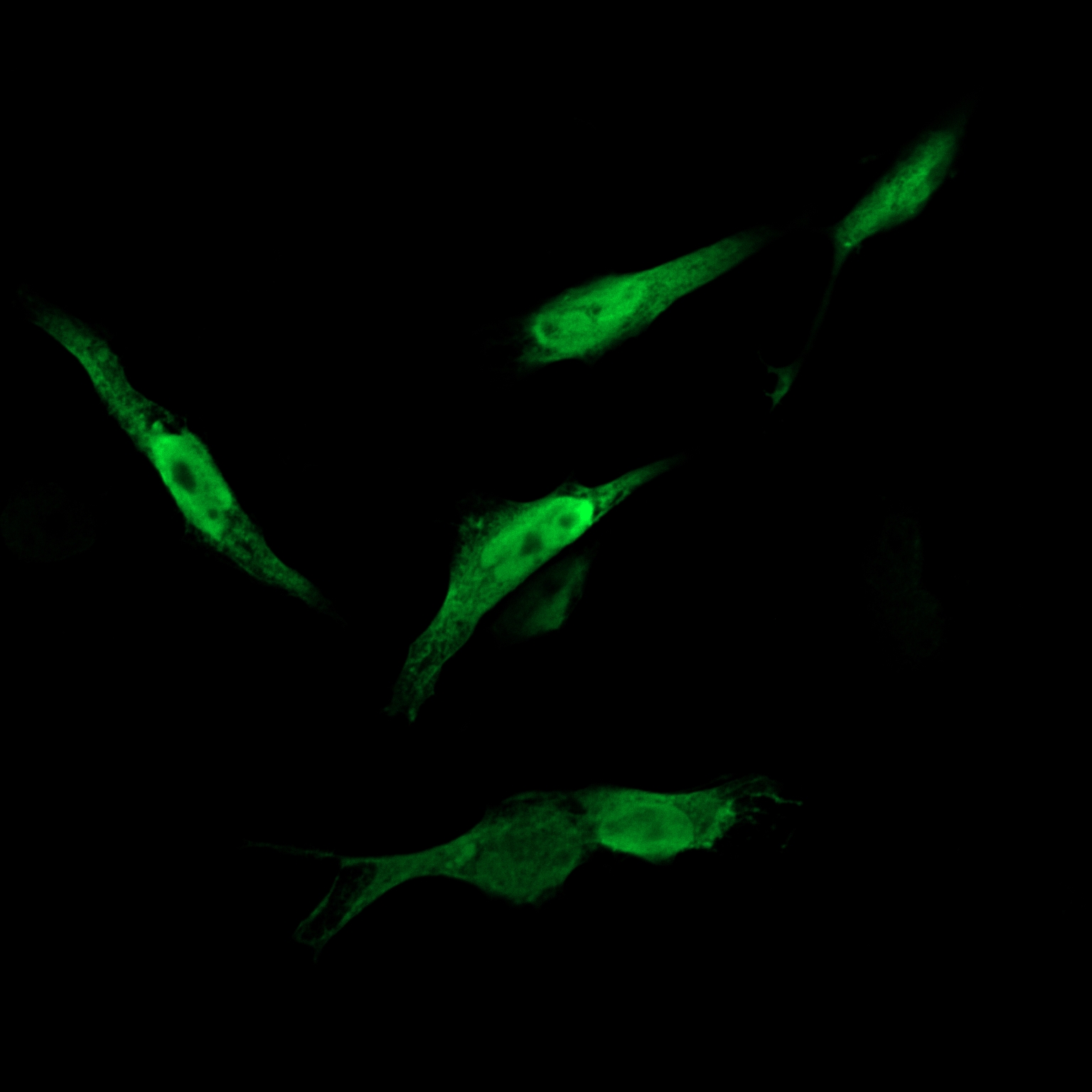

Supplement: Supplementary file 13 — Appendix Figure Source Data [file 44318_2025_487_MOESM13_ESM.zip › Appendix Figure S3/8B/40X/Copine-6(Santa).jpg]

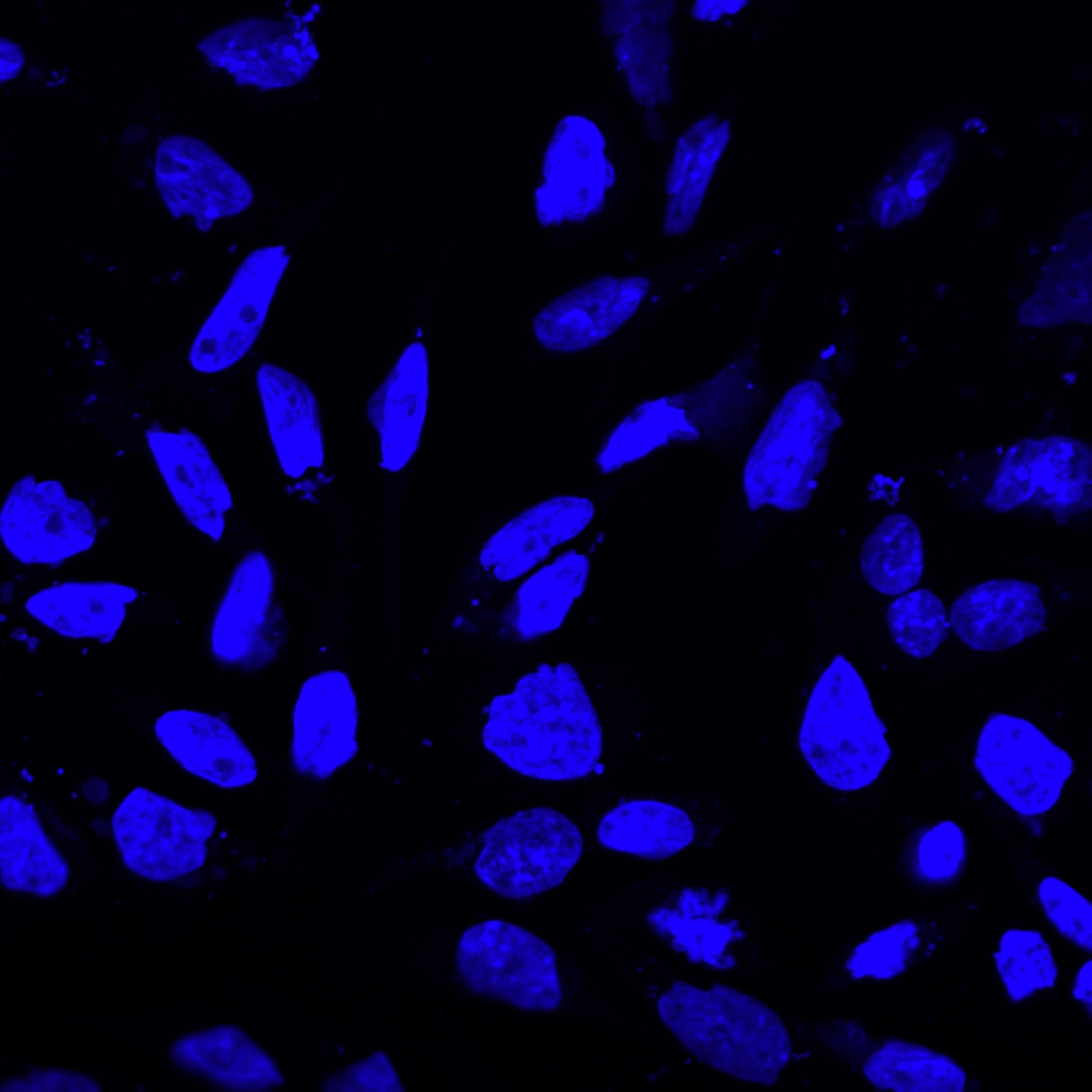

Supplement: Supplementary file 13 — Appendix Figure Source Data [file 44318_2025_487_MOESM13_ESM.zip › Appendix Figure S3/8B/40X/DAPI.jpg]

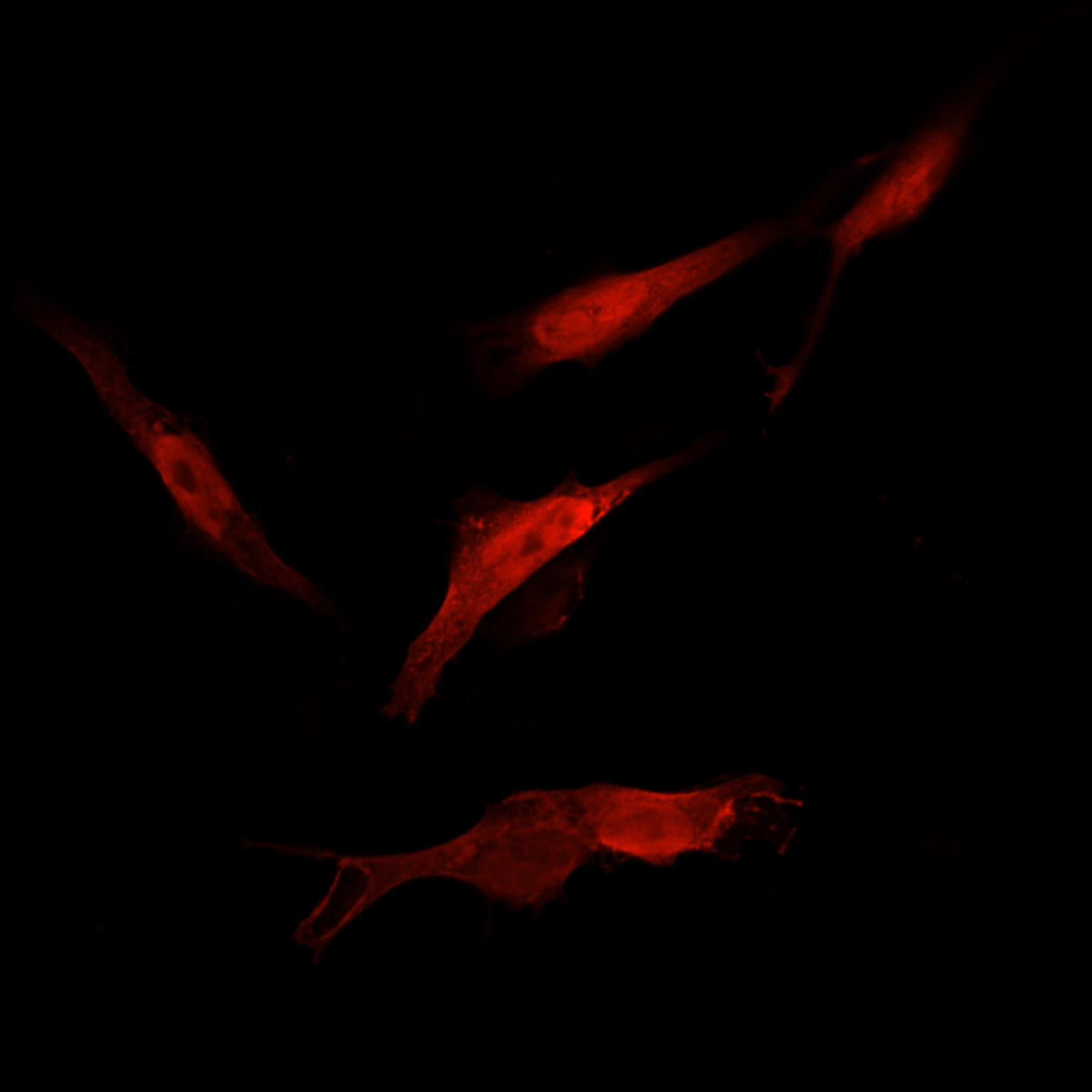

Supplement: Supplementary file 13 — Appendix Figure Source Data [file 44318_2025_487_MOESM13_ESM.zip › Appendix Figure S3/8B/40X/flag.jpg]

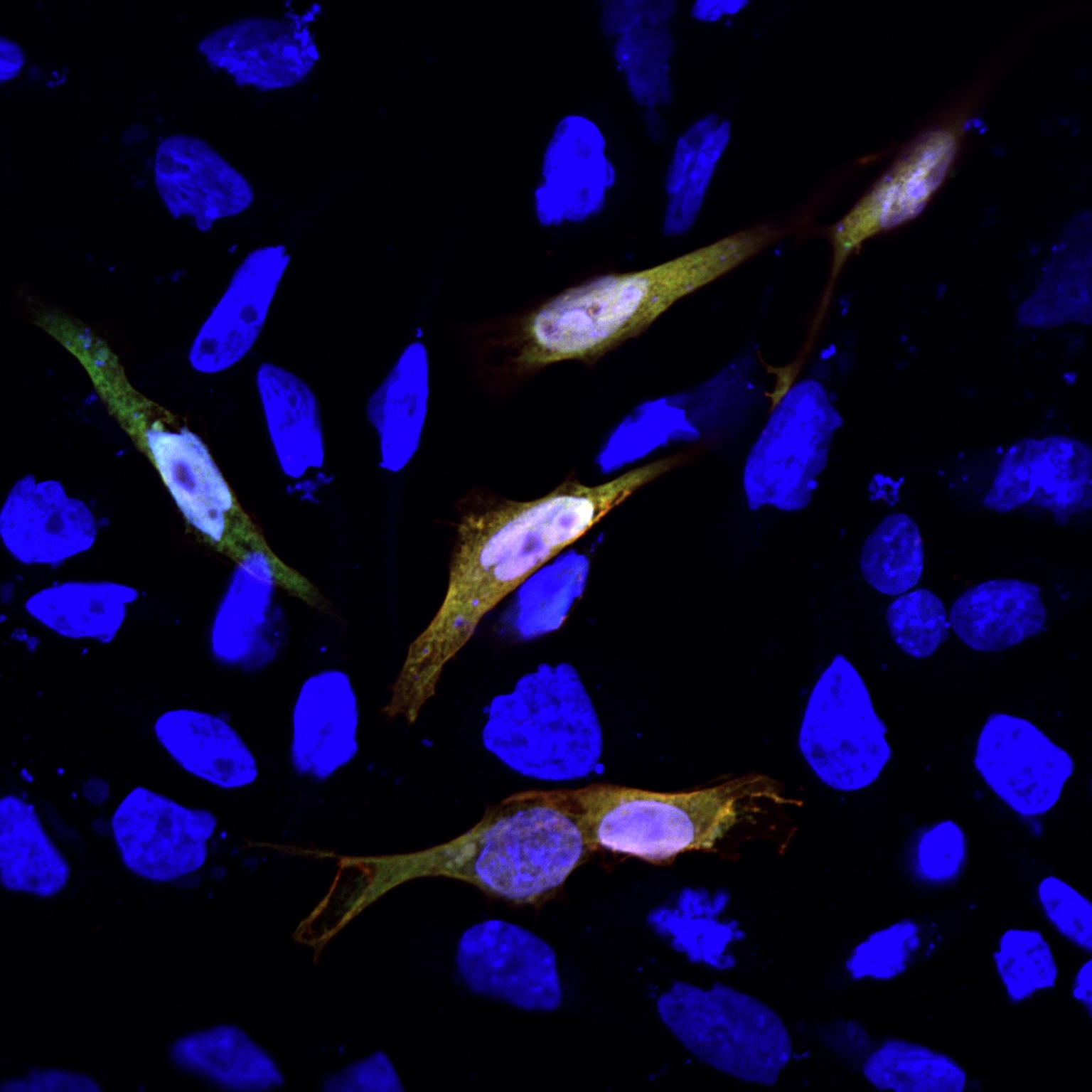

Supplement: Supplementary file 13 — Appendix Figure Source Data [file 44318_2025_487_MOESM13_ESM.zip › Appendix Figure S3/8B/40X/merge.jpg]

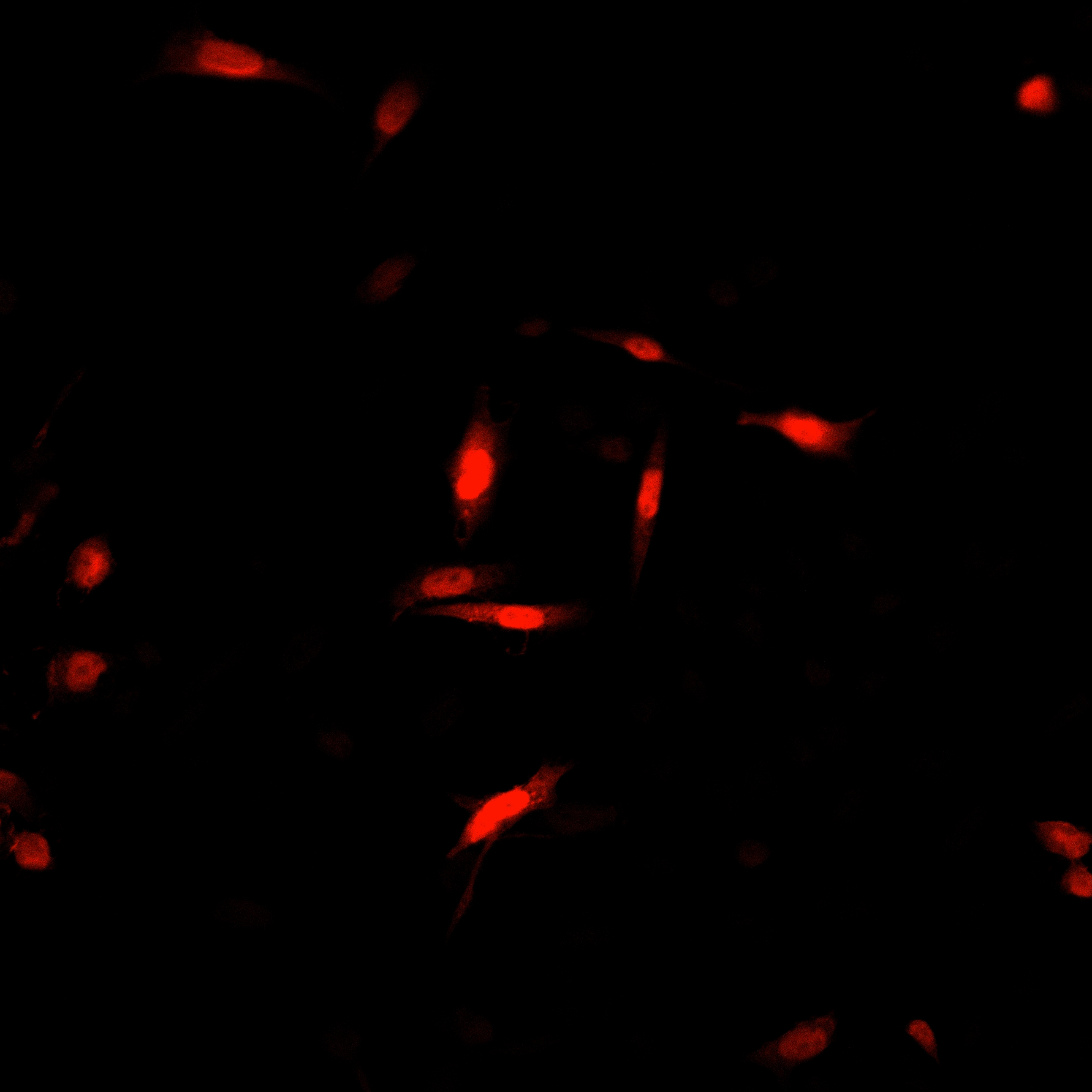

Supplement: Supplementary file 13 — Appendix Figure Source Data [file 44318_2025_487_MOESM13_ESM.zip › Appendix Figure S3/8C/20X/Copine-6(proteintech).jpg]

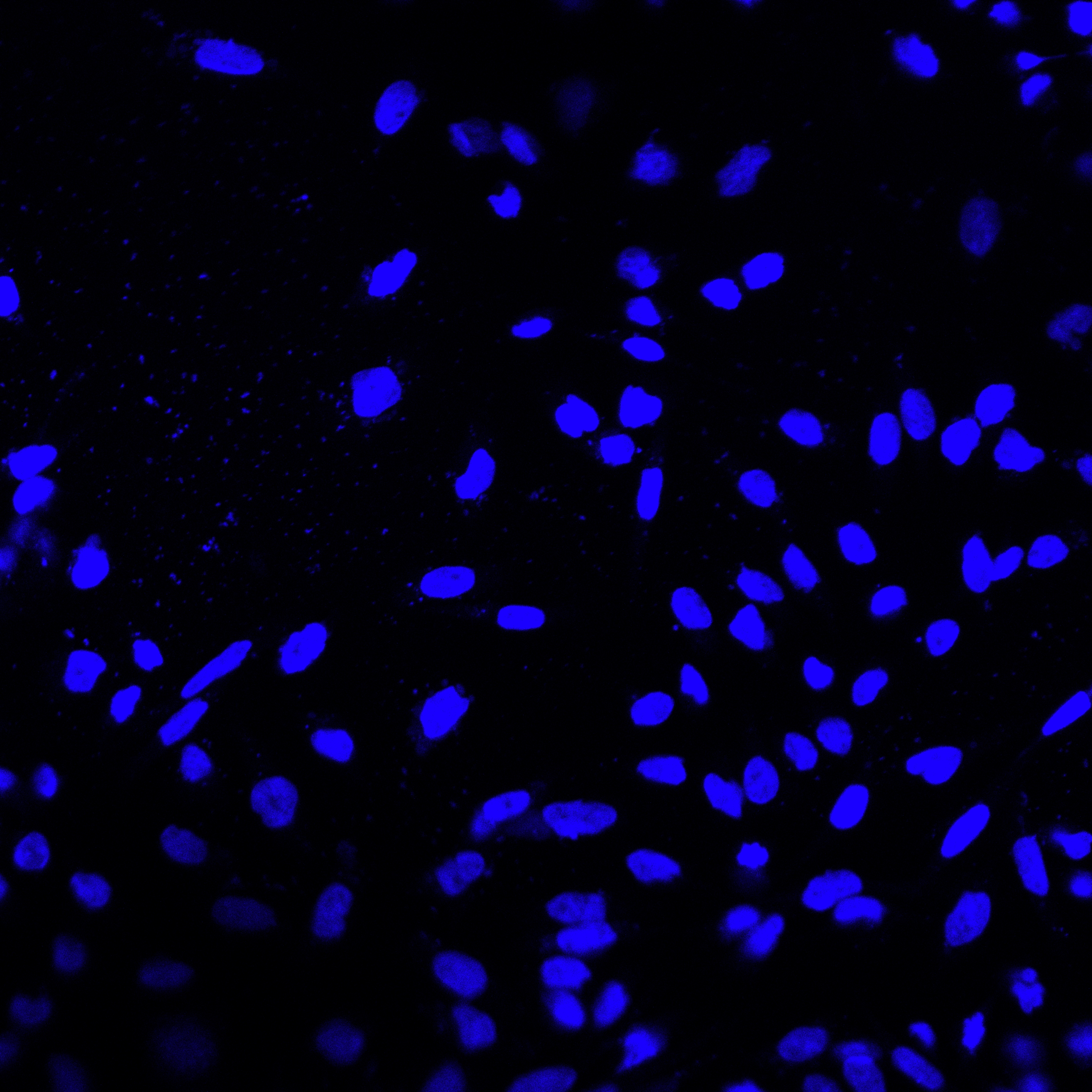

Supplement: Supplementary file 13 — Appendix Figure Source Data [file 44318_2025_487_MOESM13_ESM.zip › Appendix Figure S3/8C/20X/DAPI.jpg]

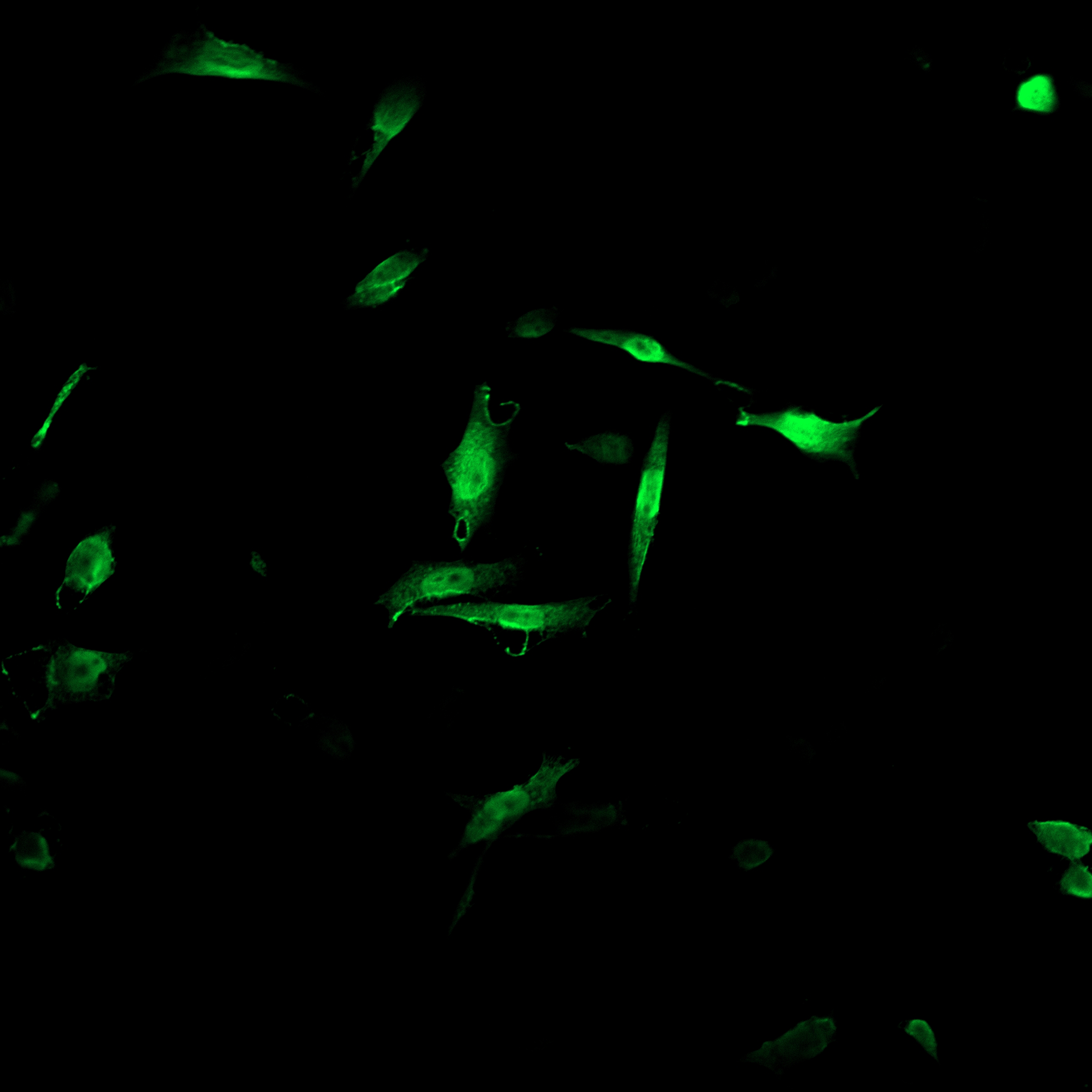

Supplement: Supplementary file 13 — Appendix Figure Source Data [file 44318_2025_487_MOESM13_ESM.zip › Appendix Figure S3/8C/20X/flag.jpg]

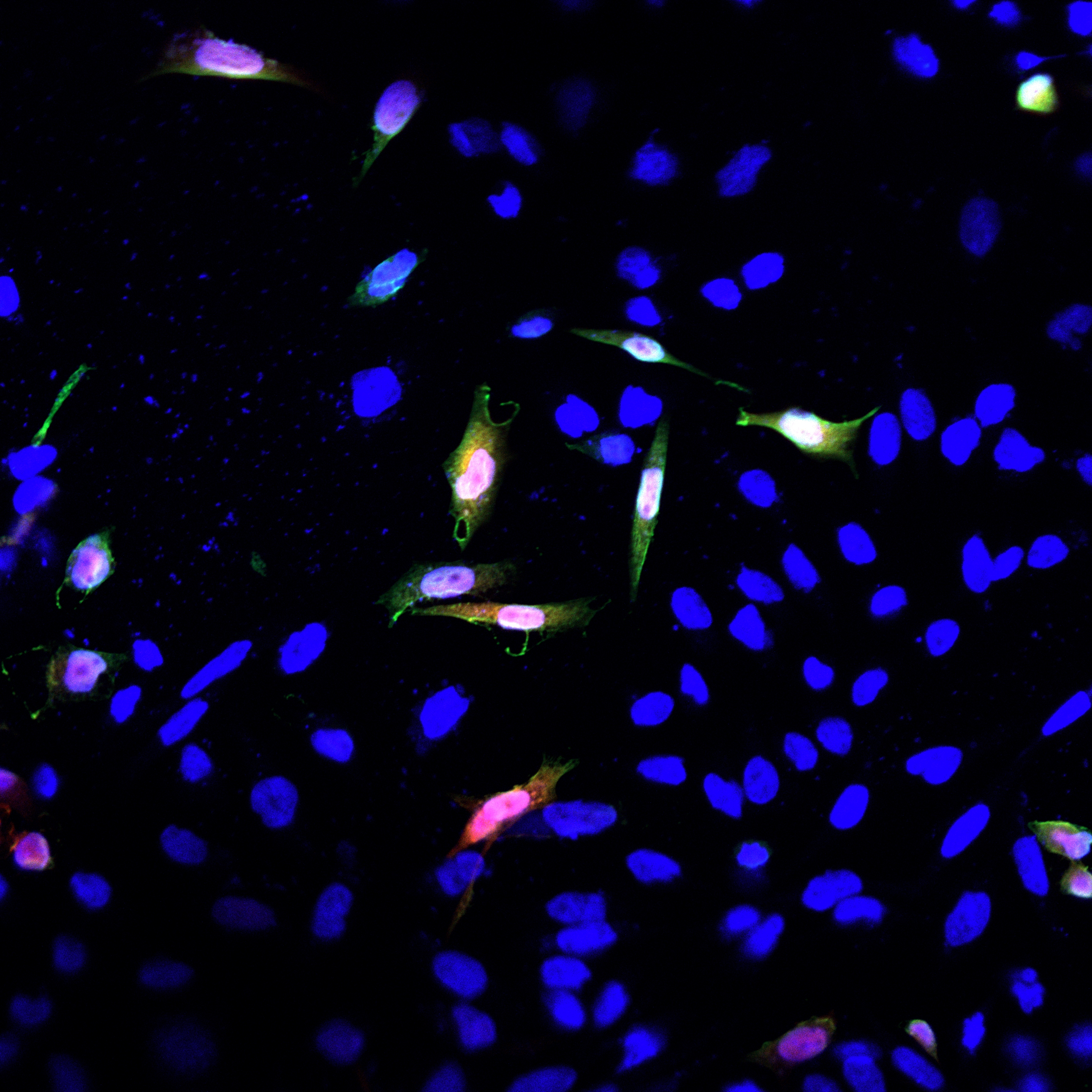

Supplement: Supplementary file 13 — Appendix Figure Source Data [file 44318_2025_487_MOESM13_ESM.zip › Appendix Figure S3/8C/20X/merge.jpg]

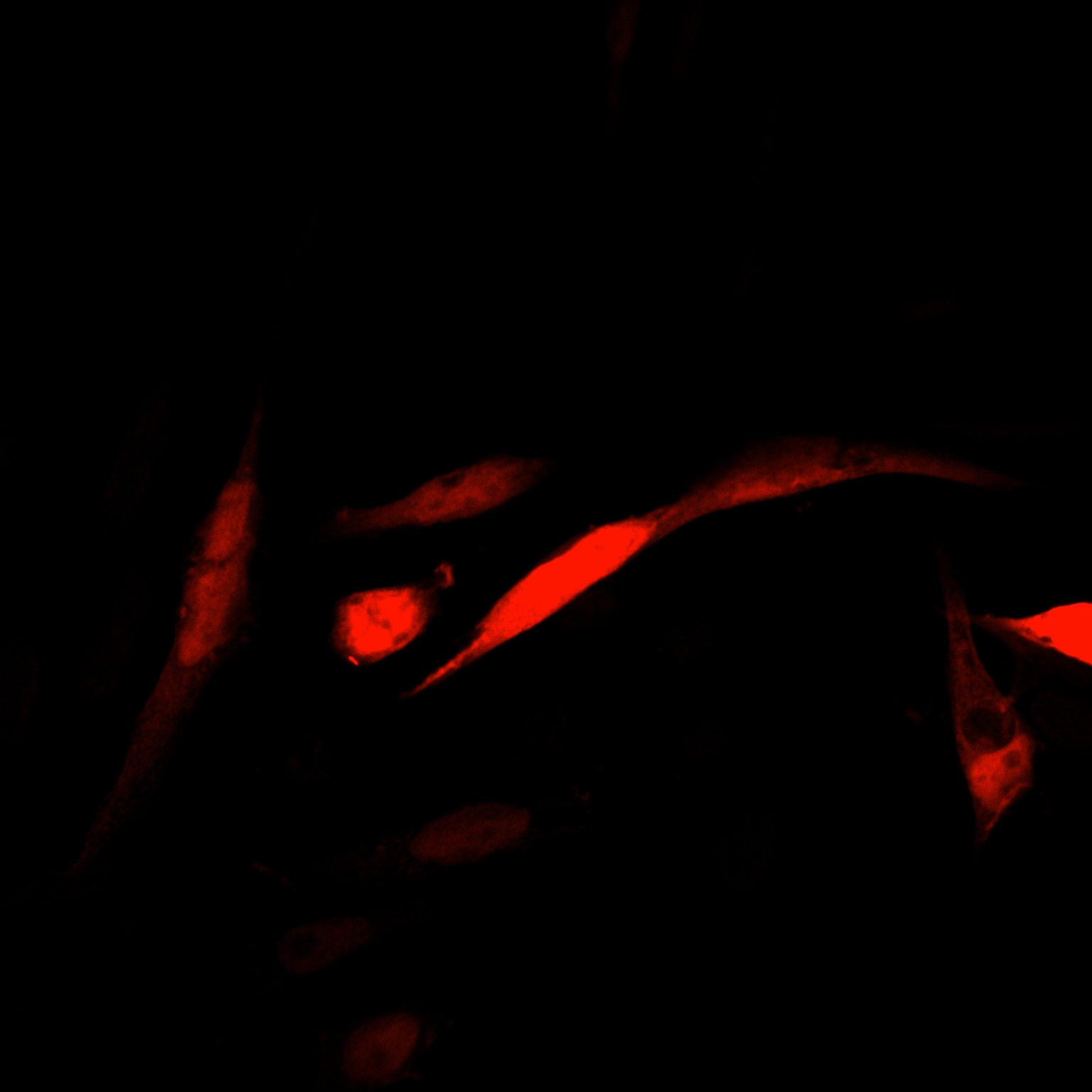

Supplement: Supplementary file 13 — Appendix Figure Source Data [file 44318_2025_487_MOESM13_ESM.zip › Appendix Figure S3/8C/40X/Copine-6(proteintech).jpg]

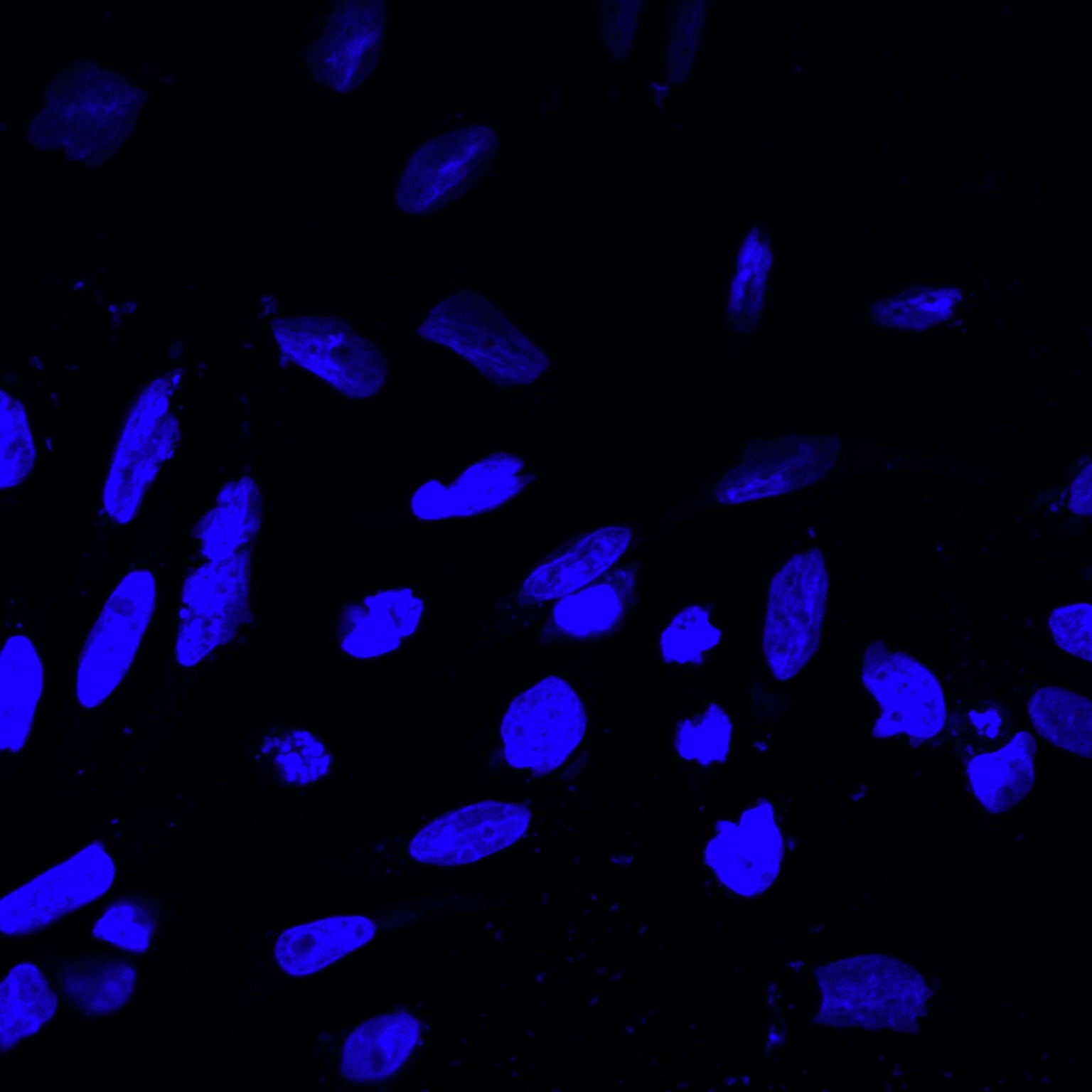

Supplement: Supplementary file 13 — Appendix Figure Source Data [file 44318_2025_487_MOESM13_ESM.zip › Appendix Figure S3/8C/40X/DAPI.jpg]

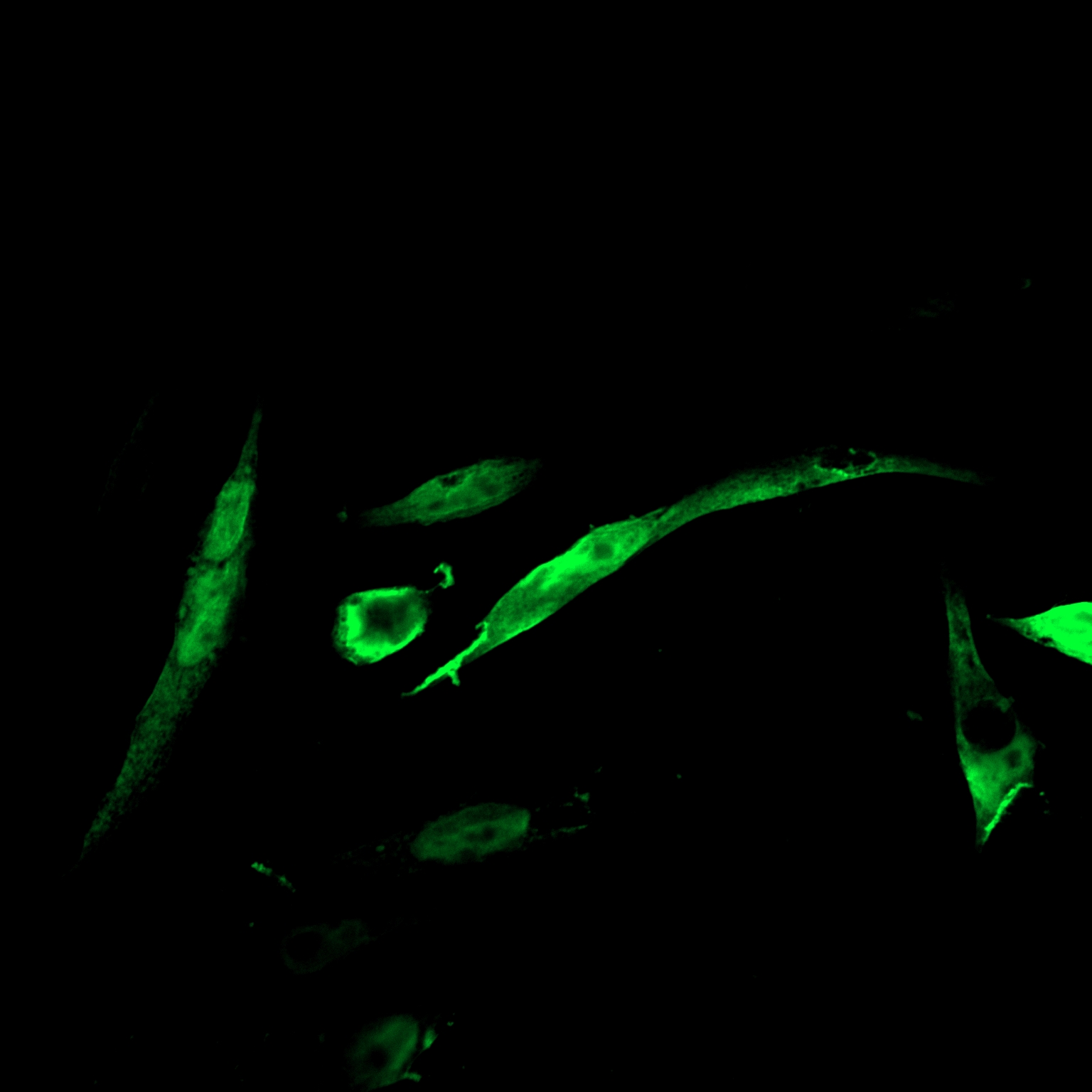

Supplement: Supplementary file 13 — Appendix Figure Source Data [file 44318_2025_487_MOESM13_ESM.zip › Appendix Figure S3/8C/40X/flag.jpg]

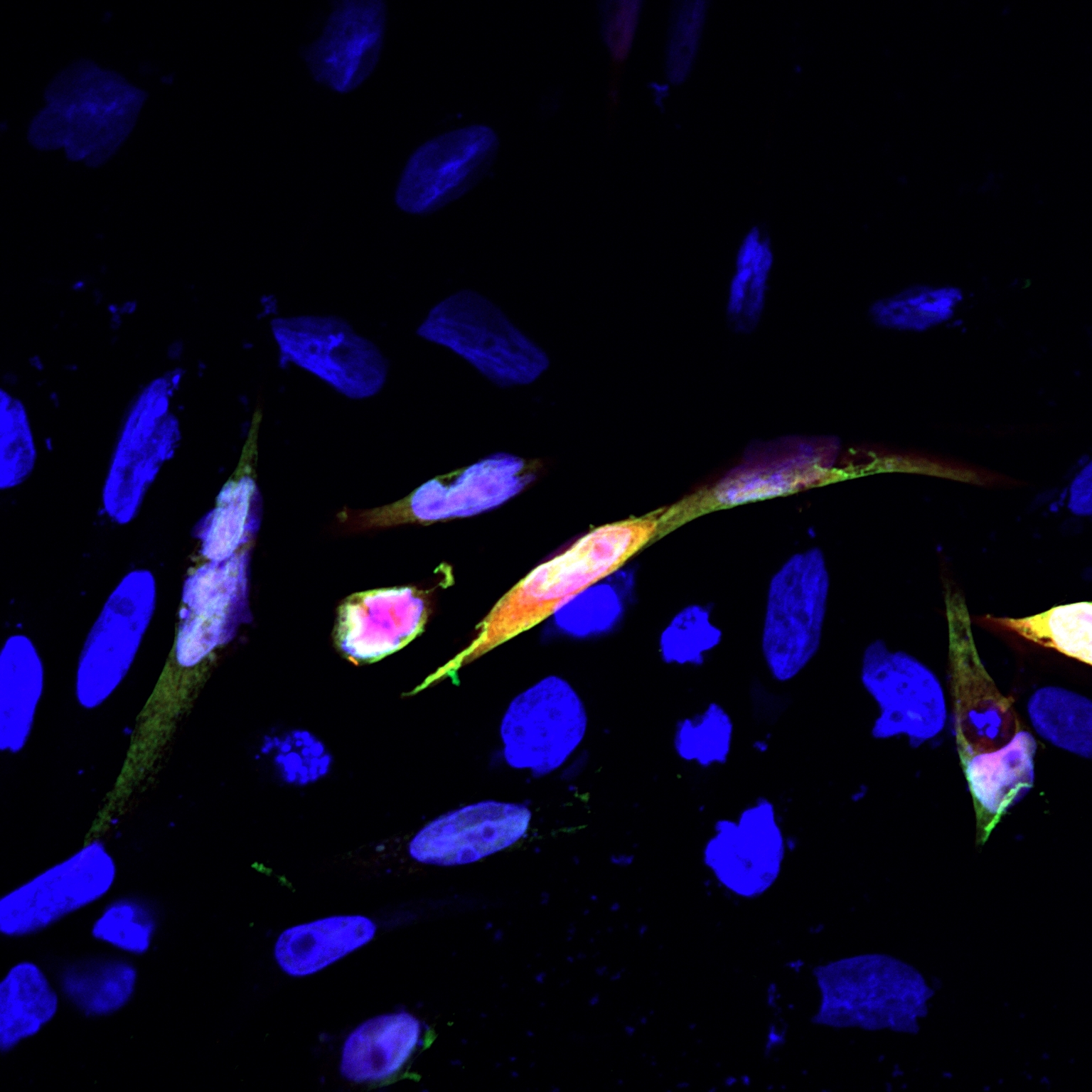

Supplement: Supplementary file 13 — Appendix Figure Source Data [file 44318_2025_487_MOESM13_ESM.zip › Appendix Figure S3/8C/40X/merge.jpg]
