# Supplementary material for: Copine-6 is a TRPM3 escort protein controlling the sensitivity of sensory neurons to noxious heat
Source: EMBO J. 2025 Jun 19;44(15):4222–51. doi: 10.1038/s44318-025-00487-0 (PMC12317139; doi:10.1038/s44318-025-00487-0)
Supplement: Supplementary file 14 — Expanded View Figures [file 44318_2025_487_MOESM14_ESM.pdf]

Expanded View Figures

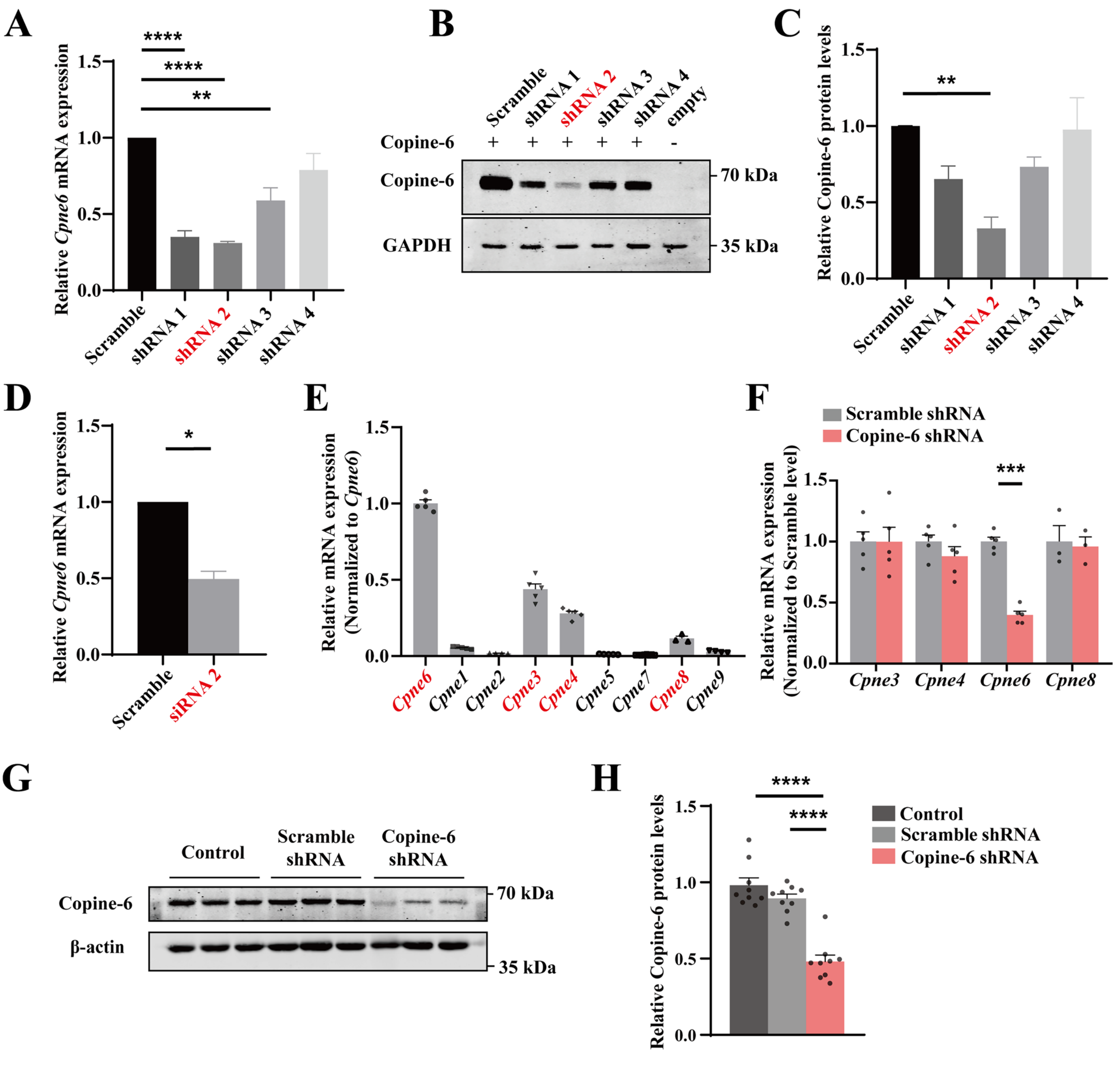

Figure EV1. Validation of *Cpne6* knockdown efficiency in vitro and in vivo in the rat.

(**A**) Summary data of *Cpne6* mRNA knockdown by four *Cpne6*-targeting shRNAs in HEK293T cells overexpressing *Cpne6*; $n = 4$; Data are shown as mean ± SEM; Scramble vs. shRNA1, ****$P < 0.0001$; Scramble vs. shRNA2, ****$P < 0.0001$; Scramble vs. shRNA3, **$P = 0.0014$ (one-way ANOVA followed by Bonferroni multiple comparisons test). (**B**) Example of western blot assay for Copine-6 expression in HEK293T cells co-transfected with Copine-6 and four shRNA constructs used. (**C**) Quantification of the Copine-6 protein expression knockdown by Copine-6 shRNAs in HEK293T cells (from the experiments as these shown in (**B**); $n = 4$; Data are shown as mean ± SEM; **$P = 0.0024$ (one-way ANOVA followed by Bonferroni multiple comparisons test). (**D**) Knockdown of *Cpne6* mRNA by siRNA2 in cultured DRG neurons; $n = 4$; Data are shown as mean ± SEM; *$P = 0.029$ (Mann–Whitney *U* test). (**E**) The mRNA expression levels of *Cpne1-9* genes in the whole DRG of rats, relative to *Cpne6*. Genes displaying considerable expression are highlighted in red. *Cpne1, 3-6*, $n = 5$; *Cpne2, 9*, $n = 4$; *Cpne7*, $n = 8$; *Cpne8*, $n = 3$; Data are shown as mean ± SEM. (**F**) qPCR analysis of the *Cpne3, 4, 6* and *8* mRNA in the whole DRG of rats injected with AAV9-U6-Copine-6-shRNA-EGFP or AAV9-U6-Scramble-shRNA-EGFP (45 days after viral injection into the DRG); *Cpne3, 4, 6*, $n = 5$; *Cpne8*, $n = 3$. Data are shown as mean ± SEM. ***$P < 0.001$ (two-tailed independent *t* test). (**G**) Example of western blot assay for Copine-6 expression in the DRGs of rats with viral DRG-targeted shRNA knockdown of *Cpne6*. (**H**) Quantification of the Copine-6 protein expression in the DRG (from the experiments as these shown in **G**); $n = 9$; Data are shown as mean ± SEM; ****$P < 0.0001$ (one-way ANOVA followed by Bonferroni multiple comparisons test). Source data are available online for this figure.

 © The Author(s)

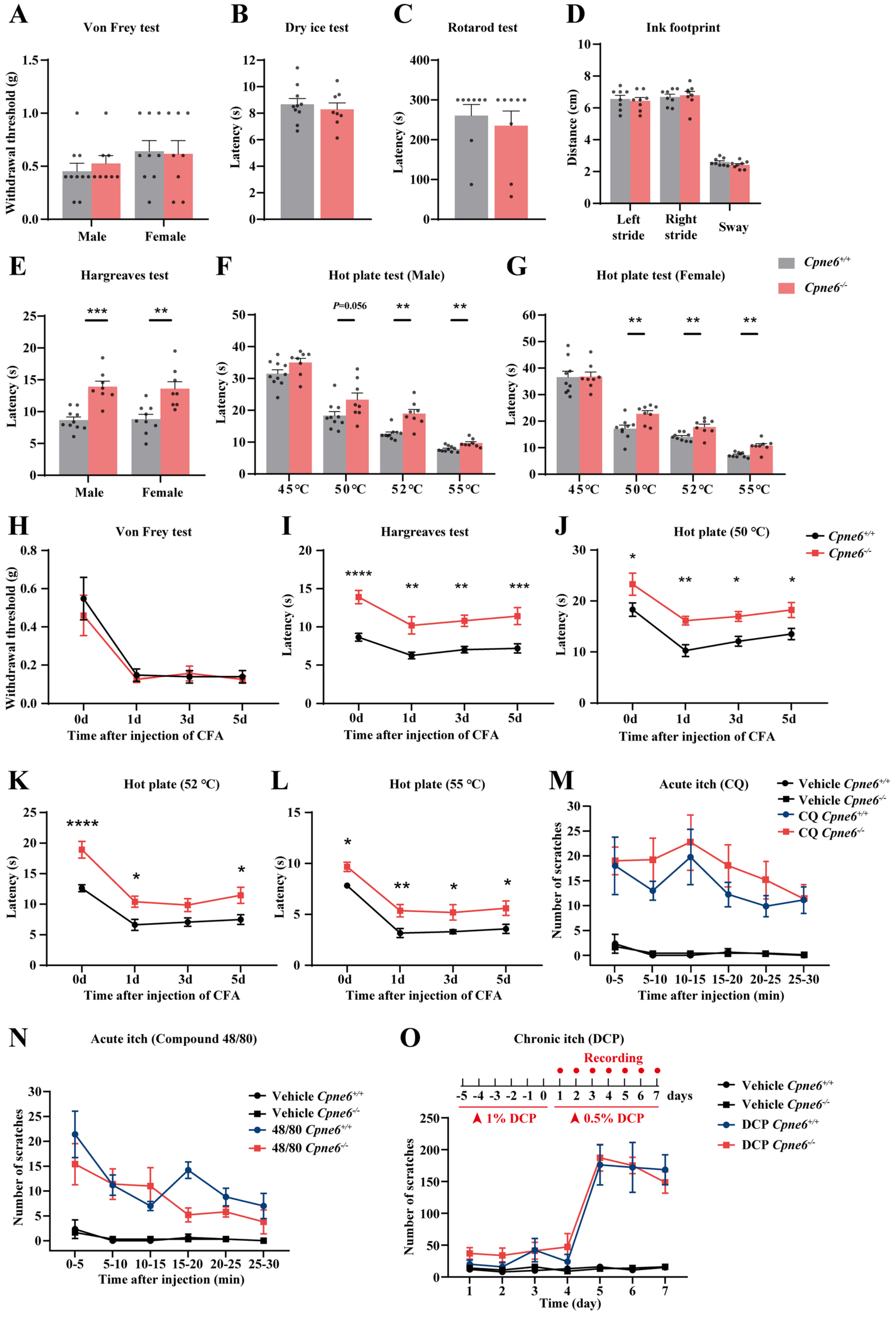

A
Von Frey test
Withdrawal threshold (g)
Male
Female
B
Dry ice test
Latency (s)
C
Rotarod test
Latency (s)
D
Ink footprint
Distance (cm)
Left stride
Right stride
Sway
E
Hargreaves test
Latency (s)
***
**
Male
Female
F
Hot plate test (Male)
P=0.056
**
**
45°C
50°C
52°C
55°C
G
Hot plate test (Female)
Cpne6+/+
Cpne6-/-
H
Von Frey test
Time after injection of CFA
I
Hargreaves test
****
***
J
Hot plate (50 °C)
K
Hot plate (52 °C)
L
Hot plate (55 °C)
M
Acute itch (CQ)
Vehicle Cpne6+/+
Vehicle Cpne6-/-
CQ Cpne6+/+
CQ Cpne6-/-
Number of scratches
Time after injection (min)
N
Acute itch (Compound 48/80)
48/80 Cpne6+/+
48/80 Cpne6-/-
O
Chronic itch (DCP)
Recording
days
1% DCP
0.5% DCP
DCP Cpne6+/+
DCP Cpne6-/-
Time (day)

© The Author(s)

Figure EV2. Additional behavioral characterization of *Cpne6*$^{-/-}$ mice.

(**A–F**) The somatosensory and motor coordination tests were performed on *Cpne6*$^{+/+}$ and *Cpne6*$^{-/-}$ mice, as follows: Von Frey test (**A**), dry ice test (**B**), rotarod test (**C**), ink footprint test (**D**), Hargreaves test (**E**), hot plate test (**F**, **G**). (**A**, **E–G**): $n = 10$ mice in male *Cpne6*$^{+/+}$ group, $n = 9$ mice in female *Cpne6*$^{+/+}$ group and $n = 8$ mice in *Cpne6*$^{-/-}$ groups of either gender. (**B**) $n = 10$ mice in in *Cpne6*$^{+/+}$, $n = 8$ mice in *Cpne6*$^{-/-}$ group. (**C**, **D**) $n = 8$ mice in both groups. Data are shown as mean ± SEM. (**E**) Male, ***$P < 0.001$; Female, **$P = 0.003$. (**F**) 50 °C, $P = 0.056$; 52 °C, **$P = 0.001$; 55 °C, **$P = 0.002$. (**G**) 50 °C, **$P = 0.007$; 52 °C, **$P = 0.005$; 55 °C, **$P = 0.001$ (two-tailed independent *t* test). (**H–L**) Knockout of *Cpne6* reduced CFA-induced heat hyperalgesia (**I–L**) but not mechanical allodynia (**H**); $n = 10$ mice in all *Cpne6*$^{+/+}$ groups and $n = 8$ mice in all *Cpne6*$^{-/-}$ groups. Data are shown as mean ± SEM. I, 0 d, ****$P < 0.0001$; 1 d, **$P = 0.0012$; 3 d, **$P = 0.0019$; 5 d, ***$P = 0.005$. J, 0 d, *$P = 0.0308$; 1 d, **$P = 0.0078$; 3 d, *$P = 0.038$; 5 d, *$P = 0.0465$. (**K**) 0 d, ****$P < 0.0001$; 1 d, *$P = 0.025$; 5 d, *$P = 0.0168$. (**L**) 0 d, *$P = 0.0406$; 1 d, **$P = 0.0099$; 3 d, *$P = 0.0334$; 5 d, *$P = 0.0198$. Two-way ANOVA with Bonferroni multiple comparisons test. (**M–O**) Acute and chronic itch tests. (**M**) Histamine-independent acute itch model. Chloroquine (CQ) (200 μg/50 μL, vehicle: Saline) was intradermally injected into dorsal neck area and number of scratching attempts quantified during 30 min period. $n = 3$ mice in each vehicle group and $n = 8$ mice in each CQ group. Data are shown as mean ± SEM. (**N**) Histamine-dependent acute itch model. Compound 48/80 (100 μg/50 μL, vehicle: Saline) was intradermally injected into dorsal neck area and number of scratching attempts quantified during 30 min period. $n = 3$ mice in each vehicle group and $n = 5$ mice in each 48/80 group. Data are shown as mean ± SEM. (**O**) Chronic itch model. Shown on the top is the experimental timeline. Diphenylcyclopropenone (DCP, 1%, then 0.5% in acetone, as indicated) was applied to the dorsal neck area (see "Methods") and scratching behavior recorded for 30 min after each application during seven consecutive days; vehicle: acetone; $n = 3$ mice in each vehicle group and $n = 5$ mice in each DCP group. Data are shown as mean ± SEM. Source data are available online for this figure.

© The Author(s)

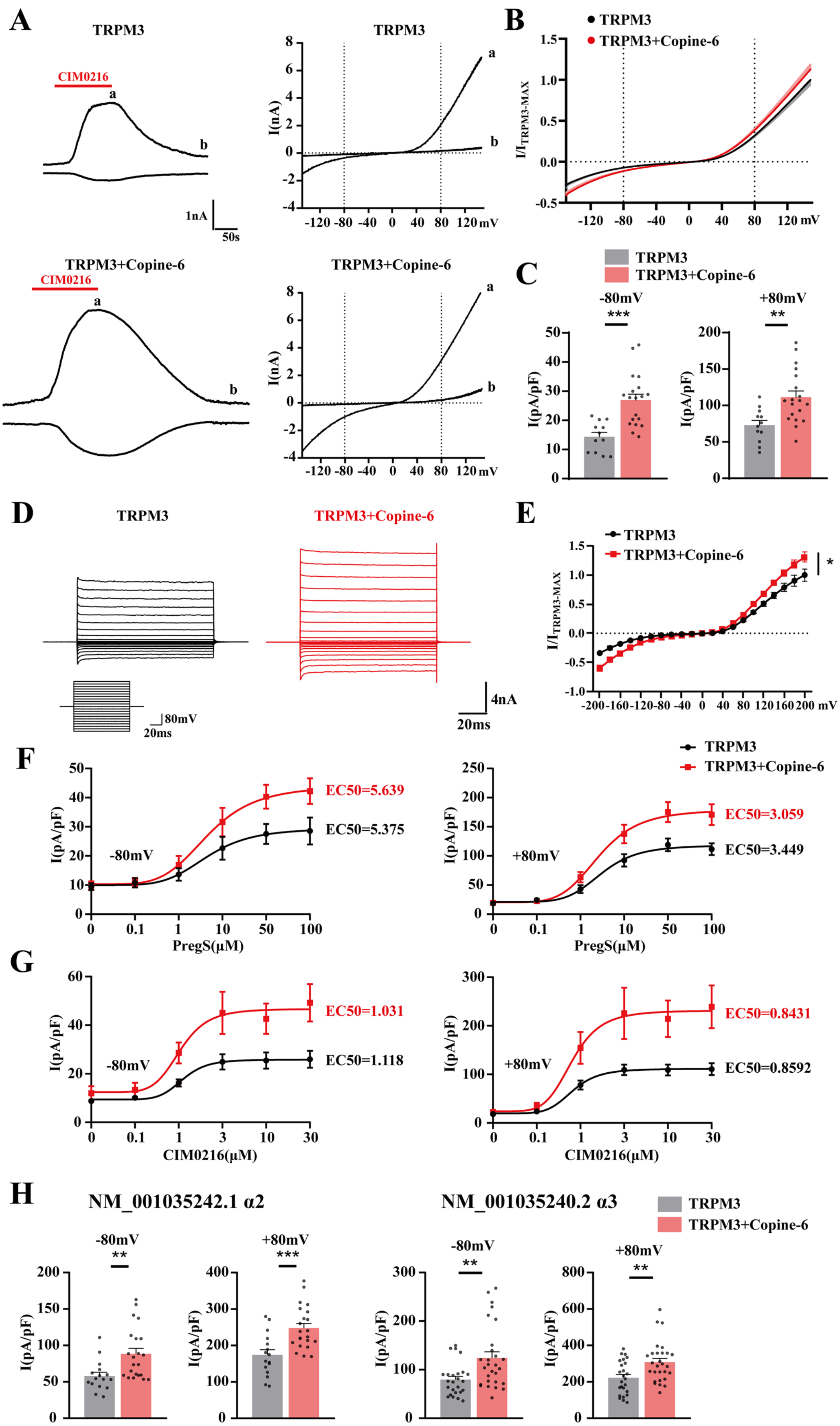
A
TRPM3
CIM0216
a
b
1nA
50s
TRPM3
I(nA)
-120 -80 -40 0 40 80 120 mV
TRPM3+Copine-6
B
TRPM3
TRPM3+Copine-6
I/I TRPM3-MAX
C
-80mV
+80mV
***
**
I(pA/pF)
D
TRPM3+Copine-6
80mV
20ms
4nA
E
-200-160-120-80 -40 0 40 80 120 160 200 mV
*
F
EC50=5.639
EC50=5.375
EC50=3.059
EC50=3.449
PregS(μM)
G
EC50=1.031
EC50=1.118
EC50=0.8431
EC50=0.8592
CIM0216(μM)
H
NM_001035242.1 α2
NM_001035240.2 α3

Figure EV3. Expression of Copine-6 increases TRPM3-mediated currents.

(**A**) Time course (left) and I-V relationship (right) of a whole-cell currents induced by CIM0216 (5 μM) in CHO cells expressing TRPM3 (top) or TRPM3 + Copine-6 (bottom). (**B**) Voltage dependence of CIM0216-induced currents in TRPM3-expressing CHO cells with (red) and without (black) Copine-6 co-expression. Currents were induced by voltage ramps from −150 to +150 mV in 900 ms. Current-voltage relationships normalized to I_{TRPM3} at +150 mV are shown. TRPM3, *n* = 12; TRPM3 + Copine-6, *n* = 19. (**C**) Comparison of 5 μM CIM0216-induced current densities in CHO cells expressing TRPM3 (NM_177341.4/γ3), either alone or together with Copine-6 at −80 or +80 mV. TRPM3, *n* = 12; TRPM3 + Copine-6, *n* = 19; Data are shown as mean ± SEM; −80 mV, ***$P < 0.001$; +80 mV, **$P = 0.003$ (two-tailed independent *t* test). (**D**) Current traces obtained with a voltage step protocol (inset on the bottom-left) in CHO cells transfected with TRPM3 alone (black) or co-transfected with Copine-6 (red) after stimulation by 5 μM CIM0216. (**E**) Voltage dependence of CIM0216-induced currents in TRPM3-expressing CHO cells with (red) and without (black) Copine-6 co-expression. Current-voltage relationships normalized to I_{TRPM3} at +200 mV are shown. TRPM3, *n* = 13; TRPM3 + Copine-6, *n* = 19; Data are shown as mean ± SEM; *$P = 0.0188$ (two-way ANOVA followed by Bonferroni multiple comparisons test). (**F**) Concentration-response curves for Pregnenolone monosulfate sodium (PregS) in CHO cells transfected with TRPM3 alone (black) or co-transfected with Copine-6 (red) at −80 mV (left) and +80 mV (right); recordings as these shown in (**A**). The EC_{50} values are shown next to the curves. Current densities (pA/pF) represent the peak values of the PregS-induced currents. The number of recordings, *n* (from low to high concentration of PregS, respectively): 22, 18, 17, 20 22, and 20 for cells expressing TRPM3 alone (black); 20, 20, 20, 20, 19 and 18 for cells expressing TRPM3 and Copine-6 (red). Data are shown as mean ± SEM. All curves were fitted with Four-Parameter Logistic function. (**G**) Similar to (**F**), but CIM0216 concentration-response curves are plotted. The number of recordings, *n* (from low to high concentration of CIM0216, respectively): 17, 16, 16, 17 15, and 12 for cells expressing TRPM3 alone (black); 17, 17, 17, 14, 13 and 13 for cells expressing TRPM3 and Copine-6 (red). Data are shown as mean ± SEM. (**H**) Comparison of 5 μM CIM0216-induced current densities in CHO cells expressing TRPM3 splice variants (NM_001035242.1/α2 or NM_001035240.2/α3), either alone or together with Copine-6 at −80 or +80 mV. NM_001035242.1/α2: TRPM3, *n* = 16; TRPM3 + Copine-6, *n* = 22; NM_001035240.2/α3: TRPM3, *n* = 25; TRPM3 + Copine-6, *n* = 28. Data are shown as mean ± SEM. NM_001035242.1/α2: -80 mV, **$P = 0.002$; +80 mV, ***$P < 0.0001$. NM_001035240.2/α3: −80 mV, **$P = 0.003$; +80 mV, **$P = 0.0003$ (two-tailed independent Student's t-test). Source data are available online for this figure.

 © The Author(s)

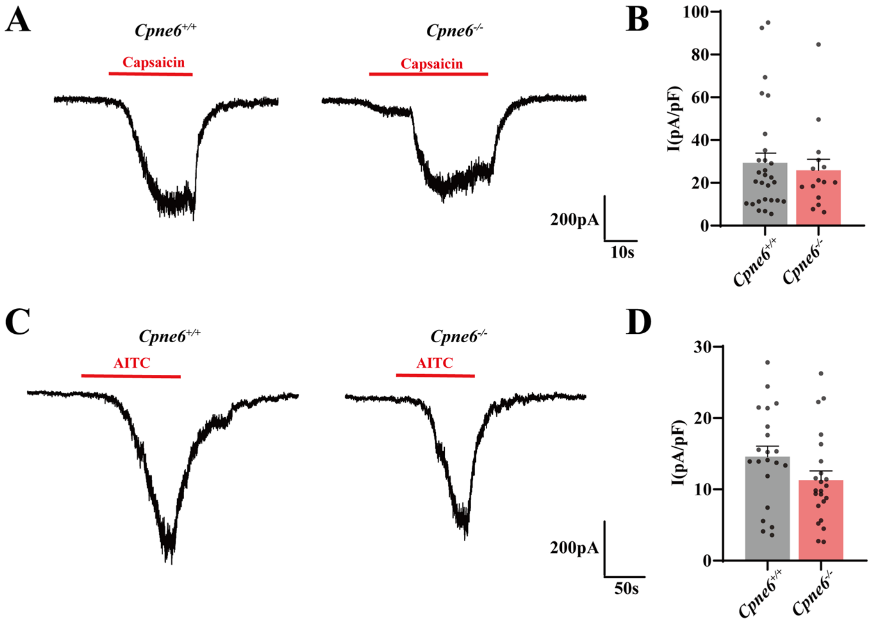

Figure EV4. TRPV1 and TRPA1 currents in DRG neurons of *Cpne6*$^{+/+}$ and *Cpne6*$^{-/-}$ mice.

(**A**) Example current traces evoked by capsaicin (1 μM) in DRG neurons from *Cpne6*$^{+/+}$ (left) or *Cpne6*$^{-/-}$ (right) mice. Recordings were performed using a gap-free protocol at a holding potential of −60 mV. (**B**) Comparison of capsaicin-evoked current densities in DRG neurons from *Cpne6*$^{+/+}$ ($n = 28$) and *Cpne6*$^{-/-}$ ($n = 15$) mice. Data are shown as mean ± SEM. (**C**) Example currents evoked by AITC (100 μM) in DRG neurons from *Cpne6*$^{+/+}$ (left) or *Cpne6*$^{-/-}$ (right) mice. Recordings were performed as in (**A**). (**D**) Comparison of AITC-evoked current densities in DRG neurons from *Cpne6*$^{+/+}$ ($n = 21$) and *Cpne6*$^{-/-}$ ($n = 23$) groups. Data are shown as mean ± SEM. Source data are available online for this figure.

© The Author(s)

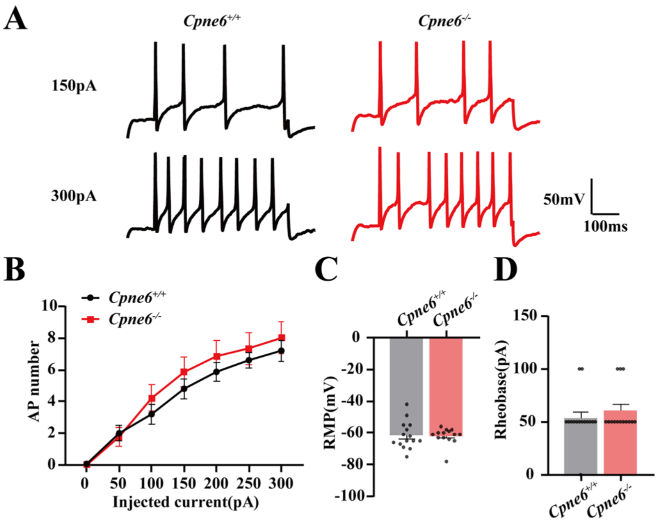

Figure EV5. Copine-6 deficiency has no impact on the general excitability of DRG neurons.

(**A**) Representative current clamp recordings from dissociated DRG neurons from *Cpne6*$^{+/+}$ (left) or *Cpne6*$^{-/-}$ (right) mice. Firing is induced by 500 ms injections of either 150 (top) or 300 pA (bottom) depolarizing currents. (**B**) Summary of the numbers of action potentials elicited by depolarizing current steps from 0 to 300 pA (in 50 pA increments) elicited in DRG neurons from *Cpne6*$^{+/+}$ ($n = 15$) or *Cpne6*$^{-/-}$ ($n = 14$) mice; Data from 3 independent preparations. Recordings were made at room temperature. Data are shown as mean ± SEM. (**C**, **D**) Bar graphs and scatter plots showing the resting membrane potential (RMP; **C**) and rheobase (**D**) of DRG neurons from *Cpne6*$^{+/+}$ ($n = 15$) or *Cpne6*$^{-/-}$ ($n = 14$) mice; data from 3 independent preparations. Data are shown as mean ± SEM. Source data are available online for this figure.

 © The Author(s)
